# Mediation analysis investigating the mechanisms of two school-based smoking prevention interventions in adolescents from Northern Ireland and Bogotá

**DOI:** 10.3389/fpubh.2026.1758481

**Published:** 2026-03-17

**Authors:** Jennifer M. Murray, Sharon C. Sánchez-Franco, Olga L. Sarmiento, Erik O. Kimbrough, Christopher Tate, Shannon C. Montgomery, Rajnish Kumar, Laura Dunne, Allen Thurston, Aideen Gildea, Abhijit Ramalingam, Erin L. Krupka, Felipe Montes, Huiyu Zhou, Laurence Moore, Linda Bauld, Blanca Llorente, Frank Kee, Ruth F. Hunter

**Affiliations:** 1 Centre for Public Health, School of Medicine, Dentistry and Biomedical Sciences, Queen's University Belfast, Belfast, United Kingdom; 2 School of Medicine, Universidad de los Andes, Bogotá, Colombia; 3 Department of Health Promotion, Education, and Behavior, Arnold School of Public Health, University of South Carolina, Columbia, SC, United States; 4 Smith Institute for Political Economy and Philosophy, Chapman University, Orange, CA, United States; 5 College of Education, Health and Human Sciences, Florida State University, Tallahassee, FL, United States; 6 Queen's Business School, Queen's University Belfast, Belfast, United Kingdom; 7 Centre for Evidence and Social Innovation, School of Social Sciences, Education and Social Work, Queen's University Belfast, Belfast, United Kingdom; 8 Centre for Effective Education, School of Social Sciences, Education and Social Work, Queen's University Belfast, Belfast, United Kingdom; 9 School of Nursing and Midwifery, School of Medicine, Dentistry and Biomedical Sciences, Queen's University Belfast, Belfast, United Kingdom; 10 Department of Economics, Appalachian State University, Boone, NC, United States; 11 School of Information, University of Michigan, Ann Arbor, MI, United States; 12 Department of Industrial Engineering, Universidad de los Andes, Bogotá, Colombia; 13 School of Informatics, University of Leicester, Leicester, United Kingdom; 14 MRC/CSO Social and Public Health Sciences Unit, University of Glasgow, Glasgow, United Kingdom; 15 Usher Institute, College of Medicine and Veterinary Medicine, University of Edinburgh, Edinburgh, United Kingdom; 16 Fundación Anáas, Bogotá, Colombia

**Keywords:** adolescents, intervention, low-middle income countries, mediation analysis, norms, prevention, smoking, vaping

## Abstract

**Background:**

Reviews have highlighted a lack of evidence on how successful intervention strategies for adolescent smoking prevention can be effectively adapted for low-middle income countries (LMICs). The MECHANISMS study compared behavioral mechanisms between two school-based smoking prevention programs for adolescents in Northern Ireland (NI; a high-income setting) and Bogotá, Colombia (middle-income). ASSIST works via peer education and diffusion. Dead Cool uses conventional classroom pedagogy. Both interventions were previously trialed in the UK and were culturally adapted for Bogotá. We investigated whether changes in smoking/vaping outcomes differed by intervention or setting. Mediation analyses were conducted to test the hypothesized intervention mechanisms.

**Methods:**

Full school year groups in 12 secondary schools participated during one semester (*n* = 1,344, target age 12–13 years). Outcomes included willingness to pay (WTP) to support anti-smoking norms, self-report and objectively measured smoking behavior, smoking intentions, susceptibility, knowledge, and attitudes. Mediators included injunctive and descriptive smoking/vaping norms, self-efficacy to resist tobacco, perceived risks and benefits, perceived behavioral control, and exposure to advertising. Structural equation models examined intervention and setting effects on outcomes. Multiple mediator models were conducted using the product-of-coefficients approach.

**Results:**

Most significant intervention effects showed more anti-smoking outcomes for Dead Cool compared to ASSIST, although exposure to advertising was lower for ASSIST (media: unstandardized coefficient (*b*) = −1.19, *p* < 0.0001, shops: *b* = −0.14, *p* = 0.04). ASSIST peer supporters also improved their knowledge (*b* = 0.31, *p* = 0.03), self-report smoking behavior (*b* = 0.10, *p* = 0.07), and perceived addiction risks (*b* = 0.31, *p* = 0.01) compared to Dead Cool. Most significant setting effects showed more anti-smoking outcomes for NI versus Bogotá. However, WTP to support anti-smoking norms (*b* = 0.48, *p* = 0.01), self-report descriptive smoking norms (*b* = 0.23, *p* = 0.01), and exposure to advertising in shops (*b* = −0.34, *p* < 0.0001) were more anti-smoking in Bogotá. Several significant mediators showed suppressive mediating effects, suggesting there were important but unmeasured mediators.

**Conclusion:**

Our results suggest school-based programs may be an appealing target for adolescent smoking prevention in LMICs and support using social norms strategies. Future research should identify additional mediating constructs for adolescent smoking prevention in LMICs (integrating intrapersonal, social, environmental, cultural, and political factors), investigate how to optimize the communication channels in peer education and diffusion programs, and provide empirically testable mechanisms.

## 1 Introduction

The *Mechanisms of Networks and Norms Influence on Smoking in Schools* (MECHANISMS) study combines multidisciplinary insights from behavioral economics and game theory, social network and complexity science, health psychology, and public health to investigate the mechanisms of adolescent smoking prevention interventions in Northern Ireland (NI), and Bogotá (Colombia) (1). It is the first study to apply financially incentivized experimental methods from behavioral economics and game theory to measure social norms for adolescent smoking and vaping behaviors (2–4). Unlike the traditional self-report methods commonly used in public health research, these experimental methods mitigate social desirability bias and offer richer insights into intervention mechanisms that may help explain variation in individuals’ health-related behaviors (5, 6). The MECHANISMS study also compared two different types of school-based smoking prevention programs previously shown to reduce adolescent smoking uptake in cluster randomized controlled trials (RCTs) conducted in the United Kingdom (UK): the “A Stop Smoking in Schools Trial” (ASSIST) and *Dead Cool* (7, 8). The programs were culturally adapted and implemented in Bogotá after a rigorous adaptation process (9). In the current paper we compare experimentally measured smoking and vaping norms and other smoking-related outcomes across the two intervention groups and research settings. Mediation analyses are also conducted to test the hypothesized intervention mechanisms (1).

MECHANISMS was not designed to compare the overall effectiveness of the two programs—both of which were previously evaluated in the UK—but rather to focus on the mechanisms underlying behavior change. Detailed assessment of norms, psychosocial and mediating constructs, and mediation analyses were not conducted as part of the original trials. By incorporating these analyses, MECHANISMS extends understanding of how the interventions operate, the pathways through which they influence behavior, and the role of contextual differences between the research settings in influencing the outcomes. The study provided contrasting conditions for examining adolescent smoking and vaping norms, attitudes, and behaviors across two distinct research settings. Variation in social norms, cultural and regulatory environments, health behavior patterns, and smoking prevalence between NI and Bogotá—combined with the contrasting behavioral strategies of the two interventions—offered a unique opportunity to investigate how intervention mechanisms operate across diverse contexts. Whilst both programs may impact school norms around smoking and vaping, smoking behavior, intentions, and attitudes, it is likely that they do so through different causal pathways. Building this knowledge base on intervention mechanisms is essential for effectively adapting and translating intervention programs to new settings (1, 9).

Tobacco consumption remains the leading preventable risk factor for chronic disease and mortality worldwide, accounting for over eight million annual deaths from direct use and passive smoking (10). The burden is particularly severe in low- and middle-income countries (LMICs), where over 80% of the world’s 1.3 billion tobacco users now reside (10). Smoking has been described as a “socially contagious” behavior (11, 12), typically initiated during adolescence when social influences—from observing others’ smoking behaviors and attitudes—and perceived norms are highly salient (11–14). Early prevention is important because young smokers are more likely to become addicted to nicotine and experience long-term health consequences (15, 16). The increasing popularity of electronic nicotine delivery systems (ENDS) and electronic non-nicotine delivery systems (ENNDS)—commonly known as e-cigarettes—has further complicated tobacco control efforts. Large-scale marketing campaigns have accelerated their uptake, particularly among adolescents (17, 18). Young people also tend to experiment with e-cigarettes like traditional cigarettes, and vaping can serve as a gateway to smoking (17, 19, 20). Consequently, the MECHANISMS study investigated social norms related to both smoking and vaping.

School-based interventions are among the most effective strategies for preventing adolescent smoking. Such programs typically target younger adolescents (aged 12–13 years) and often incorporate social influence or social norms approaches (7, 21). Schools are useful settings for delivering norms- and network-based interventions targeting adolescent health behaviors because they reach large adolescent populations and play a key role in shaping adolescent friendship formation and behaviors. Tobacco education also fits naturally into school activities and curricula (21). A 2015 meta-analysis found that school-based programs incorporating social influence and social competence components were effective in reducing smoking uptake (21). However, only four of the 50 included trials were conducted in non-high-income settings, revealing a substantial evidence gap. Other related reviews have also consistently emphasized the lack of rigorous research in LMICs (21–27). One 2012 review of studies in LMICs identified 18 studies targeting smoking prevention amongst schoolchildren. While the findings were inconclusive regarding their efficacy to prevent initial experimentation, the authors concluded that such programs probably reduce progression to regular smoking, improve knowledge, promote attitude change, and enhance refusal skills (23). The review underscored the need for high-quality studies in LMICs, incorporating intervention strategies proven effective in high-income settings, appropriately adapted for the local context and culture (23). Two more recent reviews of school-based smoking prevention programs in LMICs and developing countries identified only seven and 11 eligible studies, respectively. However, they concluded that smoking prevention programs applying anti-smoking curricula, behavior change interventions—particularly those incorporating cultural, environmental, psychological and social factors—and peer education techniques, can be effective when thoroughly culturally adapted and tailored to local contexts (22, 24). Recent research suggests that peer processes in adolescent smoking may vary as tobacco control contexts, public policies, and societal smoking norms change. As tobacco control policies evolve and smoking becomes increasingly denormalized in some societies, understanding the dynamic social and behavioral mechanisms underlying adolescent smoking becomes even more important to effectively adapt interventions to the new conditions (28, 29).

A key feature of the MECHANISMS study is its cross-contextual design. NI is a high-income country in the UK with an adolescent smoking prevalence of approximately 2.2% among those aged 11–16 years (30). Bogotá is the capital city of Colombia, an upper-middle-income country, and has a smoking prevalence of 6.2% among adolescents aged 12–18 years (31). Previous MECHANISMS analyses have already revealed differences between the two settings in social norms (5), socio-environmental and psychosocial predictors of smoking susceptibility (32), and social network processes (33–35). For example, pupils in Bogotá had stronger anti-smoking norms, but greater self-report and objectively measured smoking behavior and more pro-smoking intentions than those in NI at baseline (5). Our qualitative findings also uncovered contextual differences in how the smoking prevention interventions operated. In NI, having previous information about tobacco through schools or youth clubs was a protective factor, whilst greater access to tobacco products in local neighborhoods was a risk factor for smoking intentions and uptake in Bogotá (36).

MECHANISMS’s smoking prevention programs are grounded in different behavior change theories and use different intervention strategies (1). ASSIST is based on the *diffusion of innovations theory* (37), and is designed to leverage peer influences by training influential pupils within school year groups (“peer supporters”) to use informal contacts with their school peers to promote anti-smoking norms (7). Dead Cool is a teacher-led, skills-focused program based on the *theory of planned behavior* (38). It aims to increase pupils’ knowledge of potential influences on smoking behavior—including family, friends, and media influences—while developing personal and social skills to resist smoking (8, 39). Previous MECHANISMS work has already highlighted several differences in how the two interventions operated. Social network analyses revealed that peer influence and selection homophily processes explained a greater proportion of smoking or vaping similarity among friends in ASSIST schools compared to Dead Cool (33–35). Our qualitative findings also highlighted similarities and differences in participants’ experiences. Pupils in both programs reported enhanced knowledge about health consequences, increased self-efficacy to avoid smoking or vaping, and greater awareness of social influences. ASSIST participants more frequently discussed knowledge of cigarette ingredients, communication skills, and encouraging others not to smoke, whereas Dead Cool participants emphasized personal refusal skills and awareness of advertising influences. Moreover, the ASSIST peer supporters—who were trained to spread anti-smoking messages—demonstrated unique outcomes, as they discussed developing personal skills in their roles as health promoters more often than their untrained peers (36). Therefore, the present study also compares ASSIST peer supporters (ASSIST PS) and non-peer supporters (ASSIST non-PS) separately with Dead Cool participants.

Health behavior interventions like ASSIST and Dead Cool are complex, involving multiple interacting components, and various behavior change techniques (BCTs) (40, 41). BCTs are the irreducible “active ingredients” of complex interventions that target specific mechanisms regulating behavior (40). Identifying which components of complex interventions are effective—and which are not—is essential for understanding how interventions work, refining theoretical models, and improving future program design (40). Mediation analysis provides a valuable method for identifying effective components in complex interventions by quantifying indirect effects through psychological, social, or environmental mediators situated along the causal pathway between an exposure (e.g., intervention type or setting) and an outcome (e.g., smoking behavior) (42). That is, mediators can help explain some of an intervention’s effect on an outcome. For example, in the Project MYTRI study (“Mobilizing Youth for Tobacco-Related Initiatives in India”), knowledge about health effects, perceived social consequences, reasons for tobacco use or non-use, advocacy skills self-efficacy, and normative beliefs about tobacco were important mediators linking intervention exposure to reduced tobacco use intentions and behaviors (43, 44).

Logic models describing the hypothesized pathways of ASSIST and Dead Cool were published in the MECHANISMS study protocol (1). Using the Michie et al. (40) BCT taxonomy, we coded the BCTs included in each intervention from the intervention manuals and resources, mapping them onto targeted mediating constructs. The logic models proposed that both programs would lead to reduced smoking behavior, intentions and susceptibility, and increased knowledge of smoking and anti-smoking attitudes, via changes in hypothesized mediating variables such as norms, self-efficacy to resist tobacco, perceived risks and benefits, perceived behavioral control (PBC), and exposure to tobacco advertising (1). MECHANISMS participants completed behavioral economics experiments measuring social norms, self-report surveys, and objective carbon monoxide measures before and after receiving the interventions over one school semester (1).

In the present paper, we apply confirmatory factor analysis (CFA) and structural equation modelling (SEM) to examine differences in outcomes at follow-up adjusted for baseline, between ASSIST and Dead Cool schools and between NI and Bogotá. We also conduct mediation analyses to test the hypothesized causal pathways defined in our logic models, exploring whether intervention or setting effects on key outcomes—willingness to pay (WTP) to support anti-smoking norms, self-report and objectively measured smoking behavior, intentions, susceptibility, knowledge, and attitudes—are mediated by any of the proposed constructs. Since the intervention mechanisms may differ for the ASSIST PS, who received specialized training to diffuse anti-smoking messages, analyses are repeated comparing ASSIST PS and ASSIST non-PS separately with Dead Cool participants.

Although MECHANISMS was designed to be mechanisms-focused rather than offering a randomized head-to-head comparison of ASSIST and Dead Cool—both of which were previously evaluated in the UK (1, 7, 8)—we also conducted a sensitivity analysis using data from the control group of the original Dead Cool trial. This provided additional contextual comparison for several outcomes which were measured in both studies, including self-report and objectively measured smoking behavior, smoking intentions and susceptibility, and exposure to tobacco advertising (8, 39, 45). In presenting these analyses, we emphasize that the 2014 cohort cannot serve as a contemporaneous counterfactual for the MECHANISMS sample. Firstly, schools were not randomized between the MECHANISMS interventions versus the 2014 study’s control group. Secondly, broader population-level trends in adolescent smoking and e-cigarette use changed substantially between 2014 and 2019. For example, adolescent smoking rates in NI decreased from 5.0% in 2013 to 3.9% in 2019, whilst e-cigarette users increased from 4.9% in 2016 to 5.7% in 2019 (46). Patterns of use also shifted, with vaping increasingly acting as a precursor to cigarette smoking by 2019 (47). However, the purpose of incorporating the historical control group was not to estimate causal intervention effects, but to provide a benchmark for the short-term, within-cohort changes that might be expected over a school semester among similarly aged pupils who were not exposed to either intervention. All schools participating in MECHANISMS in 2019 received either the ASSIST or Dead Cool smoking prevention interventions, both of which were designed to target smoking-related outcomes and mediators, and no untreated comparison group was included in the study (1). For those outcomes and mediators that were measured identically in both studies, the separate 2014 cohort offers a useful reference point for understanding the expected direction and magnitude of short-term within-cohort changes. This helps to contextualize whether the observed changes in MECHANISMS are plausibly attributable to intervention processes or may reflect typical within-cohort developmental shifts in adolescent smoking-related behaviors. While we interpret these comparisons cautiously, the historical control data strengthens the interpretability of our findings by illustrating how key outcomes typically change in the absence of intervention exposure. Together, these analyses enable us to move beyond traditional effectiveness evaluations, offering new evidence on how school-based smoking prevention programs influence adolescent social norms, mediators, and behaviors across diverse cultural and policy contexts.

## 2 Methods

### 2.1 Study design

#### 2.1.1 MECHANISMS study

Twelve schools (six in NI and six in Bogotá, participation rate = 93.1%, *n* = 1344/1444 pupils) took part in the MECHANISMS study between January and November 2019 (1). Study procedures are described in detail in the study protocol and related publications (1, 5, 33). Schools were recruited throughout NI and Bogotá, targeting full school year groups aged 12–13 years (Year 9 in NI, Year 7 in Bogotá). Using a pre-post quasi-experimental study design, schools were randomized to ASSIST or Dead Cool (7, 8). Pupils completed financially incentivized norms elicitation experiments based on behavioral economics and game theory (2–4), and a self-report survey over one school semester (approximately ten weeks). Monetary payments for the experiments were provided in cash in NI and via gift cards in Bogotá.

The study was implemented in Bogotá following a comprehensive cultural adaptation process, which involved translation and back-translation of all study materials, intervention content, surveys, experiments, information sheets, and consent forms into Spanish. The cultural adaptation procedures used in MECHANISMS are described in detail in a previous publication (9).

Ethical approval was granted by the School of Medicine, Dentistry and Biomedical Sciences Ethics Committee at Queen’s University Belfast in NI (ref. 18:43, September 2018) and the Research Ethics Committee at Universidad de los Andes in Bogotá (ref. 937/2018, July 2018). All schools received teacher, pupil, and parent/guardian information sheets, pupil consent forms, and parent/guardian opt-out forms. Pupils provided written informed consent after reading the information sheet and having the opportunity to ask questions. Parents or guardians who did not wish their child to participate returned a completed opt-out form. In Bogotá, parental opt-in consent was required, with written informed consent collected from parents/guardians at the start of the study. In Bogotá, content and delivery of the study’s consent procedure, information sheets and consent forms were adapted as needed according to the requirements of the local Research Ethics Committee and local legislation on research involving human subjects. Information sheets in Bogotá were delivered in person to parents, teachers, and school administrators, and questions were addressed through multiple in-person meetings prior to collecting informed consent. Game theory earnings were paid using gift cards instead of cash in Bogotá. These procedures were fully approved by the respective ethics committees at each institution and complied with local legislation on research involving human participants.

All data collection procedures followed institutional guidelines for research involving human participants. Experiments and surveys were delivered on tablet computers using Qualtrics (Qualtrics, Provo, Utah, United States), and participants were instructed not to communicate with each other during data collection. During the cultural adaptation, all measurement tools were precisely translated into Spanish and back-translated into English. The content and delivery of the instruments were adapted as needed to ensure conceptual equivalence across settings and cultural appropriateness. All measurement instruments were pre-tested in a pilot study conducted before the full phase of the MECHANISMS study, involving three schools with 239 pupils in Bogotá and 73 pupils in NI. Full pilot results are reported in the cultural adaptation paper (9).

Given the international, multi-centre nature of MECHANISMS, several steps were taken to ensure standardized training and rigorous data-collection procedures across NI and Bogotá. The Bogotá research team took part in a study visit to NI in September 2018, during which both teams received joint training on the epistemology and implementation procedures of the intervention programs. Quarterly study meetings were held with the NI and Bogotá teams and co-investigators across the UK, USA, and Colombia. To avoid introducing bias, it was especially important to standardize the delivery and procedures used for the behavioral economics experiments. The quarterly meetings were particularly important for ensuring that the behavioral economics experiments were implemented using consistent protocols across the settings, with guidance provided by the study’s behavioral economics experts. Additional online meetings between the NI and Bogotá teams and the game theory specialists supported the study preparation and fieldwork. Following completion of the NI pilot, the NI team visited the Bogotá campus to review the pilot findings and to further refine and standardize the data collection procedures for the Bogotá pilot and the full study phase in both settings.

Further details on study procedures, including the study flow diagram and baseline characteristics, are provided in the Supplementary Methods, Supplementary Figure S1, and Supplementary Table S1.

#### 2.1.2 Control group from the original Dead Cool trial (Northern Ireland control group)

Procedures for the original Dead Cool RCT have been previously published (8, 39, 45). Ten classes across ten schools were randomized to the control arm (*n* = 235 pupils). Schools were located within a 30-mile radius of Belfast city centre, and Year 9 classes (target age 12–13 years) were recruited in October 2014. Baseline data collection occurred in November and December 2014, followed by a post-test in February 2015 (8, 39, 45).

Control schools received treatment-as-usual, and therefore continued with their standard, legally required, smoking prevention curriculum, as it was deemed ethically inappropriate to suspend the current practice (8, 39, 45). The study was approved by the School of Social Sciences, Education and Social Work Ethics Board, Queen’s University Belfast. Information sheets were provided to all participants, and opt-out consent was obtained from pupils, teachers, and senior management teams (8, 39, 45).

### 2.2 Settings

#### 2.2.1 Northern Ireland

NI is a high-income country in the UK (48), with a population of approximately two million (49). In 2022, current cigarette consumption rates among NI adolescents aged 11–16 years were 2.2%, with 1.0% reporting smoking at least once a week and 7.6% reporting having smoked tobacco at least once. Current e-cigarette use was 9.2%, with 6.3% reporting vaping at least once a week and 21.3% reporting having ever used an e-cigarette (30). In the UK, it is illegal to sell tobacco products to, or purchase them on behalf of, minors under 18 years of age (50). Tobacco education is a formal part of the UK school curriculum (51). The UK also has comprehensive tobacco control legislation regulating advertising, sale to minors, packaging, and smoking in public and indoor places (52–54). The sale of e-cigarettes to under 18 s is prohibited in the UK. While the government has sought to maximize the potential of e-cigarettes as a smoking cessation aid for adults, stricter legislation is currently being introduced to tighten regulations around sales to minors, extend indoor smoking bans to include vaping, and prohibit advertising and sponsorship of e-cigarettes and other nicotine products, in line with the existing tobacco control regulations (55, 56).

#### 2.2.2 Bogotá

Bogotá is the capital city of Colombia, an upper-middle-income country (57), with a population of over seven million (58). In 2022, current cigarette consumption rates among Colombian adolescents aged 12–18 years were 4.5%, with 11.1% reporting having tried tobacco or cigarettes at least once. In Bogotá, the corresponding figures were higher with 6.2% reporting current smoking. Current e-cigarette use across Colombia was 11.2 and 22.7% reported having used an e-cigarette at least once. In Bogotá, current e-cigarette use was slightly higher than the national average at 12.1% (31). Colombia adopted the World Health Organization’s Framework Convention on Tobacco Control (WHO-FCTC) in 2009, enacting legislation to regulate tobacco advertising, packaging, sales to minors, and smoke-free public places (59). Selling tobacco products to under 18 s is illegal, and generally well enforced within the regulatory sphere with police monitoring of commercial establishments. The legislation includes a complete ban on tobacco advertisements, sponsorships, and promotions, with high levels of implementation across television, cinemas, and public signage. However, adolescents may still access tobacco products through informal channels, such as contraband sales or street vendors (60). Latin American countries have historically been vulnerable to the effects of the tobacco epidemic, and smoking has been integrated into their culture and customs (61). The Colombian government has also faced implementation challenges related to tobacco control, including tobacco industry resistance to the WHO-FCTC measures (61, 62). At the time of MECHANISMS data collection, e-cigarettes were not covered under Colombia’s tobacco control legislation and remained unregulated until 2024, when the tobacco control law was extended to regulate ENDS and nicotine substitutes (63).

### 2.3 Interventions

The ASSIST and Dead Cool programs were previously evaluated in cluster RCTs in the UK and showed effectiveness for reducing adolescent smoking uptake (7, 8). Detailed intervention descriptions and logic models outlining the hypothesized pathways leading to changes in smoking behavior, intentions, knowledge, and attitudes were published in the study protocol and are reproduced in Supplementary Figures S2, S3 (1). BCTs were coded from the intervention manuals and resources according to the 93-item taxonomy developed by Michie et al. (40). Supplementary Table S2 provides an overview of the included BCTS with examples from each intervention and their targeted mediating constructs.

#### 2.3.1 The Dead Cool program

Dead Cool is based on the theory of planned behavior (38, 64) and is underpinned by conventional classroom pedagogy. It focuses on developing pupils’ knowledge and practical skills to recognize and resist social and environmental influences for smoking, supporting them to remain smoke-free. The program emphasizes skills like managing information, teamwork, problem-solving, decision-making, self-management, creativity, persuasive communication, refusal tactics, positive self-talk, negotiation, and visualization to achieve goals. The program also aims to increase pupils’ awareness of how friends, parents, family members, the media, and social media can influence smoking behavior (8, 39, 45).

Schoolteachers in NI and facilitators in Bogotá received program training from an employee of Cancer Focus NI, the organization responsible for the program. Each school year group then received an introductory session, followed by eight weekly 60-min lessons delivered by the trained schoolteachers or facilitators. At the start of each lesson, pupils watched short DVD clips featuring children of similar ages discussing smoking-related issues, or role-playing scenarios about resisting peer pressure to smoke. All videos were dubbed by local Spanish speakers for Bogotá. PowerPoint slides were used to guide discussions on issues like the health, social, and emotional consequences of smoking, the pros and cons of using various refusal tactics, reasons people may choose to smoke, case studies illustrating negotiation skills for avoiding passive smoking, and smoking influences in the media. Pupils completed written and group activities, such as signing a “Self-Promise Contract” to remain smoke-free, identifying effective refusal tactics, taking notes from discussions, practicing use of persuasive argument to debunk myths about smoking, role-playing peer pressure scenarios, and using positive self-talk to affirm non-smoking goals. The program concluded with group presentations where pupils demonstrated the knowledge and skills they had developed to help them avoid smoking.

Teachers received a Teachers’ Resource Pack containing lesson guides, PowerPoint slides, DVDs, and fact files. Pupils received Pupil Workbooks containing exercises for each lesson. BCTs were coded in relation to the target behavior, “smoking prevention.” Before the MECHANISMS study, Dead Cool was updated from its original format of an introductory session and four lessons (8, 39, 45), to an expanded eight-lesson version with additional BCTs. Supplementary Table S2 details the BCTs included in both the original and updated versions, and BCTs which were unique to the updated program.

#### 2.3.2 The ASSIST program

The ASSIST program is based on the diffusion of innovations theory (37), and uses a peer education and diffusion model. The program identifies the most influential pupils within school year groups—nominated through a “peer questionnaire” completed by all pupils at baseline—and trains them to act as peer supporters who use informal conversations to promote non-smoking among their school peers (7, 65, 66).

The top 18% of nominated pupils attended a peer supporter recruitment meeting where they were invited to a two-day training course to help them develop the skills to informally encourage non-smoking among their peers. If the peer supporters were current smokers, they were encouraged to quit. The recruitment meeting aimed to recruit 15% of the school year group. Day one of the training focused on increasing peer supporters’ knowledge about the health, economic, social, and environmental risks of smoking, and the benefits of staying smoke-free. Day two focused on skills development (e.g., communication, listening, teamwork, negotiation, conflict resolution, and prioritization), and personal development areas (e.g., confidence, self-esteem, empathy, assertiveness, decision-making, risk-taking attitudes, and exploring personal values). The training included various learning activity styles including role-play, student-led research, small group activities, discussions, and games. The trainers used social and physical rewards (e.g., praise, playing games, small prizes) to boost the peer supporters’ confidence, and reinforce engagement and commitment (7).

After training, the peer supporters were asked to have informal conversations with their school friends to encourage non-smoking over a ten-week period, using accurate information learned at the training course. They were asked to keep a diary record of these conversations. The ASSIST trainers made four follow-up visits to each school—each lasting approximately 60 min—to monitor progress and provide further training. Peer supporters received a certificate recognizing their contribution and skills at the end of the program (7). The ASSIST trainers were employees of Queen’s University Belfast, the Public Health Agency NI, and Universidad de los Andes, who received formal training from Evidence to Impact, the organization responsible for implementing ASSIST.

According to the diffusion of innovations theory (37, 67), the four key components driving the diffusion of an innovation include:

1. The innovation (the smoking prevention message).
2. The communication channels (informal conversations between peer supporters and their school friends).
3. Time (the ten-week intervention period when the smoking prevention message is diffused).
4. The social system (the school year group).

Successful adoption of the innovation relies on individuals being well informed about its advantages and disadvantages (37, 67). This highlights that the communication channels are vital to the success of the intervention. Therefore, BCTs were coded in relation to both “smoking prevention” and “being an effective peer supporter” (Supplementary Table S2).

#### 2.3.3 Cultural adaptation of the intervention programs and implementation fidelity in Bogotá

Since ASSIST and Dead Cool were both originally developed and tested in the UK (7, 8), a comprehensive cultural adaptation process was undertaken to ensure their relevance and appropriateness for Bogotá while maintaining fidelity to their core components (9). Led by staff at Universidad de los Andes, this process included translation and back-translation of all materials into Spanish, adaptation of delivery formats, venues, and content to align with local norms, school structures, and Colombian tobacco-control regulations, and iterative refinements based on feedback from pupils, teachers, and practitioners during pilot testing. Key adaptations included dubbing Dead Cool’s video materials with Colombian voice actors, modifying content to comply with national guidelines prohibiting depictions of smoking (68), relocating the ASSIST peer supporter training to university facilities, and employing trained practitioners to deliver Dead Cool due to teacher workload constraints. Throughout the study, fidelity and participant engagement were monitored to ensure that the interventions’ core mechanisms were preserved. Full details of the cultural adaptation procedures are reported in Sánchez-Franco et al. (9).

To assess implementation fidelity, the original program developers visited Bogotá and NI to observe delivery of selected activities and provided feedback during the pilot phase. Three intervention sessions for each program were also videotaped to assess intervention fidelity. In each setting, the original program developers provided a positive score on the fidelity assessment for both programs. Since the programs were culturally adapted and implemented in Bogotá for the first time, additional fidelity-monitoring strategies were used in Bogotá, including self-reported checklists and fieldnotes for each activity. The self-reported fidelity monitoring showed that 86% of ASSIST activities and 88% of Dead Cool activities were delivered in line with the protocol guides. The qualitative analysis showed that some delivery challenges were largely related to contextual factors such as time constraints and availability of technical resources within schools. These were managed by the trained practitioners, who ensured that all planned content and themes were ultimately covered (9).

### 2.4 Incentivized experiments

The game theory and behavioral economics experiments included financially incentivized tasks based on published behavioral economics methodologies (2–4). Part 1 included a rule-following task measuring each participant’s individual sensitivity to the effects of social norms (2, 3).

Parts 2 and 3 included financially incentivized *“co-ordination”* games designed to measure injunctive and descriptive social norms for smoking and vaping behaviors within entire school year groups (4). Participants were provided with financial incentives to *match* their responses to other pupils’ in their school year group instead of providing their personal opinions. Specifically, the participants were told they would receive a payment if their answer to a randomly chosen question matched the most common response in their school year group. This encouraged them to consider how most others in their year group would respond, thus revealing the prevailing norm. Injunctive norms—shared beliefs about what people *ought to do*—were measured as shared perceptions within school year groups about the social appropriateness of various behaviors. Descriptive norms—shared beliefs about what people *actually do*—were measured as shared perceptions within school year groups about the extent to which smoking and vaping behaviors are accepted by others (4).

In Part 2, experimentally measured injunctive smoking/vaping norms were assessed by asking participants to *co-ordinate* with others in their school year group to rate the social appropriateness of eight situations involving smoking and vaping behaviors (outcomes P2S2 to P2S9). Pupils provided their ratings on a six-point scale (“extremely socially inappropriate” to “extremely socially appropriate”).

In Part 3, experimentally measured descriptive smoking/vaping norms were assessed by asking participants to *co-ordinate* with others in their school year group to estimate the proportion of their school year group who would be accepting of a close friend smoking or vaping (outcomes P3Q1 and P3Q2). Pupils provided their ratings on a six-point scale (*“none of my peers”* to *“all of my peers”*). We have previously demonstrated the construct validity of these novel experimental measures of adolescent smoking/vaping norms, showing that they index similar underlying phenomena as established self-report measures (5).

Part 4 assessed pupils’ WTP to support prevention interventions promoting anti-smoking norms. Participants received ten virtual tokens of equal monetary value and were asked how many they wanted to donate to the smoking prevention program delivered in their school, described as *“a smoking prevention program which aims to prevent the uptake of smoking among adolescents your age.”* They were informed that they would receive a payment equal to the number of tokens not donated. A higher donation was interpreted as a greater willingness to incur a personal cost to support the promotion of anti-smoking norms (outcome “Donation to ASSIST/Dead Cool,” rated 0 to 10 tokens donated). Since a donation can be viewed as expressing a belief that smoking prevention programs are both normatively appealing and effective, this task provided behavioral evidence of injunctive anti-smoking norms (1).

Participants were informed that they would receive a participation fee of £5.00 (NI) or *COP*$5.000 (Bogotá), and could earn money in each part of the experiment (maximum £30 in NI or *COP*$50.000 in Bogotá), depending on their own and others’ responses within their school year group. Payments were made after the follow-up experiment, based on randomly selected questions from each part of the experiment at either baseline or follow-up. Table 1 and Supplementary Table S3 lists the assessed smoking- and vaping-related scenarios. More information on the experiments’ theoretical background and full experimental protocols are provided in the Supplementary Methods.

**Table 1:** Baseline and follow-up summary statistics (means and standard deviations unless otherwise stated).

Outcome	ASSIST schools (*N* = 6)		Dead Cool schools (*N* = 6)		Northern Ireland MECHANISMS schools (*N* = 6)		Bogotá MECHANISMS schools (*N* = 6)		Northern Ireland control schools (*N* = 10)		Test for baseline differences^a^^,^^b^		Direction^c^
	Baseline	Follow-up	Baseline	Follow-up	Baseline	Follow-up	Baseline	Follow-up	Baseline	Follow-up	Intervention	Setting	
Experiment, *n*	692	691	579	560	625	620	646	631	Not conducted.		N/A		N/A
Survey, *n*	702	658	572	551	630	590	644	619	220	207			
Carbon monoxide readings, *n*	668	667	571	544	591	591	648	620	200	172			
Experiment part 2: injunctive social norms (*α* = 0.78; −1 = “extremely socially inappropriate” to +1 = “extremely socially appropriate”)													
P2S2: Parent smoking in their own home in front of children under age of 5.	−0.9 (0.3)	−0.8 (0.3)	−0.9 (0.2)	−0.9 (0.3)	−0.8 (0.3)	−0.8 (0.4)	−0.9 (0.2)	−0.9 (0.2)	Not measured.		A vs. DC: *p* = 0.82	**NI vs. Bog: *p*** **≤** **0.0001**^**e**^	−ve
P2S3: An adult smoking in a car with children under the age of 16 in the car.	−0.7 (0.4)	−0.7 (0.4)	−0.7 (0.3)	−0.7 (0.3)	−0.7 (0.4)	−0.7 (0.4)	−0.7 (0.3)	−0.7 (0.3)			A vs. DC: *p* = 0.12	NI vs. Bog: *p* = 0.09	−ve
P2S4: Someone selling cigarettes to a teenager who looks younger than 16 without requesting proof of age.	−0.8 (0.4)	−0.8 (0.4)	−0.9 (0.3)	−0.8 (0.3)	−0.9 (0.3)	−0.8 (0.3)	−0.9 (0.3)	−0.8 (0.3)			**A vs. DC: *p* = 0.002**	NI vs. Bog: *p* = 0.99	−ve
P2S5: In a recent superhero movie the lead actor is seen smoking in the opening scene.	−0.4 (0.5)	−0.3 (0.4)	−0.4 (0.4)	−0.3 (0.4)	−0.3 (0.4)	−0.3 (0.4)	−0.4 (0.4)	−0.4 (0.4)			A vs. DC: *p* = 0.90	**NI vs. Bog: *p*** **≤** **0.0001** ^**e**^	−ve
P2S6: An older student from school is smoking outside school, for example, at a bus stop.	−0.5 (0.4)	−0.4 (0.4)	−0.6 (0.4)	−0.5 (0.4)	−0.5 (0.4)	−0.5 (0.4)	−0.5 (0.4)	−0.5 (0.4)			**A vs. DC: *p* = 0.0003** ^**e**^	NI vs. Bog: *p* = 0.14	−ve
P2S7: A pupil from school is using an e-cigarette while walking to school.	−0.5 (0.4)	−0.4 (0.4)	−0.6 (0.4)	−0.5 (0.4)	−0.5 (0.4)	−0.5 (0.4)	−0.5 (0.4)	−0.5 (0.4)			**A vs. DC: *p* = 0.0002** ^**e**^	NI vs. Bog: *p* = 0.23	−ve
P2S8: A pupil from school shares a photograph of him/herself using an e-cigarette on social media.	−0.5 (0.4)	−0.4 (0.4)	−0.5 (0.4)	−0.5 (0.4)	−0.5 (0.4)	−0.5 (0.4)	−0.4 (0.4)	−0.4 (0.4)			**A vs. DC: *p* = 0.01**	**NI vs. Bog: *p* = 0.0004** ^**e**^	−ve
P2S9: A pupil from school is chewing tobacco.	−0.8 (0.3)	−0.7 (0.4)	−0.8 (0.3)	−0.7 (0.3)	−0.8 (0.4)	−0.7 (0.4)	−0.8 (0.3)	−0.7 (0.3)			A vs. DC: *p* = 0.60	**NI vs. Bog: *p* = 0.05**	−ve
Experimental injunctive smoking/vaping norms scale (average P2S2 to P2S9).	−0.6 (0.3)	−0.6 (0.3)	−0.7 (0.2)	−0.6 (0.2)	−0.6 (0.3)	−0.6 (0.3)	−0.7 (0.2)	−0.6 (0.2)			**A vs. DC: *p* = 0.004**	NI vs. Bog: *p* = 0.09	−ve
Experimental injunctive smoking norms scale (average P2S2 to P2S6 and P2S9).	−0.7 (0.2)	−0.6 (0.2)	−0.7 (0.2)	−0.7 (0.2)	−0.7 (0.2)	−0.6 (0.2)	−0.7 (0.2)	−0.7 (0.2)			**A vs. DC: *p* = 0.04**	**NI vs. Bog: *p* = 0.002**	−ve
Experimental injunctive vaping norms scale (average P2S7 to P2S8).	−0.5 (0.4)	−0.4 (0.4)	−0.5 (0.4)	−0.5 (0.3)	−0.5 (0.4)	−0.5 (0.4)	−0.5 (0.4)	−0.5 (0.4)			**A vs. DC: *p* = 0.0006**	NI vs. Bog: *p* = 0.17	−ve
Experiment part 3: descriptive social norms (α = 0.85; −1 = “none of my peers” to +1 = “all of my peers”)													
P3Q1: Proportion of school year group accepting of a close friend smoking.	−0.4 (0.5)	−0.3 (0.5)	−0.5 (0.4)	−0.4 (0.5)	−0.5 (0.5)	−0.3 (0.5)	−0.5 (0.5)	−0.3 (0.5)	Not measured.		**A vs. DC: *p*** **≤** **0.0001** ^**e**^	**NI vs. Bog: *p* = 0.03**	−ve
P3Q2: Proportion of school year group accepting of a close friend vaping.	−0.3 (0.6)	−0.2 (0.6)	−0.4 (0.5)	−0.2 (0.5)	−0.3 (0.6)	−0.2 (0.6)	−0.4 (0.5)	−0.3 (0.6)			**A vs. DC: *p*** **≤** **0.0001** ^**e**^	**NI vs. Bog: *p* = 0.0003** ^**e**^	−ve
Experimental descriptive smoking/vaping norms scale (average P3Q1 to P3Q2).	−0.4 (0.5)	−0.3 (0.5)	−0.5 (0.4)	−0.3 (0.5)	−0.4 (0.5)	−0.3 (0.5)	−0.5 (0.5)	−0.3 (0.5)			**A vs. DC: *p*** **≤** **0.0001** ^**e**^	**NI vs. Bog: *p* = 0.002**	−ve
Experiment part 4: willingness to pay to support anti-smoking norms (0 = “0 tokens donated to ASSIST/Dead Cool” to 10 = “10 tokens donated to ASSIST/Dead Cool”)													
Donation to ASSIST/Dead Cool (0 to 10).	3.6 (2.8)	3.4 (2.6)	3.9 (2.9)	3.2 (2.7)	3.5 (3.1)	3.0 (2.8)	3.9 (2.6)	3.6 (2.4)	Not measured.		**A vs. DC: *p* = 0.05**	**NI vs. Bog: *p* = 0.008**	+ve
Survey: injunctive social norms (α = 0.75; −2 = “think(s) that I definitely should smoke” to +2 = “think(s) that I definitely should not smoke”)													
IN1: Most of the people who are important to me.	1.8 (0.7)	1.7 (0.7)	1.7 (0.7)	1.7 (0.7)	1.7 (0.7)	1.7 (0.7)	1.8 (0.7)	1.7 (0.8)	Not measured.		A vs. DC: *p* = 0.78	NI vs. Bog: *p* = 0.48	+ve
IN2: Mother.	1.9 (0.4)	1.9 (0.5)	1.9 (0.3)	1.9 (0.4)	1.9 (0.3)	1.9 (0.4)	1.9 (0.4)	1.9 (0.5)			A vs. DC: *p* = 0.13	NI vs. Bog: *p* = 0.11	+ve
IN3: Father.	1.7 (0.7)	1.7 (0.7)	1.8 (0.6)	1.8 (0.6)	1.8 (0.6)	1.8 (0.6)	1.7 (0.7)	1.7 (0.7)			**A vs. DC: *p* = 0.001**	**NI vs. Bog: *p*** **≤** **0.0001** ^**e**^	+ve
IN4: Brother(s).	1.3 (0.9)	1.4 (0.9)	1.4 (0.9)	1.4 (0.9)	1.4 (0.9)	1.4 (0.9)	1.4 (0.9)	1.5 (0.8)			**A vs. DC: *p* = 0.05**	NI vs. Bog: *p* = 0.78	+ve
IN5: Sister(s).	1.3 (1.0)	1.4 (0.9)	1.4 (0.9)	1.4 (0.9)	1.4 (0.9)	1.4 (0.9)	1.3 (0.9)	1.4 (0.8)			**A vs. DC: *p* = 0.03**	**NI vs. Bog: *p* = 0.05**	+ve
IN6: Friends.	1.4 (0.9)	1.4 (1.0)	1.4 (0.9)	1.5 (0.9)	1.5 (0.9)	1.5 (0.9)	1.3 (0.9)	1.3 (0.9)			A vs. DC: *p* = 0.22	**NI vs. Bog: *p* = 0.003**	+ve
IN7: Best friend.	1.5 (0.8)	1.5 (0.9)	1.6 (0.8)	1.6 (0.7)	1.7 (0.7)	1.7 (0.8)	1.5 (0.9)	1.5 (0.9)			A vs. DC: *p* = 0.18	**NI vs. Bog: *p*** **≤** **0.0001** ^**e**^	+ve
Self-report injunctive smoking norms scale (average IN1 to IN7).	1.6 (0.5)	1.6 (0.5)	1.6 (0.5)	1.6 (0.5)	1.6 (0.5)	1.6 (0.5)	1.5 (0.5)	1.6 (0.5)			**A vs. DC: *p* = 0.008**	**NI vs. Bog: *p* = 0.0007**	+ve
Survey: descriptive social norms 1 (α = 0.54; 1 = “smoke(s) very often” to 5 = “never smoke(s)”/“do not know”)													
DN1.1: Best friend.	4.7 (0.8)	4.7 (0.8)	4.9 (0.5)	4.9 (0.5)	4.8 (0.8)	4.7 (0.8)	4.9 (0.6)	4.8 (0.6)	Not measured.		**A vs. DC: *p*** **≤** **0.0001** ^**e**^	**NI vs. Bog: *p* = 0.009**	+ve
DN1.2: Mother.	4.3 (1.3)	4.3 (1.2)	4.6 (1.0)	4.6 (1.0)	4.2 (1.4)	4.3 (1.3)	4.6 (0.9)	4.6 (0.9)			**A vs. DC: *p*** **≤** **0.0001** ^**e**^	**NI vs. Bog: *p*** **≤** **0.0001** ^**e**^	+ve
DN1.3: Father.	4.2 (1.4)	4.3 (1.3)	4.4 (1.2)	4.4 (1.2)	4.1 (1.4)	4.2 (1.4)	4.4 (1.1)	4.5 (1.1)			**A vs. DC: *p* = 0.0009**	**NI vs. Bog: *p* = 0.0001** ^**e**^	+ve
DN1.4: Brother(s).	4.7 (0.9)	4.6 (1.0)	4.8 (0.8)	4.7 (0.8)	4.7 (0.9)	4.7 (0.9)	4.7 (0.8)	4.7 (0.9)			**A vs. DC: *p* = 0.05**	NI vs. Bog: *p* = 0.93	+ve
DN1.5: Sister(s).	4.8 (0.7)	4.8 (0.8)	4.8 (0.7)	4.8 (0.7)	4.8 (0.7)	4.8 (0.8)	4.8 (0.7)	4.8 (0.7)			A vs. DC: *p* = 0.25	NI vs. Bog: *p* = 0.98	+ve
Self-report descriptive smoking norms scale 1 (average DN1.1 to DN1.5).	4.5 (0.7)	4.5 (0.7)	4.7 (0.5)	4.7 (0.5)	4.5 (0.7)	4.5 (0.7)	4.7 (0.5)	4.7 (0.5)			**A vs. DC: *p*** **≤** **0.0001** ^**e**^	**NI vs. Bog: *p*** **≤** **0.0001** ^**e**^	+ve
Survey: descriptive social norms 2 (α = 0.53; 1 = “almost all of them smoke” to 5 = “almost none of them smoke”/“do not know”)													
DN2.1: Friends.	4.7 (0.8)	4.6 (0.9)	4.8 (0.6)	4.8 (0.6)	4.7 (0.7)	4.6 (0.8)	4.7 (0.6)	4.7 (0.7)	Not measured		**A vs. DC: *p* = 0.0008**	NI vs. Bog: *p* = 0.07	+ve
DN2.2: Other family members.	4.2 (1.0)	4.2 (1.0)	4.4 (0.9)	4.4 (0.9)	4.1 (1.0)	4.1 (1.1)	4.4 (0.9)	4.5 (0.9)			**A vs. DC: *p* = 0.004**	**NI vs. Bog: *p*** **≤** **0.0001** ^**e**^	+ve
DN2.3: Classmates.	4.7 (0.6)	4.6 (0.7)	4.8 (0.5)	4.8 (0.6)	4.7 (0.7)	4.6 (0.7)	4.8 (0.5)	4.8 (0.6)			**A vs. DC: *p* = 0.0001** ^**e**^	**NI vs. Bog: *p*** **≤** **0.0001** ^**e**^	+ve
Self-report descriptive smoking norms scale 2 (average DN2.1 to DN2.3).	4.5 (0.6)	4.5 (0.7)	4.7 (0.5)	4.6 (0.5)	4.5 (0.6)	4.5 (0.7)	4.7 (0.5)	4.6 (0.5)			**A vs. DC: *p*** **≤** **0.0001** ^**e**^	**NI vs. Bog: *p*** **≤** **0.0001** ^**e**^	+ve
Survey: Smoking behavior, intentions, and susceptibility													
Self-report smoking behavior (1 = “sometimes smoke” to 4 = “never smoked”)^c^.	3.7 (0.7)	3.7 (0.7)	3.8 (0.5)	3.7 (0.6)	3.8 (0.6)	3.8 (0.7)	3.7 (0.7)	3.6 (0.7)	3.8 (0.5)	3.8 (0.6)	**NIC vs. A vs. DC: *F* = 5.80, df = 2, *p* = 0.003** **NIC vs. A: *p* = 0.02** NIC vs. DC: *p* = 0.67 **A vs. DC: *p* = 0.004**	**NIC vs. NI vs. Bog: *F* = 5.17 df = 2, *p* = 0.006** NIC vs. NI: *p* = 0.55 **NIC vs. Bog: *p* = 0.01** **NI vs. Bog: *p* = 0.007**	+ve
Intentions (1 = “I am a smoker” to 6 = “definitely remain a non-smoker”)^c^.	5.6 (1.0)	5.4 (1.3)	5.7 (0.9)	5.6 (1.0)	5.7 (0.8)	5.7 (0.9)	5.5 (1.1)	5.3 (1.3)	5.6 (0.8)	5.6 (0.8)	NIC vs. A vs. DC: *F* = 1.30, df = 2, *p* = 0.27	**NIC vs. NI vs. Bog: *F* = 5.21, df = 2, *p* = 0.006** NIC vs. NI: *p* = 0.21 NIC vs. Bog: *p* = 0.26 **NI vs. Bog: *p* = 0.002**	+ve
Susceptible to commencing smoking, *n*(%)^d^.	261 (37.2%)	297 (45.1%)	197 (34.4%)	217 (39.4%)	199 (31.6%)	199 (33.7%)	259 (40.2%)	315 (50.9%)	67 (30.5%)	69 (33.3%)	NIC vs. A vs. DC: χ^2^ = 2.71, df = 2, *p* = 0.26	**NIC vs. NI vs. Bog: χ** ^ **2** ^ **= 11.55, df = 2, *p* = 0.003** NIC vs. NI: *p* = 0.93 **NIC vs. Bog: *p* = 0.02** **NI vs. Bog: *p* = 0.002**	−ve
Survey: smoking knowledge and attitudes													
Knowledge (0 = “0 correct” to 6 = “6 correct”).	2.5 (1.5)	2.7 (1.6)	2.7 (1.5)	3.1 (1.4)	3.0 (1.5)	3.3 (1.5)	2.2 (1.4)	2.5 (1.5)	Not measured.		**A vs. DC: *p* = 0.03**	**NI vs. Bog: *p*** **≤** **0.0001** ^**e**^	+ve
Attitudes (1 = “least anti-smoking” to 5 = “most anti-smoking”; α = 0.81).	3.9 (0.6)	3.9 (0.7)	4.0 (0.6)	4.0 (0.6)	4.0 (0.6)	4.0 (0.6)	3.9 (0.7)	3.9 (0.7)			**A vs. DC: *p* = 0.02**	NI vs. Bog: *p* = 0.16	+ve
Survey: psycho-social variables and mediators													
Self-efficacy (Emotional; 1 = “least self-efficacy to resist smoking” to 6 = “greatest self-efficacy to resist smoking”; α = 0.97).	5.6 (0.9)	5.5 (1.0)	5.7 (0.7)	5.6 (0.8)	5.7 (0.8)	5.7 (0.9)	5.6 (0.8)	5.4 (0.9)	Not measured.		**A vs. DC: *p* = 0.01**	**NI vs. Bog: *p* = 0.01**	+ve
Self-efficacy (Friends; 1 = “least” to 6 = “greatest”; α = 0.96).	5.6 (0.9)	5.5 (0.9)	5.7 (0.6)	5.6 (0.8)	5.7 (0.8)	5.7 (0.8)	5.6 (0.7)	5.5 (0.9)			**A vs. DC: *p* = 0.01**	NI vs. Bog: *p* = 0.13	+ve
Self-efficacy (Opportunity; 1 = “least” to 6 = “greatest”; α = 0.98).	5.7 (0.7)	5.7 (0.8)	5.9 (0.4)	5.8 (0.6)	5.8 (0.6)	5.8 (0.6)	5.7 (0.6)	5.6 (0.8)			**A vs. DC: *p* = 0.002**	**NI vs. Bog: *p* = 0.008**	+ve
Perceived physical risks (0% = “lowest perceived risk” to 100% = “highest perceived risk”; α = 0.87).	60.1 (24.1)	62.1 (23.4)	61.9 (24.4)	67.1 (22.4)	62.5 (21.6)	66.0 (20.4)	59.4 (26.5)	62.9 (25.3)			A vs. DC: *p* = 0.19	**NI vs. Bog: *p* = 0.02**	+ve
Perceived social risks (0% = “lowest” to 100% = “highest”; α = 0.71).	67.5 (26.6)	67.4 (25.8)	68.9 (26.9)	72.5 (24.6)	75.1 (22.0)	75.9 (22.2)	61.5 (29.1)	63.8 (26.8)			A vs. DC: *p* = 0.33	**NI vs. Bog: *p*** **≤** **0.0001** ^**e**^	+ve
Perceived addiction risks (0% = “lowest” to 100% = “highest”; *α* = 0.49).	35.3 (25.2)	38.1 (25.6)	35.1 (27.1)	39.7 (26.6)	43.4 (24.9)	47.5 (24.0)	27.7 (24.9)	30.2 (25.1)			A vs. DC: *p* = 0.88	**NI vs. Bog: *p*** **≤** **0.0001** ^**e**^	+ve
Perceived benefits (0% = “lowest perceived benefit” to 100% = “highest perceived benefit”; α = 0.79).	24.4 (22.1)	23.6 (21.5)	22.7 (20.9)	24.1 (21.5)	23.4 (22.1)	24.0 (20.9)	23.8 (21.1)	23.7 (22.0)			A vs. DC: *p* = 0.16	NI vs. Bog: *p* = 0.75	−ve
Perceived behavioral control (easy to quit; 1 = “strongly disagree” to 5 = “strongly agree”).	3.0 (1.4)	3.0 (1.5)	3.0 (1.5)	2.9 (1.4)	2.5 (1.4)	2.4 (1.4)	3.5 (1.3)	3.5 (1.3)			A vs. DC: *p* = 0.57	**NI vs. Bog: *p*** **≤** **0.0001** ^**e**^	−ve
Perceived behavioral control (to avoid smoking; 1 = “strongly disagree” to 5 = “strongly agree”).	4.1 (1.2)	4.1 (1.2)	4.2 (1.2)	4.2 (1.1)	4.3 (1.1)	4.3 (1.0)	4.0 (1.3)	4.0 (1.3)			A vs. DC: *p* = 0.14	**NI vs. Bog: *p* = 0.0001** ^**e**^	+ve
Exposure to advertising in the media (0 = “saw smoking adverts in 0 locations in the media” to 8 = “saw smoking adverts in 8 locations in the media”).	2.7 (2.1)	2.8 (2.3)	2.4 (2.0)	3.9 (2.2)	2.4 (2.2)	2.8 (2.5)	2.8 (1.9)	3.7 (2.0)			**A vs. DC: *p* = 0.008**	**NI vs. Bog: *p* = 0.0006**	−ve
Exposure to advertising in shops (0 = “saw smoking adverts in 0 types of shop” to 4 = “saw smoking adverts in 4 types of shop”)^c^.	2.2 (1.2)	2.2 (1.3)	2.2 (1.2)	2.4 (1.2)	2.4 (1.3)	2.5 (1.3)	2.1 (1.1)	2.1 (1.2)	2.5 (1.1)	2.6 (1.2)	**NIC vs. A vs. DC: *F* = 5.17, df = 2, *p* = 0.006** **NIC vs. A: *p* = 0.005** **NIC vs. DC: *p* = 0.002** A vs. DC: *p* = 0.60	**NIC vs. NI vs. Bog: *F* = 16.50, df = 2, *p*** **≤** **0.0001** ^**e**^ NIC vs. NI: *p* = 0.23 **NIC vs. Bog: *p*** **≤** **0.0001** ^**e**^ **NI vs. Bog: *p*** **≤** **0.0001** ^**e**^	−ve
Objectively measured smoking behavior													
Carbon monoxide reading (expelled air; 0 to 30 parts per million)^c^.	2.7 (1.8)	2.7 (1.6)	2.3 (1.6)	2.8 (1.5)	1.5 (1.4)	2.0 (1.0)	3.4 (1.5)	3.5 (1.7)	2.3 (1.9)	1.9 (1.1)	**NIC vs. A vs. DC:** ***F* = 9.18, df = 2, *p* = 0.0001** ^**e**^ **NIC vs. A: *p* = 0.005** NIC vs. DC: *p* = 0.91 **A vs. DC: *p* = 0.0001** ^**e**^	**NIC vs. NI vs. Bog:** ***F* = 221.55, df = 2, *p*** **≤** **0.0001** ^**e**^ **NIC vs. NI: *p*** **≤** **0.0001** ^**e**^ **NIC vs. Bog: *p*** **≤** **0.0001** ^**e**^ **NI vs. Bog: *p*** **≤** **0.0001** ^**e**^	−ve

a Tests for baseline differences on pupil outcomes between three groups (intervention [0 = NI control; 1 = Dead Cool; 2 = ASSIST], or setting [0 = NI control; 1 = NI; 2 = bogotá]) were conducted using Kruskal-Wallis tests with adjustment for ties for ordinal variables, and analyses of variance (ANOVAs) for continuous variables. Bold and underlined text indicates tests which were significant at the *p* < 0.05 level. N/A = not applicable; NIC = NI control; A = ASSIST; DC = Dead Cool; NI = Northern Ireland; Bog = Bogotá; χ^2^ = chi-square statistic for Kruskal-Wallis tests; df = degrees of freedom; *F* = F-statistic for ANOVAs.

b Pairwise comparison tests were conducted using Wilcoxon rank-sum (Mann–Whitney) tests with adjustment for ties for ordinal variables, and independent samples t-tests (two-sided) for continuous variables. Bold and underlined text indicates tests which were significant at the *p* < 0.05 level. N/A = not applicable; NIC = NI control; A = ASSIST; DC = Dead Cool; NI = Northern Ireland; Bog = Bogotá.

c Indicates the direction of the outcome variable. “+ve” means that higher numerical values indicate greater anti-smoking values of the outcome. “−ve” means that lower numerical values indicate greater anti-smoking values of the outcome. Notably, we consider lower exposure to advertising in the media and shops as being more anti-smoking. We also consider higher perceived behavioral control to avoid smoking and lower perceived behavioral control that it would be easy to quit smoking if you smoked regularly as more anti-smoking. The rationale is that having higher perceived behavioral control to avoid smoking is presumed to be similar to having higher self-efficacy to resist smoking. Having higher perceived behavioral control that it would be easy to quit smoking if you smoked regularly is presumed to be similar to having lower perceived addiction risks.

d Smoking susceptibility is an ordinal outcome variable (also measured in the NI control group). Tests for baseline differences between three groups were conducted using Kruskal-Wallis tests with adjustment for ties. Pairwise comparisons were conducted using Wilcoxon rank-sum (Mann–Whitney) tests with adjustment for ties. Bold and underlined text indicates tests which were significant at the *p* < 0.05 level. All other outcomes were treated as continuous variables. See footnotes a,b.

e Retained statistical significance at the 5% level after using the Holm-Bonferroni procedure to correct the *p*-values for multiple testing (*p* < 0.05; based on all tests for baseline differences reported in Table 1, i.e., 124 tests).

### 2.5 Self-report survey and carbon monoxide measurements

In the MECHANISMS study, a survey was used to collect socio-demographic characteristics (gender, age, ethnicity, socio-economic status), social network data, smoking behavior, intentions, susceptibility, knowledge, attitudes, psychosocial constructs and mediators, personal characteristics, and wellbeing. In NI, socio-economic status (SES) was based on the Northern Ireland Multiple Deprivation Measure (NIMDM2017) (69). In Bogotá, SES was based on the Colombian government’s socio-economic level index (70).

All survey items were previously validated and adopted from studies involving similarly aged children (1). Self-report injunctive smoking norms were assessed with seven items enquiring about perceived approval of smoking from others (IN1 to IN7) (71). Self-report descriptive smoking norms were assessed with two scales: a five-item scale enquiring about how often specific individuals or groups engage in smoking (DN1.1 to DN1.5), and a three-item scale enquiring about the proportion of particular groups who are smokers (DN2.1 to DN2.3) (71).

Self-report smoking behavior was assessed with three items, with the main outcome variable rated on a four-point scale (*“I sometimes smoke”* to *“I have never smoked”*) (39, 72). Smoking intentions and susceptibility were measured with four items, and a binary variable indicating smoking susceptibility was derived from three of the items (39, 73, 74). The main smoking intentions outcome variable asked, *“If you DON’T currently smoke, do you intend to take up smoking in the next 6 months?,”* and was rated on a six-point scale (*“I am a smoker”* to *“Definitely remain a non-smoker”*). Knowledge of the effects of smoking was assessed with six questions, and the outcome variable was calculated as the number of questions answered correctly (0 to 6) (71). Attitudes towards smoking were measured with a 12-item scale (AT1 to AT12) (75).

Self-efficacy to avoid smoking was assessed across three domains: emotional (SEE1 to SEE9), friends (SEF1 to SEF9), and opportunity (SEO1 to SEO11) (76, 77). Separate subscales were used to measure perceived physical (RP1 to RP7), social (RS1 to RS3), and addiction risks (RA1 to RA3). Perceived benefits were measured with a five-item scale (BE1 to BE5) (78–80). PBC was measured with two single items capturing ease of quitting smoking and ease of avoiding smoking (81). Exposure to advertising in the media was assessed with eight items and summed as the number of media locations where participants reported seeing tobacco advertisements (0 to 8) (82). Exposure to advertising in shops was assessed with one item, representing the number of types of shop where tobacco advertisements were seen (0 to 4) (39).

Objective smoking behavior in the last 24 h was measured using hand-held carbon monoxide monitors (PICOAdvantage Smokerlyzer, Bedfont). Smokerlyzers are electrochemical sensors measuring exhaled carbon monoxide in parts per million (ppm; range 0-150 ppm, accuracy ±2 ppm or 5%) (83). A cut-off of >9 ppm indicated recent smoking behavior (8, 84). Objectively measured smoking behavior was analyzed as a continuous variable (ppm) (8).

Participants in the NI control group had their gender, age, ethnicity, and SES assessed similarly to MECHANISMS participants. The following smoking outcomes were also measured: self-report smoking behavior (39, 72), smoking intentions and susceptibility (39, 73, 74), exposure to advertising in shops (39), and objectively measured smoking behavior (8, 83, 84). All measures were identical to those used in MECHANISMS, except for the main smoking intentions outcome, which asked, *“Do you think you will smoke a cigarette at any time in the next year?,”* rated on a five-point scale (*“Definitely yes”* to *“Definitely not”*). This was treated as an approximation for the MECHANISMS six-month smoking intentions measure.

All survey items were coded such that higher numerical values indicated stronger anti-smoking norms, behaviors, intentions, knowledge, and attitudes, or higher values of the psychosocial constructs and mediators. Supplementary Table S3 provides a detailed breakdown of all measurement instruments. Table 1 shows the direction of the outcome variables (i.e., whether higher scores indicate more anti- or pro-smoking responses).

### 2.6 Statistical analysis

Analyses were conducted using Stata version 13 (StataCorp) (85) and R version 4.4.1 (86). The significance level was *p* < 0.05. Throughout the results and Supplementary Tables, we have indicated which results remained statistically significant after using the Holm-Bonferroni correction for multiple testing (87). Descriptive statistics for all outcomes at baseline and follow-up (means and standard deviations or frequencies and percentages) are reported in Table 1 and Supplementary Table S4.

Baseline differences between three groups (MECHANISMS interventions/settings and the NI control group) were assessed using Kruskal-Wallis tests (88) with adjustment for ties for ordinal variables, and analyses of variance (ANOVAs) for continuous variables. Pairwise comparisons by intervention or setting were conducted using Wilcoxon rank-sum (Mann–Whitney) tests (89, 90) with adjustment for ties for ordinal variables, and two-sided independent samples t-tests for continuous variables.

Histograms were generated to visualize distributions of all outcome variables at baseline and follow-up, as well as the distributions of change scores between baseline and follow-up by intervention group and setting (Supplementary Figures S4–S26). Cronbach’s alpha coefficients were calculated for individual scales and subscales (Table 1). Associations between all baseline outcomes were examined using Spearman’s rank-order correlations (Supplementary Tables S5, S6). To assess potential multicollinearity, variance inflation factors (VIFs) were calculated in Stata before model estimation. All VIFs were below the accepted threshold (VIF < 5) (91).

Covariate selection was determined using established criteria. Directed acyclic graphs (DAGs) were constructed using the systematic method of Ferguson et al. (92) and covariate selection was determined using VanderWeele’s “modified disjunctive cause” criterion. This involves adjusting for variables identified as causes of the exposure, outcome, or both, while excluding known instrumental variables (93).

The primary models compared all ASSIST participants with Dead Cool. Given that the intervention mechanisms may differ for ASSIST PS, who were trained to diffuse anti-smoking messages, we repeated the models using dummy variables to compare ASSIST non-PS and ASSIST PS separately with Dead Cool.

#### 2.6.1 Latent variable modelling and measurement invariance confirmatory factor analyses

Multiple item scales were modelled as latent variables in SEMs. Latent variables are not directly observed but are inferred through a mathematical model from a set of indicators that can be directly observed or measured (Supplementary Table S3) (94). For the latent variable representing smoking attitudes, item AT2 was excluded due to poor factor loading. We conducted measurement invariance CFA models to assess construct and factorial validity, as well as the equivalence of the latent variables across baseline and follow-up and across intervention groups and settings.

Measurement invariance of the latent variables between baseline and follow-up was examined using longitudinal CFA models, following the procedures described by Mackinnon et al. (95). CFAs were specified with the *‘lavaan’* package in R (96), using robust maximum likelihood estimation (MLR estimator), and full information maximum likelihood (FIML) to handle missing data (97, 98).

Longitudinal CFAs tested successive levels of measurement invariance:

- Configural invariance—testing whether each construct had the same pattern of fixed and free loadings across timepoints;
- Metric (weak) invariance—testing whether the factor loadings were equivalent across timepoints, meaning that the latent factor has the same interpretation;
- Scalar (strong) invariance—testing whether the item intercepts were equivalent across timepoints, permitting comparison of latent means; and
- Residual (strict) invariance—testing whether the item residual variances were equivalent across timepoints (99).

Model fit was evaluated using the chi-square statistic, Comparative Fit Index (CFI), Tucker-Lewis Index (TLI), Root Mean Square Error of Approximation (RMSEA), Standardized Root Mean Square Residual (SRMR), and three parsimony based fit indices—the Akaike Information Criterion (AIC), Bayesian Information Criterion (BIC), and adjusted BIC. Models were not rejected based on the chi-square statistic (100, 101). Good model fit was indicated by CFI ≥ 0.96, TLI ≥ 0.95, RMSEA ≤0.06, and SRMR ≤0.08 (100, 101). Acceptable fit was indicated by CFI ≥ 0.90, TLI ≥ 0.90, RMSEA ≤0.08, and SRMR ≤0.09 (95, 100).

Measurement invariance was assessed based on changes in model fit indices between nested models (99). Specifically, decreases in CFI and TLI ≤ 0.010, increases in RMSEA ≤0.015, and increases in SRMR ≤0.030 for metric invariance or ≤0.015 for scalar or residual invariance, provided evidence that measurement invariance was present (99, 102, 103). Complete or partial measurement invariance was considered sufficient (104). Subsequent SEMs were run with the complete or partial longitudinal measurement invariance constraints included (105, 106).

We also examined measurement invariance between settings (NI and Bogotá) and intervention groups (Dead Cool and ASSIST), examining configural, metric, and scalar invariance using the same criteria. Complete or partial scalar measurement invariance was required to compare latent means between groups (99). These multiple-group CFAs included latent variables at both baseline and follow-up along with the longitudinal invariance constraints. When some fit indices or changes in fit indices exceeded acceptable thresholds, we based our decision primarily on the CFI and on whether changes in most indices were acceptable (95, 102, 103).

Example *lavaan* syntax and additional statistical details are provided in the Supplementary Methods.

#### 2.6.2 Structural equation models examining differences in outcomes between intervention groups and settings at follow-up adjusted for baseline

Intervention group or setting effects on outcomes and mediators were estimated using SEMs with each latent or observed outcome (or mediator) variable as the dependent variable, and intervention group and setting as the key predictor variables. All models were adjusted for baseline values of the dependent variable, gender, age, ethnicity, and SES.

SEMs were specified with the *lavaan* package, using the MLR estimator and FIML to handle missing data (96). SEMs with binary dependent variables (smoking susceptibility) were estimated using the weighted least square mean and variance adjusted (WLSMV) estimator. Following Hayashi et al. (107), ordered categorical dependent variables with at least four or five categories were treated as continuous variables. Model fit was evaluated using the chi-square statistic, CFI, TLI, RMSEA, SRMR, and R-squared (R^2^; the proportion of variance in the dependent variable explained by the model) (108). Both unstandardized and standardized parameter estimates were extracted.

Post-hoc power analyses were conducted using the *“WebPower”* package in R (109). For models with observed outcome variables, power was estimated using the “linear regression” method (110, 111). For models with latent outcome variables, power was estimated using the “RMSEA-based” method (112). We also conducted general power calculations to estimate the minimum detectable effect sizes at 80% power for different sample sizes.

The models were repeated with two dummy variables comparing ASSIST non-PS and ASSIST PS separately with Dead Cool. To test whether intervention effects varied between NI and Bogotá, we ran additional models with intervention × setting interaction effects. Finally, models were run separately for NI and Bogotá to illustrate differences in intervention effect sizes by setting.

Since our experiment assessed injunctive and descriptive norms for both smoking and vaping, we repeated these models separately for smoking-related and vaping-related norms. The results of the disaggregated analyses were consistent with the main models that included smoking and vaping norms together.

Example syntax for the SEMs and power calculations is provided in the Supplementary Methods.

#### 2.6.3 Single and multiple mediator models

Mediation models were specified for MECHANISMS schools to compare the hypothesized causal pathways between the interventions and settings. The dependent variables included experimental donations to ASSIST/Dead Cool, self-report smoking behavior, objectively measured smoking behavior, smoking intentions, smoking susceptibility, knowledge of smoking, and attitudes towards smoking. These outcomes were selected because they represent the main outcomes from the behavioral economics experiments, and the primary outcomes targeted by the ASSIST and Dead Cool interventions (see “game theory and behavioral economics experiments” in the Supplementary Methods and the intervention logic models in Supplementary Figures S2, S3).

**Figure 1:**
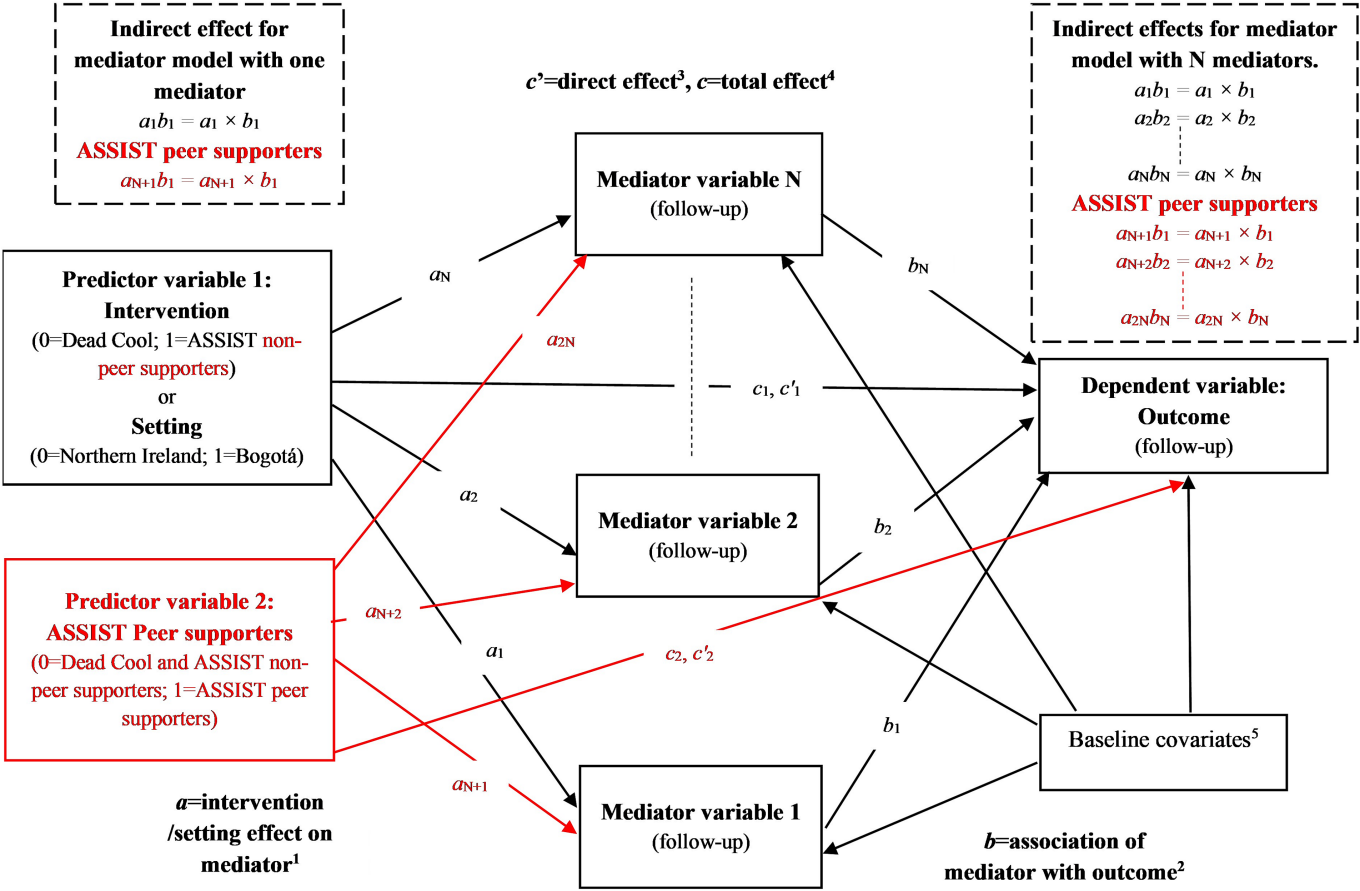
Structure of single and multiple mediator models with intervention group or setting as the predictor variable, including data from MECHANISMS schools. ^1^*a* = path coefficient(s) representing the magnitude of the effect(s) of the predictor variable(s) on the mediator variable(s), where the first predictor variable is a dummy variable indicating intervention (for ASSIST schools compared to Dead Cool schools) or setting (for Northern Ireland schools compared to Bogotá schools). For models comparing ASSIST non-peer supporters and ASSIST peer supporters separately with Dead Cool schools, a second predictor dummy variable is included. This is indicated by the shapes and text highlighted in red in the figure. *a*_1_ = predictor variable 1 effect on mediator 1, *a*_2_ = predictor variable 1 effect on mediator 2,…, *a*_N_ = predictor variable 1 effect on mediator N, *a*_N + 1_ = predictor variable 2 effect on mediator 1, *a*_N + 2_ = predictor variable 2 effect on mediator 2,…, *a*_2N_ = predictor variable 2 effect on mediator N. ^2^*b* = path coefficient(s) representing the magnitude of the association(s) of the mediator variable(s) (measured at follow-up) with the outcome variable (measured at follow-up). *b*_1_ = association of mediator 1 with outcome, *b*_2_ = association of mediator 2 with outcome,…, *b*_N_ = association of mediator N with outcome. ^3^*c’* = path coefficient(s) representing the magnitude of the effect(s) of the predictor variable(s) on the outcome variable adjusted for the mediator(s). Represents the direct effect(s). ^4^*c* = path coefficient(s) representing the magnitude of the effect(s) of the predictor variable(s) on the outcome variable unadjusted for the mediator(s). Represents the total effect(s). ^5^In addition to intervention (and ASSIST peer supporters) and setting, all models included the following baseline covariates: baseline values of the outcome, baseline values of the mediator(s), gender (0 = boy, 1 = girl/prefer not to say), age (1 = 12 years or less, 2 = 13 years, 3 = 14 years or more; entered into SEMs as two dummy variables), ethnicity (0 = no ethnic minority, 1 = ethnic minority), and socio-economic status (Northern Ireland: 1 = NIMDM2017 ≤ 296.6, 2 = 296.6 < NIMDM2017 ≤ 593.2, 3 = NIMDM2017 > 593.2; Bogotá: 1 = Informal settlement/Lowest/Low, 2 = Middle-Low/Middle, 3 = Middle-High/High; entered into SEMs as two dummy variables). See Supplementary Table S3 for details of variables.

In each mediation model, the predictor variable was either intervention group, two dummy variables comparing ASSIST non-PS and ASSIST PS separately with Dead Cool, or setting. The mediating variable was the mediator score at follow-up, and the dependent variable was the outcome score at follow-up. All paths were adjusted for baseline values of the mediator and outcome, gender, age, ethnicity, and SES (Figure 1).

Single mediator models were specified using the SEM product-of-coefficients approach (113). Results are reported for:

- *a*-path coefficients—intervention or setting effects on mediators,
- *b*-path coefficients—associations between mediators and outcomes,
- *c’*-path coefficients—direct effects of intervention or setting on outcomes, adjusted for the mediator, and
- indirect effects (*ab*)—mediated effects, defined as the product of the *a*- and *b*-path coefficients (114).

The total effects of intervention and setting (*c*-path coefficients, unadjusted for mediators) correspond to the SEMs described in the previous section.

The significance of the indirect effects was determined by 95% confidence intervals (CIs) computed using the maximum likelihood (ML) estimator and the bias-corrected bootstrap procedure with 10,000 iterations (114, 115). Mediation models with binary outcomes (smoking susceptibility) were estimated using the diagonally weighted least squares (DWLS) estimator and the bias-corrected bootstrap procedure with 10,000 iterations (114, 115). Model fit was evaluated using standard and robust versions of the chi-square statistic, CFI, TLI, RMSEA, SRMR, and R^2^ statistic.

Mediation was considered present when:

1. Intervention or setting significantly predicted the mediator (*a*-path),
2. The mediator was significantly associated with the outcome (*b*-path), and
3. The indirect effect was significant (*ab*) (42).

A *p*-value <0.05 indicated significance for the *a*-, *b*-, *c’*-, or *c*-path coefficients. A significant test of the indirect effect was established when the 95% bias-corrected bootstrap CI did not include 0 (116). Unstandardized and standardized parameter estimates were extracted.

Multiple mediator models were conducted examining parallel mediation using the same estimation procedures (Figure 1). Although our measured mediators were determined *a priori* based on theory-driven logic models (1), the number of candidate mediators was too large to include simultaneously in a single SEM-based multiple-mediator model, and many did not show significant indirect effects in the single mediator analyses. To avoid overfitting and reduce the risk of unstable parameter estimates associated with including a large set of correlated mediators, we used the data-driven “Coordinate-wise Mediation Filter” (CMF) method to identify a parsimonious subset of mediators to include in the multiple mediator models. The CMF method was implemented using the *“cmfilter”* package in R (117, 118). Briefly, CMF applies a systematic filtering procedure that retains only those mediators showing robust evidence of mediation, conditional on the set of currently selected mediators. CMF has demonstrated superior performance because it can identify important mediators that may be missed by alternative mediator subset selection approaches, such as unconditional filtering based on significant paths in single-mediator models (117). The product-of-coefficients test with *p* < 0.1 was used as the decision function, and 10,000 random-start iterations were run. Scree plots of selection rates were inspected to set cut-off points for selection (117). Mediators with significant indirect effects in the single mediator models—either the unstandardized or standardized solutions—were also included in the multiple mediator models. When more than four or five mediators were selected, they were divided into separate models. The theory of planned behavior variables (intentions, attitudes, norms, and PBC) were retained in the same model where possible^1^.

Post-hoc power to detect the indirect effects in the final single and multiple mediator models was calculated using Monte Carlo based power analysis for mediation models with the *‘power.boot’* function in the *‘bmem’* package in R (119, 120).

Figure 1 shows the structure of the single and multiple mediator models. Shapes and text highlighted in red denote the inclusion of the second dummy predictor variable for models comparing ASSIST non-PS and ASSIST PS separately with Dead Cool. Example syntax for the single and multiple mediation models, CMF function, and power analyses are provided in the Supplementary Methods.

#### 2.6.4 Sensitivity analyses comparing MECHANISMS participants with the Northern Ireland control group from the Dead Cool study

For outcomes and mediators measured in the NI control group—self-report smoking behavior, objectively measured smoking behavior, smoking intentions, smoking susceptibility, and exposure to advertising in shops—we conducted sensitivity analyses to compare MECHANISMS participants with the NI control group from the Dead Cool study.

Setting and intervention effects (equivalent to total effects or *c*-path coefficients) were estimated using SEMs with the outcome or mediator as the dependent variable and four dummy predictor variables representing each MECHANISMS setting/intervention group—NI Dead Cool, NI ASSIST, Bogotá Dead Cool, and Bogotá ASSIST—compared with the NI control group. All models were adjusted for baseline values of the dependent variable, gender, age, ethnicity, and SES. Statistical procedures and power calculations were conducted as described for the main SEMs above.

**Figure 2:**
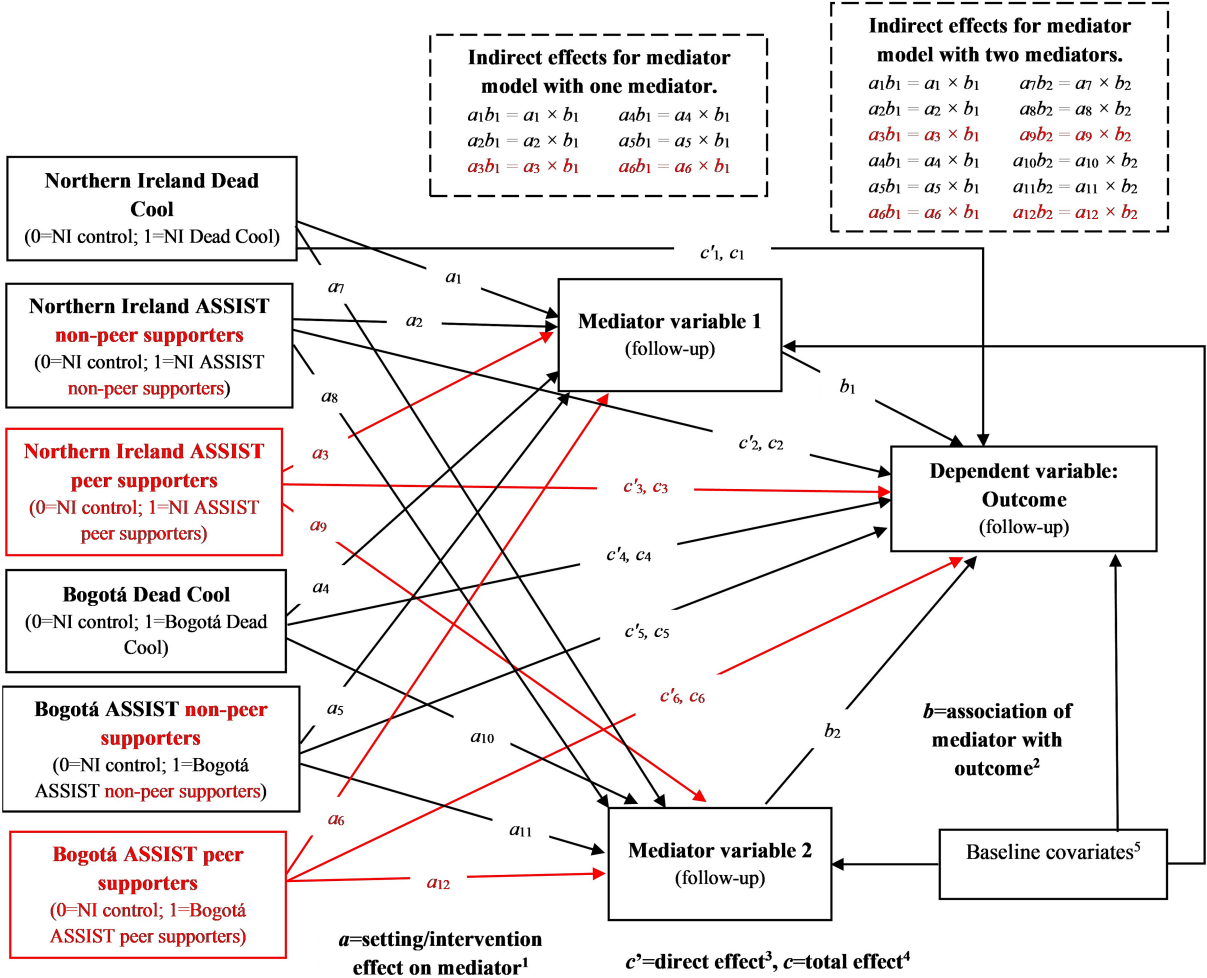
Structure of single and multiple mediator models for sensitivity analyses with predictor variables comparing MECHANISMS setting/intervention groups with the Northern Ireland control group. ^1^*a* = path coefficients representing the magnitude of the effects of the predictor variables on the mediator variable(s), where the predictor variables are dummy variables representing the MECHANISMS setting/intervention group compared to the Northern Ireland (NI) control group. For models comparing ASSIST non-peer supporters and ASSIST peer supporters separately with NI control schools, two further predictor dummy variables are included. This is indicated by the shapes and text highlighted in red in the figure. *a*_1_ = NI Dead Cool effect on mediator 1, *a*_2_ = NI ASSIST (non-peer supporters) effect on mediator 1, *a*_3_ = NI ASSIST peer supporters effect on mediator 1, *a*_4_ = Bogotá Dead Cool effect on mediator 1, *a*_5_ = Bogotá ASSIST (non-peer supporters) effect on mediator 1, *a*_6_ = Bogotá ASSIST peer supporters effect on mediator 1, *a*_7_ = NI Dead Cool effect on mediator 2, *a*_8_ = NI ASSIST (non-peer supporters) effect on mediator 2, *a*_9_ = NI ASSIST peer supporters effect on mediator 2, *a*_10_ = Bogotá Dead Cool effect on mediator 2, *a*_11_ = Bogotá ASSIST (non-peer supporters) effect on mediator 2, *a*_12_ = Bogotá ASSIST peer supporters effect on mediator 2. ^2^*b* = path coefficient(s) representing the magnitude of the association(s) of the mediator variable(s) (measured at follow-up) with the outcome variable (measured at follow-up). *b*_1_ = association of mediator 1 with outcome, *b*_2_ = association of mediator 2 with outcome. ^3^*c’* = path coefficients representing the magnitude of the effects of each MECHANISMS setting/intervention group compared to the NI control group on the outcome variable adjusted for the mediator(s). Represents the direct effects. *c’*_1_ = NI Dead Cool direct effect, *c’*_2_ = NI ASSIST (non-peer supporters) direct effect, *c’*_3_ = NI ASSIST peer supporters direct effect, *c’*_4_ = Bogotá Dead Cool direct effect, *c’*_5_ = Bogotá ASSIST (non-peer supporters) direct effect, *c’*_6_ = Bogotá ASSIST peer supporters direct effect. ^4^*c* = path coefficients representing the magnitude of the effects of each MECHANISMS setting/intervention group compared to the NI control group on the outcome variable unadjusted for the mediator(s). Represents the total effects. *c*_1_ = NI Dead Cool total effect, *c*_2_ = NI ASSIST (non-peer supporters) total effect, *c*_3_ = NI ASSIST peer supporters total effect, *c*_4_ = Bogotá Dead Cool total effect, *c*_5_ = Bogotá ASSIST (non-peer supporters) total effect, *c*_6_ = Bogotá ASSIST peer supporters total effect. ^5^In addition to setting/intervention group, all models included the following baseline covariates: baseline values of the outcome, baseline values of the mediator(s), gender (0 = boy, 1 = girl/prefer not to say), age (1 = 12 years or less, 2 = 13 years, 3 = 14 years or more; entered into SEMs as two dummy variables), ethnicity (0 = no ethnic minority, 1 = ethnic minority), and socio-economic status (Northern Ireland: 1 = NIMDM2017 ≤ 296.6, 2 = 296.6 < NIMDM2017 ≤ 593.2, 3 = NIMDM2017 > 593.2; Bogotá: 1 = Informal settlement/lowest/low, 2 = middle-low/middle, 3 = middle-high/high; entered into SEMs as two dummy variables). See Supplementary Table S3 for details of variables.

Mediation models were specified with the following outcomes as dependent variables: self-report smoking behavior, objectively measured smoking behavior, smoking intentions, and smoking susceptibility. Predictor variables were the same four dummy variables comparing each MECHANISMS setting/intervention group with the NI control group. All paths were adjusted for baseline values of the mediator and outcome, gender, age, ethnicity, and SES (Figure 2).

For models with self-report or objectively measured smoking behavior as the dependent variables, the potential mediators were smoking intentions and exposure to advertising in shops. For models with smoking intentions or smoking susceptibility as the dependent variables, exposure to advertising in shops was the only potential mediator. Multiple mediator models were conducted with self-report smoking behavior and objectively measured smoking behavior as the dependent variables. The CMF method was not applied because only two mediators were available for inclusion in models using NI control group data. The statistical procedures and power analyses for the mediation models were conducted as described for the main mediation models above.

The analyses were repeated to compare ASSIST non-PS and ASSIST PS separately with Dead Cool. Models included six dummy predictor variables representing each MECHANISMS setting/intervention group—NI Dead Cool, NI ASSIST non-PS, NI ASSIST PS, Bogotá Dead Cool, Bogotá ASSIST non-PS, and Bogotá ASSIST PS—compared to the NI control group.

The structure of single and multiple mediator models comparing MECHANISMS groups with the NI control group is shown in Figure 2. Shapes and text highlighted in red denote the addition of the two dummy predictor variables representing ASSIST non-PS and ASSIST PS compared separately with the NI control group. Example syntax is shown in the Supplementary Methods.

## 3 Results

There were significant baseline differences between settings for age, SES, ethnicity, and most smoking-related mediators and outcomes (Table 1; Supplementary Tables S1, S4). Although the study targeted the 12–13-year-old school year group in both settings, a higher proportion of pupils in Bogotá were older (26.6% aged ≥14 years versus 0.9% in NI). SES was lower overall in Bogotá, and a slightly higher percentage of pupils were classified as belonging to an ethnic minority group. Experimental injunctive and descriptive norms, and self-report descriptive norms, were more anti-smoking in Bogotá, and Bogotá pupils made higher donations to ASSIST/Dead Cool in the experimental tasks. In contrast, self-report injunctive norms, self-report and objectively measured smoking behavior, intentions, susceptibility, knowledge, self-efficacy, and perceived risks were more anti-smoking in NI. Whilst PBC that it would be easy to quit smoking was higher in Bogotá, PBC to avoid smoking was higher in NI. Exposure to tobacco advertising in the media was higher in Bogotá, while exposure to advertising in shops was higher in NI.

There were significant baseline differences between intervention groups for gender, SES, experimental injunctive and descriptive smoking/vaping norms, experimental donations to ASSIST/Dead Cool, self-report injunctive and descriptive smoking norms, self-report and objectively measured smoking behavior, knowledge, attitudes, self-efficacy, and exposure to advertising in shops and the media (Table 1; Supplementary Tables S1, S4). There was a slightly higher percentage of girls in Dead Cool schools. Although the proportion of pupils in the lowest SES category was similar across groups, a higher percentage of ASSIST pupils were in the highest SES category compared to Dead Cool. Most outcomes were more anti-smoking in Dead Cool schools at baseline.

The results of the longitudinal measurement invariance CFAs are shown in Supplementary Tables S7, S8. There was complete or partial strict (residual) measurement invariance for all latent variables apart from experimentally measured descriptive smoking/vaping norms which showed complete weak (metric) measurement invariance. CFI values ranged from 0.928 to 0.999, TLI ranged from 0.927 to 0.997, RMSEA ranged from 0.030 to 0.067, and SRMR ranged from 0.030 to 0.075.

Subsequent SEMs were estimated with the corresponding complete or partial measurement invariance constraints included. Due to the large increase in the BIC between the scalar and residual models for opportunity self-efficacy, subsequent SEMs included the partial strong (scalar) measurement invariance constraints for this construct.

The results of the multiple-group CFAs examining measurement invariance between settings and intervention groups are shown in Supplementary Tables S9–S12. There was supporting evidence for complete or partial strong (scalar) measurement invariance across settings and intervention groups for all latent variables. Therefore, we have evidence that our latent variables showed longitudinal invariance and measurement equivalence across intervention groups and settings.

**Figure 3:**
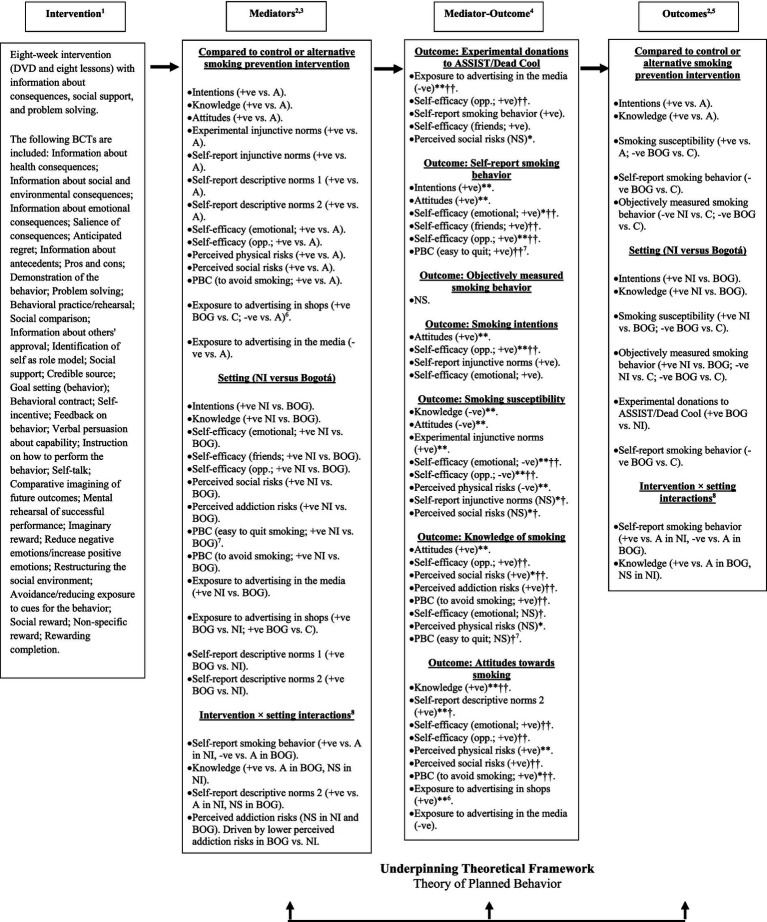
Adapted logic model for the Dead Cool intervention showing study results. ^1^Intervention column. Behavior change techniques (BCTs) are coded based on the Michie et al. (40) 93-item taxonomy. ^2^Mediators and outcomes columns. +ve vs. A: indicates changes in the mediator or outcome were more anti-smoking compared to ASSIST. –ve vs. A: indicates changes were more pro-smoking compared to ASSIST. +ve vs. C: indicates changes in the mediator or outcome were more anti-smoking compared to NI control. –ve vs. C: indicates changes were more pro-smoking compared to NI control. +ve NI vs. BOG: indicates changes in the mediator or outcome were more anti-smoking in NI compared to Bogotá. +ve BOG vs. NI: indicates changes were more anti-smoking in Bogotá compared to NI. +ve NI vs. C: indicates changes in the mediator or outcome were more anti-smoking in NI Dead Cool compared to NI control. −ve NI vs. C: indicates changes were more pro-smoking in NI Dead Cool compared to NI control. +ve BOG vs. C: indicates changes in the mediator or outcome were more anti-smoking in Bogotá Dead Cool compared to NI control. −ve BOG vs. C: indicates changes were more pro-smoking in Bogotá Dead Cool compared to NI control. ^3^Mediators column. Based on results for total effects of intervention or setting reported in Tables 3, 10, and intervention or setting effects on mediators (a-paths) reported in Tables 7, 9 and Supplementary Table S70. ^4^Mediator-outcome column. +ve: indicates the association between the mediator and outcome was positive. −ve: indicates the association was negative. NS: indicates no significant association. *Significant indirect effect for ASSIST vs. Dead Cool. **Significant mediator for ASSIST vs. Dead Cool. †Significant indirect effect for NI vs. Bogotá. ††Significant mediator for NI vs. Bogotá. Based on results of the mediator models reported in Tables 7–9. ^5^Outcomes column. Based on results for total effects of intervention or setting reported in Tables 3, 10. ^6^See footnote c of Table 2. ^7^See footnote d of Table 2. ^8^Intervention × setting interactions. Based on results of significant intervention × setting interactions and the corresponding separate estimation of intervention effects in NI and Bogotá reported in Table 5 and Supplementary Table S18. +ve vs. A in NI: indicates changes in the mediator or outcome were more anti-smoking for Dead Cool compared to ASSIST in NI. –ve vs. A in BOG: indicates changes were more pro-smoking for Dead Cool compared to ASSIST in Bogotá. +ve vs. A in BOG: indicates changes were more anti-smoking for Dead Cool compared to ASSIST in Bogotá. NS in NI: indicates intervention effect was non-significant in NI. NS in BOG: indicates intervention effect was non-significant in Bogotá.

**Figure 4:**
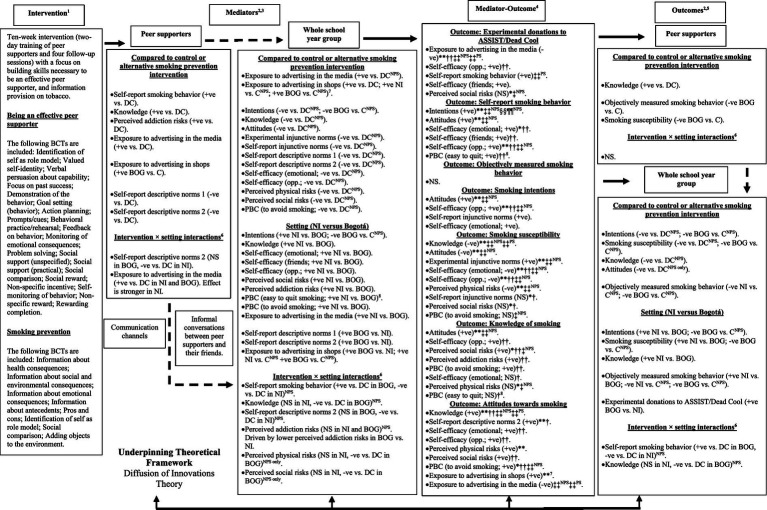
Adapted logic model for the ASSIST intervention showing study results. ^1^Intervention column. Behavior change techniques (BCTs) are coded based on the Michie et al. (40) 93-item taxonomy. ^2^Mediators and outcomes columns. +ve vs. DC: indicates changes in the mediator or outcome were more anti-smoking compared to Dead Cool. –ve vs. DC: indicates changes were more pro-smoking compared to Dead Cool. +ve vs. C: indicates changes in the mediator or outcome were more anti-smoking compared to NI control. –ve vs. C: indicates changes were more pro-smoking compared to NI control. +ve NI vs. BOG: indicates changes in the mediator or outcome were more anti-smoking in NI compared to Bogotá. +ve BOG vs. NI: indicates changes were more anti-smoking in Bogotá compared to NI. +ve NI vs. C: indicates changes in the mediator or outcome were more anti-smoking in NI ASSIST compared to NI control. –ve NI vs. C: indicates changes were more pro-smoking in NI ASSIST compared to NI control. +ve BOG vs. C: indicates changes in the mediator or outcome were more anti-smoking in Bogotá ASSIST compared to NI control. –ve BOG vs. C: indicates changes were more pro-smoking in Bogotá ASSIST compared to NI control. ^NPS^: indicates that the significant effects were also observed for the ASSIST non-peer supporter group. ^NPS^ only: indicates that the significant effects were observed for the ASSIST non-peer supporter group only. ^3^Mediators columns. Based on results for total effects of intervention or setting reported in Tables 3, 4, 10, 11, and intervention or setting effects on mediators (a-paths) reported in Tables 7–9 and Supplementary Tables S70, S74. ^4^Mediator-outcome column. +ve: indicates the association between the mediator and outcome was positive. −ve: indicates the association was negative. NS: indicates no significant association. *Significant indirect effect for ASSIST vs. Dead Cool. **Significant mediator for ASSIST vs. Dead Cool. ‡^NPS^Significant indirect effect for ASSIST non-peer supporters vs. Dead Cool. ‡‡^NPS^Significant mediator for ASSIST non-peer supporters vs. Dead Cool. ‡^PS^Significant indirect effect for ASSIST peer supporters vs. Dead Cool. ‡‡^PS^Significant mediator for ASSIST peer supporters vs. Dead Cool. †Significant indirect effect for NI vs. Bogotá. ††Significant mediator for NI vs. Bogotá. §§Significant mediator for Bogotá ASSIST vs. NI control. ¶¶^NPS^Significant mediator for Bogotá ASSIST non-peer supporters vs. NI control. ^5^Outcome columns. Based on results for total effects of intervention or setting reported in Tables 3, 4, 10, 11. ^6^Intervention × setting interactions. Based on results of significant intervention × setting interactions and the corresponding separate estimation of intervention effects in NI and Bogotá reported in Tables 5, 6 and Supplementary Tables S18, S21. +ve vs. DC in NI: indicates changes in the mediator or outcome were more anti-smoking for ASSIST compared to Dead Cool in NI. +ve vs. DC in BOG: indicates changes were more anti-smoking for ASSIST compared to Dead Cool in Bogotá. –ve vs. DC in NI: indicates changes were more pro-smoking for ASSIST compared to Dead Cool in NI. –ve vs. DC in BOG: indicates changes were more pro-smoking for ASSIST compared to Dead Cool in Bogotá. NS in NI: indicates intervention effect was non-significant in NI. NS in BOG: indicates intervention effect was non-significant in Bogotá. ^NPS^: indicates that the significant interactions and effects were also observed for the ASSIST non-peer supporter group. ^NPS^ only: indicates that the significant interactions and effects were observed for the ASSIST non-peer supporter group only. ^7^See footnote c of Table 2. ^8^See footnote d of Table 2.

An overview of the main results from the SEMs and final mediation models is presented in Table 2. The following paragraphs describe these results in detail, and readers are directed to consult the tables and Supplementary Files for additional numerical information. Adapted logic models for the ASSIST and Dead Cool interventions, depicting the study results described below, are also shown in Figures 3, 4. Throughout the results section, we describe significant results at the *p* < 0.05 level.

**Table 2:** Overview of results of the structural equation models and final mediation models.

Model^a^	Predictor(s)	Outcome variable(s)	Results^b^	Results tables
SEMs with intervention group and setting as predictors (MECHANISMS schools only).	Intervention (0 = DC, 1 = ASSIST). Setting (0 = NI, 1 = Bogotá). All paths adjusted for gender, age, ethnicity, SES, and baseline values of the outcome variable.	Separate models were run with the following outcomes: - Experiment donations to ASSIST/Dead Cool. - Self-report smoking behavior. - Objectively measured smoking behavior. - Intentions. - Smoking susceptibility. - Knowledge. - Attitudes. - Experiment injunctive smoking/vaping norms. - Experiment injunctive smoking norms. - Experiment injunctive vaping norms. - Experiment descriptive smoking/vaping norms. - Experiment descriptive smoking norms. - Experiment descriptive vaping norms. - Self-report injunctive smoking norms. - Self-report descriptive smoking norms 1. - Self-report descriptive smoking norms 2. - Self-efficacy (emotional). - Self-efficacy (friends). - Self-efficacy (opportunity). - Perceived physical risks. - Perceived social risks. - Perceived addiction risks. - Perceived benefits. - PBC (easy to quit). - PBC (to avoid smoking). - Exposure to advertising in the media. - Exposure to advertising in shops.	Intervention effects (*p* ≤ 0.05) Favors ASSIST: - Exposure to advertising in the media (↓). - Exposure to advertising in shops (↓). Favors DC: - Intentions (↑ anti-smoking). - Smoking susceptibility (↓). - Knowledge (↑). - Self-report descriptive smoking norms 2 (↑ anti-smoking). - PBC (easy to avoid) (↑). Setting effects (*p* ≤ 0.05) Favors Bogotá: - Experiment donations to ASSIST/Dead Cool (↑). - Self-report descriptive smoking norms 2 (↑ anti-smoking). - Exposure to advertising in shops (↓). Favors NI: - Objectively measured smoking behavior (↓). - Intentions (↑ anti-smoking). - Smoking susceptibility (↓). - Knowledge (↑). - Self-efficacy (emotional) (↑). - Self-efficacy (opportunity) (↑). - Perceived social risks (↑). - Perceived addiction risks (↑). - PBC (easy to quit) (↓). - PBC (to avoid smoking) (↑). - Exposure to advertising in the media (↓).	Table 2 and Supplementary Table S15
SEMs with intervention group as the predictor, comparing ASSIST non-PS and ASSIST PS separately with DC (MECHANISMS schools only).	Intervention (0 = DC and ASSIST PS, 1 = ASSIST non-PS). ASSIST PS (0 = DC and ASSIST non-PS, 1 = ASSIST PS). All paths adjusted for gender, age, ethnicity, SES, and baseline values of the outcome variable.			Intervention effects (*p* ≤ 0.05) Favours ASSIST PS vs. DC: - Knowledge (↑). - Perceived addiction risks (↑). - Exposure to advertising in the media (↓). Favours ASSIST non-PS vs. DC: - Exposure to advertising in the media (↓). Favors DC vs. ASSIST non-PS: - Intentions (↑ anti-smoking). - Smoking susceptibility (↓). - Knowledge (↑). - Attitudes (↑ anti-smoking). - Perceived physical risks (↑). - PBC (to avoid smoking) (↑).	Table 3 and Supplementary Table S16
SEMs with intervention × setting interactions (MECHANISMS schools only).	Intervention (0 = DC, 1 = ASSIST). Setting (0 = NI, 1 = Bogotá). Intervention × Setting interaction. All paths adjusted for gender, age, ethnicity, SES, and baseline values of the outcome variable.			Interaction effects (*p* ≤ 0.05) Self-report smoking behavior: - Favors DC in NI, favors ASSIST in Bogotá (↑ anti-smoking). Knowledge: - Favors DC in Bogotá (↑), NS in NI. Self-report descriptive smoking norms 2: - Favors DC in NI (↑ anti-smoking), NS in Bogotá. Perceived addiction risks: - NS in NI and Bogotá. Driven by lower perceived addiction risks in Bogotá vs. NI.	Table 4 and Supplementary Tables S17–S19
SEMs with intervention × setting interactions, comparing ASSIST non-PS and ASSIST PS separately with DC (MECHANISMS schools only).	Intervention (0 = DC and ASSIST PS, 1 = ASSIST non-PS). ASSIST PS (0 = DC and ASSIST non-PS, 1 = ASSIST PS). Setting (0 = NI, 1 = Bogotá). Intervention × Setting interaction. PS × Setting interaction. All paths adjusted for gender, age, ethnicity, SES, and baseline values of the outcome variable.			Interaction effects (*p* ≤ 0.05). Self-report smoking behavior: - Favors DC vs. ASSIST non-PS in NI, favors ASSIST non-PS in Bogotá (↑ anti-smoking). - Knowledge. - Favors DC vs. ASSIST non-PS in Bogotá (↑), NS in NI. Self-report descriptive smoking norms 2: - Favors DC vs. ASSIST non-PS in NI (↑ anti-smoking), NS in Bogotá. - Favors DC vs. ASSIST PS in NI (↑ anti-smoking), NS in Bogotá. Perceived physical risks: - Favors DC vs. ASSIST non-PS in Bogotá (↑), NS in NI. Perceived social risks: - Favors DC vs. ASSIST non-PS in Bogotá (↑), NS in NI. Perceived addiction risks: - NS in NI and Bogotá (DC vs. ASSIST non-PS). Driven by lower perceived addiction risks in Bogotá vs. NI. Exposure to advertising in the media: - Favors ASSIST PS vs. DC in NI and Bogotá (↓). Effect is stronger in NI.	Table 5 and Supplementary Tables S20–S22
Mediation models with intervention group as the predictor (MECHANISMS schools only).	Intervention (0 = DC, 1 = ASSIST). All paths adjusted for gender, age, ethnicity, SES, and baseline values of the outcome variable and mediator(s).	Experiment donations to ASSIST/Dead Cool.	Indirect effects (*p* ≤ 0.05), significant mediators* Perceived social risks: ↑ donations in DC vs. ASSIST due to ↑ perceived social risks. Exposure to advertising in the media*: ↑ donations in ASSIST vs. DC due to ↓ exposure to advertising in the media.	Table 6 and Supplementary Table S23
	
		Self-report smoking behavior.	Indirect effects (*p* ≤ 0.05), significant mediators* Intentions*: ↑ anti-smoking behavior in DC vs. ASSIST due to ↑ anti-smoking intentions. Attitudes*: ↑ anti-smoking behavior in DC vs. ASSIST due to ↑ anti-smoking attitudes. Self-efficacy (emotional): ↑ anti-smoking behavior in DC vs. ASSIST due to ↑ self-efficacy (emotional). Self-efficacy (opportunity)*: ↑ anti-smoking behavior in DC vs. ASSIST due to ↑ self-efficacy (opportunity).		
	
		Objectively measured smoking behavior.	No significant indirect effects or mediators.		
	
		Intentions.	Indirect effects (*p* ≤ 0.05), significant mediators* Attitudes*: ↑ anti-smoking intentions in DC vs. ASSIST due to ↑ anti-smoking attitudes. Self-efficacy (opportunity)*: ↑ anti-smoking intentions in DC vs. ASSIST due to ↑ self-efficacy (opportunity).		
	
Smoking susceptibility.	Indirect effects (*p* ≤ 0.05), significant mediators* Knowledge*: ↓ smoking susceptibility in DC vs. ASSIST due to ↑ knowledge. Attitudes*: ↓ smoking susceptibility in DC vs. ASSIST due to ↑ anti-smoking attitudes. Experiment injunctive smoking/vaping norms*: ↓ smoking susceptibility in DC vs. ASSIST due to ↑ anti-smoking injunctive smoking/vaping norms. Self-report injunctive smoking norms: ↓ smoking susceptibility in DC vs. ASSIST due to ↑ anti-smoking injunctive smoking norms. Self-efficacy (emotional)*: ↓ smoking susceptibility in DC vs. ASSIST due to ↑ self-efficacy (emotional). Self-efficacy (opportunity)*: ↓ smoking susceptibility in DC vs. ASSIST due to ↑ self-efficacy (opportunity). Perceived physical risks*: ↓ smoking susceptibility in DC vs. ASSIST due to ↑ perceived physical risks. Perceived social risks: ↓ smoking susceptibility in DC vs. ASSIST due to ↑ perceived social risks.	
	
Knowledge.	Indirect effects (*p* ≤ 0.05), significant mediators* Attitudes*: ↑ knowledge in DC vs. ASSIST due to ↑ anti-smoking attitudes. Perceived physical risks: ↑ knowledge in DC vs. ASSIST due to ↑ perceived physical risks. Perceived social risks: ↑ knowledge in DC vs. ASSIST due to ↑ perceived social risks.	
	
Attitudes.	Indirect effects (*p* ≤ 0.05), significant mediators* Knowledge*: ↑ anti-smoking attitudes in DC vs. ASSIST due to ↑ knowledge. Self-report descriptive smoking norms 2*: ↑ anti-smoking attitudes in DC vs. ASSIST due to ↑ anti-smoking descriptive smoking norms. Perceived physical risks*: ↑ anti-smoking attitudes in DC vs. ASSIST due to ↑ perceived physical risks. PBC (to avoid smoking): ↑ anti-smoking attitudes in DC vs. ASSIST due to ↑ PBC (to avoid smoking). Exposure to advertising in shops*^c^: ↑ anti-smoking attitudes in DC vs. ASSIST due to ↑ exposure to advertising in shops.	
Mediation models with intervention group as the predictor, comparing ASSIST non-PS and ASSIST PS separately with DC (MECHANISMS schools only).	Intervention (0 = DC and ASSIST PS, 1 = ASSIST non-PS). ASSIST PS (0 = DC and ASSIST non-PS, 1 = ASSIST PS). All paths adjusted for gender, age, ethnicity, SES, and baseline values of the outcome variable and mediator(s).	Experiment donations to ASSIST/Dead Cool.	Indirect effects (*p* ≤ 0.05), significant mediators* Self-report smoking behavior*: ↑ donations in ASSIST PS vs. DC due to ↑ anti-smoking behavior. Perceived social risks: ↑ donations in DC vs. ASSIST non-PS due to ↑ perceived social risks. Exposure to advertising in the media*: ↑ donations in ASSIST non-PS vs. DC due to ↓ exposure to advertising in the media. ↑ donations in ASSIST PS vs. DC due to ↓ exposure to advertising in the media.	Table 7 and Supplementary Table S38		
		Self-report smoking behavior.	Indirect effects (*p* ≤ 0.05), significant mediators* Intentions*: ↑ anti-smoking behavior in DC vs. ASSIST non-PS due to ↑ anti-smoking intentions. Attitudes*: ↑ anti-smoking behavior in DC vs. ASSIST non-PS due to ↑ anti-smoking attitudes. Self-efficacy (opportunity)*: ↑ anti-smoking behavior in DC vs. ASSIST non-PS due to ↑ self-efficacy (opportunity).				
		Objectively measured smoking behavior.	No significant indirect effects or mediators.				
		Intentions.	Indirect effects (*p* ≤ 0.05), significant mediators* Attitudes*: ↑ anti-smoking intentions in DC vs. ASSIST non-PS due to ↑ anti-smoking attitudes. Self-efficacy (opportunity)*: ↑ anti-smoking intentions in DC vs. ASSIST non-PS due to ↑ self-efficacy (opportunity).				
		Smoking susceptibility.	Indirect effects (*p* ≤ 0.05), significant mediators* Knowledge*: ↓ smoking susceptibility in DC vs. ASSIST non-PS due to ↑ knowledge. ↓ smoking susceptibility in ASSIST PS vs. DC due to ↑ knowledge. Attitudes*: ↓ smoking susceptibility in DC vs. ASSIST non-PS due to ↑ anti-smoking attitudes. Experiment injunctive smoking/vaping norms*: ↓ smoking susceptibility in DC vs. ASSIST non-PS due to ↑ anti-smoking injunctive smoking/vaping norms. Self-efficacy (emotional)*: ↓ smoking susceptibility in DC vs. ASSIST non-PS due to ↑ self-efficacy (emotional). Self-efficacy (opportunity)*: ↓ smoking susceptibility in DC vs. ASSIST non-PS due to ↑ self-efficacy (opportunity). Perceived physical risks*: ↓ smoking susceptibility in DC vs. ASSIST non-PS due to ↑ perceived physical risks. PBC (to avoid smoking): ↓ smoking susceptibility in DC vs. ASSIST non-PS due to ↑ PBC (to avoid smoking).				
		Knowledge.	Indirect effects (*p* ≤ 0.05), significant mediators* Attitudes*: ↑ knowledge in DC vs. ASSIST non-PS due to ↑ anti-smoking attitudes. Perceived physical risks: ↑ knowledge in DC vs. ASSIST non-PS due to ↑ perceived physical risks. Perceived social risks: ↑ knowledge in DC vs. ASSIST non-PS due to ↑ perceived social risks.				
		Attitudes.	Indirect effects (*p* ≤ 0.05), significant mediators* Knowledge*: ↑ anti-smoking attitudes in DC vs. ASSIST non-PS due to ↑ knowledge. ↑ anti-smoking attitudes in ASSIST PS vs. DC due to ↑ knowledge. PBC (to avoid smoking)*: ↑ anti-smoking attitudes in DC vs. ASSIST non-PS due to ↑ PBC (to avoid smoking). Exposure to advertising in the media*: ↑ anti-smoking attitudes in ASSIST non-PS vs. DC due to ↓ exposure to advertising in the media. ↑ anti-smoking attitudes in ASSIST PS vs. DC due to ↓ exposure to advertising in the media.		
Mediation models with setting as the predictor (MECHANISMS schools only).	Setting (0 = NI, 1 = Bogotá). All paths adjusted for gender, age, ethnicity, SES, and baseline values of the outcome variable and mediator(s).	Experiment donations to ASSIST/Dead Cool.	Indirect effects (*p* ≤ 0.05), significant mediators* Self-efficacy (opportunity)*: ↑ donations in NI vs. Bogotá due to ↑ self-efficacy (opportunity). Exposure to advertising in the media*: ↑ donations in NI vs. Bogotá due to ↓ exposure to advertising in the media.	Table 8 and Supplementary Table S53		
		Self-report smoking behavior.	Indirect effects (*p* ≤ 0.05), significant mediators* Self-efficacy (emotional)*: ↑ anti-smoking behavior in NI vs. Bogotá due to ↑ self-efficacy (emotional). Self-efficacy (friends)*: ↑ anti-smoking behavior in NI vs. Bogotá due to ↑ self-efficacy (friends). Self-efficacy (opportunity)*: ↑ anti-smoking behavior in NI vs. Bogotá due to ↑ self-efficacy (opportunity). PBC (easy to quit)*^d^: ↑ anti-smoking behavior in Bogotá vs. NI due to ↑ PBC (easy to quit).				
		Objectively measured smoking behavior.	No significant indirect effects or mediators.				
		Intentions.	Indirect effects (*p* ≤ 0.05), significant mediators* Self-efficacy (opportunity)*: ↑ anti-smoking intentions in NI vs. Bogotá due to ↑ self-efficacy (opportunity).				
		Smoking susceptibility.	Indirect effects (*p* ≤ 0.05), significant mediators* Self-efficacy (emotional)*: ↓ smoking susceptibility in NI vs. Bogotá due to ↑ self-efficacy (emotional). Self-efficacy (opportunity)*: ↓ smoking susceptibility in NI vs. Bogotá due to ↑ self-efficacy (opportunity). Self-report injunctive smoking norms: ↓ smoking susceptibility in NI vs. Bogotá due to ↑ anti-smoking injunctive smoking norms. Perceived social risks: ↓ smoking susceptibility in NI vs. Bogotá due to ↑ perceived social risks.				
		Knowledge.	Indirect effects (*p* ≤ 0.05), significant mediators* Self-efficacy (emotional): ↑ knowledge in NI vs. Bogotá due to ↑ self-efficacy (emotional). Self-efficacy (opportunity)*: ↑ knowledge in NI vs. Bogotá due to ↑ self-efficacy (opportunity). Perceived social risks*: ↑ knowledge in NI vs. Bogotá due to ↑ perceived social risks. Perceived addiction risks*: ↑ knowledge in NI vs. Bogotá due to ↑ perceived addiction risks. PBC (easy to quit): ↑ knowledge in NI vs. Bogotá due to ↓ PBC (easy to quit). PBC (to avoid smoking)*: ↑ knowledge in NI vs. Bogotá due to ↑ PBC (to avoid smoking).				
		Attitudes.	Indirect effects (*p* ≤ 0.05), significant mediators* Knowledge*: ↑ anti-smoking attitudes in NI vs. Bogotá due to ↑ knowledge. Self-report descriptive smoking norms 2: ↑ anti-smoking attitudes in Bogotá vs. NI due to ↑ anti-smoking descriptive smoking norms. Self-efficacy (emotional)*: ↑ anti-smoking attitudes in NI vs. Bogotá due to ↑ self-efficacy (emotional). Self-efficacy (opportunity)*: ↑ anti-smoking attitudes in NI vs. Bogotá due to ↑ self-efficacy (opportunity). Perceived social risks*: ↑ anti-smoking attitudes in NI vs. Bogotá due to ↑ perceived social risks. PBC (to avoid smoking)*: ↑ anti-smoking attitudes in NI vs. Bogotá due to ↑ PBC (to avoid smoking).		
Sensitivity analyses: SEMs with predictor variables comparing MECHANISMS schools with the NI control group (MECHANISMS and NI control group schools).	Four dummy variables indicating NI DC, NI ASSIST, Bogotá DC, and Bogotá ASSIST schools (NI control group = 0). All paths adjusted for gender, age, ethnicity, SES, and baseline values of the outcome variable.	Separate models were run with the following outcomes: - Self-report smoking behavior. - Objectively measured smoking behavior. - Intentions. - Smoking susceptibility. - Exposure to advertising in shops.	Self-report smoking behavior: - Favors NI control vs. Bogotá DC (↑ anti-smoking). Objectively measured smoking behavior: - Favors NI control vs. NI DC (↓). - Favors NI control vs. NI ASSIST (↓). - Favors NI control vs. Bogotá DC (↓). - Favors NI control vs. Bogotá ASSIST (↓). Intentions: - Favors NI control vs. Bogotá ASSIST (↑ anti-smoking). Smoking susceptibility: - Favors NI control vs. Bogotá DC (↓). - Favors NI control vs. Bogotá ASSIST (↓). Exposure to advertising in shops: - Favors Bogotá DC vs. NI control (↓). - Favors Bogotá ASSIST vs. NI control (↓).	Table 9 and Supplementary Table S68
Sensitivity analyses: SEMs with predictor variables comparing MECHANISMS schools with the NI control group, with ASSIST non-PS and ASSIST PS compared separately (MECHANISMS and NI control group schools).	Six dummy variables indicating NI DC, NI ASSIST non-PS, NI ASSIST PS, Bogotá DC, Bogotá ASSIST non-PS, and Bogotá ASSIST PS (NI control group = 0). All paths adjusted for gender, age, ethnicity, SES, and baseline values of the outcome variable.			Self-report smoking behavior: - Favors NI control vs. Bogotá DC (↑ anti-smoking). Objectively measured smoking behavior: - Favors NI control vs. NI DC (↓). - Favors NI control vs. NI ASSIST non-PS (↓). - Favors NI control vs. Bogotá DC (↓). - Favors NI control vs. Bogotá ASSIST non-PS (↓). - Favors NI control vs. Bogotá ASSIST PS (↓). Intentions: - Favors NI control vs. Bogotá ASSIST non-PS (↑ anti-smoking). Smoking susceptibility: - Favors NI control vs. Bogotá DC (↓). - Favors NI control vs. Bogotá ASSIST non-PS (↓). - Favors NI control vs. Bogotá ASSIST PS (↓). Exposure to advertising in shops: - Favors Bogotá DC vs. NI control (↓). - Favors Bogotá ASSIST non-PS vs. NI control (↓). - Favors Bogotá ASSIST PS vs. NI control (↓).	Table 10 and Supplementary Table S69
Sensitivity analyses: Mediation models with predictor variables comparing MECHANISMS schools with the NI control group (MECHANISMS and NI control group schools).	Four dummy variables indicating NI DC, NI ASSIST, Bogotá DC, and Bogotá ASSIST schools (NI control group = 0). All paths adjusted for gender, age, ethnicity, SES, and baseline values of the outcome variable and mediator(s).	Self-report smoking behavior.	Indirect effects (*p* ≤ 0.05), significant mediators* Intentions*: ↑ anti-smoking behavior in NI control vs. Bogotá ASSIST due to ↑ anti-smoking intentions.	Supplementary Tables S70, S71
		Objectively measured smoking behavior.	No significant indirect effects or mediators.	
		Intentions.	No significant indirect effects or mediators.	
		Smoking susceptibility.	No significant indirect effects or mediators.	
Sensitivity analyses: Mediation models with predictor variables comparing MECHANISMS schools with the NI control group, with ASSIST non-PS and ASSIST PS compared separately (MECHANISMS and NI control group schools).	Six dummy variables indicating NI DC, NI ASSIST non-PS, NI ASSIST PS, Bogotá DC, Bogotá ASSIST non-PS, and Bogotá ASSIST PS (NI control group = 0). All paths adjusted for gender, age, ethnicity, SES, and baseline values of the outcome variable and mediator(s).	Self-report smoking behavior.	Indirect effects (*p* ≤ 0.05), significant mediators* Intentions*: ↑ anti-smoking behavior in NI control vs. Bogotá ASSIST non-PS due to ↑ anti-smoking intentions.	Supplementary Tables S74, S75
		Objectively measured smoking behavior.	No significant indirect effects or mediators.	
		Intentions.	No significant indirect effects or mediators.	
		Smoking susceptibility.	No significant indirect effects or mediators.	

a DC = Dead Cool; N/A = not applicable; NI = Northern Ireland; NS = non-significant; PBC = perceived behavioral control; PS = peer supporters; SEM = structural equation model; SES = socio-economic status.

b The arrows in brackets indicate the direction of a “favorable” outcome. E.g., “Exposure to advertising in the media (↓)” indicates lower exposure to advertising in the media is considered a more favorable outcome. “Intentions (↑ anti-smoking)” indicates that higher anti-smoking intentions is considered a more favourable outcome. For the mediation models summary, the significant indirect effects (*p* < 0.05) are listed with significant mediators indicated with asterisks (*). The criteria to determine significant mediation included: a significant *a*-path, a significant *b*-path, and a significant indirect effect. Arrows are used to describe the direction of the significant indirect effects.

c Exposure to advertising in shops was higher in Dead Cool schools compared to ASSIST at follow-up controlling for baseline values. We considered this to be a negative (more pro-smoking) outcome for Dead Cool compared to ASSIST. However, higher exposure to advertising in shops was positively associated with having more negative attitudes towards smoking and was a significant mediator which increased anti-smoking attitudes in Dead Cool schools compared to ASSIST. One possible explanation could be that pupils considered negative or regulatory advertisements for smoking in shops when responding (e.g., health warnings on cigarette packaging).

d Perceived behavioral control (PBC) that it would be easy to quit smoking if you smoked regularly was lower in NI compared to Bogotá at follow-up controlling for baseline values. We considered this to be a positive (more anti-smoking) outcome for NI compared to Bogotá because having lower PBC that it would be easy to quit smoking if you smoked regularly is presumed to be similar to having higher perceived addiction risks. However, higher PBC that it would be easy to quit smoking if you smoked regularly was positively associated with having higher self-report anti-smoking behavior, and was a significant mediator which increased self-report anti-smoking behavior in Bogotá schools compared to NI. Conversely, higher PBC that it would be easy to quit smoking was negatively associated with knowledge about smoking, with a significant indirect effect which increased knowledge in NI schools compared to Bogotá (in the opposite direction to its mediating effect on self-report smoking behavior). One possible explanation could be that pupils who are self-report “never smokers” and have never tried smoking may have lower perceptions of how the addictive nature of smoking might impact their ability to quit if they were regular smokers. Pupils with higher knowledge of the effects of smoking will have higher perceptions of how the addictive nature of smoking might impact their ability to quit if they were regular smokers.

### 3.1 Structural equation models examining differences in outcomes between intervention groups and settings at follow-up adjusted for baseline

The results of the general power calculations estimating the minimum detectable effect sizes at different sample sizes with 80% power are reported in Supplementary Tables S13, S14. For models with observed outcomes, a sample size of 1,150 provided 80% power to detect an effect size of 0.0084 at *p* < 0.05, where the effect size was based on the change in the R^2^ when including intervention group and setting as predictors in the model. For models with latent outcomes, a sample size of 1,190 and 395 degrees of freedom had 80% power to detect an effect size of 0.004 at *p* < 0.05, where the effect size was based on the change in the RMSEA when including intervention group and setting as predictors.

The results of SEMs comparing intervention groups and settings for MECHANISMS participants are reported in Table 3 (unstandardized coefficients) and Supplementary Table S15 (standardized coefficients). At follow-up controlling for baseline, ASSIST schools showed lower exposure to advertising in the media and shops compared to Dead Cool. Conversely, Dead Cool schools showed greater anti-smoking intentions, lower smoking susceptibility, greater knowledge about smoking, greater PBC that it would be easy to avoid smoking, and more anti-smoking values of self-report descriptive norms (scale 2).

**Table 3:** Results of structural equation models with intervention group (ASSIST versus Dead Cool) and setting (Bogotá versus Northern Ireland) as predictor variables, including data from MECHANISMS schools.

Outcome^a^	*n*	Intervention (ASSIST vs. DC)^b^			Setting (Bogotá vs. NI)^c^			Model fit statistics^a^^,^^d^							Power^g^
		b	SE	*p*-value	b	SE	*p*-value	χ^2^, *p*-value^e^	df	CFI^e^	TLI^e^	RMSEA (90% CI)^e^	SRMR	R^2,^^f^	
Observed outcome variables (O)															
Donation to ASSIST/Dead Cool (0 to 10)	1,141	0.26	0.15	0.0884	**0.48**	**0.17**	**0.0056**	0.00, *p* = 1.00	0	1.000	1.000	0.000 (0.000, 0.000)	0.000	0.110	0.885
Self-report smoking behavior (1 to 4)	1,190	0.03	0.03	0.3787	−0.01	0.03	0.6877	0.00, *p* = 1.00	0	1.000	1.000	0.000 (0.000, 0.000)	0.000	0.475	0.094
Objectively measured smoking behavior (0 to 30)	1,133	−0.07	0.09	0.4330	**1.18**	**0.11**	**<0.0001** ^**i**^	0.00, *p* = 1.00	0	1.000	1.000	0.000 (0.000, 0.000)	0.000	0.247	1.000
Intentions (1 to 6)	1,187	**−0.22**	**0.07**	**0.0017** ^**i**^	**−0.15**	**0.07**	**0.0378**	0.00, *p* = 1.00	0	1.000	1.000	0.000 (0.000, 0.000)	0.000	0.120	0.969
Smoking susceptibility (0 = not susceptible; 1 = susceptible)^h^	1,087	**0.22**	**0.09**	**0.0122**	**0.38**	**0.10**	**0.0001** ^**i**^	0.00, *p* = 1.00	0	1.000	1.000	0.000 (0.000, 0.000)	0.000	0.355	1.000
Knowledge (0 to 6)	1,188	**−0.39**	**0.08**	**<0.0001** ^**i**^	**−0.38**	**0.09**	**<0.0001** ^**i**^	0.00, *p* = 1.00	0	1.000	1.000	0.000 (0.000, 0.000)	0.000	0.277	1.000
Experiment descriptive smoking norms (P3Q1; −1 to +1)	1,142	0.03	0.03	0.3253	0.05	0.03	0.1814	0.00, *p* = 1.00	0	1.000	1.000	0.000 (0.000, 0.000)	0.000	0.180	0.367
Experiment descriptive vaping norms (P3Q2; −1 to +1)	1,141	−0.01	0.03	0.6475	−0.01	0.04	0.6868	0.00, *p* = 1.00	0	1.000	1.000	0.000 (0.000, 0.000)	0.000	0.234	0.070
Perceived behavioral control (easy to quit; 1 to 5)	1,182	0.15	0.08	0.0796	**0.72**	**0.10**	**<0.0001** ^**i**^	0.00, *p* = 1.00	0	1.000	1.000	0.000 (0.000, 0.000)	0.000	0.212	1.000
Perceived behavioral control (to avoid smoking; 1 to 5)	1,188	**−0.14**	**0.07**	**0.0495**	**−0.30**	**0.08**	**0.0001** ^**i**^	0.00, *p* = 1.00	0	1.000	1.000	0.000 (0.000, 0.000)	0.000	0.065	0.984
Exposure to advertising in the media (0 to 8)	1,184	**−1.19**	**0.12**	**<0.0001** ^**i**^	**0.56**	**0.14**	**0.0001** ^**i**^	0.00, *p* = 1.00	0	1.000	1.000	0.000 (0.000, 0.000)	0.000	0.263	1.000
Exposure to advertising in shops (0 to 4)	1,177	**−0.14**	**0.07**	**0.0420**	**−0.34**	**0.08**	**<0.0001** ^**i**^	0.00, *p* = 1.00	0	1.000	1.000	0.000 (0.000, 0.000)	0.000	0.191	0.997
Latent outcome variables (L)															
Attitudes (AT1 to AT12, excluding AT2)	1,190	−0.12	0.06	0.0575	0.05	0.07	0.4764	(1) 1327.00, *p* < 0.0001	395	(1) 0.883	(1) 0.880	(1) 0.045 (0.042, 0.047)	0.050	0.186	(1) 0.635
								(2) 1163.03, *p* < 0.0001		(2) 0.890	(2) 0.886	(2) 0.044 (0.041, 0.047)			(2) 0.683
Experiment injunctive smoking/vaping norms (P2S2 to P2S9)	1,190	0.10	0.06	0.1082	−0.01	0.06	0.9246	(1) 777.16, *p* < 0.0001	236	(1) 0.901	(1) 0.896	(1) 0.044 (0.040, 0.047)	0.047	0.272	(1) 0.743
								(2) 619.68, *p* < 0.0001		(2) 0.912	(2) 0.907	(2) 0.042 (0.038, 0.047)			(2) 0.839
Experiment injunctive smoking norms (P2S2 to P2S6 and P2S9)	1,190	0.07	0.07	0.2886	0.02	0.07	0.7349	(1) 418.04, *p* < 0.0001	147	(1) 0.903	(1) 0.893	(1) 0.039 (0.035, 0.044)	0.039	0.170	(1) 0.916
								(2) 332.13, *p* < 0.0001		(2) 0.917	(2) 0.909	(2) 0.037 (0.032, 0.043)			(2) 0.972
Experiment injunctive vaping norms (P2S7 to P2S8)	1,190	0.10	0.06	0.0816	−0.07	0.06	0.2748	(1) 76.93, *p* < 0.0001	29	(1) 0.964	(1) 0.952	(1) 0.037 (0.027, 0.047)	0.028	0.090	(1) 0.483
								(2) 74.67, *p* < 0.0001		(2) 0.965	(2) 0.954	(2) 0.038 (0.027, 0.048)			(2) 0.529
Experiment descriptive smoking/vaping norms (P3Q1 to P3Q2)	1,190	0.03	0.07	0.6703	0.05	0.07	0.4614	(1) 94.38, *p* < 0.0001	26	(1) 0.969	(1) 0.955	(1) 0.047 (0.037, 0.057)	0.038	0.258	(1) 0.417
								(2) 88.56, *p* < 0.0001		(2) 0.970	(2) 0.956	(2) 0.048 (0.037, 0.059)			(2) 0.454
Self-report injunctive smoking norms (IN1 to IN7)	1,190	−0.05	0.07	0.4910	−0.01	0.08	0.9225	(1) 612.65, *p* < 0.0001	191	(1) 0.920	(1) 0.915	(1) 0.043 (0.039, 0.047)	0.050	0.315	(1) 0.071
								(2) 490.34, *p* < 0.0001		(2) 0.930	(2) 0.926	(2) 0.041 (0.037, 0.046)			(2) 0.098
Self-report descriptive smoking norms 1 (DN1.1 to DN1.5)	1,190	−0.03	0.08	0.7188	0.12	0.08	0.1411	(1) 463.02, *p* < 0.0001	116	(1) 0.932	(1) 0.927	(1) 0.050 (0.045, 0.055)	0.063	0.451	(1) 0.082
								(2) 317.80, *p* < 0.0001		(2) 0.946	(2) 0.941	(2) 0.047 (0.041, 0.053)			(2) 0.128
Self-report descriptive smoking norms 2 (DN2.1 to DN2.3)	1,190	**−0.16**	**0.08**	**0.0383**	**0.23**	**0.09**	**0.0121**	(1) 194.57, *p* < 0.0001	54	(1) 0.919	(1) 0.905	(1) 0.047 (0.040, 0.054)	0.045	0.364	(1) 0.322
								(2) 148.26, *p* < 0.0001		(2) 0.932	(2) 0.921	(2) 0.045 (0.036, 0.053)			(2) 0.395
Self-efficacy (emotional; SEE1 to SEE9)	1,190	−0.05	0.06	0.3487	**−0.17**	**0.06**	**0.0062**	(1) 1082.91, *p* < 0.0001	282	(1) 0.967	(1) 0.965	(1) 0.049 (0.046, 0.052)	0.048	0.228	(1) 0.004
								(2) 468.54, *p* < 0.0001		(2) 0.982	(2) 0.981	(2) 0.037 (0.031, 0.043)			(2) 0.033
Self-efficacy (friends; SEF1 to SEF9)	1,190	−0.01	0.06	0.8080	−0.12	0.06	0.0513	(1) 1228.09, *p* < 0.0001	284	(1) 0.959	(1) 0.957	(1) 0.053 (0.050, 0.056)	0.056	0.233	(1) 0.003
								(2) 601.20, *p* < 0.0001		(2) 0.971	(2) 0.970	(2) 0.045 (0.040, 0.050)			(2) 0.013
Self-efficacy (opportunity; SEO1 to SEO11)	1,190	−0.08	0.06	0.1816	**−0.14**	**0.07**	**0.0434**	(1) 2245.55, *p* < 0.0001	373	(1) 0.945	(1) 0.940	(1) 0.065 (0.062, 0.068)	0.048	0.143	(1) 0.000
								(2) 678.76, *p* < 0.0001		(2) 0.971	(2) 0.968	(2) 0.048 (0.041, 0.054)			(2) 0.001
Perceived physical risks (RP1 to RP7)	1,190	−0.11	0.06	0.0636	−0.01	0.06	0.8393	(1) 1149.14, *p* < 0.0001	190	(1) 0.885	(1) 0.877	(1) 0.065 (0.062, 0.069)	0.075	0.219	(1) 0.987
								(2) 1050.98, *p* < 0.0001		(2) 0.887	(2) 0.879	(2) 0.066 (0.062, 0.070)			(2) 0.987
Perceived social risks (RS1 to RS3)	1,190	−0.11	0.07	0.0972	**−0.17**	**0.07**	**0.0163**	(1) 240.30, *p* < 0.0001	53	(1) 0.897	(1) 0.877	(1) 0.054 (0.048, 0.062)	0.064	0.191	(1) 0.587
								(2) 227.46, *p* < 0.0001		(2) 0.900	(2) 0.881	(2) 0.055 (0.048, 0.062)			(2) 0.596
Perceived addiction risks (RA1 to RA3)	1,190	−0.01	0.06	0.8353	**−0.46**	**0.08**	**<0.0001** ^**i**^	(1) 436.54, *p* < 0.0001	53	(1) 0.742	(1) 0.693	(1) 0.078 (0.071, 0.085)	0.066	0.175	(1) 1.000
								(2) 440.70, *p* < 0.0001		(2) 0.749	(2) 0.701	(2) 0.080 (0.073, 0.087)			(2) 1.000
Perceived benefits (BE1 to BE5)	1,190	−0.04	0.06	0.5145	−0.02	0.06	0.7926	(1) 377.99, *p* < 0.0001	115	(1) 0.949	(1) 0.944	(1) 0.044 (0.039, 0.049)	0.045	0.142	(1) 0.505
								(2) 320.01, *p* < 0.0001		(2) 0.954	(2) 0.950	(2) 0.043 (0.037, 0.049)			(2) 0.585

a DC = Dead Cool; NI = Northern Ireland; b = unstandardized regression coefficient; SE = standard error; χ^2^ = Chi-square Goodness of Fit statistic; df = degrees of freedom (χ^2^ test); CFI = Comparative Fit Index; TLI = Tucker Lewis Index; RMSEA = Root Mean Square Error of Approximation; CI = confidence interval; SRMR = Standardized Root Mean Square Residual; R^2^ = R-squared; (O) = observed variable; (L) = latent variable.

b Regression coefficients represent the average change in the observed/latent outcome variable for ASSIST schools compared to Dead Cool schools, adjusted for baseline values of the outcome variable. In addition to baseline values of the outcome, all models also include the following baseline variables as covariates: setting (0 = Northern Ireland, 1 = Bogotá), gender (0 = boy, 1 = girl/prefer not to say), age (1 = 12 years or less, 2 = 13 years, 3 = 14 years or more; entered into SEMs as two dummy variables), ethnicity (0 = No ethnic minority, 1 = ethnic minority), and socio-economic status (NI: 1 = NIMDM2017 ≤ 296.6, 2 = 296.6 < NIMDM2017 ≤ 593.2, 3 = NIMDM2017 > 593.2; Bogotá: 1 = Informal settlement/Lowest/Low, 2 = Middle-Low/Middle, 3 = Middle-High/High; entered into SEMs as two dummy variables). See Supplementary Table S3 for details of variables. Bold and underlined text indicates tests which were significant at the *p* < 0.05 level. Structural equation models were specified using maximum likelihood estimation with robust (Huber-White) standard errors (MLR estimator) and imputation of missing data by full information maximum likelihood (FIML). Structural equation models with smoking susceptibility as the outcome variable were specified using the weighted least square mean and variance adjusted (WLSMV) estimator. Models were run including the longitudinal measurement invariance constraints on the factor loadings, intercepts, and residual variances for latent variables (as outlined in Supplementary Tables S7, S8). Unstandardized parameter values are reported in Table 3 (std.lv, which standardizes all latent variables in the model only). Standardized parameter values are reported in Supplementary Table S15 (std.all, which standardizes all latent and observed variables in the model).

c Regression coefficients represent the average change in the observed/latent outcome variable for Bogotá schools compared to Northern Ireland schools, adjusted for baseline values of the outcome variable, intervention (0 = Dead Cool, 1 = ASSIST), gender, age, ethnicity, and socio-economic status. Bold and underlined text indicates tests which were significant at the *p* < 0.05 level.

d Model fit statistics were estimated using the robust (Huber-White) maximum likelihood (MLR) estimator. For models with smoking susceptibility as the outcome variable, model fit statistics were estimated using the weighted least square mean and variance adjusted (WLSMV) estimator.

e (1) standard test statistic; (2) robust test statistic. For models with observed outcome variables (O), the results were the same for the standard and robust tests.

f R-squared: the percentage of variation in the outcome variable explained by the model.

g Power to detect the observed “Intervention” and “Setting” effects with a significance level of *p* < 0.05, calculated using WebPower. For models with observed outcome variables (O), the “linear regression” method was used. For models with latent outcome variables (L), the “power analysis based on the RMSEA” method was used: (1) power calculated based on the standard RMSEA; (2) power calculated based on the robust RMSEA.

h Structural equation models with smoking susceptibility as the outcome variable were specified using the weighted least square mean and variance adjusted (WLSMV) estimator.

i Retained statistical significance at the 5% level after using the Holm-Bonferroni procedure to correct the *p*-values for multiple testing (*p* < 0.05; based on the number of models in Table 3, i.e., 27 tests).

Bogotá schools compared to NI made higher experimental donations to ASSIST/Dead Cool, and reported lower exposure to advertising in shops and more anti-smoking values of self-report descriptive norms (scale 2). On the other hand, NI schools had lower levels of objectively measured smoking behavior, greater anti-smoking intentions, lower smoking susceptibility, greater knowledge, lower PBC that it would be easy to quit smoking, greater PBC to avoid smoking, lower exposure to advertising in the media, higher self-efficacy to resist smoking (emotional and opportunity subscales), and higher perceived social and addiction risks. R^2^ ranged between 0.065 and 0.475 and power ranged between 0.001 and 1.000.

The results of SEMs comparing ASSIST non-PS and ASSIST PS separately with Dead Cool are reported in Table 4 (unstandardized coefficients) and Supplementary Table S16 (standardized coefficients). At follow-up controlling for baseline, the ASSIST non-PS showed lower exposure to advertising in the media compared to Dead Cool schools. Meanwhile, the ASSIST PS showed greater knowledge, lower exposure to advertising in the media, and higher perceived addiction risks compared to Dead Cool.

**Table 4:** Results of structural equation models with predictor variables comparing ASSIST non-peer supporters and ASSIST peer supporters separately with Dead Cool, including data from MECHANISMS schools.

Outcome^a^	*n*	Intervention (ASSIST non-PS vs. DC)^b^			Peer supporters (ASSIST PS vs. DC)^c^			Setting (Bogotá vs. NI)^d^			Model fit statistics^a^^,^^e^							Power^h^
		b	SE	*p*-value	b	SE	*p*-value	b	SE	*p*-value	χ^2^, *p*-value^f^	df	CFI^f^	TLI^f^	RMSEA (90% CI)^f^	SRMR	R^2,^^g^	
Observed outcome variables (O)																		
Donation to ASSIST/Dead Cool	1,141	0.26	0.16	0.1075	0.26	0.24	0.2816	**0.48**	**0.17**	**0.0057**	0.00, *p* = 1.00	0	1.000	1.000	0.000 (0.000, 0.000)	0.000	0.110	0.842
Self-report smoking behavior	1,190	0.01	0.03	0.7137	0.10	0.05	0.0651	−0.01	0.03	0.6998	0.00, *p* = 1.00	0	1.000	1.000	0.000 (0.000, 0.000)	0.000	0.477	0.365
Objectively measured smoking behavior	1,133	−0.09	0.10	0.3393	0.01	0.15	0.9290	**1.18**	**0.11**	**<0.0001** ^**j**^	0.00, *p* = 1.00	0	1.000	1.000	0.000 (0.000, 0.000)	0.000	0.247	1.000
Intentions	1,187	**−0.24**	**0.08**	**0.0017** ^**j**^	−0.16	0.12	0.1620	**−0.15**	**0.07**	**0.0381**	0.00, *p* = 1.00	0	1.000	1.000	0.000 (0.000, 0.000)	0.000	0.120	0.958
Smoking susceptibility^i^	1,087	**0.21**	**0.09**	**0.0211**	0.25	0.15	0.0973	**0.38**	**0.10**	**0.0001** ^**j**^	0.00, *p* = 1.00	0	1.000	1.000	0.000 (0.000, 0.000)	0.000	0.355	1.000
Knowledge	1,188	**−0.57**	**0.09**	**<0.0001** ^**j**^	**0.31**	**0.14**	**0.0262**	**−0.38**	**0.09**	**<0.0001** ^**j**^	0.00, *p* = 1.00	0	1.000	1.000	0.000 (0.000, 0.000)	0.000	0.306	1.000
Experiment descriptive smoking norms	1,142	0.03	0.03	0.3199	0.02	0.05	0.6430	0.04	0.03	0.1834	0.00, *p* = 1.00	0	1.000	1.000	0.000 (0.000, 0.000)	0.000	0.180	0.320
Experiment descriptive vaping norms	1,141	−0.01	0.03	0.7234	−0.02	0.05	0.6353	−0.01	0.04	0.6797	0.00, *p* = 1.00	0	1.000	1.000	0.000 (0.000, 0.000)	0.000	0.234	0.073
Perceived behavioral control (easy to quit)	1,182	0.15	0.09	0.0933	0.14	0.13	0.2965	**0.72**	**0.10**	**<0.0001** ^**j**^	0.00, *p* = 1.00	0	1.000	1.000	0.000 (0.000, 0.000)	0.000	0.212	1.000
Perceived behavioral control (to avoid smoking)	1,188	**−0.19**	**0.08**	**0.0136**	0.06	0.11	0.6058	**−0.30**	**0.08**	**0.0001** ^**j**^	0.00, *p* = 1.00	0	1.000	1.000	0.000 (0.000, 0.000)	0.000	0.069	0.992
Exposure to advertising in the media	1,184	**−1.24**	**0.13**	**<0.0001** ^**j**^	**−0.99**	**0.20**	**<0.0001** ^**j**^	**0.56**	**0.14**	**0.0001** ^**j**^	0.00, *p* = 1.00	0	1.000	1.000	0.000 (0.000, 0.000)	0.000	0.264	1.000
Exposure to advertising in shops	1,177	−0.13	0.07	0.0847	−0.20	0.11	0.0880	**−0.34**	**0.08**	**<0.0001** ^**j**^	0.00, *p* = 1.00	0	1.000	1.000	0.000 (0.000, 0.000)	0.000	0.191	0.994
Latent outcome variables (L)																		
Attitudes	1,190	**−0.14**	**0.07**	**0.0426**	−0.05	0.11	0.6729	0.05	0.07	0.4702	(1) 1343.36, *p* < 0.0001	416	(1) 0.884	(1) 0.880	(1) 0.043 (0.041, 0.046)	0.049	0.187	(1) 0.346
											(2) 1184.04, *p* < 0.0001		(2) 0.890	(2) 0.887	(2) 0.043 (0.040, 0.046)			(2) 0.398
Experiment injunctive smoking/vaping norms	1,190	0.10	0.06	0.1296	0.09	0.10	0.3619	−0.01	0.06	0.9229	(1) 790.67, *p* < 0.0001	251	(1) 0.901	(1) 0.896	(1) 0.043 (0.039, 0.046)	0.045	0.272	(1) 0.515
											(2) 638.28, *p* < 0.0001		(2) 0.912	(2) 0.907	(2) 0.041 (0.037, 0.045)			(2) 0.666
Experiment injunctive smoking norms	1,190	0.08	0.07	0.2614	0.03	0.10	0.7860	0.02	0.07	0.7482	(1) 431.03, *p* < 0.0001	158	(1) 0.902	(1) 0.892	(1) 0.038 (0.034, 0.042)	0.038	0.170	(1) 0.844
											(2) 347.35, *p* < 0.0001		(2) 0.917	(2) 0.908	(2) 0.036 (0.031, 0.041)			(2) 0.947
Experiment injunctive vaping norms	1,190	0.09	0.06	0.1248	0.12	0.09	0.2206	−0.06	0.06	0.2799	(1) 78.46, *p* < 0.0001	32	(1) 0.965	(1) 0.953	(1) 0.035 (0.025, 0.045)	0.025	0.091	(1) 0.358
											(2) 75.93, *p* < 0.0001		(2) 0.966	(2) 0.956	(2) 0.035 (0.025, 0.046)			(2) 0.399
Experiment descriptive smoking/vaping norms	1,190	0.03	0.07	0.6449	0.01	0.10	0.9046	0.05	0.07	0.4644	(1) 94.58, *p* < 0.0001	29	(1) 0.970	(1) 0.957	(1) 0.044 (0.034, 0.054)	0.034	0.258	(1) 0.239
											(2) 88.62, *p* < 0.0001		(2) 0.971	(2) 0.958	(2) 0.044 (0.034, 0.055)			(2) 0.262
Self-report injunctive smoking norms	1,190	−0.06	0.08	0.3922	0.02	0.14	0.8887	−0.01	0.08	0.9259	(1) 630.48, *p* < 0.0001	204	(1) 0.919	(1) 0.914	(1) 0.042 (0.038, 0.046)	0.048	0.315	(1) 0.024
											(2) 512.26, *p* < 0.0001		(2) 0.929	(2) 0.925	(2) 0.040 (0.036, 0.045)			(2) 0.040
Self-report descriptive smoking norms 1	1,190	−0.02	0.08	0.8430	−0.07	0.12	0.5302	0.12	0.08	0.1419	(1) 466.74, *p* < 0.0001	125	(1) 0.933	(1) 0.928	(1) 0.048 (0.043, 0.053)	0.059	0.451	(1) 0.015
											(2) 324.08, *p* < 0.0001		(2) 0.947	(2) 0.943	(2) 0.045 (0.038, 0.051)			(2) 0.027
Self-report descriptive smoking norms 2	1,190	−0.15	0.08	0.0704	−0.21	0.14	0.1228	**0.23**	**0.09**	**0.0124**	(1) 198.30, *p* < 0.0001	59	(1) 0.919	(1) 0.906	(1) 0.045 (0.038, 0.051)	0.042	0.365	(1) 0.172
											(2) 152.09, *p* < 0.0001		(2) 0.933	(2) 0.922	(2) 0.042 (0.034, 0.051)			(2) 0.221
Self-efficacy (emotional)	1,190	−0.07	0.06	0.2865	−0.0002	0.09	0.9981	**−0.17**	**0.06**	**0.0063**	(1) 1101.52, *p* < 0.0001	299	(1) 0.967	(1) 0.965	(1) 0.047 (0.045, 0.051)	0.046	0.229	(1) 0.000
											(2) 492.08, *p* < 0.0001		(2) 0.982	(2) 0.981	(2) 0.036 (0.030, 0.041)			(2) 0.009
Self-efficacy (friends)	1,190	−0.03	0.06	0.6686	0.04	0.10	0.6897	−0.12	0.06	0.0518	(1) 1243.05, *p* < 0.0001	301	(1) 0.959	(1) 0.957	(1) 0.051 (0.048, 0.054)	0.054	0.233	(1) 0.000
											(2) 624.53, *p* < 0.0001		(2) 0.972	(2) 0.970	(2) 0.044 (0.039, 0.049)			(2) 0.001
Self-efficacy (opportunity)	1,190	−0.08	0.06	0.1814	−0.05	0.11	0.6139	**−0.14**	**0.07**	**0.0433**	(1) 2261.29, *p* < 0.0001	394	(1) 0.945	(1) 0.940	(1) 0.063 (0.061, 0.066)	0.047	0.143	(1) 0.000
											(2) 711.21, *p* < 0.0001		(2) 0.971	(2) 0.968	(2) 0.046 (0.040, 0.052)			(2) 0.000
Perceived physical risks	1,190	**−0.18**	**0.06**	**0.0043**	0.19	0.10	0.0719	−0.01	0.06	0.8714	(1) 1176.42, *p* < 0.0001	203	(1) 0.884	(1) 0.876	(1) 0.063 (0.060, 0.067)	0.073	0.222	(1) 0.945
											(2) 1082.29, *p* < 0.0001		(2) 0.885	(2) 0.877	(2) 0.065 (0.061, 0.068)			(2) 0.946
Perceived social risks	1,190	−0.13	0.07	0.0561	0.001	0.12	0.9903	**−0.17**	**0.07**	**0.0162**	(1) 247.20, *p* < 0.0001	58	(1) 0.896	(1) 0.876	(1) 0.052 (0.046, 0.059)	0.061	0.191	(1) 0.413
											(2) 234.80, *p* < 0.0001		(2) 0.899	(2) 0.880	(2) 0.053 (0.046, 0.060)			(2) 0.419
Perceived addiction risks	1,190	−0.09	0.06	0.1622	**0.31**	**0.11**	**0.0068**	**−0.46**	**0.08**	**<0.0001** ^**j**^	(1) 455.11, *p* < 0.0001	58	(1) 0.737	(1) 0.687	(1) 0.076 (0.069, 0.082)	0.063	0.188	(1) 1.000
											(2) 456.12, *p* < 0.0001		(2) 0.745	(2) 0.697	(2) 0.078 (0.071, 0.084)			(2) 1.000
Perceived benefits	1,190	−0.07	0.06	0.2658	0.08	0.11	0.4591	−0.02	0.06	0.8118	(1) 394.10, *p* < 0.0001	124	(1) 0.947	(1) 0.943	(1) 0.043 (0.038, 0.048)	0.044	0.146	(1) 0.386
											(2) 337.53, *p* < 0.0001		(2) 0.952	(2) 0.948	(2) 0.042 (0.037, 0.048)			(2) 0.480

a PS = peer supporters; DC = Dead Cool; NI = Northern Ireland; b = unstandardized regression coefficient; SE = standard error; χ^2^ = Chi-square Goodness of Fit statistic; df = degrees of freedom (χ^2^ test); CFI = Comparative Fit Index; TLI = Tucker Lewis Index; RMSEA = Root Mean Square Error of Approximation; CI = confidence interval; SRMR = Standardized Root Mean Square Residual; R^2^ = R-squared; (O) = observed variable; (L) = latent variable.

b Regression coefficients represent the average change in the observed/latent outcome variable for ASSIST non-peer supporters compared to Dead Cool schools, adjusted for baseline values of the outcome variable. In addition to baseline values of the outcome, all models also include the following baseline variables as covariates: ASSIST peer supporters (0 = Dead Cool and ASSIST non-peer supporters, 1 = ASSIST peer supporters), setting (0 = Northern Ireland, 1 = Bogotá), gender (0 = boy, 1 = girl/prefer not to say), age (1 = 12 years or less, 2 = 13 years, 3 = 14 years or more; entered into SEMs as two dummy variables), ethnicity (0 = No ethnic minority, 1 = ethnic minority), and socio-economic status (NI: 1 = NIMDM2017 ≤ 296.6, 2 = 296.6 < NIMDM2017 ≤ 593.2, 3 = NIMDM2017 > 593.2; Bogotá: 1 = Informal settlement/Lowest/Low, 2 = Middle-Low/Middle, 3 = Middle-High/High; entered into SEMs as two dummy variables). See Supplementary Table S3 for details of variables. Bold and underlined text indicates tests which were significant at the *p* < 0.05 level. Structural equation models were specified using maximum likelihood estimation with robust (Huber-White) standard errors (MLR estimator) and imputation of missing data by full information maximum likelihood (FIML). Structural equation models with smoking susceptibility as the outcome variable were specified using the weighted least square mean and variance adjusted (WLSMV) estimator. Models were run including the longitudinal measurement invariance constraints on the factor loadings, intercepts, and residual variances for latent variables (as outlined in Supplementary Tables S7, S8). Unstandardized parameter values are reported in Table 4 (std.lv, which standardizes all latent variables in the model only). Standardized parameter values are reported in Supplementary Table S16 (std.all, which standardizes all latent and observed variables in the model).

c Regression coefficients represent the average change in the observed/latent outcome variable for ASSIST peer supporters compared to Dead Cool schools, adjusted for baseline values of the outcome variable, intervention (0 = Dead Cool and ASSIST peer supporters, 1 = ASSIST non-peer supporters), setting, gender, age, ethnicity, and socio-economic status. Bold and underlined text indicates tests which were significant at the *p* < 0.05 level.

d Regression coefficients represent the average change in the observed/latent outcome variable for Bogotá schools compared to Northern Ireland schools, adjusted for baseline values of the outcome variable, intervention (0 = Dead Cool and ASSIST peer supporters, 1 = ASSIST non-peer supporters), ASSIST peer supporters (0 = Dead Cool and ASSIST non-peer supporters, 1 = ASSIST peer supporters), gender, age, ethnicity, and socio-economic status. Bold and underlined text indicates tests which were significant at the *p* < 0.05 level.

e Model fit statistics were estimated using the robust (Huber-White) maximum likelihood (MLR) estimator. For models with smoking susceptibility as the outcome variable, model fit statistics were estimated using the weighted least square mean and variance adjusted (WLSMV) estimator.

f (1) standard test statistic; (2) robust test statistic. For models with observed outcome variables (O), the results were the same for the standard and robust tests.

g R-squared: the percentage of variation in the outcome variable explained by the model.

h Power to detect the observed “Intervention” and “Setting” effects with a significance level of *p* < 0.05, calculated using WebPower. For models with observed outcome variables (O), the “linear regression” method was used. For models with latent outcome variables (L), the “power analysis based on the RMSEA” method was used: (1) power calculated based on the standard RMSEA; (2) power calculated based on the robust RMSEA.

i Structural equation models with smoking susceptibility as the outcome variable were specified using the weighted least square mean and variance adjusted (WLSMV) estimator.

j Retained statistical significance at the 5% level after using the Holm-Bonferroni procedure to correct the *p*-values for multiple testing (*p* < 0.05; based on the number of models in Table 4, i.e., 27 tests).

Conversely, Dead Cool schools compared to the ASSIST non-PS showed greater anti-smoking intentions, lower smoking susceptibility, greater knowledge, higher PBC to avoid smoking, more anti-smoking attitudes, and higher perceived physical risks. There were no statistically significant effects indicating more favorable outcomes for Dead Cool schools compared to the ASSIST PS. R^2^ ranged between 0.069 and 0.477 and power ranged between 0.001 and 1.000.

Notably, the sign of the coefficient for knowledge was in the opposite direction for the ASSIST non-PS (who showed decreases in knowledge compared to Dead Cool) and ASSIST PS (who showed increases in knowledge compared to Dead Cool). The more pro-smoking outcomes observed for ASSIST compared to Dead Cool, for intentions, smoking susceptibility, PBC to avoid smoking, and attitudes, appeared to be concentrated in the ASSIST non-PS group. However, it is important to note the reduced power to detect effects in the ASSIST PS group due to their smaller number (*n* = 603 ASSIST non-PS, *n* = 142 ASSIST PS, *n* = 599 Dead Cool).

#### 3.1.1 Structural equation models with interactions examining differences in intervention effects between settings

The results of SEMs with intervention × setting interactions are reported in Table 5 and Supplementary Table S17. The results of SEMs examining the intervention effects separately for NI and Bogotá are reported in Supplementary Tables S18, S19. Significant interaction effects were observed for self-report smoking behavior, knowledge, self-report descriptive smoking norms (scale 2), and perceived addiction risks.

**Table 5:** Results of structural equation models with intervention × setting interactions, including data from MECHANISMS schools.

Outcome^a^	*n*	Intervention (ASSIST vs. DC)^b^			Setting (Bogotá vs. NI)^c^			Interaction (Intervention × Setting)^d^			Model fit statistics^a^^,^^e^							Power^h^
		b	SE	*p*-value	b	SE	*p*-value	b	SE	*p*-value	χ^2^, *p*-value^f^	df	CFI^f^	TLI^f^	RMSEA (90% CI)^f^	SRMR	R^2,^^g^	
Observed outcome variables (O)																		
Donation to ASSIST/Dead Cool (0 to 10)	1,141	0.22	0.26	0.3835	0.45	0.24	0.0603	0.06	0.32	0.8551	0.00, *p* = 1.00	0	1.000	1.000	0.000 (0.000, 0.000)	0.000	0.110	0.844
Self-report smoking behavior (1 to 4)	1,190	−0.09	0.05	0.0757	**−0.12**	**0.04**	**0.0045**	**0.20**	**0.07**	**0.0021**	0.00, *p* = 1.00	0	1.000	1.000	0.000 (0.000, 0.000)	0.000	0.480	0.836
Objectively measured smoking behavior (0 to 30)	1,133	−0.02	0.10	0.8649	**1.22**	**0.13**	**<0.0001** ^**j**^	−0.09	0.18	0.6277	0.00, *p* = 1.00	0	1.000	1.000	0.000 (0.000, 0.000)	0.000	0.247	1.000
Intentions (1 to 6)	1,187	−0.17	0.09	0.0529	−0.10	0.09	0.2271	−0.09	0.13	0.5077	0.00, *p* = 1.00	0	1.000	1.000	0.000 (0.000, 0.000)	0.000	0.120	0.955
Smoking susceptibility (0 = not susceptible; 1 = susceptible)^i^	1,087	0.09	0.15	0.5483	**0.27**	**0.13**	**0.0413**	0.22	0.18	0.2333	0.00, *p* = 1.00	0	1.000	1.000	0.000 (0.000, 0.000)	0.000	0.357	1.000
Knowledge (0 to 6)	1,188	−0.16	0.13	0.2331	−0.19	0.12	0.1136	**−0.40**	**0.17**	**0.0180**	0.00, *p* = 1.00	0	1.000	1.000	0.000 (0.000, 0.000)	0.000	0.280	1.000
Experiment descriptive smoking norms (P3Q1; −1 to +1)	1,142	0.01	0.05	0.8267	0.03	0.04	0.4988	0.03	0.06	0.5895	0.00, *p* = 1.00	0	1.000	1.000	0.000 (0.000, 0.000)	0.000	0.180	0.326
Experiment descriptive vaping norms (P3Q2; −1 to +1)	1,141	0.02	0.05	0.6751	0.01	0.05	0.7657	−0.06	0.07	0.3714	0.00, *p* = 1.00	0	1.000	1.000	0.000 (0.000, 0.000)	0.000	0.235	0.150
Perceived behavioral control (easy to quit; 1 to 5)	1,182	0.12	0.13	0.3561	**0.70**	**0.13**	**<0.0001** ^**j**^	0.04	0.17	0.8238	0.00, *p* = 1.00	0	1.000	1.000	0.000 (0.000, 0.000)	0.000	0.212	1.000
Perceived behavioral control (to avoid smoking; 1 to 5)	1,188	**−0.22**	**0.10**	**0.0204**	**−0.37**	**0.10**	**0.0003** ^**j**^	0.14	0.14	0.3283	0.00, *p* = 1.00	0	1.000	1.000	0.000 (0.000, 0.000)	0.000	0.066	0.979
Exposure to advertising in the media (0 to 8)	1,184	**−1.45**	**0.20**	**<0.0001** ^**j**^	0.35	0.19	0.0595	0.43	0.25	0.0894	0.00, *p* = 1.00	0	1.000	1.000	0.000 (0.000, 0.000)	0.000	0.266	1.000
Exposure to advertising in shops (0 to 4)	1,177	−0.11	0.12	0.3466	**−0.32**	**0.10**	**0.0021** ^**j**^	−0.05	0.15	0.7357	0.00, *p* = 1.00	0	1.000	1.000	0.000 (0.000, 0.000)	0.000	0.191	0.994
Latent outcome variables (L)																		
Attitudes (AT1 to AT12, excluding AT2)	1,190	−0.17	0.09	0.0524	0.01	0.08	0.9138	0.10	0.13	0.4437	(1) 1360.53, *p* < 0.0001	416	(1) 0.882	(1) 0.878	(1) 0.044 (0.041, 0.046)	0.051	0.187	(1) 0.443
											(2) 1201.27, *p* < 0.0001		(2) 0.888	(2) 0.885	(2) 0.043 (0.040, 0.046)			(2) 0.503
Experiment injunctive smoking/vaping norms (P2S2 to P2S9)	1,190	**0.21**	**0.08**	**0.0106**	0.08	0.08	0.3077	−0.22	0.12	0.0758	(1) 816.77, *p* < 0.0001	251	(1) 0.897	(1) 0.892	(1) 0.044 (0.040, 0.047)	0.048	0.268	(1) 0.705
											(2) 658.85, *p* < 0.0001		(2) 0.908	(2) 0.903	(2) 0.042 (0.038, 0.046)			(2) 0.832
Experiment injunctive smoking norms (P2S2 to P2S6 and P2S9)	1,190	0.16	0.09	0.0768	0.09	0.09	0.2978	−0.17	0.14	0.2188	(1) 448.85, *p* < 0.0001	158	(1) 0.896	(1) 0.886	(1) 0.039 (0.035, 0.044)	0.042	0.165	(1) 0.930
											(2) 361.95, *p* < 0.0001		(2) 0.911	(2) 0.902	(2) 0.037 (0.032, 0.043)			(2) 0.983
Experiment injunctive vaping norms (P2S7 to P2S8)	1,190	**0.18**	**0.08**	**0.0185**	0.001	0.07	0.9842	−0.17	0.11	0.1390	(1) 101.13, *p* < 0.0001	32	(1) 0.948	(1) 0.932	(1) 0.043 (0.033, 0.052)	0.027	0.097	(1) 0.832
											(2) 98.67, *p* < 0.0001		(2) 0.949	(2) 0.933	(2) 0.044 (0.034, 0.054)			(2) 0.884
Experiment descriptive smoking/vaping norms (P3Q1 to P3Q2)	1,190	0.04	0.10	0.6941	0.06	0.10	0.4979	−0.02	0.14	0.8750	(1) 129.51, *p* < 0.0001	29	(1) 0.955	(1) 0.935	(1) 0.054 (0.045, 0.064)	0.038	0.258	(1) 0.857
											(2) 122.24, *p* < 0.0001		(2) 0.955	(2) 0.935	(2) 0.056 (0.046, 0.066)			(2) 0.907
Self-report injunctive smoking norms (IN1 to IN7)	1,190	−0.09	0.11	0.4097	−0.04	0.09	0.6716	0.08	0.16	0.6050	(1) 636.08, *p* < 0.0001	204	(1) 0.918	(1) 0.913	(1) 0.042 (0.039, 0.046)	0.051	0.315	(1) 0.032
											(2) 517.46, *p* < 0.0001		(2) 0.928	(2) 0.924	(2) 0.041 (0.036, 0.045)			(2) 0.053
Self-report descriptive smoking norms 1 (DN1.1 to DN1.5)	1,190	−0.12	0.13	0.3371	0.05	0.09	0.5736	0.18	0.15	0.2370	(1) 514.95, *p* < 0.0001	125	(1) 0.924	(1) 0.918	(1) 0.051 (0.047, 0.056)	0.061	0.446	(1) 0.158
											(2) 363.06, *p* < 0.0001		(2) 0.938	(2) 0.933	(2) 0.048 (0.042, 0.054)			(2) 0.282
Self-report descriptive smoking norms 2 (DN2.1 to DN2.3)	1,190	**−0.39**	**0.13**	**0.0020**	0.06	0.09	0.4988	**0.44**	**0.16**	**0.0075**	(1) 232.46, *p* < 0.0001	59	(1) 0.902	(1) 0.885	(1) 0.050 (0.043, 0.057)	0.046	0.360	(1) 0.621
											(2) 181.25, *p* < 0.0001		(2) 0.916	(2) 0.902	(2) 0.048 (0.040, 0.056)			(2) 0.727
Self-efficacy (emotional; SEE1 to SEE9)	1,190	−0.06	0.08	0.4771	**−0.18**	**0.08**	**0.0234**	0.01	0.12	0.9254	(1) 1102.82, *p* < 0.0001	299	(1) 0.967	(1) 0.965	(1) 0.048 (0.045, 0.051)	0.047	0.228	(1) 0.000
											(2) 494.85, *p* < 0.0001		(2) 0.982	(2) 0.981	(2) 0.036 (0.030, 0.041)			(2) 0.011
Self-efficacy (friends; SEF1 to SEF9)	1,190	0.05	0.08	0.5451	−0.07	0.08	0.3316	−0.12	0.12	0.3097	(1) 1246.03, *p* < 0.0001	301	(1) 0.959	(1) 0.957	(1) 0.051 (0.048, 0.054)	0.055	0.235	(1) 0.000
											(2) 629.85, *p* < 0.0001		(2) 0.971	(2) 0.970	(2) 0.044 (0.039, 0.049)			(2) 0.002
Self-efficacy (opportunity; SEO1 to SEO11)	1,190	0.01	0.08	0.9360	−0.07	0.08	0.3776	−0.16	0.12	0.1804	(1) 2272.80, *p* < 0.0001	394	(1) 0.945	(1) 0.940	(1) 0.063 (0.061, 0.066)	0.047	0.147	(1) 0.000
											(2) 716.30, *p* < 0.0001		(2) 0.971	(2) 0.968	(2) 0.047 (0.041, 0.053)			(2) 0.000
Perceived physical risks (RP1 to RP7)	1,190	0.004	0.08	0.9595	0.08	0.08	0.3155	−0.22	0.12	0.0598	(1) 1167.86, *p* < 0.0001	203	(1) 0.884	(1) 0.876	(1) 0.063 (0.060, 0.067)	0.076	0.221	(1) 0.929
											(2) 1075.81, *p* < 0.0001		(2) 0.886	(2) 0.878	(2) 0.064 (0.061, 0.068)			(2) 0.928
Perceived social risks (RS1 to RS3)	1,190	−0.01	0.09	0.9416	−0.09	0.09	0.2851	−0.20	0.14	0.1470	(1) 249.72, *p* < 0.0001	58	(1) 0.895	(1) 0.875	(1) 0.053 (0.046, 0.060)	0.063	0.195	(1) 0.446
											(2) 238.04, *p* < 0.0001		(2) 0.898	(2) 0.878	(2) 0.053 (0.047, 0.060)			(2) 0.461
Perceived addiction risks (RA1 to RA3)	1,190	0.14	0.09	0.1114	**−0.34**	**0.10**	**0.0005** ^**j**^	**−0.29**	**0.13**	**0.0204**	(1) 438.64, *p* < 0.0001	58	(1) 0.744	(1) 0.695	(1) 0.074 (0.068, 0.081)	0.066	0.181	(1) 0.999
											(2) 436.61, *p* < 0.0001		(2) 0.751	(2) 0.704	(2) 0.076 (0.070, 0.083)			(2) 0.999
Perceived benefits (BE1 to BE5)	1,190	0.03	0.09	0.7578	0.03	0.08	0.6750	−0.13	0.12	0.2879	(1) 386.72, *p* < 0.0001	124	(1) 0.949	(1) 0.944	(1) 0.042 (0.037, 0.047)	0.044	0.143	(1) 0.310
											(2) 331.52, *p* < 0.0001		(2) 0.954	(2) 0.949	(2) 0.042 (0.036, 0.047)			(2) 0.397

a b = unstandardized regression coefficient; SE = standard error; χ^2^ = Chi-square Goodness of Fit statistic; df = degrees of freedom (χ^2^ test); CFI = Comparative Fit Index; TLI = Tucker Lewis Index; RMSEA = Root Mean Square Error of Approximation; CI = confidence interval; SRMR = Standardized Root Mean Square Residual; R^2^ = R-squared; (O) = observed variable; (L) = latent variable.

b Regression coefficients represent the average change in the observed/latent outcome variable for ASSIST schools compared to Dead Cool schools, adjusted for baseline values of the outcome variable. In addition to baseline values of the outcome, all models also include the following baseline variables as covariates: setting (0 = Northern Ireland, 1 = Bogotá), intervention × setting interaction, gender (0 = boy, 1 = girl/prefer not to say), age (1 = 12 years or less, 2 = 13 years, 3 = 14 years or more; entered into SEMs as two dummy variables), ethnicity (0 = No ethnic minority, 1 = ethnic minority), and socio-economic status (NI: 1 = NIMDM2017 ≤ 296.6, 2 = 296.6 < NIMDM2017 ≤ 593.2, 3 = NIMDM2017 > 593.2; Bogotá: 1 = Informal settlement/Lowest/Low, 2 = Middle-Low/Middle, 3 = Middle-High/High; entered into SEMs as two dummy variables). See Supplementary Table S3 for details of variables. Bold and underlined text indicates tests which were significant at the *p* < 0.05 level. Structural equation models were specified using maximum likelihood estimation with robust (Huber-White) standard errors (MLR estimator) and imputation of missing data by full information maximum likelihood (FIML). Structural equation models with smoking susceptibility as the outcome variable were specified using the weighted least square mean and variance adjusted (WLSMV) estimator. Models were run including the longitudinal measurement invariance constraints on the factor loadings, intercepts, and residual variances for latent variables (as outlined in Supplementary Tables S7, S8). Unstandardized parameter values are reported in Table 5 (std.lv, which standardizes all latent variables in the model only). Standardized parameter values are reported in Supplementary Table S17 (std.all, which standardizes all latent and observed variables in the model).

c Regression coefficients represent the average change in the observed/latent outcome variable for Bogotá schools compared to Northern Ireland schools, adjusted for baseline values of the outcome variable, intervention (0 = Dead Cool, 1 = ASSIST), intervention × setting interaction, gender, age, ethnicity, and socio-economic status. Bold and underlined text indicates tests which were significant at the *p* < 0.05 level.

d Regression coefficients represent the average change in the intervention effect on the observed/latent outcome variable for Bogotá schools compared to Northern Ireland schools adjusted for baseline values of the outcome variable, intervention, setting, gender, age, ethnicity, and socio-economic status. Bold and underlined text indicates tests which were significant at the *p* < 0.05 level.

e Model fit statistics were estimated using the robust (Huber-White) maximum likelihood (MLR) estimator. For models with smoking susceptibility as the outcome variable, model fit statistics were estimated using the weighted least square mean and variance adjusted (WLSMV) estimator.

f (1) standard test statistic; (2) robust test statistic. For models with observed outcome variables (O), the results were the same for the standard and robust tests.

g R-squared: the percentage of variation in the outcome variable explained by the model.

h Power to detect the observed “Intervention” and “Setting” effects with a significance level of *p* < 0.05, calculated using WebPower. For models with observed outcome variables (O), the “linear regression” method was used. For models with latent outcome variables (L), the “power analysis based on the RMSEA” method was used: (1) power calculated based on the standard RMSEA; (2) power calculated based on the robust RMSEA.

i Structural equation models with smoking susceptibility as the outcome variable were specified using the weighted least square mean and variance adjusted (WLSMV) estimator.

j Retained statistical significance at the 5% level after using the Holm-Bonferroni procedure to correct the *p*-values for multiple testing (*p* < 0.05; based on the number of models in Table 5, i.e., 23 tests).

In NI, self-report smoking behavior was more anti-smoking at follow-up controlling for baseline for Dead Cool compared to ASSIST, whereas the opposite pattern was observed for Bogotá. Knowledge of the effects of smoking was higher for Dead Cool compared to ASSIST in Bogotá, with no significant differences between interventions in NI. Dead Cool schools showed more anti-smoking values of self-report descriptive smoking norms (scale 2) in NI, but there was no significant difference between interventions in Bogotá. Differences in perceived addiction risks between interventions were non-significant in both NI and Bogotá. The negative interaction effect appeared to be driven by the overall lower perceived addiction risks in Bogotá.

The results of SEMs with intervention × setting interactions comparing ASSIST non-PS and ASSIST PS separately with Dead Cool are reported in Table 6 and Supplementary Table S20. The results of SEMs examining the effects separately for NI and Bogotá are reported in Supplementary Tables S21, S22. For ASSIST non-PS, there were significant interaction effects for self-report smoking behavior, knowledge, self-report descriptive smoking norms (scale 2), and perceived physical, social, and addiction risks. The direction of effects was consistent with the overall ASSIST versus Dead Cool comparison. In addition, perceived physical and social risks were higher for Dead Cool compared to ASSIST non-PS in Bogotá at follow-up, with non-significant differences between groups in NI.

**Table 6:** Results of structural equation models with intervention × setting interactions comparing ASSIST non-peer supporters and ASSIST peer supporters separately with Dead Cool, including data from MECHANISMS schools.

Outcome^a^	*n*	Intervention (ASSIST non-PS vs. DC)^b^			Peer supporters (ASSIST PS vs. DC)^c^			Setting (Bogotá vs. NI)^d^			Interaction (intervention × setting)^e^			Interaction (peer supporters × Setting)^f^			Model fit statistics^a^^,^^g^							Power^j^
		b	SE	*p*-value	b	SE	*p*-value	b	SE	*p*-value	b	SE	*p*-value	b	SE	*p*-value	χ^2^, *p*-value^h^	df	CFI^h^	TLI^h^	RMSEA (90% CI)^h^	SRMR	R^2,^^i^	
Observed outcome variables (O)																								
Donation to ASSIST/Dead Cool	1,141	0.26	0.28	0.3369	0.10	0.37	0.7975	0.45	0.24	0.0603	−0.01	0.34	0.9693	0.32	0.49	0.5161	0.00, *p* = 1.00	0	1.000	1.000	0.000 (0.000, 0.000)	0.000	0.110	0.786
Self-report smoking behavior	1,190	**−0.12**	**0.06**	**0.0293**	0.04	0.07	0.5971	**−0.12**	**0.04**	**0.0047**	**0.23**	**0.07**	**0.0009** ^**l**^	0.09	0.11	0.4180	0.00, *p* = 1.00	0	1.000	1.000	0.000 (0.000, 0.000)	0.000	0.482	0.912
Objectively measured smoking behavior	1,133	−0.03	0.11	0.7920	0.03	0.15	0.8557	**1.22**	**0.13**	**<0.0001** ^**l**^	−0.10	0.19	0.5896	−0.02	0.28	0.9421	0.00, *p* = 1.00	0	1.000	1.000	0.000 (0.000, 0.000)	0.000	0.247	1.000
Intentions	1,187	−0.16	0.09	0.0616	−0.20	0.18	0.2529	−0.10	0.09	0.2301	−0.13	0.14	0.3483	0.09	0.23	0.7019	0.00, *p* = 1.00	0	1.000	1.000	0.000 (0.000, 0.000)	0.000	0.122	0.951
Smoking susceptibility^k^	1,087	0.03	0.16	0.8321	0.28	0.21	0.1788	**0.27**	**0.13**	**0.0411**	0.30	0.19	0.1248	−0.10	0.30	0.7471	0.00, *p* = 1.00	0	1.000	1.000	0.000 (0.000, 0.000)	0.000	0.359	1.000
Knowledge	1,188	**−0.31**	**0.14**	**0.0234**	**0.46**	**0.22**	**0.0318**	−0.19	0.12	0.1079	**−0.43**	**0.17**	**0.0131**	−0.25	0.28	0.3685	0.00, *p* = 1.00	0	1.000	1.000	0.000 (0.000, 0.000)	0.000	0.309	1.000
Experiment descriptive smoking norms	1,142	0.004	0.05	0.9335	0.03	0.07	0.6505	0.03	0.04	0.4991	0.05	0.07	0.4609	−0.02	0.09	0.7901	0.00, *p* = 1.00	0	1.000	1.000	0.000 (0.000, 0.000)	0.000	0.181	0.303
Experiment descriptive vaping norms	1,141	0.03	0.05	0.6374	0.01	0.08	0.9037	0.01	0.05	0.7665	−0.06	0.07	0.3843	−0.06	0.11	0.5862	0.00, *p* = 1.00	0	1.000	1.000	0.000 (0.000, 0.000)	0.000	0.235	0.133
Perceived behavioral control (easy to quit)	1,182	0.13	0.14	0.3529	0.09	0.20	0.6595	**0.70**	**0.13**	**<0.0001** ^**l**^	0.03	0.18	0.8786	0.08	0.26	0.7567	0.00, *p* = 1.00	0	1.000	1.000	0.000 (0.000, 0.000)	0.000	0.212	1.000
Perceived behavioral control (to avoid smoking)	1,188	**−0.26**	**0.10**	**0.0135**	−0.10	0.15	0.5113	**−0.37**	**0.10**	**0.0004** ^**l**^	0.10	0.15	0.4811	0.27	0.22	0.2115	0.00, *p* = 1.00	0	1.000	1.000	0.000 (0.000, 0.000)	0.000	0.070	0.988
Exposure to advertising in the media	1,184	**−1.44**	**0.21**	**<0.0001** ^**l**^	**−1.51**	**0.31**	**<0.0001** ^**l**^	0.35	0.18	0.0591	0.31	0.27	0.2379	**0.91**	**0.40**	**0.0224**	0.00, *p* = 1.00	0	1.000	1.000	0.000 (0.000, 0.000)	0.000	0.268	1.000
Exposure to advertising in shops	1,177	−0.13	0.13	0.2971	−0.03	0.19	0.8683	**−0.32**	**0.10**	**0.0020** ^**l**^	0.01	0.16	0.9393	−0.30	0.23	0.1992	0.00, *p* = 1.00	0	1.000	1.000	0.000 (0.000, 0.000)	0.000	0.193	0.993
Latent outcome variables (L)																								
Attitudes	1,190	**−0.19**	**0.09**	**0.0405**	−0.11	0.17	0.5292	0.01	0.08	0.9062	0.10	0.14	0.4946	0.12	0.22	0.5888	(1) 1397.16, *p* < 0.0001	458	(1) 0.882	(1) 0.879	(1) 0.042 (0.039, 0.044)	0.048	0.187	(1) 0.065
																	(2) 1245.71, *p* < 0.0001		(2) 0.889	(2) 0.885	(2) 0.041 (0.038, 0.044)			(2) 0.090
Experiment injunctive smoking/vaping norms	1,190	**0.20**	**0.09**	**0.0209**	0.24	0.14	0.0895	0.08	0.08	0.3068	−0.20	0.13	0.1262	−0.29	0.21	0.1526	(1) 842.40, *p* < 0.0001	281	(1) 0.898	(1) 0.892	(1) 0.041 (0.038, 0.044)	0.044	0.268	(1) 0.255
																	(2) 693.86, *p* < 0.0001		(2) 0.908	(2) 0.903	(2) 0.040 (0.036, 0.044)			(2) 0.437
Experiment injunctive smoking norms	1,190	0.16	0.10	0.0916	0.14	0.14	0.3332	0.09	0.09	0.3005	−0.16	0.15	0.2851	−0.22	0.21	0.3000	(1) 468.04, *p* < 0.0001	180	(1) 0.897	(1) 0.887	(1) 0.037 (0.033, 0.041)	0.038	0.165	(1) 0.728
																	(2) 386.15, *p* < 0.0001		(2) 0.912	(2) 0.903	(2) 0.035 (0.030, 0.040)			(2) 0.912
Experiment injunctive vaping norms	1,190	**0.16**	**0.08**	**0.0494**	0.25	0.13	0.0602	0.002	0.07	0.9763	−0.14	0.12	0.2405	−0.26	0.19	0.1720	(1) 108.53, *p* < 0.0001	38	(1) 0.947	(1) 0.931	(1) 0.039 (0.031, 0.048)	0.023	0.098	(1) 0.725
																	(2) 105.61, *p* < 0.0001		(2) 0.948	(2) 0.932	(2) 0.041 (0.032, 0.050)			(2) 0.801
Experiment descriptive smoking/vaping norms	1,190	0.03	0.11	0.7556	0.07	0.16	0.6761	0.07	0.10	0.4979	0.001	0.14	0.9952	−0.11	0.21	0.6050	(1) 132.16, *p* < 0.0001	35	(1) 0.957	(1) 0.938	(1) 0.048 (0.040, 0.057)	0.032	0.257	(1) 0.602
																	(2) 124.40, *p* < 0.0001		(2) 0.957	(2) 0.938	(2) 0.050 (0.041, 0.059)			(2) 0.688
Self-report injunctive smoking norms	1,190	−0.12	0.12	0.3269	0.03	0.18	0.8682	−0.04	0.09	0.6763	0.10	0.17	0.5342	−0.02	0.28	0.9531	(1) 670.17, *p* < 0.0001	230	(1) 0.917	(1) 0.911	(1) 0.040 (0.037, 0.044)	0.048	0.314	(1) 0.002
																	(2) 559.07, *p* < 0.0001		(2) 0.927	(2) 0.922	(2) 0.039 (0.035, 0.043)			(2) 0.006
Self-report descriptive smoking norms 1	1,190	−0.14	0.14	0.3110	−0.06	0.20	0.7830	0.05	0.09	0.5723	0.23	0.16	0.1459	−0.03	0.25	0.8925	(1) 532.50, *p* < 0.0001	143	(1) 0.924	(1) 0.918	(1) 0.048 (0.044, 0.052)	0.055	0.446	(1) 0.012
																	(2) 381.88, *p* < 0.0001		(2) 0.939	(2) 0.933	(2) 0.045 (0.039, 0.051)			(2) 0.036
Self-report descriptive smoking norms 2	1,190	**−0.36**	**0.14**	**0.0102**	**−0.55**	**0.20**	**0.0069**	0.06	0.09	0.5046	**0.39**	**0.18**	**0.0287**	**0.64**	**0.27**	**0.0178**	(1) 240.79, *p* < 0.0001	69	(1) 0.903	(1) 0.886	(1) 0.046 (0.040, 0.052)	0.040	0.360	(1) 0.282
																	(2) 189.33, *p* < 0.0001		(2) 0.918	(2) 0.904	(2) 0.044 (0.036, 0.051)			(2) 0.382
Self-efficacy (emotional)	1,190	−0.06	0.09	0.5397	−0.07	0.12	0.5549	**−0.18**	**0.08**	**0.0238**	−0.02	0.13	0.8887	0.14	0.19	0.4632	(1) 1150.74, *p* < 0.0001	333	(1) 0.966	(1) 0.964	(1) 0.045 (0.043, 0.048)	0.044	0.229	(1) 0.000
																	(2) 546.79, *p* < 0.0001		(2) 0.981	(2) 0.980	(2) 0.034 (0.029, 0.040)			(2) 0.001
Self-efficacy (friends)	1,190	0.06	0.08	0.4586	−0.01	0.14	0.9460	−0.07	0.08	0.3333	−0.17	0.13	0.1793	0.09	0.19	0.6588	(1) 1298.07, *p* < 0.0001	335	(1) 0.958	(1) 0.956	(1) 0.049 (0.046, 0.052)	0.051	0.236	(1) 0.000
																	(2) 687.81, *p* < 0.0001		(2) 0.971	(2) 0.969	(2) 0.042 (0.038, 0.047)			(2) 0.000
Self-efficacy (opportunity)	1,190	0.02	0.08	0.7843	−0.06	0.16	0.6948	−0.07	0.08	0.3770	−0.21	0.13	0.1160	0.01	0.22	0.9480	(1) 2306.74, *p* < 0.0001	436	(1) 0.945	(1) 0.940	(1) 0.060 (0.058, 0.062)	0.044	0.148	(1) 0.000
																	(2) 781.54, *p* < 0.0001		(2) 0.971	(2) 0.968	(2) 0.044 (0.039, 0.050)			(2) 0.000
Perceived physical risks	1,190	−0.01	0.09	0.9128	0.05	0.13	0.6962	0.08	0.08	0.3009	**−0.34**	**0.13**	**0.0080**	0.25	0.20	0.2129	(1) 1218.56, *p* < 0.0001	229	(1) 0.882	(1) 0.874	(1) 0.060 (0.057, 0.064)	0.071	0.228	(1) 0.581
																	(2) 1134.71, *p* < 0.0001		(2) 0.883	(2) 0.875	(2) 0.061 (0.058, 0.065)			(2) 0.591
Perceived social risks	1,190	0.04	0.10	0.6836	−0.22	0.17	0.2145	−0.09	0.09	0.2721	**−0.34**	**0.14**	**0.0200**	0.40	0.24	0.0952	(1) 263.10, *p* < 0.0001	68	(1) 0.894	(1) 0.873	(1) 0.049 (0.043, 0.055)	0.057	0.199	(1) 0.168
																	(2) 252.28, *p* < 0.0001		(2) 0.896	(2) 0.876	(2) 0.050 (0.043, 0.056)			(2) 0.183
Perceived addiction risks	1,190	0.07	0.09	0.4377	**0.44**	**0.14**	**0.0013** ^**l**^	**−0.33**	**0.10**	**0.0007** ^**l**^	**−0.30**	**0.13**	**0.0229**	−0.25	0.21	0.2366	(1) 463.36, *p* < 0.0001	68	(1) 0.738	(1) 0.688	(1) 0.070 (0.064, 0.076)	0.060	0.195	(1) 0.993
																	(2) 459.54, *p* < 0.0001		(2) 0.746	(2) 0.697	(2) 0.072 (0.066, 0.078)			(2) 0.993
Perceived benefits	1,190	0.02	0.09	0.8315	0.06	0.16	0.7257	0.03	0.08	0.6624	−0.17	0.12	0.1716	0.05	0.23	0.8308	(1) 415.21, *p* < 0.0001	142	(1) 0.947	(1) 0.942	(1) 0.040 (0.036, 0.045)	0.042	0.147	(1) 0.126
																	(2) 362.72, *p* < 0.0001		(2) 0.952	(2) 0.947	(2) 0.040 (0.035, 0.045)			(2) 0.201

a PS = peer supporters; DC = Dead Cool; NI = Northern Ireland; b = unstandardized regression coefficient; SE = standard error; χ^2^ = Chi-square Goodness of Fit statistic; df = degrees of freedom (χ^2^ test); CFI = Comparative Fit Index; TLI = Tucker Lewis Index; RMSEA = Root Mean Square Error of Approximation; CI = confidence interval; SRMR = Standardized Root Mean Square Residual; R^2^ = R-squared; (O) = observed variable; (L) = latent variable.

b Regression coefficients represent the average change in the observed/latent outcome variable for ASSIST non-peer supporters compared to Dead Cool schools, adjusted for baseline values of the outcome variable. In addition to baseline values of the outcome, all models also include the following baseline variables as covariates: ASSIST peer supporters (0 = Dead Cool and ASSIST non-peer supporters, 1 = ASSIST peer supporters), setting (0 = Northern Ireland, 1 = Bogotá), intervention × setting interaction, peer supporters × setting interaction, gender (0 = boy, 1 = girl/prefer not to say), age (1 = 12 years or less, 2 = 13 years, 3 = 14 years or more; entered into SEMs as two dummy variables), ethnicity (0 = No ethnic minority, 1 = ethnic minority), and socio-economic status (NI: 1 = NIMDM2017 ≤ 296.6, 2 = 296.6 < NIMDM2017 ≤ 593.2, 3 = NIMDM2017 > 593.2; Bogotá: 1 = Informal settlement/Lowest/Low, 2 = Middle-Low/Middle, 3 = Middle-High/High; entered into SEMs as two dummy variables). See Supplementary Table S3 for details of variables. Bold and underlined text indicates tests which were significant at the *p* < 0.05 level. Structural equation models were specified using maximum likelihood estimation with robust (Huber-White) standard errors (MLR estimator) and imputation of missing data by full information maximum likelihood (FIML). Structural equation models with smoking susceptibility as the outcome variable were specified using the weighted least square mean and variance adjusted (WLSMV) estimator. Models were run including the longitudinal measurement invariance constraints on the factor loadings, intercepts, and residual variances for latent variables (as outlined in Supplementary Tables S7, S8). Unstandardized parameter values are reported in Table 6 (std.lv, which standardizes all latent variables in the model only). Standardized parameter values are reported in Supplementary Table S20 (std.all, which standardizes all latent and observed variables in the model).

c Regression coefficients represent the average change in the observed/latent outcome variable for ASSIST peer supporters compared to Dead Cool schools, adjusted for baseline values of the outcome variable, intervention (0 = Dead Cool and ASSIST peer supporters, 1 = ASSIST non-peer supporters), setting, intervention × setting interaction, peer supporters × setting interaction, gender, age, ethnicity, and socio-economic status. Bold and underlined text indicates tests which were significant at the *p* < 0.05 level.

d Regression coefficients represent the average change in the observed/latent outcome variable for Bogotá schools compared to Northern Ireland schools, adjusted for baseline values of the outcome variable, intervention (0 = Dead Cool and ASSIST peer supporters, 1 = ASSIST non-peer supporters), ASSIST peer supporters (0 = Dead Cool and ASSIST non-peer supporters, 1 = ASSIST peer supporters), intervention × setting interaction, peer supporters × setting interaction, gender, age, ethnicity, and socio-economic status. Bold and underlined text indicates tests which were significant at the *p* < 0.05 level.

e Regression coefficients represent the average change in the intervention effect on the observed/latent outcome variable for Bogotá schools compared to Northern Ireland schools adjusted for baseline values of the outcome variable, intervention, setting, ASSIST peer supporters, peer supporters × setting interaction, gender, age, ethnicity, and socio-economic status. Bold and underlined text indicates tests which were significant at the *p* < 0.05 level.

f Regression coefficients represent the average change in the peer supporters effect on the observed/latent outcome variable for Bogotá schools compared to Northern Ireland schools adjusted for baseline values of the outcome variable, intervention, setting, ASSIST peer supporters, intervention × setting interaction, gender, age, ethnicity, and socio-economic status. Bold and underlined text indicates tests which were significant at the *p* < 0.05 level.

g Model fit statistics were estimated using the robust (Huber-White) maximum likelihood (MLR) estimator. For models with smoking susceptibility as the outcome variable, model fit statistics were estimated using the weighted least square mean and variance adjusted (WLSMV) estimator.

h (1) standard test statistic; (2) robust test statistic. For models with observed outcome variables (O), the results were the same for the standard and robust tests.

i R-squared: the percentage of variation in the outcome variable explained by the model.

j Power to detect the observed “Intervention” and “Setting” effects with a significance level of *p* < 0.05, calculated using WebPower. For models with observed outcome variables (O), the “linear regression” method was used. For models with latent outcome variables (L), the “power analysis based on the RMSEA” method was used: (1) power calculated based on the standard RMSEA; (2) power calculated based on the robust RMSEA.

k Structural equation models with smoking susceptibility as the outcome variable were specified using the weighted least square mean and variance adjusted (WLSMV) estimator.

l Retained statistical significance at the 5% level after using the Holm-Bonferroni procedure to correct the *p*-values for multiple testing (*p* < 0.05; based on the number of models in Table 6, i.e., 23 tests).

For ASSIST PS, significant interactions were observed for exposure to advertising in the media, and self-report descriptive smoking norms (scale 2). Exposure to advertising was lower for ASSIST PS than Dead Cool in both settings at follow-up, with stronger differences in NI. Dead Cool also showed more anti-smoking values on self-report descriptive smoking norms compared to ASSIST PS in NI, with non-significant differences in Bogotá.

### 3.2 Single and multiple mediator models

#### 3.2.1 Mediator models with intervention group as the predictor variable

The results of the final single and multiple mediator models with intervention group (ASSIST versus Dead Cool) as the predictor variable are reported in Table 7 (unstandardized parameter estimates) and Supplementary Table S23 (standardized parameter estimates). R^2^ ranged between 0.125 and 0.565. Power to detect the indirect effects ranged between 0.061 and 1.000.

**Table 7:** Results of final single and multiple mediator models with intervention group (ASSIST versus Dead Cool) as the predictor variable, including data from MECHANISMS schools.

Mediator^a^	*n*	Intervention group effect on mediator^b^			Association of mediator with outcome^b^			Direct effect^b^			Indirect effect^b^			Proportion mediated (%)^c^	Power (SE)^d^	Model fit statistics^a^^,^^e^						
		a	SE	*p*-value	b	SE	*p*-value	c’	SE	*p*-value	ab	SE	95% CI	[ab/(c’ + Σab)] × 100		χ^2^, *p*-value^f^	df	CFI^f^	TLI^f^	RMSEA (90% CI)^f^	SRMR	R^2,^^g^
Model 1: experimental donations to ASSIST/Dead Cool (O) as the outcome variable																						
Perceived social risks (L)	1,136	**−0.15**	**0.07**	**0.0360**	0.20	0.10	0.0558	0.23	0.16	0.1611	**−0.03**	**0.02**	**−0.09, −0.001**	**−9.87**	0.988 (0.003)	(1) 243.57, *p* < 0.0001		(1) 0.921	(1) 0.882	(1) 0.046 (0.040, 0.052)	0.052	0.125
Exposure to advertising in the media (O)		**−1.19**	**0.12**	**<0.0001** ^**i**^	**−0.08**	**0.04**	**0.0368**				**0.10**	**0.05**	**0.01, 0.20**	**33.35**	0.794 (0.013)	(2) 236.89, *p* < 0.0001		(2) 0.924	(2) 0.886	(2) 0.046 (0.040, 0.053)		
Model 2: self-report smoking behavior (O) as the outcome variable																						
Intentions (O)	1,186	**−0.18**	**0.07**	**0.0111**	**0.08**	**0.02**	**0.0002** ^**i**^	**0.07**	**0.03**	**0.0215**	**−0.01**	**0.01**	**−0.03, −0.004**	**−36.75**	0.771 (0.013)	(1) 3137.08, *p* < 0.0001	1,094	(1) 0.864	(1) 0.855	(1) 0.040 (0.038, 0.041)	0.069	0.490
Attitudes (L)		**−0.18**	**0.07**	**0.0111**	**0.08**	**0.02**	**0.0002** ^**i**^				**−0.02**	**0.01**	**−0.04, −0.01**	**−42.11**	0.683 (0.015)							
Self-report injunctive smoking norms (L)		−0.14	0.08	0.0798	0.02	0.03	0.5277				−0.003	0.00	−0.02, 0.004	−6.99	0.111 (0.010)	(2) 2343.54, *p* < 0.0001		(2) 0.876	(2) 0.867	(2) 0.039 (0.037, 0.041)		
PBC (to avoid smoking; O)		−0.13	0.07	0.0764	0.02	0.02	0.1833				−0.003	0.00	−0.01, 0.001	−7.15	0.148 (0.011)							
Model 3: self-report smoking behavior (O) as the outcome variable																						
Self-efficacy: Emotional (L)	1,184	−0.12	0.06	0.0574	**0.16**	**0.03**	**<0.0001** ^**i**^	0.06	0.03	0.0846	**−0.02**	**0.01**	**−0.04, −0.001**	**−57.97**	0.972 (0.005)	(1) 1560.89, *p* < 0.0001		(1) 0.953	(1) 0.947	(1) 0.054 (0.051, 0.057)	0.102	0.484
Exposure to advertising in the media (O)		**−1.18**	**0.12**	**<0.0001** ^**i**^	0.01	0.01	0.3933				−0.01	0.01	−0.03, 0.01	−29.22	0.061 (0.008)	(2) 583.49, *p* < 0.0001		(2) 0.969	(2) 0.966	(2) 0.045 (0.040, 0.050)		
Model 4: self-report smoking behavior (O) as the outcome variable																						
Self-efficacy: Opportunity (L)	1,190	**−0.17**	**0.07**	**0.0107**	**0.10**	**0.03**	**0.0001** ^**i**^	0.04	0.03	0.1806	**−0.02**	**0.01**	**−0.04, −0.01**	**−64.24**	0.951 (0.007)	(1) 2639.61, *p* < 0.0001	414	(1) 0.937	(1) 0.929	(1) 0.067 (0.065, 0.070)	0.089	0.470
																(2) 484.73, *p* = 0.0093		(2) 0.964	(2) 0.960	(2) 0.051 (0.046, 0.057)		
Model 5: objectively measured smoking behavior (O) as the outcome variable^h^																						
Intentions (O)	1,131	**−0.20**	**0.07**	**0.0057**	−0.08	0.07	0.2712	−0.08	0.09	0.3586	0.02	0.02	−0.01, 0.07	−25.04	0.799 (0.013)	0.00, *p* = 1.00	0	1.000	1.000	0.000 (0.000, 0.000)	0.000	0.251
Model 6: smoking intentions (O) as the outcome variable																						
Knowledge (O)	1,186	**−0.37**	**0.08**	**<0.0001** ^**i**^	0.03	0.02	0.1620	−0.09	0.07	0.1580	−0.01	0.01	−0.03, 0.004	7.39	0.213 (0.013)	(1) 5999.97, *p* < 0.0001	2,404	(1) 0.913	(1) 0.909	(1) 0.036 (0.034, 0.037)	0.072	0.221
Attitudes (L)		**−0.14**	**0.07**	**0.0441**	**0.10**	**0.05**	**0.0433**				**−0.01**	**0.01**	**−0.04, −0.001**	**8.54**	0.847 (0.011)							
Self-report injunctive smoking norms (L)		−0.08	0.08	0.3216	**0.13**	**0.05**	**0.0129**				−0.01	0.01	−0.04, 0.01	6.35	0.602 (0.015)							
																(2) 3305.87, *p* < 0.0001		(2) 0.933	(2) 0.930	(2) 0.032 (0.030, 0.033)		
Self-report descriptive smoking norms 2 (L)		**−0.16**	**0.08**	**0.0458**	0.10	0.05	0.0571				−0.02	0.01	−0.05, 0.0001	9.75	0.780 (0.013)							
Self-efficacy: Emotional (L)		−0.08	0.06	0.2039	**0.20**	**0.05**	**0.0002** ^**i**^				−0.02	0.01	−0.05, 0.01	9.71	0.764 (0.013)							
Model 7: smoking intentions (O) as the outcome variable																						
Self-efficacy: Opportunity (L)	1,187	**−0.16**	**0.06**	**0.0103**	**0.21**	**0.05**	**<0.0001** ^**i**^	**−0.18**	**0.07**	**0.0091**	**−0.03**	**0.02**	**−0.07, −0.01**	**15.83**	1.000 (0.000)	(1) 2506.36, *p* < 0.0001	414	(1) 0.938	(1) 0.932	(1) 0.065 (0.063, 0.068)	0.072	0.146
																(2) 473.79, *p* = 0.0223		(2) 0.965	(2) 0.961	(2) 0.050 (0.044, 0.055)		
Model 8: smoking susceptibility (O) as the outcome variable																						
Attitudes (L)	1,002	**−0.20**	**0.08**	**0.0120**	**−0.41**	**0.05**	**<0.0001** ^**i**^	0.09	0.09	0.3013	**0.08**	**0.04**	**0.02, 0.17**	**39.65**	1.000 (0.000)	(1) 3144.57, *p* < 0.0001	1,122	(1) 0.927	(1) 0.949	(1) 0.042 (0.041, 0.044)	0.042	0.542
Experiment injunctive smoking/vaping norms (L)		**0.18**	**0.09**	**0.0409**	**0.14**	**0.06**	**0.0135**				**0.03**	**0.02**	**0.003, 0.07**	**12.25**	0.961 (0.006)							
																(2) 2478.08, *p* < 0.0001		(2) 0.842	(2) 0.890	(2) 0.035 (0.033, 0.037)		
PBC (to avoid smoking; O)		**−0.16**	**0.08**	**0.0441**	−0.06	0.04	0.0930				0.01	0.01	−0.0002, 0.03	4.58	0.572 (0.016)							
Model 9: smoking susceptibility (O) as the outcome variable																						
Self-report injunctive smoking norms (L)	1,067	**−0.22**	**0.09**	**0.0141**	−0.31	0.18	0.0973	0.06	0.24	0.8170	**0.07**	**0.05**	**0.03, 0.54**	**30.65**	1.000 (0.000)	(1) 3934.81, *p* < 0.0001	920	(1) 0.849	(1) 0.891	(1) 0.055 (0.054, 0.057)	0.055	0.542
Self-report injunctive smoking norms (L)		**−0.37**	**0.12**	**0.0022**	−0.13	0.37	0.7176				0.05	0.15	−0.07, 0.53	21.89	0.672 (0.015)							
Self-report descriptive smoking norms 2 (L)		**−0.34**	**0.11**	**0.0025**	−0.04	0.24	0.8686				0.01	0.10	−0.13, 0.20	6.14	0.100 (0.009)	(2) 2934.08, *p* < 0.0001		(2) 0.735	(2) 0.808	(2) 0.045 (0.043, 0.047)		
Perceived social risks (L)		**−0.31**	**0.09**	**0.0003** ^**i**^	−0.12	0.09	0.1747				**0.04**	**0.03**	**0.00003, 0.11**	**16.38**	0.858 (0.011)							
Model 10: smoking susceptibility (O) as the outcome variable																						
Knowledge (O)	1,067	**−0.38**	**0.08**	**<0.0001** ^**i**^	**−0.07**	**0.03**	**0.0370**	0.09	0.09	0.3387	**0.02**	**0.01**	**0.002, 0.06**	**10.47**	0.563 (0.016)	(1) 2797.63, *p* < 0.0001	839	(1) 0.969	(1) 0.979	(1) 0.047 (0.045, 0.049)	0.038	0.565
Self-efficacy: Emotional (L)		**−0.18**	**0.08**	**0.0172**	**−0.47**	**0.07**	**<0.0001** ^**i**^				**0.09**	**0.04**	**0.02, 0.18**	**36.70**	1.000 (0.000)							
																(2) 2263.79, *p* < 0.0001		(2) 0.885	(2) 0.923	(2) 0.040 (0.038, 0.042)		
Perceived physical risks (L)		**−0.25**	**0.08**	**0.0013** ^**i**^	**−0.16**	**0.05**	**0.0006** ^**i**^				**0.04**	**0.02**	**0.01, 0.08**	**16.56**	0.998 (0.001)							
Model 11: smoking susceptibility (O) as the outcome variable																						
Self-efficacy: Opportunity (L)	1,083	**−0.17**	**0.07**	**0.0163**	**−0.42**	**0.05**	**<0.0001** ^**i**^	0.16	0.09	0.0743	**0.07**	**0.03**	**0.01, 0.14**	**30.74**	0.596 (0.016)	(1) 2282.01, *p* < 0.0001	414	(1) 0.984	(1) 0.990	(1) 0.065 (0.062, 0.067)	0.048	0.489
																(2) 1777.87, *p* < 0.0001		(2) 0.915	(2) 0.948	(2) 0.055 (0.053, 0.058)		
Model 12: knowledge of smoking (O) as the outcome variable																						
Attitudes (L)	1,187	**−0.18**	**0.07**	**0.0072**	**0.15**	**0.07**	**0.0256**	**−0.25**	**0.11**	**0.0246**	**−0.03**	**0.02**	**−0.08, −0.01**	**7.48**	0.960 (0.006)	(1) 4355.16, *p* < 0.0001	1,306	(1) 0.852	(1) 0.842	(1) 0.044 (0.043, 0.046)	0.073	0.318
Perceived physical risks (L)		**−0.22**	**0.07**	**0.0012** ^**i**^	0.20	0.11	0.0753				**−0.04**	**0.03**	**−0.14, −0.01**	**11.88**	0.856 (0.011)							
Perceived social risks (L)		**−0.23**	**0.08**	**0.0046**	0.18	0.14	0.1988				**−0.04**	**0.04**	**−0.19, −0.002**	**11.49**	0.644 (0.015)	(2) 3334.27, *p* < 0.0001		(2) 0.857	(2) 0.848	(2) 0.044 (0.043, 0.046)		
PBC (to avoid smoking; O)		**−0.14**	**0.07**	**0.0539**	0.06	0.05	0.2420				−0.01	0.01	−0.04, 0.002	2.27	0.380 (0.015)							
Model 13: attitudes towards smoking (L) as the outcome variable																						
Knowledge (O)	1,187	**−0.37**	**0.08**	**<0.0001** ^**i**^	**0.07**	**0.03**	**0.0050**	**−0.15**	**0.07**	**0.0271**	**−0.03**	**0.01**	**−0.05, −0.01**	**12.64**	0.599 (0.015)	(1) 1958.86, *p* < 0.0001	680	(1) 0.878	(1) 0.868	(1) 0.040 (0.038, 0.042)	0.054	0.253
Self-report descriptive smoking norms 2 (L)		**−0.17**	**0.07**	**0.0209**	**0.13**	**0.04**	**0.0028**				**−0.02**	**0.01**	**−0.06, −0.003**	**10.76**	0.896 (0.010)							
																(2) 1537.43, *p* < 0.0001		(2) 0.888	(2) 0.879	(2) 0.039 (0.037, 0.041)		
PBC (to avoid smoking; O)		−0.14	0.08	0.0666	**0.09**	**0.03**	**0.0004** ^**i**^				**−0.01**	**0.01**	**−0.03, −0.001**	**6.15**	0.855 (0.011)							
Model 14: attitudes towards smoking (L) as the outcome variable																						
Perceived physical risks (L)	1,176	**−0.17**	**0.06**	**0.0047**	**0.16**	**0.04**	**<0.0001** ^**i**^	−0.09	0.07	0.1763	**−0.03**	**0.01**	**−0.06, −0.01**	**27.40**	0.995 (0.002)	(1) 3076.37, *p* < 0.0001	1,024	(1) 0.880	(1) 0.873	(1) 0.041 (0.040, 0.043)	0.056	0.217
Exposure to advertising in the media (O)		**−1.18**	**0.12**	**<0.0001** ^**i**^	−0.03	0.02	0.1989				0.03	0.02	−0.02, 0.08	−31.17	0.112 (0.010)							
																(2) 2433.33, *p* < 0.0001		(2) 0.885	(2) 0.878	(2) 0.041 (0.040, 0.043)		
Exposure to advertising in shops (O)		**−0.16**	**0.07**	**0.0231**	**0.07**	**0.03**	**0.0183**				**−0.01**	**0.01**	**−0.03, −0.001**	**11.69**	0.687 (0.015)							

a a = unstandardized regression coefficient for intervention group effect on mediator; b = unstandardized regression coefficient for association of mediator with outcome; c’ = unstandardized regression coefficient for direct effect; ab = unstandardized regression coefficient for indirect effect; SE = standard error; χ^2^ = Chi-square Goodness of Fit statistic; df = degrees of freedom (χ^2^ test); CFI = Comparative Fit Index; TLI = Tucker Lewis Index; RMSEA = Root Mean Square Error of Approximation; CI = confidence interval; SRMR = Standardized Root Mean Square Residual; R^2^ = R-squared; (O) = observed variable; (L) = latent variable; PBC = Perceived behavioral control.

b Results are unstandardized regression coefficients from single and multiple mediator structural equation models (SEMs). Bias-corrected bootstrap confidence intervals for the indirect effect are reported, along with the standard errors (standard errors and *p*-values are reported for the intervention effect on the mediator, association of the mediator with the outcome, and direct effect). In each model, the predictor variable is group assignment (0 = Dead Cool; 1 = ASSIST), the mediating variable is the follow-up score of the mediator, and the dependent variable is the follow-up score of the outcome variable. In addition to baseline values of the outcome and mediator, all models also include the following baseline variables as covariates: setting (0 = Northern Ireland, 1 = Bogotá), gender (0 = boy, 1 = girl/prefer not to say), age (1 = 12 years or less, 2 = 13 years, 3 = 14 years or more; entered into SEMs as two dummy variables), ethnicity (0 = No ethnic minority, 1 = ethnic minority), and socio-economic status (NI: 1 = NIMDM2017 ≤ 296.6, 2 = 296.6 < NIMDM2017 ≤ 593.2, 3 = NIMDM2017 > 593.2; Bogotá: 1 = Informal settlement/Lowest/Low, 2 = Middle-Low/Middle, 3 = Middle-High/High; entered into SEMs as two dummy variables). The outcome variable in each model is shown in the row above where the results for each model are presented. See Supplementary Table S3 for details of variables. Bold and underlined text indicates tests which were significant at the *p* < 0.05 level. Structural equation models were specified using the maximum likelihood (ML) estimator with bias-corrected bootstrap standard errors (10,000 repetitions) and imputation of missing data by full information maximum likelihood (FIML). Structural equation models with smoking susceptibility as the outcome variable were specified using the diagonally weighted least squares (DWLS) estimator with bias-corrected bootstrap standard errors (10,000 repetitions). Models were run including the longitudinal measurement invariance constraints on the factor loadings, intercepts, and residual variances for latent variables (as outlined in Supplementary Tables S7, S8). Unstandardized parameter values are reported in Table 7 (std.lv, which standardizes all latent variables in the model only). Standardized parameter values are reported in Supplementary Table S23 (std.all, which standardizes all latent and observed variables in the model).

c Proportion of the total effect of intervention group on the outcome (unadjusted for the mediator) that is mediated via the mediator. In each single or multiple mediator model, the total effect (denominator) is equal to the sum of the direct effect (*c’*) and the indirect effect(s) (Σ*ab*).

d Power to detect the indirect effects calculated using Monte Carlo based statistical power analysis for mediation models with the “power.boot” function in the “bmem” package in R (119, 120).

e Model fit statistics were estimated using the maximum likelihood (ML) estimator. Robust variants of model fit statistics were calculated by specifying “test = ‘yuan.bentler’” in the call of the ‘sem’ function. This calculates robust model fit statistics which are equivalent to those calculated by the robust maximum likelihood (MLR) estimator. For models with smoking susceptibility as the outcome variable, model fit statistics were estimated using the diagonally weighted least squares (DWLS) estimator. Robust variants of model fit statistics were calculated by specifying “test = ‘scaled.shifted’” in the call of the “sem” function. This calculates robust model fit statistics which are equivalent to those calculated by the weighted least square mean and variance adjusted (WLSMV) estimator.

f (1) standard test statistic; (2) robust test statistic. For single mediator models with observed outcome and mediator variables (O), the results were the same for the standard and robust tests (this applies for the model with objectively measured smoking behavior as the outcome variable).

g R-squared: the percentage of variation in the outcome variable explained by the model.

h For the model with objectively measured smoking behavior as the outcome variable, which is a single mediator model with observed outcome and mediator variables (O), the results were the same for the standard and robust tests.

i Retained statistical significance at the 5% level after using the Holm-Bonferroni procedure to correct the *p*-values for multiple testing (*p* < 0.05; based on the number of tests of each type of effect in Table 7, i.e., 37 tests for intervention group effects on mediators, 37 tests for association of mediators with outcomes, 14 tests for direct effects). The procedure is not applied for the indirect effects.

Model 1 included experimental donations to ASSIST/Dead Cool as the outcome variable. Significant indirect effects were observed for perceived social risks (*ab* = −0.03, 95% CI: −0.09 to −0.001) and exposure to advertising in the media (*ab* = 0.10, 95% CI: 0.01, 0.20). Exposure to advertising in the media met all the criteria for mediation (significant *a*-, *b*-, and *ab*-paths), with 33.35% of the total effect mediated. That is, experimental donations to ASSIST/Dead Cool were higher at follow-up for ASSIST participants compared to Dead Cool due to lower exposure to advertising in the media (controlling for baseline). The total effect of intervention on donations was non-significant (Table 3), suggesting there may be other mechanisms (e.g., perceived social risks, or other unmeasured mediators) acting in the opposite direction to increase donations in Dead Cool schools.

Models 2 to 4 included self-report smoking behavior as the outcome variable. In model 2, significant indirect effects were observed for intentions (*ab* = −0.01, 95% CI: −0.03, −0.004) and attitudes (*ab* = −0.02, 95% CI: −0.04 -0.01). In models 3 and 4, there were significant indirect effects for emotional self-efficacy (*ab* = −0.02, 95% CI: −0.04, −0.001) and opportunity self-efficacy (*ab* = −0.02, 95% CI:-0.04, −0.01), respectively. Intentions, attitudes, and opportunity self-efficacy met all the criteria for mediation with proportions mediated of −36.75, −42.11%, and −64.24%, respectively. Dead Cool participants showed more anti-smoking behavior at follow-up compared to ASSIST due to their more anti-smoking intentions, attitudes, and higher opportunity self-efficacy (controlling for baseline). However, the negative proportions mediated indicate suppressor effects, with mediators acting in the opposite direction to the total effect^2^. That is, although the total effect of intervention on self-report smoking behavior was non-significant (Table 3), there would have been more self-report pro-smoking behavior in Dead Cool schools compared to ASSIST at follow-up without the mediating effects of intentions, attitudes, and opportunity self-efficacy.

Model 5 included objectively measured smoking behavior as the outcome variable. There were no significant indirect effects or mediators.

Models 6 and 7 included self-report smoking intentions as the outcome variable. Significant indirect effects were observed for attitudes (*ab* = −0.01, 95% CI: −0.04, −0.001) and opportunity self-efficacy (*ab* = −0.03, 95% CI: −0.07 to −0.01) in models 6 and 7, respectively. Attitudes and opportunity self-efficacy met all the criteria for mediation with proportions mediated of 8.54 and 15.83%, respectively. Dead Cool schools had more anti-smoking intentions at follow-up compared to ASSIST due to their more anti-smoking attitudes and higher opportunity self-efficacy (controlling for baseline).

Models 8 to 11 included smoking susceptibility as the outcome variable. In model 8, significant indirect effects were observed for attitudes (*ab* = 0.08, 95% CI: 0.02, 0.17) and experimentally measured injunctive smoking/vaping norms (*ab* = 0.03, 95% CI: 0.003, 0.07). In model 9, significant indirect effects were observed for self-report injunctive smoking norms (*ab* = 0.07, 95% CI: 0.03, 0.54) and perceived social risks (*ab* = 0.04, 95% CI: 0.00003, 0.11). In model 10, significant indirect effects were observed for knowledge (*ab* = 0.02, 95% CI: 0.002, 0.06), emotional self-efficacy (*ab* = 0.09, 95% CI: 0.02, 0.18), and perceived physical risks (*ab* = 0.04, 95% CI: 0.01, 0.08). In model 11, there was a significant indirect effect for opportunity self-efficacy (*ab* = 0.07, 95% CI: 0.01, 0.14). Attitudes, experimentally measured injunctive smoking/vaping norms, knowledge, emotional and opportunity self-efficacy, and perceived physical risks met all the criteria for mediation with proportions mediated of 39.65, 12.25, 10.47, 36.70, 30.74, and 16.56%, respectively. Dead Cool schools had lower smoking susceptibility at follow-up compared to ASSIST due to their more anti-smoking attitudes and experimentally measured injunctive smoking/vaping norms, and higher knowledge, self-efficacy, and perceived physical risks (controlling for baseline).

Model 12 included knowledge about smoking as the outcome variable. Significant indirect effects were observed for attitudes (*ab* = −0.03, 95% CI: −0.08, −0.01), perceived physical risks (*ab* = −0.04, 95% CI: −0.14, −0.01), and perceived social risks (*ab* = −0.04, 95% CI: −0.19, −0.002). Attitudes met all the criteria for mediation with a proportion mediated of 7.48%. Dead Cool schools had higher knowledge about smoking at follow-up compared to ASSIST due to their more anti-smoking attitudes (controlling for baseline).

Models 13 and 14 included attitudes towards smoking as the outcome variable. In model 13, significant indirect effects were observed for knowledge (*ab* = −0.03, 95% CI: −0.05, −0.01), self-report descriptive smoking norms scale 2 (*ab* = −0.02, 95% CI: −0.06, −0.003), and PBC to avoid smoking (*ab* = −0.01, 95% CI: −0.03, −0.001). In model 14, significant indirect effects were observed for perceived physical risks (*ab* = −0.03, 95% CI: −0.06, −0.01) and exposure to advertising in shops (*ab* = −0.01, 95% CI: −0.03, −0.001). Knowledge, self-report descriptive smoking norms (scale 2), perceived physical risks, and exposure to advertising in shops met all the criteria for mediation with proportions mediated of 12.64, 10.76, 27.40, and 11.69%, respectively. Dead Cool schools had more anti-smoking attitudes at follow-up compared to ASSIST due to their more anti-smoking values of self-report descriptive smoking norms (scale 2), and higher knowledge, perceived physical risks, and exposure to advertising in shops (controlling for baseline).

The results of single mediator models with intervention group as the predictor variable are reported in Supplementary Tables S24–S37.

#### 3.2.2 Mediator models with predictor variables comparing ASSIST non-peer supporters and ASSIST peer supporters separately with Dead Cool

The results of the final single and multiple mediator models with predictor variables comparing ASSIST non-PS and ASSIST PS separately with Dead Cool are reported in Table 8 (unstandardized parameter estimates) and Supplementary Table S38 (standardized parameter estimates). R^2^ ranged between 0.129 and 0.566. Power to detect the indirect effects ranged between 0.005 and 1.000. The following paragraphs highlight where these results diverge from the mediator models with predictor variables comparing all ASSIST participants with Dead Cool.

**Table 8:** Results of final single and multiple mediator models with predictor variables comparing ASSIST non-peer supporters and ASSIST peer supporters separately with Dead Cool, including data from MECHANISMS schools.

Mediator^a^	Intervention group (versus Dead Cool)^a^^,^^b^	n	Intervention group effect on mediator^c^			Association of mediator with outcome^c^			Direct effect^c^			Indirect effect^c^			Proportion mediated (%)^d^	Power (SE)^e^	Model fit statistics^a^^,^^f^						
			a	SE	*p*-value	b	SE	*p*-value	c’	SE	*p*-value	ab	SE	95% CI	[ab/(c’ + Σab)] × 100		χ^2^, *p*-value^g^	df	CFI^g^	TLI^g^	RMSEA (90% CI)^g^	SRMR	R^2,^^h^
Model 1: experimental donations to ASSIST/Dead Cool (O) as the outcome variable.																							
Self-report smoking behavior (O)	ASSIST non-PS	1,136	0.04	0.04	0.2875	**0.28**	**0.14**	**0.0429**	0.26	0.18	0.1392	0.01	0.01	−0.01, 0.05	1.74	0.250 (0.014)	(1) 2755.76, *p* < 0.0001	1,008	(1) 0.864	(1) 0.850	(1) 0.039 (0.037, 0.041)	0.062	0.129
	ASSIST PS		**0.12**	**0.05**	**0.0258**				0.23	0.25	0.3710	**0.03**	**0.02**	**0.002, 0.10**	**5.59**	0.979 (0.005)							
Attitudes (L)	ASSIST non-PS		−0.12	0.07	0.1040	0.06	0.10	0.5628	N/A.			−0.01	0.02	−0.05, 0.01	−1.22	0.440 (0.016)							
	ASSIST PS		−0.06	0.11	0.6039							−0.004	0.02	−0.06, 0.01	−0.60	0.223 (0.013)							
Self-report descriptive smoking norms 2 (L)	ASSIST non-PS		**−0.18**	**0.09**	**0.0500**	0.11	0.11	0.2877				−0.02	0.02	−0.09, 0.01	−3.36	0.861 (0.011)							
	ASSIST PS		−0.23	0.14	0.0997							−0.03	0.03	−0.13, 0.01	−4.29	0.859 (0.011)	(2) 2151.91, *p* < 0.0001		(2) 0.875	(2) 0.862	(2) 0.038 (0.036, 0.040)		
Perceived social risks (L)	ASSIST non-PS		**−0.25**	**0.08**	**0.0013** ^**j**^	0.19	0.11	0.0719				**−0.05**	**0.03**	**−0.12, −0.001**	**−7.88**	0.999 (0.001)							
	ASSIST PS		−0.04	0.12	0.7264							−0.01	0.03	−0.08, 0.03	−1.32	0.250 (0.014)							
Exposure to advertising in the media (O)	ASSIST non-PS		**−1.22**	**0.13**	**<0.0001** ^**j**^	**−0.08**	**0.04**	**0.0470**				**0.10**	**0.05**	**0.003, 0.21**	**16.25**	0.739 (0.014)							
	ASSIST PS		**−1.01**	**0.20**	**<0.0001** ^**j**^							**0.08**	**0.05**	**0.01, 0.19**	**13.50**	0.740 (0.014)							
Model 2: self-report smoking behavior (O) as the outcome variable																							
Intentions (O)	ASSIST non-PS	1,186	**−0.18**	**0.08**	**0.0154**	**0.06**	**0.02**	**0.0046**	0.06	0.03	0.0669	**−0.01**	**0.01**	**−0.03, −0.002**	**−8.92**	0.496 (0.016)	(1) 5920.01, *p* < 0.0001	2,170	(1) 0.907	(1) 0.902	(1) 0.038 (0.037, 0.039)	0.088	0.487
	ASSIST PS		−0.11	0.11	0.2855				**0.11**	**0.05**	**0.0315**	−0.01	0.01	−0.03, 0.003	−5.57	0.517 (0.016)							
Attitudes (L)	ASSIST non-PS		**−0.18**	**0.07**	**0.0135**	**0.06**	**0.02**	**0.0177**	N/A.			**−0.01**	**0.01**	**−0.03, −0.002**	**−8.87**	0.435 (0.016)							
	ASSIST PS		−0.12	0.12	0.2937							−0.01	0.01	−0.03, 0.004	−5.83	0.453 (0.016)							
Self-report injunctive smoking norms (L)	ASSIST non-PS		−0.13	0.08	0.1139	−0.002	0.03	0.9563				0.0002	0.00	−0.01, 0.01	0.17	0.061 (0.008)							
	ASSIST PS		−0.01	0.14	0.9215							2.12E-05	0.00	−0.01, 0.01	0.02	0.005 (0.002)	(2) 3271.30, *p* < 0.0001		(2) 0.925	(2) 0.921	(2) 0.035 (0.033, 0.036)		
Self-efficacy: Emotional (L)	ASSIST non-PS		−0.11	0.07	0.0950	**0.12**	**0.03**	**<0.0001** ^**j**^				−0.01	0.01	−0.03, 0.001	−11.17	0.970 (0.005)							
	ASSIST PS		−0.04	0.09	0.7083							−0.004	0.01	−0.03, 0.02	−3.54	0.258 (0.014)							
PBC (to avoid smoking; O)	ASSIST non-PS		**−0.17**	**0.08**	**0.0282**	0.01	0.02	0.4442				−0.002	0.00	−0.01, 0.002	−1.67	0.091 (0.009)							
	ASSIST PS		0.06	0.11	0.5678							0.001	0.00	−0.002, 0.01	0.63	0.068 (0.008)							
Model 3: self-report smoking behavior (O) as the outcome variable																							
Self-efficacy: Opportunity (L)	ASSIST non-PS	1,190	**−0.18**	**0.07**	**0.0103**	**0.10**	**0.03**	**0.0001** ^**j**^	0.03	0.03	0.3879	**−0.02**	**0.01**	**−0.04, −0.01**	**−18.01**	0.940 (0.008)	(1) 2656.81, *p* < 0.0001	435	(1) 0.937	(1) 0.930	(1) 0.066 (0.063, 0.068)	0.086	0.471
	ASSIST PS		−0.13	0.11	0.2359				**0.10**	**0.05**	**0.0494**	−0.01	0.01	−0.04, 0.01	−13.39	0.946 (0.007)	(2) 507.13, *p* = 0.0095		(2) 0.964	(2) 0.960	(2) 0.050 (0.045, 0.055)		
Model 4: objectively measured smoking behavior (O) as the outcome variable^i^																							
Intentions (O)	ASSIST non-PS	1,131	**−0.23**	**0.08**	**0.0050**	−0.08	0.07	0.2665	−0.10	0.09	0.2742	0.02	0.02	−0.01, 0.08	−23.98	0.801 (0.013)	0.00, *p* = 1.00	0	1.000	1.000	0.000 (0.000, 0.000)	0.000	0.251
	ASSIST PS		−0.12	0.11	0.3075				−0.002	0.15	0.9898	0.01	0.01	−0.004, 0.07	−12.18	0.822 (0.012)							
Model 5: smoking intentions (O) as the outcome variable																							
Knowledge (O)	ASSIST non-PS	1,186	**−0.55**	**0.09**	**<0.0001** ^**j**^	0.03	0.02	0.1729	−0.10	0.07	0.1866	−0.02	0.01	−0.05, 0.01	6.25	0.203 (0.013)	(1) 6062.17, *p* < 0.0001	2,460	(1) 0.913	(1) 0.909	(1) 0.035 (0.034, 0.036)	0.071	0.221
	ASSIST PS		**0.34**	**0.14**	**0.0159**				−0.09	0.10	0.3713	0.01	0.01	−0.002, 0.04	−3.92	0.206 (0.013)							
Attitudes (L)	ASSIST non-PS		**−0.16**	**0.08**	**0.0365**	**0.10**	**0.05**	**0.0476**	N/A.			**−0.02**	**0.01**	**−0.05, −0.001**	**5.58**	0.856 (0.011)							
	ASSIST PS		−0.08	0.12	0.5083							−0.01	0.01	−0.05, 0.01	2.66	0.621 (0.015)							
Self-report injunctive smoking norms (L)	ASSIST non-PS		−0.11	0.08	0.2044	**0.13**	**0.05**	**0.0131**				−0.01	0.01	−0.05, 0.004	5.05	0.831 (0.012)							
	ASSIST PS		0.03	0.14	0.8203							0.004	0.02	−0.03, 0.05	−1.53	0.193 (0.012)	(2) 3364.71, *p* < 0.0001		(2) 0.933	(2) 0.930	(2) 0.032 (0.030, 0.033)		
Self-report descriptive smoking norms 2 (L)	ASSIST non-PS		−0.14	0.09	0.0913	0.10	0.05	0.0570				−0.01	0.01	−0.05, 0.001	5.25	0.780 (0.013)							
	ASSIST PS		−0.21	0.14	0.1234							−0.02	0.02	−0.08, 0.003	7.54	0.782 (0.013)							
Self-efficacy: Emotional (L)	ASSIST non-PS		−0.10	0.07	0.1406	**0.20**	**0.05**	**0.0001** ^**j**^				−0.02	0.01	−0.06, 0.003	7.12	0.918 (0.009)							
	ASSIST PS		0.001	0.10	0.9942							0.0001	0.02	−0.04, 0.04	−0.05	0.066 (0.008)							
Model 6: smoking intentions (O) as the outcome variable																							
Self-efficacy: Opportunity (L)	ASSIST non-PS	1,187	**−0.17**	**0.07**	**0.0086**	**0.21**	**0.05**	**<0.0001** ^**j**^	**−0.19**	**0.07**	**0.0110**	**−0.04**	**0.02**	**−0.07, −0.01**	**9.46**	1.000 (0.000)	(1) 2523.99, *p* < 0.0001	435	(1) 0.939	(1) 0.932	(1) 0.064 (0.061, 0.066)	0.070	0.147
	ASSIST PS		−0.11	0.11	0.3188				−0.14	0.11	0.2076	−0.02	0.03	−0.09, 0.01	5.93	0.963 (0.006)	(2) 495.37, *p* = 0.0238		(2) 0.965	(2) 0.962	(2) 0.048 (0.043, 0.054)		
Model 7: smoking susceptibility (O) as the outcome variable																							
Attitudes (L)	ASSIST non-PS	1,002	**−0.21**	**0.09**	**0.0180**	**−0.42**	**0.05**	**<0.0001** ^**j**^	0.06	0.10	0.4995	**0.09**	**0.04**	**0.01, 0.17**	**17.39**	1.000 (0.000)	(1) 3177.69, *p* < 0.0001	1,158	(1) 0.927	(1) 0.951	(1) 0.042 (0.040, 0.043)	0.042	0.543
	ASSIST PS		−0.21	0.14	0.1318				0.20	0.13	0.1465	0.09	0.06	−0.02, 0.22	17.98	1.000 (0.000)							
Experiment injunctive smoking/vaping norms (L)	ASSIST non-PS		**0.20**	**0.09**	**0.0334**	**0.14**	**0.06**	**0.0126**	N/A.			**0.03**	**0.02**	**0.004, 0.08**	**5.96**	0.968 (0.006)							
	ASSIST PS		0.12	0.14	0.3840							0.02	0.02	−0.02, 0.08	3.53	0.981 (0.010)	(2) 2514.77, *p* < 0.0001		(2) 0.842	(2) 0.894	(2) 0.034 (0.032, 0.036)		
PBC (to avoid smoking; O)	ASSIST non-PS		**−0.21**	**0.08**	**0.0110**	−0.07	0.04	0.0813				**0.01**	**0.01**	**0.00001, 0.04**	**2.81**	0.563 (0.016)							
	ASSIST PS		0.06	0.11	0.6300							−0.004	0.01	−0.03, 0.01	−0.74	0.300 (0.014)							
Model 8: smoking susceptibility (O) as the outcome variable																							
Self-report injunctive smoking norms (L)	ASSIST non-PS	1,067	**−0.26**	**0.10**	**0.0105**	−0.18	0.18	0.3148	0.06	0.24	0.8036	0.05	0.06	−0.04, 0.14	9.83	0.975 (0.005)	(1) 3978.79, *p* < 0.0001	952	(1) 0.848	(1) 0.894	(1) 0.055 (0.053, 0.056)	0.055	0.505
	ASSIST PS		−0.14	0.16	0.3799				0.16	0.32	0.6184	0.03	0.06	−0.04, 0.15	5.34	0.948 (0.007)							
Self-report descriptive smoking norms 1 (L)	ASSIST non-PS		**−0.35**	**0.12**	**0.0048**	−0.14	0.37	0.7153	N/A.			0.05	0.15	−0.08, 0.57	9.87	0.694 (0.015)							
	ASSIST PS		**−0.43**	**0.20**	**0.0350**							0.06	0.19	−0.13, 0.66	12.20	0.686 (0.015)							
Self-report descriptive smoking norms 2 (L)	ASSIST non-PS		**−0.32**	**0.12**	**0.0075**	−0.04	0.28	0.8842				0.01	0.10	−0.12, 0.23	2.66	0.088 (0.009)	(2) 2965.46, *p* < 0.0001		(2) 0.733	(2) 0.813	(2) 0.045 (0.043, 0.046)		
	ASSIST PS		**−0.45**	**0.20**	**0.0296**							0.02	0.14	−0.15, 0.36	3.71	0.082 (0.009)							
Perceived social risks (L)	ASSIST non-PS		**−0.36**	**0.09**	**0.0001** ^**j**^	−0.12	0.09	0.1838				0.04	0.03	−0.001, 0.12	9.06	0.858 (0.011)							
	ASSIST PS		−0.09	0.14	0.5347							0.01	0.02	−0.02, 0.08	2.22	0.677 (0.015)							
Model 9: smoking susceptibility (O) as the outcome variable																							
Knowledge (O)	ASSIST non-PS	1,067	**−0.55**	**0.09**	**<0.0001** ^**j**^	**−0.07**	**0.03**	**0.0276**	0.03	0.09	0.7129	**0.04**	**0.02**	**0.01, 0.08**	**7.54**	0.654 (0.015)	(1) 2814.00, *p* < 0.0001	869	(1) 0.969	(1) 0.980	(1) 0.046 (0.044, 0.048)	0.038	0.566
	ASSIST PS		**0.28**	**0.14**	**0.0439**				0.27	0.15	0.0633	**−0.02**	**0.01**	**−0.06, −0.001**	**−3.88**	0.661 (0.015)							
Self-efficacy: Emotional (L)	ASSIST non-PS		**−0.20**	**0.08**	**0.0169**	**−0.47**	**0.07**	**<0.0001** ^**j**^	N/A.			**0.09**	**0.04**	**0.03, 0.19**	**17.90**	1.000 (0.000)							
	ASSIST PS		−0.13	0.11	0.2397							0.06	0.05	−0.03, 0.19	12.23	0.987 (0.004)	(2) 2280.15, *p* < 0.0001		(2) 0.886	(2) 0.926	(2) 0.039 (0.037, 0.041)		
Perceived physical risks (L)	ASSIST non-PS		**−0.35**	**0.08**	**<0.0001** ^**j**^	**−0.16**	**0.04**	**0.0003** ^**j**^				**0.06**	**0.02**	**0.02, 0.11**	**10.69**	0.996 (0.002)							
	ASSIST PS		0.12	0.13	0.3371							−0.02	0.02	−0.07, 0.02	−3.81	0.968 (0.006)							
Model 10: smoking susceptibility (O) as the outcome variable																							
Self-efficacy: Opportunity (L)	ASSIST non-PS	1,083	**−0.18**	**0.07**	**0.0142**	**−0.42**	**0.05**	**<0.0001** ^**j**^	0.14	0.09	0.1276	**0.08**	**0.03**	**0.01, 0.14**	**15.28**	0.571 (0.016)	(1) 2272.28, *p* < 0.0001	435	(1) 0.984	(1) 0.991	(1) 0.062 (0.060, 0.065)	0.048	0.490
	ASSIST PS		−0.13	0.12	0.2657				0.22	0.14	0.1232	0.05	0.05	−0.03, 0.16	11.04	0.584 (0.016)	(2) 1752.89, *p* < 0.0001		(2) 0.916	(2) 0.951	(2) 0.053 (0.050, 0.056)		
Model 11: knowledge of smoking (O) as the outcome variable																							
Attitudes (L)	ASSIST non-PS	1,187	**−0.20**	**0.07**	**0.0070**	**0.15**	**0.07**	**0.0221**	**−0.41**	**0.12**	**0.0008** ^**j**^	**−0.03**	**0.02**	**−0.08, −0.01**	**13.35**	0.960 (0.006)	(1) 4409.80, *p* < 0.0001	1,345	(1) 0.851	(1) 0.842	(1) 0.044 (0.042, 0.045)	0.072	0.343
	ASSIST PS		−0.13	0.11	0.2479				**0.34**	**0.13**	**0.0090**	−0.02	0.02	−0.09, 0.01	8.86	0.926 (0.008)							
Perceived physical risks (L)	ASSIST non-PS		**−0.30**	**0.07**	**<0.0001** ^**j**^	0.16	0.11	0.1317	N/A.			**−0.05**	**0.04**	**−0.16, −0.01**	**21.96**	0.730 (0.014)							
	ASSIST PS		0.10	0.12	0.4004							0.02	0.03	−0.01, 0.11	−7.29	0.411 (0.016)							
Perceived social risks (L)	ASSIST non-PS		**−0.26**	**0.09**	**0.0025**	0.19	0.14	0.1521				**−0.05**	**0.04**	**−0.21, −0.01**	**23.06**	0.696 (0.015)	(2) 3389.98, *p* < 0.0001		(2) 0.857	(2) 0.847	(2) 0.044 (0.042, 0.046)		
	ASSIST PS		−0.11	0.14	0.4494							−0.02	0.04	−0.16, 0.02	9.55	0.303 (0.015)							
PBC (to avoid smoking; O)	ASSIST non-PS		**−0.19**	**0.08**	**0.0172**	0.05	0.05	0.3361				−0.01	0.01	−0.04, 0.005	4.00	0.259 (0.014)							
	ASSIST PS		0.05	0.11	0.6697							0.002	0.01	−0.01, 0.03	−0.99	0.088 (0.009)							
Model 12: attitudes towards smoking (L) as the outcome variable																							
Knowledge (O)	ASSIST non-PS	1,183	**−0.54**	**0.09**	**<0.0001** ^**j**^	**0.06**	**0.03**	**0.0315**	**−0.19**	**0.09**	**0.0281**	**−0.03**	**0.02**	**−0.07, −0.004**	**9.45**	0.153 (0.011)	(1) 4483.72, *p* < 0.0001	1,424	(1) 0.854	(1) 0.843	(1) 0.043 (0.041, 0.044)	0.071	0.234
	ASSIST PS		**0.30**	**0.14**	**0.0331**				−0.21	0.11	0.0596	**0.02**	**0.01**	**0.001, 0.05**	**−5.24**	0.175 (0.012)							
Perceived physical risks (L)	ASSIST non-PS		**−0.32**	**0.07**	**<0.0001** ^**j**^	0.05	0.06	0.3707	N/A.			−0.02	0.02	−0.05, 0.02	4.58	0.103 (0.010)							
	ASSIST PS		0.09	0.12	0.4502							0.005	0.01	−0.01, 0.04	−1.32	0.032 (0.006)							
Perceived social risks (L)	ASSIST non-PS		**−0.27**	**0.09**	**0.0033**	0.04	0.06	0.5253				−0.01	0.02	−0.05, 0.02	3.05	0.077 (0.008)							
	ASSIST PS		−0.11	0.15	0.4650							−0.004	0.01	−0.05, 0.01	1.25	0.020 (0.004)	(2) 3474.18, *p* < 0.0001		(2) 0.859	(2) 0.849	(2) 0.043 (0.041, 0.044)		
PBC (to avoid smoking; O)	ASSIST non-PS		**−0.18**	**0.08**	**0.0229**	**0.11**	**0.03**	**0.0001** ^**j**^				**−0.02**	**0.01**	**−0.04, −0.004**	**5.48**	0.726 (0.014)							
	ASSIST PS		0.05	0.11	0.6511							0.01	0.01	−0.02, 0.03	−1.50	0.246 (0.014)							
Exposure to advertising in the media (O)	ASSIST non-PS		**−1.22**	**0.13**	**<0.0001** ^**j**^	**−0.05**	**0.02**	**0.0323**				**0.05**	**0.03**	**0.01, 0.11**	**−15.71**	0.172 (0.012)							
	ASSIST PS		**−1.00**	**0.20**	**<0.0001** ^**j**^							**0.05**	**0.02**	**0.01, 0.10**	**−12.92**	0.174 (0.012)							

a a = unstandardized regression coefficient for intervention group effect on mediator; b = unstandardized regression coefficient for association of mediator with outcome; c’ = unstandardized regression coefficient for direct effect; ab = unstandardized regression coefficient for indirect effect; SE = standard error; χ^2^ = Chi-square Goodness of Fit statistic; df = degrees of freedom (χ^2^ test); CFI = Comparative Fit Index; TLI = Tucker Lewis Index; RMSEA = Root Mean Square Error of Approximation; CI = confidence interval; SRMR = Standardized Root Mean Square Residual; R^2^ = R-squared; (O) = observed variable; (L) = latent variable; PBC = Perceived behavioral control; ASSIST = ‘A Stop Smoking in Schools Trial’; PS = peer supporters; N/A = not applicable.

b For intervention group effects on mediators, direct effects, indirect effects, and proportions mediated, this column indicates whether the specific row refers to ASSIST non-peer supporters compared to Dead Cool or ASSIST peer supporters compared to Dead Cool.

c Results are unstandardized regression coefficients from single and multiple mediator structural equation models (SEMs). Bias-corrected bootstrap confidence intervals for the indirect effects are reported, along with the standard errors (standard errors and *p*-values are reported for the intervention effects on the mediator, association of the mediator with the outcome, and direct effects). In each model, the predictor variables are group assignment [two dummy variables: (1) 0 = Dead Cool and ASSIST peer supporters, 1 = ASSIST non-peer supporters; (2) 0 = Dead Cool and ASSIST non-peer supporters, 1 = ASSIST peer supporters], the mediating variable is the follow-up score of the mediator, and the dependent variable is the follow-up score of the outcome variable. In addition to baseline values of the outcome and mediator, all models also include the following baseline variables as covariates: setting (0 = Northern Ireland, 1 = Bogotá), gender (0 = boy, 1 = girl/prefer not to say), age (1 = 12 years or less, 2 = 13 years, 3 = 14 years or more; entered into SEMs as two dummy variables), ethnicity (0 = No ethnic minority, 1 = ethnic minority), and socio-economic status (NI: 1 = NIMDM2017 ≤ 296.6, 2 = 296.6 < NIMDM2017 ≤ 593.2, 3 = NIMDM2017 > 593.2; Bogotá: 1 = Informal settlement/Lowest/Low, 2 = Middle-Low/Middle, 3 = Middle-High/High; entered into SEMs as two dummy variables). The outcome variable in each model is shown in the row above where the results for each model are presented. See Supplementary Table S3 for details of variables. Bold and underlined text indicates tests which were significant at the *p* < 0.05 level. Structural equation models were specified using the maximum likelihood (ML) estimator with bias-corrected bootstrap standard errors (10,000 repetitions) and imputation of missing data by full information maximum likelihood (FIML). Structural equation models with smoking susceptibility as the outcome variable were specified using the diagonally weighted least squares (DWLS) estimator with bias-corrected bootstrap standard errors (10,000 repetitions). Models were run including the longitudinal measurement invariance constraints on the factor loadings, intercepts, and residual variances for latent variables (as outlined in Supplementary Tables S7, S8). Unstandardized parameter values are reported in Table 8 (std.lv, which standardizes all latent variables in the model only). Standardized parameter values are reported in Supplementary Table S38 (std.all, which standardizes all latent and observed variables in the model).

d Proportion of the total effect of intervention group on the outcome (unadjusted for the mediator) that is mediated via the mediator. In each single or multiple mediator model, the total effect (denominator) is equal to the sum of the direct effect (*c’*) and the indirect effect(s) (Σ*ab*). Calculated separately for ASSIST non-peer supporters compared to Dead Cool, and ASSIST peer supporters compared to Dead Cool.

e Power to detect the indirect effects calculated using Monte Carlo based statistical power analysis for mediation models with the “power.boot” function in the “bmem” package in R (119, 120).

f Model fit statistics were estimated using the maximum likelihood (ML) estimator. Robust variants of model fit statistics were calculated by specifying “test = ‘yuan.bentler’” in the call of the “sem” function. This calculates robust model fit statistics which are equivalent to those calculated by the robust maximum likelihood (MLR) estimator. For models with smoking susceptibility as the outcome variable, model fit statistics were estimated using the diagonally weighted least squares (DWLS) estimator. Robust variants of model fit statistics were calculated by specifying “test = ‘scaled.shifted’” in the call of the ‘sem’ function. This calculates robust model fit statistics which are equivalent to those calculated by the weighted least square mean and variance adjusted (WLSMV) estimator.

g (1) standard test statistic; (2) robust test statistic. For single mediator models with observed outcome and mediator variables (O), the results were the same for the standard and robust tests (this applies for the model with objectively measured smoking behavior as the outcome variable).

h R-squared: the percentage of variation in the outcome variable explained by the model.

i For the model with objectively measured smoking behavior as the outcome variable, which is a single mediator model with observed outcome and mediator variables (O), the results were the same for the standard and robust tests.

j Retained statistical significance at the 5% level after using the Holm-Bonferroni procedure to correct the *p*-values for multiple testing [*p* < 0.05; based on the number of tests of each type of effect in Table 8, i.e., 38 tests for intervention group effects on mediators (ASSIST non-peer supporters), 38 tests for intervention group effects on mediators (ASSIST peer supporters), 38 tests for association of mediators with outcomes, 12 tests for direct effects of intervention group (ASSIST non-peer supporters), 12 tests for direct effects of intervention group (ASSIST peer supporters)]. The procedure is not applied for the indirect effects.

Model 1 included experimental donations to ASSIST/Dead Cool as the outcome variable. The significant indirect effect for perceived social risks was observed for the ASSIST non-PS group only. The significant indirect effect for exposure to advertising in the media was observed in both the ASSIST non-PS and ASSIST PS groups, meeting all the criteria for mediation in both groups (significant *a*-, *b*-, and *ab*-paths). A new significant indirect effect emerged for self-report smoking behavior in the ASSIST PS group (*ab* = 0.03, 95% CI: 0.002, 0.10), which also met all mediation criteria with 5.59% of the total effect mediated. Experimental donations to ASSIST/Dead Cool were higher at follow-up for the ASSIST PS compared to Dead Cool due to their more anti-smoking behavior and lower exposure to advertising in the media (controlling for baseline).

Models 2 and 3 included self-report smoking behavior as the outcome variable. The significant indirect effects for intentions, attitudes, and opportunity self-efficacy were observed for the ASSIST non-PS group only and met all the criteria for mediation. There were no significant indirect effects for emotional self-efficacy.

Model 4 included objectively measured smoking behavior as the outcome variable. There were no significant indirect effects or mediators.

Models 5 and 6 included self-report smoking intentions as the outcome variable. The significant indirect effects for attitudes and opportunity self-efficacy were observed for the ASSIST non-PS group only and met all the criteria for mediation.

Models 7 to 10 included smoking susceptibility as the outcome variable. Significant indirect effects for attitudes, experimentally measured injunctive smoking/vaping norms, emotional and opportunity self-efficacy, and perceived physical risks were observed for the ASSIST non-PS group only, each meeting all the criteria for mediation. Self-report injunctive smoking norms and perceived social risks were not significant mediators for either the ASSIST non-PS or ASSIST PS groups. A new significant indirect effect was identified for PBC to avoid smoking in the ASSIST non-PS group (*ab* = 0.01, 95% CI: 0.00001, 0.04). There were significant indirect effects for knowledge which were in the opposite directions for the ASSIST non-PS (*ab* = 0.04, 95% CI: 0.01, 0.08) and ASSIST PS groups (*ab* = −0.02, 95% CI: −0.06, −0.001), both meeting all the mediation criteria with proportions mediated of 7.54% and −3.88%, respectively. Dead Cool participants had lower smoking susceptibility compared to ASSIST non-PS at follow-up due to their higher knowledge of smoking (controlling for baseline), whilst the opposite pattern was observed for the ASSIST PS. However, the negative proportion mediated for ASSIST PS indicates suppression, suggesting that knowledge acted in the opposite direction to the total effect. Although the total intervention effect on smoking susceptibility was non-significant for the ASSIST PS group (Table 4), their smoking susceptibility would have been higher without this suppressive mediating pathway.

Model 11 included knowledge about smoking as the outcome variable. Significant indirect effects for attitudes, perceived physical risks, and perceived social risks were observed for the ASSIST non-PS group only. Attitudes met all the criteria for mediation.

Model 12 included attitudes towards smoking as the outcome variable. A significant indirect effect for PBC to avoid smoking was observed for the ASSIST non-PS group only, which met all the criteria for mediation. Self-report descriptive smoking norms (scale 2) and exposure to advertising in shops were not selected by the CMF method or single mediator models. No significant indirect effects were found for perceived physical risks in either the ASSIST non-PS or ASSIST PS groups. As in the smoking susceptibility model, knowledge had significant indirect effects in opposite directions for the ASSIST non-PS (*ab* = −0.03, 95% CI: −0.07, −0.004) and ASSIST PS groups (*ab* = 0.02, 95% CI: 0.001, 0.05), each meeting all the mediation criteria with proportions mediated of 9.45% and −5.24%, respectively. The negative proportion mediated for ASSIST PS again indicated a suppressive effect. New significant indirect effects were identified for exposure to advertising in the media in both the ASSIST non-PS (*ab* = 0.05, 95% CI: 0.01, 0.11) and ASSIST PS groups (*ab* = 0.05, 95% CI: 0.01, 0.10), both also meeting all the mediation criteria with proportions mediated of −15.71% and −12.92%, respectively. Both ASSIST groups showed stronger anti-smoking attitudes at follow-up than Dead Cool participants due to their lower exposure to media advertising (controlling for baseline). The negative proportions mediated again indicate suppressive mediation effects acting counter to the total effect.

The results of single mediator models with predictor variables comparing ASSIST non-PS and ASSIST PS separately with Dead Cool are reported in Supplementary Tables S39–S52.

#### 3.2.3 Mediator models with setting as the predictor variable

The results of the final single and multiple mediator models with setting (Bogotá versus NI) as the predictor variable are reported in Table 9 (unstandardized parameter estimates) and Supplementary Table S53 (standardized parameter estimates). R^2^ ranged between 0.118 and 0.571. Power to detect the indirect effects ranged between 0.056 and 1.000.

**Table 9:** Results of final single and multiple mediator models with setting (Bogotá versus Northern Ireland) as the predictor variable, including data from MECHANISMS schools.

Mediator^a^	*n*	Setting effect on mediator^b^			Association of mediator with outcome^b^			Direct effect^b^			Indirect effect^b^			Proportion mediated (%)^c^	Power (SE)^d^	Model fit statistics^a^^,^^e^						
		a	SE	*p*-value	b	SE	*p*-value	c’	SE	*p*-value	ab	SE	95% CI	[ab/(c’ + Σab)] × 100		χ^2^, *p*-value^f^	df	CFI^f^	TLI^f^	RMSEA (90% CI)^f^	SRMR	R^2,^^g^
Model 1: experimental donations to ASSIST/Dead Cool (O) as the outcome variable																						
Self-efficacy: friends (L)	1,136	−0.09	0.07	0.2167	**0.15**	**0.08**	**0.0431**	**0.62**	**0.18**	**0.0005** ^**i**^	−0.01	0.01	−0.05, 0.004	−2.64	0.840 (0.012)	(1) 1673.95, *p* < 0.0001	528	(1) 0.953	(1) 0.948	(1) 0.044 (0.041, 0.046)	0.058	0.129
Perceived social risks (L)		**−0.22**	**0.08**	**0.0040**	0.19	0.11	0.0788				−0.04	0.03	−0.12, 0.0003	−8.00	0.999 (0.001)							
																(2) 842.17, *p* < 0.0001		(2) 0.965	(2) 0.962	(2) 0.038 (0.035, 0.042)		
Exposure to advertising in the media (O)		**0.62**	**0.15**	**<0.0001** ^**i**^	**−0.08**	**0.04**	**0.0409**				**−0.05**	**0.03**	**−0.12, −0.01**	**−10.05**	0.800 (0.013)							
Model 2: experimental donations to ASSIST/Dead Cool (O) as the outcome variable																						
Self-efficacy: opportunity (L)	1,141	**−0.16**	**0.07**	**0.0240**	**0.17**	**0.07**	**0.0159**	**0.51**	**0.17**	**0.0028** ^**i**^	**−0.03**	**0.02**	**−0.07, −0.004**	**−5.70**	1.000 (0.000)	(1) 2299.54, *p* < 0.0001	414	(1) 0.942	(1) 0.935	(1) 0.063 (0.061, 0.066)	0.046	0.118
																(2) 454.51, *p* = 0.0827		(2) 0.968	(2) 0.965	(2) 0.047 (0.041, 0.053)		
Model 3: self-report smoking behavior (O) as the outcome variable																						
Intentions (O)	1,175	−0.12	0.08	0.1281	**0.10**	**0.02**	**<0.0001** ^**i**^	0.01	0.04	0.8281	−0.01	0.01	−0.03, 0.002	−65.53	0.938 (0.008)	(1) 1.59, *p* = 0.6615	3	(1) 1.000	(1) 1.018	(1) 0.000 (0.000, 0.038)	0.003	0.507
PBC (easy to quit; O)		**0.74**	**0.10**	**<0.0001** ^**i**^	**0.03**	**0.01**	**0.0233**				**0.02**	**0.01**	**0.003, 0.04**	**103.48**	0.147 (0.011)							
																(2) 1.46, *p* = 0.6913		(2) 1.000	(2) 1.021	(2) 0.000 (0.000, 0.040)		
Exposure to advertising in the media (O)		**0.61**	**0.15**	**0.0001** ^**i**^	0.01	0.01	0.5838				0.003	0.01	−0.01, 0.02	17.61	0.056 (0.007)							
Model 4: self-report smoking behavior (O) as the outcome variable																						
Self-efficacy: emotional (L)	1,188	**−0.30**	**0.07**	**<0.0001** ^**i**^	**0.08**	**0.04**	**0.0338**	0.02	0.04	0.5726	**−0.02**	**0.01**	**−0.06, −0.005**	**91.44**	0.784 (0.013)	(1) 5398.57, *p* < 0.0001	1,021	(1) 0.916	(1) 0.911	(1) 0.060 (0.059, 0.062)	0.162	0.470
Self-efficacy: friends (L)		**−0.20**	**0.07**	**0.0038**	**0.09**	**0.04**	**0.0153**				**−0.02**	**0.01**	**−0.05, −0.004**	**70.35**	0.873 (0.011)							
																(2) 1615.35, *p* < 0.0001		(2) 0.939	(2) 0.936	(2) 0.052 (0.049, 0.055)		
PBC (to avoid smoking; O)		**−0.32**	**0.08**	**<0.0001** ^**i**^	0.01	0.02	0.3364				−0.005	0.00	−0.02, 0.004	17.49	0.082 (0.009)							
Model 5: self-report smoking behavior (O) as the outcome variable																						
Self-efficacy: opportunity (L)	1,190	**−0.25**	**0.07**	**0.0006** ^**i**^	**0.10**	**0.03**	**0.0001** ^**i**^	0.005	0.03	0.8953	**−0.03**	**0.01**	**−0.05, −0.01**	**122.13**	0.940 (0.008)	(1) 2639.61, *p* < 0.0001	414	(1) 0.937	(1) 0.929	(1) 0.067 (0.065, 0.070)	0.089	0.470
																(2) 484.73, *p* = 0.0093		(2) 0.964	(2) 0.960	(2) 0.051 (0.046, 0.057)		
Model 6: objectively measured smoking behavior (O) as the outcome variable^h^																						
Intentions (O)	1,131	−0.08	0.09	0.3614	−0.08	0.07	0.2712	**1.18**	**0.11**	**<0.0001** ^**i**^	0.01	0.01	−0.005, 0.05	0.54	0.647 (0.015)	0.00, *p* = 1.00	0	1.000	1.000	0.000 (0.000, 0.000)	0.000	0.251
Model 7: smoking intentions (O) as the outcome variable																						
Knowledge (O)	1,179	**−0.41**	**0.10**	**<0.0001** ^**i**^	0.03	0.03	0.2230	−0.19	0.13	0.1232	−0.01	0.01	−0.04, 0.01	6.06	0.169 (0.012)	(1) 5636.94, *p* < 0.0001	1,383	(1) 0.920	(1) 0.914	(1) 0.051 (0.050, 0.052)	0.135	0.261
Self-report descriptive smoking norms 2 (L)		0.19	0.14	0.1543	0.07	0.07	0.2857				0.01	0.02	−0.01, 0.08	−6.49	0.299 (0.014)							
Self-efficacy: emotional (L)		**−0.46**	**0.09**	**<0.0001** ^**i**^	0.10	0.15	0.5086				−0.05	0.07	−0.15, 0.10	20.14	0.300 (0.014)							
																(2) 1910.05, *p* < 0.0001		(2) 0.945	(2) 0.941	(2) 0.043 (0.041, 0.045)		
Self-efficacy: friends (L)		**−0.39**	**0.09**	**<0.0001** ^**i**^	−0.12	0.14	0.3944				0.05	0.06	−0.02, 0.22	−20.94	0.522 (0.016)							
PBC (easy to quit; O)		**0.65**	**0.11**	**<0.0001** ^**i**^	−0.05	0.03	0.0780				−0.03	0.02	−0.08, 0.00004	14.91	0.395 (0.015)							
Model 8: smoking intentions (O) as the outcome variable																						
Self-efficacy: opportunity (L)	1,187	**−0.23**	**0.07**	**0.0016** ^**i**^	**0.21**	**0.05**	**<0.0001** ^**i**^	−0.11	0.07	0.1007	**−0.05**	**0.02**	**−0.09, −0.02**	**30.34**	1.000 (0.000)	(1) 2506.36, *p* < 0.0001	414	(1) 0.938	(1) 0.932	(1) 0.065 (0.063, 0.068)	0.072	0.146
																(2) 473.79, *p* = 0.0223		(2) 0.965	(2) 0.961	(2) 0.050 (0.044, 0.055)		
Model 9: smoking susceptibility (O) as the outcome variable																						
Self-report injunctive smoking norms (L)	1,070	−0.18	0.10	0.0862	−0.21	0.14	0.1319	0.42	0.60	0.4826	**0.04**	**0.04**	**0.0004, 0.15**	**10.59**	0.994 (0.002)	(1) 3329.30, *p* < 0.0001	741	(1) 0.857	(1) 0.904	(1) 0.057 (0.055, 0.059)	0.056	0.488
Self-report descriptive smoking norms 1 (L)		**0.61**	**0.14**	**<0.0001** ^**i**^	−0.14	0.23	0.5295				−0.09	0.16	−0.92, 0.03	−25.33	0.743 (0.014)							
Self-report descriptive smoking norms 2 (L)		**0.60**	**0.14**	**<0.0001** ^**i**^	−0.08	0.20	0.6971				−0.05	0.15	−0.72, 0.07	−13.37	0.194 (0.013)	(2) 2523.61, *p* < 0.0001		(2) 0.743	(2) 0.828	(2) 0.047 (0.045, 0.049)		
PBC (to avoid smoking; O)		**−0.30**	**0.08**	**0.0001** ^**i**^	−0.07	1.52	0.9621				0.02	0.55	−0.01, 0.22	6.17	0.446 (0.016)							
Model 10: smoking susceptibility (O) as the outcome variable																						
Knowledge (O)	1,070	**−0.40**	**0.09**	**<0.0001** ^**i**^	−0.05	0.04	0.2122	0.11	0.74	0.8803	0.02	0.02	−0.003, 0.05	6.33	0.407 (0.016)	(1) 1977.12, *p* < 0.0001	526	(1) 0.974	(1) 0.984	(1) 0.051 (0.048, 0.053)	0.033	0.571
Self-efficacy: Emotional (L)		**−0.25**	**0.08**	**0.0015** ^**i**^	**−0.47**	**0.15**	**0.0015** ^**i**^				**0.12**	**0.06**	**0.06, 0.23**	**36.10**	1.000 (0.000)							
																(2) 1679.95, *p* < 0.0001		(2) 0.889	(2) 0.931	(2) 0.045 (0.043, 0.048)		
Perceived social risks (L)		**−0.61**	**0.27**	**0.0260**	−0.13	0.16	0.4196				**0.08**	**0.69**	**0.01, 0.17**	**23.75**	0.904 (0.009)							
Model 11: smoking susceptibility (O) as the outcome variable																						
Self-efficacy: opportunity (L)	1,083	**−0.20**	**0.08**	**0.0096**	**−0.42**	**0.05**	**<0.0001** ^**i**^	**0.30**	**0.10**	**0.0023** ^**i**^	**0.08**	**0.03**	**0.02, 0.15**	**21.81**	1.000 (0.000)	(1) 2282.01, *p* < 0.0001	414	(1) 0.984	(1) 0.990	(1) 0.065 (0.062, 0.067)	0.048	0.489
																(2) 1777.87, *p* < 0.0001		(2) 0.915	(2) 0.948	(2) 0.055 (0.053, 0.058)		
Model 12: knowledge of smoking (O) as the outcome variable																						
Self-efficacy: emotional (L)	1,181	**−0.25**	**0.08**	**0.0013** ^**i**^	0.12	0.07	0.0619	**−0.21**	**0.10**	**0.0478**	**−0.03**	**0.02**	**−0.08, −0.002**	**8.31**	0.983 (0.004)	(1) 5306.78, *p* < 0.0001	1,303	(1) 0.925	(1) 0.920	(1) 0.051 (0.050, 0.052)	0.124	0.317
Self-efficacy: friends (L)		**−0.18**	**0.08**	**0.0147**	0.01	0.07	0.9198				−0.001	0.01	−0.03, 0.02	0.34	0.079 (0.009)							
Perceived social risks (L)		**−0.30**	**0.08**	**0.0003** ^**i**^	**0.27**	**0.06**	**<0.0001** ^**i**^				**−0.08**	**0.03**	**−0.15, −0.03**	**22.81**	1.000 (0.000)	(2) 2201.06, *p* < 0.0001		(2) 0.946	(2) 0.943	(2) 0.044 (0.042, 0.046)		
PBC (easy to quit; O)		**0.68**	**0.10**	**<0.0001** ^**i**^	−0.06	0.03	0.0502				**−0.04**	**0.02**	**−0.09, −0.001**	**11.61**	0.549 (0.016)							
Model 13: knowledge of smoking (O) as the outcome variable																						
Perceived addiction risks (L)	1,187	**−0.50**	**0.08**	**<0.0001** ^**i**^	**0.12**	**0.05**	**0.0137**	**−0.23**	**0.10**	**0.0238**	**−0.06**	**0.03**	**−0.12, −0.01**	**18.31**	0.959 (0.006)	(1) 524.95, *p* < 0.0001	72	(1) 0.773	(1) 0.660	(1) 0.073 (0.067, 0.079)	0.062	0.285
PBC (to avoid smoking; O)		**−0.39**	**0.08**	**<0.0001** ^**i**^	**0.09**	**0.04**	**0.0201**				**−0.03**	**0.02**	**−0.07, −0.01**	**10.44**	0.863 (0.011)	(2) 535.33, *p* < 0.0001		(2) 0.780	(2) 0.669	(2) 0.075 (0.069, 0.081)		
Model 14: knowledge of smoking (O) as the outcome variable																						
Self-efficacy: opportunity (L)	1,188	**−0.14**	**0.07**	**0.0429**	**0.11**	**0.04**	**0.0110**	**−0.36**	**0.09**	**0.0001** ^**i**^	**−0.02**	**0.01**	**−0.04, −0.001**	**4.09**	0.983 (0.004)	(1) 2296.05, *p* < 0.0001	414	(1) 0.945	(1) 0.939	(1) 0.062 (0.059, 0.064)	0.047	0.282
																(2) 455.77, *p* = 0.0766		(2) 0.971	(2) 0.967	(2) 0.046 (0.040, 0.051)		
Model 15: attitudes towards smoking (L) as the outcome variable																						
Knowledge (O)	1,187	**−0.38**	**0.10**	**0.0001** ^**i**^	**0.07**	**0.03**	**0.0050**	−0.11	0.07	0.1365	**−0.03**	**0.01**	**−0.06, −0.01**	**18.99**	0.621 (0.015)	(1) 1958.86, *p* < 0.0001	680	(1) 0.878	(1) 0.868	(1) 0.040 (0.038, 0.042)	0.054	0.253
Self-report descriptive smoking norms 2 (L)		0.16	0.09	0.0743	**0.13**	**0.04**	**0.0028**				**0.02**	**0.02**	**0.0004, 0.06**	**−14.59**	0.891 (0.010)							
																(2) 1537.43, *p* < 0.0001		(2) 0.888	(2) 0.879	(2) 0.039 (0.037, 0.041)		
PBC (to avoid smoking; O)		**−0.33**	**0.08**	**<0.0001** ^**i**^	**0.09**	**0.03**	**0.0004** ^**i**^				**−0.03**	**0.01**	**−0.06, −0.01**	**21.23**	0.830 (0.012)							
Model 16: attitudes towards smoking (L) as the outcome variable																						
Self-efficacy: emotional (L)	1,177	**−0.16**	**0.07**	**0.0179**	**0.28**	**0.05**	**<0.0001** ^**i**^	**0.15**	**0.07**	**0.0298**	**−0.04**	**0.02**	**−0.09, −0.01**	**−67.60**	1.000 (0.000)	(1) 3825.82, *p* < 0.0001	1,440	(1) 0.932	(1) 0.929	(1) 0.038 (0.036, 0.039)	0.058	0.276
Perceived social risks (L)		**−0.20**	**0.07**	**0.0078**	**0.15**	**0.05**	**0.0012** ^**i**^				**−0.03**	**0.01**	**−0.07, −0.01**	**−45.00**	0.976 (0.005)							
																(2) 2194.83, *p* < 0.0001		(2) 0.946	(2) 0.944	(2) 0.034 (0.032, 0.036)		
Exposure to advertising in shops (O)		**−0.32**	**0.08**	**0.0001** ^**i**^	0.05	0.03	0.1336				−0.01	0.01	−0.04, 0.003	−22.07	0.315 (0.015)							
Model 17: attitudes towards smoking (L) as the outcome variable																						
Self-efficacy: opportunity (L)	1,190	**−0.13**	**0.07**	**0.0450**	**0.22**	**0.04**	**<0.0001** ^**i**^	0.07	0.07	0.3247	**−0.03**	**0.02**	**−0.06, −0.002**	**−75.07**	0.996 (0.002)	(1) 4435.93, *p* < 0.0001	1,248	(1) 0.925	(1) 0.922	(1) 0.046 (0.045, 0.048)	0.052	0.232
																(2) 1543.70, *p* < 0.0001		(2) 0.949	(2) 0.947	(2) 0.039 (0.036, 0.041)		

a a = unstandardized regression coefficient for setting effect on mediator; b = unstandardized regression coefficient for association of mediator with outcome; c’ = unstandardized regression coefficient for direct effect; ab = unstandardized regression coefficient for indirect effect; SE = standard error; χ^2^ = Chi-square Goodness of Fit statistic; df = degrees of freedom (χ^2^ test); CFI = Comparative Fit Index; TLI = Tucker Lewis Index; RMSEA = Root Mean Square Error of Approximation; CI = confidence interval; SRMR = Standardized Root Mean Square Residual; R^2^ = R-squared; (O) = observed variable; (L) = latent variable; PBC = Perceived behavioral control.

b Results are unstandardized regression coefficients from single and multiple mediator structural equation models (SEMs). Bias-corrected bootstrap confidence intervals for the indirect effect are reported, along with the standard errors (standard errors and *p*-values are reported for the setting effect on the mediator, association of the mediator with the outcome, and direct effect). In each model, the predictor variable is setting (0 = Northern Ireland, 1 = Bogotá), the mediating variable is the follow-up score of the mediator, and the dependent variable is the follow-up score of the outcome variable. In addition to baseline values of the outcome and mediator, all models also include the following baseline variables as covariates: intervention group (0 = Dead Cool, 1 = ASSIST), gender (0 = boy, 1 = girl/prefer not to say), age (1 = 12 years or less, 2 = 13 years, 3 = 14 years or more; entered into SEMs as two dummy variables), ethnicity (0 = No ethnic minority, 1 = ethnic minority), and socio-economic status (NI: 1 = NIMDM2017 ≤ 296.6, 2 = 296.6 < NIMDM2017 ≤ 593.2, 3 = NIMDM2017 > 593.2; Bogotá: 1 = Informal settlement/Lowest/Low, 2 = Middle-Low/Middle, 3 = Middle-High/High; entered into SEMs as two dummy variables). The outcome variable in each model is shown in the row above where the results for each model are presented. See Supplementary Table S3 for details of variables. Bold and underlined text indicates tests which were significant at the *p* < 0.05 level. Structural equation models were specified using the maximum likelihood (ML) estimator with bias-corrected bootstrap standard errors (10,000 repetitions) and imputation of missing data by full information maximum likelihood (FIML). Structural equation models with smoking susceptibility as the outcome variable were specified using the diagonally weighted least squares (DWLS) estimator with bias-corrected bootstrap standard errors (10,000 repetitions). Models were run including the longitudinal measurement invariance constraints on the factor loadings, intercepts, and residual variances for latent variables (as outlined in Supplementary Tables S7, S8). Unstandardized parameter values are reported in Table 9 (std.lv, which standardizes all latent variables in the model only). Standardized parameter values are reported in Supplementary Table S53 (std.all, which standardizes all latent and observed variables in the model).

c Proportion of the total effect of setting on the outcome (unadjusted for the mediator) that is mediated via the mediator. In each single or multiple mediator model, the total effect (denominator) is equal to the sum of the direct effect (*c’*) and the indirect effect(s) (Σ*ab*).

d Power to detect the indirect effects calculated using Monte Carlo based statistical power analysis for mediation models with the “power.boot” function in the “bmem” package in R (119, 120).

e Model fit statistics were estimated using the maximum likelihood (ML) estimator. Robust variants of model fit statistics were calculated by specifying “test = ‘yuan.bentler’” in the call of the “sem” function. This calculates robust model fit statistics which are equivalent to those calculated by the robust maximum likelihood (MLR) estimator. For models with smoking susceptibility as the outcome variable, model fit statistics were estimated using the diagonally weighted least squares (DWLS) estimator. Robust variants of model fit statistics were calculated by specifying “test = ‘scaled.shifted’” in the call of the “sem” function. This calculates robust model fit statistics which are equivalent to those calculated by the weighted least square mean and variance adjusted (WLSMV) estimator.

f (1) standard test statistic; (2) robust test statistic. For single mediator models with observed outcome and mediator variables (O), the results were the same for the standard and robust tests (this applies for the model with objectively measured smoking behavior as the outcome variable).

g R-squared: the percentage of variation in the outcome variable explained by the model.

h For the model with objectively measured smoking behavior as the outcome variable, which is a single mediator model with observed outcome and mediator variables (O), the results were the same for the standard and robust tests.

i Retained statistical significance at the 5% level after using the Holm-Bonferroni procedure to correct the *p*-values for multiple testing (*p* < 0.05; based on the number of tests of each type of effect in Table 9, i.e., 40 tests for setting effects on mediators, 40 tests for association of mediators with outcomes, 17 tests for direct effects). The procedure is not applied for the indirect effects.

Models 1 and 2 included experimental donations to ASSIST/Dead Cool as the outcome variable. Significant indirect effects were identified for exposure to advertising in the media in model 1 (*ab* = −0.05, 95% CI: −0.12, −0.01) and opportunity self-efficacy in model 2 (*ab* = −0.03, 95% CI: −0.07, −0.004). Both met all the criteria for mediation (significant *a*-, *b*-, and *ab*-paths) with −10.05% and −5.70% of the total effect mediated, respectively. Experimental donations to ASSIST/Dead Cool were higher in NI compared to Bogotá at follow-up due to NI participants’ lower exposure to advertising in the media and higher opportunity self-efficacy (controlling for baseline). However, the negative proportions mediated indicate suppressor effects with mediators acting in the opposite direction to the total effect. Without the mediating effects of exposure to advertising in the media and opportunity self-efficacy, the total effect of setting on experimental donations would have been more strongly in the opposite direction, with even higher donations in Bogotá compared to NI at follow-up.

Models 3 to 5 included self-report smoking behavior as the outcome variable. In model 3, a significant indirect effect was observed for PBC that it would be easy to quit smoking (*ab* = 0.02, 95% CI: 0.003, 0.04). In model 4, significant indirect effects were identified for emotional self-efficacy (*ab* = −0.02, 95% CI: −0.06, −0.005) and friend self-efficacy (*ab* = −0.02, 95% CI: −0.05, −0.004). In model 5, there was a significant indirect effect for opportunity self-efficacy (*ab* = −0.03, 95% CI: −0.05, −0.01). All four mediators met the criteria for mediation with proportions mediated of 103.48, 91.44, 70.35, and 122.13%, respectively. The total effect of setting on self-report smoking behavior was non-significant (Table 3), and the mediators acted in the opposite direction with self-efficacy raising anti-smoking behavior in NI and PBC raising anti-smoking behavior in Bogotá.

Model 6 included objectively measured smoking behavior as the outcome variable. There were no significant indirect effects or mediators.

Models 7 and 8 included self-report smoking intentions as the outcome variable. A significant indirect effect was observed for opportunity self-efficacy in model 8 (*ab* = −0.05, 95% CI: −0.09, −0.02), which met all the criteria for mediation with a proportion mediated of 30.34%. NI participants compared to Bogotá had higher anti-smoking intentions at follow-up due to their higher levels of opportunity self-efficacy (controlling for baseline).

Models 9 to 11 included smoking susceptibility as the outcome variable. In model 9, a significant indirect effect was observed for self-report injunctive smoking norms (*ab* = 0.04, 95% CI: 0.0004, 0.15). In model 10, significant indirect effects were observed for emotional self-efficacy (*ab* = 0.12, 95% CI: 0.06, 0.23) and perceived social risks (*ab* = 0.08, 95% CI: 0.01, 0.17). In model 11, there was a significant indirect effect for opportunity self-efficacy (*ab* = 0.08, 95% CI: 0.02, 0.15). Emotional and opportunity self-efficacy met all the criteria for mediation with proportions mediated of 36.10 and 21.81%, respectively. NI participants compared to Bogotá had lower smoking susceptibility at follow-up due to their higher levels of self-efficacy (controlling for baseline).

Models 12 to 14 included knowledge of the effects of smoking as the outcome variable. In model 12, significant indirect effects were observed for emotional self-efficacy (*ab* = −0.03, 95% CI: −0.08, −0.002), perceived social risks (*ab* = −0.08, 95% CI: −0.15, −0.03) and PBC that it would be easy to quit smoking (*ab* = −0.04, 95% CI: −0.09, −0.001). In model 13, significant indirect effects were observed for perceived addiction risks (*ab* = −0.06, 95% CI: −0.12, −0.01) and PBC to avoid smoking (*ab* = −0.03, 95% CI: −0.07, −0.01). In model 14, there was a significant indirect effect for opportunity self-efficacy (*ab* = −0.02, 95% CI: −0.04, −0.001). Perceived social and addiction risks, PBC to avoid smoking, and opportunity self-efficacy met all the criteria for mediation with proportions mediated of 22.81, 18.31, 10.44, and 4.09%, respectively. NI participants compared to Bogotá had higher knowledge at follow-up due to their higher levels of perceived social and addiction risks, PBC to avoid smoking, and opportunity self-efficacy (controlling for baseline).

Models 15 to 17 included attitudes towards smoking as the outcome variable. In model 15, significant indirect effects were observed for knowledge (*ab* = −0.03, 95% CI: −0.06, −0.01), self-report descriptive smoking norms scale 2 (*ab* = 0.02, 95% CI: 0.0004, 0.06), and PBC to avoid smoking (*ab* = −0.03, 95% CI: −0.06, −0.01). In model 16, significant indirect effects were observed for emotional self-efficacy (*ab* = −0.04, 95% CI: −0.09, −0.01) and perceived social risks (*ab* = −0.03, 95% CI: −0.07, −0.01). In model 17, there was a significant indirect effect for opportunity self-efficacy (*ab* = −0.03, 95% CI: −0.06, −0.002). Knowledge, PBC to avoid smoking, emotional and opportunity self-efficacy, and perceived social risks met all the criteria for mediation with proportions mediated of 18.99, 21.23, −67.60%, −75.07%, and −45.00%, respectively. Anti-smoking attitudes were higher for NI participants compared to Bogotá at follow-up due to their higher knowledge, PBC to avoid smoking, self-efficacy, and perceived social risks (controlling for baseline). The total effect of setting on attitudes was non-significant (Table 3), suggesting that other unmeasured mediators may have acted in the opposite direction to increase anti-smoking attitudes in Bogotá.

The results of single mediator models with setting as the predictor variable are reported in Supplementary Tables S54–S67.

### 3.3 Sensitivity analyses comparing MECHANISMS participants with the Northern Ireland control group from the Dead Cool study

The results of SEMs comparing MECHANISMS setting/intervention groups with the NI control group are reported in Table 10 (unstandardized coefficients) and Supplementary Table S68 (standardized coefficients). At follow-up, controlling for baseline, more pro-smoking values of self-report smoking behavior were observed for Bogotá Dead Cool schools compared with the NI control group. Objectively measured smoking behavior was more pro-smoking at follow-up for all four groups compared with the NI control. Smoking intentions were more pro-smoking at follow-up for Bogotá ASSIST compared with the NI control, and smoking susceptibility was higher for both Bogotá Dead Cool and Bogotá ASSIST. Exposure to advertising in shops was lower at follow-up for Bogotá Dead Cool and Bogotá ASSIST compared with the NI control. R^2^ ranged between 0.131 and 0.484 and power ranged between 0.833 and 1.000.

**Table 10:** Sensitivity analyses with structural equation models including predictor variables comparing MECHANISMS setting/intervention groups with the Northern Ireland control group.

Outcome^a^	*n*	NI Dead Cool^b^			NI ASSIST^c^			Bogotá Dead Cool^d^			Bogotá ASSIST^e^			Model fit statistics^a^^,^^f^		Power^h^
		b	SE	*p*-value	b	SE	*p*-value	b	SE	*p*-value	b	SE	*p*-value	SRMR	R^2,^^g^	
Observed outcome variables (O)																
Self-report smoking behavior (1 to 4)	1,374	0.03	0.04	0.4425	−0.06	0.05	0.1952	**−0.09**	**0.04**	**0.0459**	0.02	0.04	0.5412	0.000	0.484	0.833
Objectively measured smoking behavior (0 to 30)	1,303	**0.28**	**0.10**	**0.0054** ^**j**^	**0.25**	**0.11**	**0.0296**	**1.49**	**0.14**	**<0.0001** ^**j**^	**1.39**	**0.14**	**<0.0001** ^**j**^	0.000	0.272	1.000
Intentions (1 to 6)	1,369	0.06	0.07	0.3815	−0.08	0.08	0.3460	−0.03	0.09	0.7103	**−0.29**	**0.09**	**0.0015** ^**j**^	0.000	0.131	0.964
Smoking susceptibility (0 = not susceptible; 1 = susceptible)^i^	1,245	0.03	0.15	0.8230	0.09	0.15	0.5541	**0.30**	**0.15**	**0.0401**	**0.60**	**0.15**	**<0.0001** ^**j**^	0.000	0.356	1.000
Exposure to advertising in shops (0 to 4)	1,357	−0.07	0.11	0.5500	−0.19	0.11	0.0881	**−0.36**	**0.10**	**0.0005** ^**j**^	**−0.53**	**0.11**	**<0.0001** ^**j**^	0.000	0.214	0.999

a b = unstandardized regression coefficient; SE = standard error; SRMR = Standardized Root Mean Square Residual; R^2^ = R-squared; (O) = observed variable.

b Regression coefficients represent the average change in the observed outcome variable for NI Dead Cool schools compared to NI control schools, adjusted for baseline values of the outcome variable. In addition to baseline values of the outcome, all models also include the following baseline variables as covariates: dummy variables indicating NI ASSIST schools (0 = NI control schools and MECHANISMS schools other than NI ASSIST, 1 = NI ASSIST schools), Bogotá Dead Cool schools (0 = NI control schools and MECHANISMS schools other than Bogotá Dead Cool, 1 = Bogotá Dead Cool schools), Bogotá ASSIST schools (0 = NI control schools and MECHANISMS schools other than Bogotá ASSIST, 1 = Bogotá ASSIST schools), gender (0 = boy, 1 = girl/prefer not to say), age (1 = 12 years or less, 2 = 13 years, 3 = 14 years or more; entered into SEMs as two dummy variables), ethnicity (0 = No ethnic minority, 1 = ethnic minority), and socio-economic status (NI: 1 = NIMDM2017 ≤ 296.6, 2 = 296.6 < NIMDM2017 ≤ 593.2, 3 = NIMDM2017 > 593.2; Bogotá: 1 = Informal settlement/Lowest/Low, 2 = Middle-Low/Middle, 3 = Middle-High/High; entered into SEMs as two dummy variables). See Supplementary Table S3 for details of variables. Bold and underlined text indicates tests which were significant at the *p* < 0.05 level. Structural equation models were specified using maximum likelihood estimation with robust (Huber-White) standard errors (MLR estimator) and imputation of missing data by full information maximum likelihood (FIML). Structural equation models with smoking susceptibility as the outcome variable were specified using the weighted least square mean and variance adjusted (WLSMV) estimator. Unstandardized parameter values are reported in Table 10 (std.lv, which standardizes all latent variables in the model only). Standardized parameter values are reported in Supplementary Table S68 (std.all, which standardizes all latent and observed variables in the model).

c Regression coefficients represent the average change in the observed outcome variable for NI ASSIST schools compared to NI control schools, adjusted for baseline values of the outcome variable, dummy variables indicating NI Dead Cool schools, Bogotá Dead Cool schools and Bogotá ASSIST schools, gender, age, ethnicity, and socio-economic status. Bold and underlined text indicates tests which were significant at the *p* < 0.05 level.

d Regression coefficients represent the average change in the observed outcome variable for Bogotá Dead Cool schools compared to NI control schools, adjusted for baseline values of the outcome variable, dummy variables indicating NI ASSIST schools, NI Dead Cool schools and Bogotá ASSIST schools, gender, age, ethnicity, and socio-economic status. Bold and underlined text indicates tests which were significant at the *p* < 0.05 level.

e Regression coefficients represent the average change in the observed outcome variable for Bogotá ASSIST schools compared to NI control schools, adjusted for baseline values of the outcome variable, dummy variables indicating NI ASSIST schools, NI Dead Cool schools and Bogotá Dead Cool schools, gender, age, ethnicity, and socio-economic status. Bold and underlined text indicates tests which were significant at the *p* < 0.05 level.

f Model fit statistics were estimated using the robust (Huber-White) maximum likelihood (MLR) estimator. For models with smoking susceptibility as the outcome variable, model fit statistics were estimated using the weighted least square mean and variance adjusted (WLSMV) estimator. For all models, the chi-square goodness of fit statistic (χ^2^) = 0.00 with 0 degrees of freedom and *p* = 1.00. The Comparative Fit Index (CFI) and Tucker Lewis Index (TLI) = 1.000. The Root Mean Square Error of Approximation (RMSEA) and 90% confidence interval = 0.000 (0.000, 0.000). For models with observed outcome variables (O), the results were the same for the standard and robust tests.

g R-squared: the percentage of variation in the outcome variable explained by the model.

h Power to detect the observed “Intervention” and “Setting” effects with a significance level of *p* < 0.05, calculated using WebPower. For models with observed outcome variables (O), the “linear regression” method was used.

i Structural equation models with smoking susceptibility as the outcome variable were specified using the weighted least square mean and variance adjusted (WLSMV) estimator.

j Retained statistical significance at the 5% level after using the Holm-Bonferroni procedure to correct the *p*-values for multiple testing (*p* < 0.05; based on the number of models in Table 10, i.e., 5 tests).

The results of SEMs comparing MECHANISMS setting/intervention groups with the NI control group, analyzing the ASSIST non-PS and ASSIST PS separately, are reported in Table 11 (unstandardized coefficients) and Supplementary Table S69 (standardized coefficients). The overall pattern of results was consistent with the models comparing all ASSIST participants with the NI control group. The significant effects for the NI ASSIST and Bogotá ASSIST groups compared to NI control were mostly observed in both the non-PS and PS groups. However, there were no statistically significant effects for NI ASSIST PS for objectively measured smoking behavior, or for Bogotá ASSIST PS for intentions. R^2^ ranged between 0.132 and 0.486 and power ranged between 0.920 and 1.000.

**Table 11:** Sensitivity analyses with structural equation models including predictor variables comparing MECHANISMS setting/intervention groups with the Northern Ireland control group, with ASSIST non-peer supporters and ASSIST peer supporters analyzed separately.

Outcome^a^	n	NI Dead Cool^b^			NI ASSIST non-peer supporters^c^			NI ASSIST peer supporters^d^			Bogotá Dead Cool^e^			Bogotá ASSIST non-peer supporters^f^			Bogotá ASSIST peer supporters^g^			Model fit statistics^a^^,^^h^		Power^j^
		b	SE	*p*-value	b	SE	*p*-value	b	SE	*p*-value	b	SE	*p*-value	b	SE	*p*-value	b	SE	*p*-value	SRMR	R^2,^^i^	
Observed outcome variables (O)																						
Self-report smoking behavior	1,374	0.03	0.04	0.4300	−0.09	0.05	0.0768	0.07	0.07	0.3079	**−0.09**	**0.04**	**0.0491**	0.02	0.04	0.5798	0.04	0.07	0.6177	0.000	0.486	0.920
Objectively measured smoking behavior	1,303	**0.28**	**0.10**	**0.0054** ^**l**^	**0.24**	**0.12**	**0.0485**	0.30	0.17	0.0737	**1.50**	**0.14**	**<0.0001** ^**l**^	**1.36**	**0.15**	**<0.0001** ^**l**^	**1.50**	**0.24**	**<0.0001** ^**l**^	0.000	0.272	1.000
Intentions	1,369	0.06	0.07	0.3848	−0.07	0.08	0.3998	−0.11	0.17	0.5118	−0.03	0.09	0.7117	**−0.33**	**0.10**	**0.0015** ^**l**^	−0.15	0.14	0.2995	0.000	0.132	0.964
Smoking susceptibility^k^	1,245	0.04	0.15	0.8109	0.04	0.16	0.8229	0.29	0.22	0.1742	**0.30**	**0.15**	**0.0383**	**0.63**	**0.15**	**<0.0001** ^**l**^	**0.49**	**0.24**	**0.0380**	0.000	0.357	1.000
Exposure to advertising in shops	1,357	−0.07	0.11	0.5521	−0.21	0.12	0.0749	−0.10	0.19	0.5908	**−0.36**	**0.10**	**0.0005** ^**l**^	**−0.49**	**0.11**	**<0.0001** ^**l**^	**−0.70**	**0.15**	**<0.0001** ^**l**^	0.000	0.216	0.999

a b = unstandardized regression coefficient; SE = standard error; SRMR = Standardized Root Mean Square Residual; R^2^ = R-squared; (O) = observed variable.

b Regression coefficients represent the average change in the observed outcome variable for NI Dead Cool schools compared to NI control schools, adjusted for baseline values of the outcome variable. In addition to baseline values of the outcome, all models also include the following baseline variables as covariates: NI ASSIST non-peer supporters (0 = NI control schools and MECHANISMS participants other than NI ASSIST non-peer supporters, 1 = NI ASSIST non-peer supporters), NI ASSIST peer supporters (0 = NI control schools and MECHANISMS participants other than NI ASSIST peer supporters, 1 = NI ASSIST peer supporters), Bogotá Dead Cool schools (0 = NI control schools and MECHANISMS schools other than Bogotá Dead Cool, 1 = Bogotá Dead Cool schools), Bogotá ASSIST non-peer supporters (0 = NI control schools and MECHANISMS participants other than Bogotá ASSIST non-peer supporters, 1 = Bogotá ASSIST non-peer supporters), Bogotá ASSIST peer supporters (0 = NI control schools and MECHANISMS participants other than Bogotá ASSIST peer supporters, 1 = Bogotá ASSIST peer supporters), gender (0 = boy, 1 = girl/prefer not to say), age (1 = 12 years or less, 2 = 13 years, 3 = 14 years or more; entered into SEMs as two dummy variables), ethnicity (0 = No ethnic minority, 1 = ethnic minority), and socio-economic status (NI: 1 = NIMDM2017 ≤ 296.6, 2 = 296.6 < NIMDM2017 ≤ 593.2, 3 = NIMDM2017 > 593.2; Bogotá: 1 = Informal settlement/Lowest/Low, 2 = Middle-Low/Middle, 3 = Middle-High/High; entered into SEMs as two dummy variables). See Supplementary Table S3 for details of variables. Bold and underlined text indicates tests which were significant at the *p* < 0.05 level. Structural equation models were specified using maximum likelihood estimation with robust (Huber-White) standard errors (MLR estimator) and imputation of missing data by full information maximum likelihood (FIML). Structural equation models with smoking susceptibility as the outcome variable were specified using the weighted least square mean and variance adjusted (WLSMV) estimator. Unstandardized parameter values are reported in Table 11 (std.lv, which standardizes all latent variables in the model only). Standardized parameter values are reported in Supplementary Table S69 (std.all, which standardizes all latent and observed variables in the model).

c Regression coefficients represent the average change in the observed outcome variable for NI ASSIST non-peer supporters compared to NI control schools, adjusted for baseline values of the outcome variable, dummy variables indicating NI Dead Cool schools, NI ASSIST peer supporters, Bogotá Dead Cool schools, Bogotá ASSIST non-peer supporters and Bogotá ASSIST peer supporters, gender, age, ethnicity, and socio-economic status. Bold and underlined text indicates tests which were significant at the *p* < 0.05 level.

d Regression coefficients represent the average change in the observed outcome variable for NI ASSIST peer supporters compared to NI control schools, adjusted for baseline values of the outcome variable, dummy variables indicating NI Dead Cool schools, NI ASSIST non-peer supporters, Bogotá Dead Cool schools, Bogotá ASSIST non-peer supporters and Bogotá ASSIST peer supporters, gender, age, ethnicity, and socio-economic status. Bold and underlined text indicates tests which were significant at the *p* < 0.05 level.

e Regression coefficients represent the average change in the observed outcome variable for Bogotá Dead Cool schools compared to NI control schools, adjusted for baseline values of the outcome variable, dummy variables indicating NI Dead Cool schools, NI ASSIST non-peer supporters, NI ASSIST peer supporters, Bogotá ASSIST non-peer supporters and Bogotá ASSIST peer supporters, gender, age, ethnicity, and socio-economic status. Bold and underlined text indicates tests which were significant at the *p* < 0.05 level.

f Regression coefficients represent the average change in the observed outcome variable for Bogotá ASSIST non-peer supporters compared to NI control schools, adjusted for baseline values of the outcome variable, dummy variables indicating NI Dead Cool schools, NI ASSIST non-peer supporters, NI ASSIST peer supporters, Bogotá Dead Cool schools and Bogotá ASSIST peer supporters, gender, age, ethnicity, and socio-economic status. Bold and underlined text indicates tests which were significant at the *p* < 0.05 level.

g Regression coefficients represent the average change in the observed outcome variable for Bogotá ASSIST peer supporters compared to NI control schools, adjusted for baseline values of the outcome variable, dummy variables indicating NI Dead Cool schools, NI ASSIST non-peer supporters, NI ASSIST peer supporters, Bogotá Dead Cool schools and Bogotá ASSIST non-peer supporters, gender, age, ethnicity, and socio-economic status. Bold and underlined text indicates tests which were significant at the *p* < 0.05 level.

h Model fit statistics were estimated using the robust (Huber-White) maximum likelihood (MLR) estimator. For models with smoking susceptibility as the outcome variable, model fit statistics were estimated using the weighted least square mean and variance adjusted (WLSMV) estimator. For all models, the chi-square goodness of fit statistic (χ^2^) = 0.00 with 0 degrees of freedom and *p* = 1.00. The Comparative Fit Index (CFI) and Tucker Lewis Index (TLI) = 1.000. The Root Mean Square Error of Approximation (RMSEA) and 90% confidence interval = 0.000 (0.000, 0.000). For models with observed outcome variables (O), the results were the same for the standard and robust tests.

i R-squared: the percentage of variation in the outcome variable explained by the model.

j Power to detect the observed “Intervention” and “Setting” effects with a significance level of *p* < 0.05, calculated using WebPower. For models with observed outcome variables (O), the “linear regression” method was used.

k Structural equation models with smoking susceptibility as the outcome variable were specified using the weighted least square mean and variance adjusted (WLSMV) estimator.

l Retained statistical significance at the 5% level after using the Holm-Bonferroni procedure to correct the *p*-values for multiple testing (*p* < 0.05; based on the number of models in Table 11, i.e., 5 tests).

#### 3.3.1 Mediator models

The results of the final single and multiple mediator models with predictor variables comparing MECHANISMS setting/intervention groups with the NI control group are reported in Supplementary Table S70 (unstandardized parameter estimates) and Supplementary Table S71 (standardized parameter estimates). In the model with self-report smoking behavior as the outcome variable, smoking intentions was a significant mediator for Bogotá ASSIST participants compared to NI control (*ab* = −0.03, 95% CI: −0.06, −0.01). However, the total effect of Bogotá ASSIST versus NI control on self-report smoking behavior was non-significant, suggesting that unmeasured mediators may have acted to cancel out the negative mediating effect of intentions for Bogotá ASSIST schools. R^2^ ranged between 0.134 and 0.524. Power to detect indirect effects ranged between 0.047 and 0.958. The results of the single mediator models are reported in Supplementary Tables S72, S73.

The results of the final single and multiple mediator models comparing MECHANISMS setting/intervention groups with the NI control group, analyzing ASSIST non-PS and ASSIST PS separately, are reported in Supplementary Table S74 (unstandardized parameter estimates) and Supplementary Table S75 (standardized parameter estimates). The negative mediating effect of intentions on self-report smoking behavior for Bogotá ASSIST compared to NI control was significant for the ASSIST non-PS group only. R^2^ ranged between 0.135 and 0.525. Power to detect indirect effects ranged between 0.050 and 0.966. The results of the single mediator models are reported in Supplementary Tables S76, S77.

## 4 Discussion

This paper compares intervention mechanisms between two school-based smoking prevention programs—ASSIST and Dead Cool—among adolescents participating in the MECHANISMS study in a high-income setting (NI) and a middle-income setting (Bogotá) in 2019 (1). The MECHANISMS study was not designed to provide a direct comparison of the overall effectiveness of the two programs, both of which had previously been evaluated in cluster RCTs in the UK. Accordingly, a control group was not included in the MECHANISMS study design, which instead focused on exploring the mechanisms of change. Logic models outlining how the ASSIST and Dead Cool programs were hypothesized to influence smoking behavior, intentions, susceptibility, knowledge, and attitudes via specific BCTs and targeted mediating constructs were published in the MECHANISMS study protocol (1). In the present paper, we conducted mediation analyses to compare these hypothesized intervention mechanisms between the two programs and research settings. Experimental donations to ASSIST/Dead Cool—representing participants’ WTP to support anti-smoking norms—were also modelled as an outcome variable in the mediator models, as this was the primary behavioral outcome in the MECHANISMS study’s novel behavioral economics and game theory experiments measuring social norms for adolescent smoking and vaping. Detailed assessment of norms, psychosocial constructs and other mediating constructs were not conducted in the original ASSIST and Dead Cool trials. However, several smoking-related outcomes—including self-report and objectively measured smoking behavior, intentions, susceptibility, and exposure to advertising in shops—were also collected in the original Dead Cool RCT conducted in 2014 in NI (8). Therefore, as a sensitivity analysis, we compared these outcomes for MECHANISMS schools implementing either ASSIST or Dead Cool, with NI control group schools from the previously published Dead Cool study who received treatment-as-usual.

### 4.1 Comparison of intervention mechanisms between ASSIST and Dead Cool

When comparing the targeted outcomes and mediators between the interventions, most of the significant intervention effects showed more anti-smoking outcomes for Dead Cool schools compared to ASSIST at follow-up, controlling for baseline. This pattern was observed for smoking intentions, susceptibility, knowledge of the effects of smoking, attitudes towards smoking, experimental injunctive smoking/vaping norms, self-report injunctive and descriptive smoking norms, self-efficacy to resist smoking, perceived physical and social risks of smoking, and PBC to avoid smoking. These findings reflect the theoretical framework, and content of the Dead Cool program. Dead Cool is grounded in the theory of planned behavior, which includes intentions, attitudes, normative beliefs, and PBC as central constructs (38, 64). The course emphasizes the health, social, environmental, and emotional consequences of smoking, encouraging reflection on anticipated regret and the salience of health risks. This directly targets outcomes like intentions, knowledge, attitudes, and perceived risks. Knowledge and perceived risks are also targeted since pupils are asked to evaluate the pros and cons of smoking, and practice using persuasive language to debunk myths about smoking. The intervention directly targets smoking intentions as pupils are asked to sign a “Self-Promise Contract” agreeing to remain smoke-free and make plans to reward themselves for achieving their non-smoking goals (behavioral contract, goal setting, self-incentive). Normative beliefs are addressed by providing accurate data on smoking prevalence (descriptive norms) and approval of smoking (injunctive norms), and by prompting pupils to seek social support for remaining smoke-free. The program also increases pupils’ self-efficacy and PBC to quit or remain tobacco-free using BCTs like positive self-talk, problem solving, behavioral practice/rehearsal, identification of self as a role model, and social support (Supplementary Table S2). Thus, the eight-week, multi-component, classroom-delivered Dead Cool program effectively targeted smoking-related outcomes through its hypothesized mediating constructs.

Most significant mediators described how Dead Cool participants demonstrated higher self-report anti-smoking behavior, intentions, and attitudes, lower susceptibility, and greater knowledge than ASSIST participants at follow-up. However, in models with self-report smoking behavior as the outcome, the mediators exerted suppressive effects. Although the total effect of intervention on self-report smoking behavior was non-significant, Dead Cool schools would have shown more pro-smoking behavior relative to ASSIST without the mediating effects of intentions, attitudes, and opportunity self-efficacy. This suggests potential unmeasured mediators acting in the opposite direction to enhance anti-smoking behavior in ASSIST schools.

Another notable finding is that exposure to advertising in shops and the media was higher in Dead Cool schools compared to ASSIST at follow-up controlling for baseline. We considered this to be a negative outcome for Dead Cool compared to ASSIST. However, higher exposure to advertising in shops was positively associated with anti-smoking attitudes and significantly mediated the increase in anti-smoking attitudes among Dead Cool pupils versus ASSIST. One explanation may be that pupils considered negative or regulatory messaging seen in shops (e.g., health warnings on cigarette packaging). Conversely, lower exposure to advertising in the media significantly mediated an increase in experimental donations (WTP to support anti-smoking norms) for ASSIST compared to Dead Cool, although the total effect was non-significant. Since Dead Cool emphasizes media and social media influences on smoking, pupils may have become more aware of tobacco advertising after completing the program.

At the time of data collection, NI and Colombia both had comprehensive legislation banning tobacco advertising in shops and the media (52–54, 59, 60). Standardized packaging has been fully implemented in the UK since May 2017, and 75% of each cigarette pack must display health warnings and graphic images. The legislation also bans retail displays and cigarette sales from vending machines (54). In Colombia, standardized packaging had not been introduced. However, under the WHO-FCTC, tobacco packs were required to include health warnings covering at least 30% of the surface, with restricted public accessibility in shops and bans on vending machine sales (52–54, 59, 60). Both countries also required warning signs prohibiting tobacco sales to minors in shops (59, 60). Although sales of e-cigarettes to minors were prohibited, they were less stringently regulated in both countries in 2019. No bans on retail displays were in place, and marketing and promotion via social media were widespread. Evidence suggests that exposure to large-scale social media marketing has been associated with more positive perceptions of e-cigarettes among young people (121–124). Legislation regulating ENDS and nicotine substitutes was only introduced in Colombia in 2024, while the UK is currently introducing stricter legislation to regulate e-cigarettes similar to the tobacco control measures (55, 56, 63). In MECHANISMS, we observed opposing mediation effects for exposure to advertising in shops versus the media. Higher exposure to advertising in shops increased anti-smoking attitudes for Dead Cool schools, whilst lower exposure to media advertising increased WTP to support anti-smoking norms for ASSIST. This pattern may reflect differences in how pupils interpret advertising contexts, with shop-based regulatory warnings acting as deterrents and media portrayals more often promoting smoking or use of e-cigarettes and other nicotine substitutes.

Since the intervention mechanisms may differ for the ASSIST PS and non-PS, outcomes were compared for each subgroup relative to Dead Cool. Notably, Dead Cool schools showed higher smoking knowledge at follow-up than ASSIST non-PS, but the opposite was true for the ASSIST PS who showed greater knowledge than Dead Cool pupils. ASSIST PS also demonstrated higher self-report anti-smoking behavior, greater perceived addiction risks, and lower exposure to media advertising than Dead Cool pupils. For ASSIST non-PS, only lower exposure to media advertising was significant. Apart from self-report descriptive norms, most intervention effects favoring Dead Cool over ASSIST at follow-up were limited to the non-PS group but were non-significant for the PS group. However, it is also important to note the reduced power to detect effects due to the smaller sample size in the ASSIST PS group. Overall, these findings are consistent with the content and delivery of the ASSIST program. During training, peer supporters improve their smoking knowledge by learning about the ingredients of a cigarette, and the health, social, emotional, and environmental consequences of smoking. The peer supporters also discuss the pros and cons of smoking, which targets perceived risks and benefits. Meanwhile, smoking behavior is directly targeted as the peer supporters are encouraged to quit if they already smoke with emphasis placed on their role-model status (Supplementary Table S2).

Whilst the ASSIST training and follow-ups improved knowledge, self-report smoking behavior, and perceived addiction risks for the ASSIST PS compared to Dead Cool, any subsequent diffusion of these benefits to the wider school cohort via the intervention’s peer communication channels appears to have been too limited to produce a detectable improvement for the ASSIST non-PS relative to Dead Cool. Follow-up survey data showed that 68.1% of Dead Cool participants, 61.7% of ASSIST participants, and 87.3% of ASSIST PS recalled conversations about smoking, with 17.6, 13.4, and 28.3% recalling more than five such discussions, respectively. Since the communication channels are vital to the success of peer education and diffusion programs, future research should explore ways to strengthen and evaluate their effectiveness. To enhance diffusion, future peer-led or social network-based interventions could incorporate environmental prompts (e.g., posters, classroom reminders) and social cues (e.g., text or email prompts) to encourage peer supporters’ engagement, as similar strategies have effectively promoted health behaviors in previous studies (125–127). Although ASSIST includes optional poster activities during training, making these a required or more visible component within the school might strengthen message diffusion. Interventionists might also consider alternative criteria for identifying “influential” pupils, such as using social network analysis to select the most central individuals (35), or recruiting a larger portion of the year group to encourage more conversations. Since the peer supporters’ conversations are central to ASSIST’s mechanism of change, future evaluations should consider examining mediators linked to peer supporter effectiveness (e.g., self-efficacy as a peer educator, social support, or frequency of conversations), ensuring that the hypothesized mechanisms are explicitly testable within intervention designs.

### 4.2 Comparison between the research settings

According to the Medical Research Council guidance on developing complex interventions, the context within which an intervention operates can influence both its implementation and effectiveness (41, 128, 129). The MECHANISMS study was designed to compare intervention mechanisms between schools in a high-income setting (NI) and a middle-income setting (Bogotá), providing a unique opportunity to examine how contextual factors affect intervention processes and outcomes. Previous reviews have highlighted the need for high-quality studies of school-based smoking prevention interventions in LMICs, including successful strategies from high-income settings adapted for the local culture and context. Trials of multiple-component interventions—considering cultural, environmental, psychological and social factors—with deep cultural adaptation are particularly recommended (22–24). MECHANISMS partly addresses this gap, as both ASSIST and Dead Cool are complex, multi-component interventions that have shown effectiveness in reducing adolescent smoking uptake in UK trials and were carefully culturally adapted for Bogotá (7–9).

When comparing our targeted outcomes and mediators across settings, most significant effects indicated stronger anti-smoking outcomes among NI schools at follow-up, controlling for baseline. However, Bogotá schools showed more positive effects for experimental donations to ASSIST/Dead Cool, self-report descriptive norms, and reduced exposure to advertising in shops. These findings support the use of social norms intervention strategies in LMICs. Social norms interventions aim to correct misperceptions within a reference group—for example, the belief that “most adolescents smoke”—by providing accurate information about the actual prevalence of the behavior (descriptive norms) (130). Social norms strategies are important components in both the ASSIST and Dead Cool programs (7, 8). Previous research also recommends combining descriptive norms (what others do) with injunctive norms (what others approve of) to avoid inadvertently reinforcing the perception that some peers do smoke (35, 131, 132). At follow-up, participants in Bogotá made higher experimental donations to ASSIST/Dead Cool, which reflects their greater WTP to support anti-smoking norms and suggests that they viewed school-based smoking prevention programs like ASSIST and Dead Cool as normatively appealing and effective. Tobacco education is not yet formally embedded in Colombia’s school curriculum, as in the UK, and recent research highlights that exposure to school-based smoking prevention programs is generally suboptimal in LMICs (133). These results provide further evidence that more widespread implementation of school-based programs could be both appealing and effective for adolescent smoking prevention in LMICs, and should be considered in ongoing tobacco control efforts (22, 24, 133).

At follow-up, exposure to advertising in shops was lower in Bogotá, whilst exposure to advertising in the media was lower in NI. Since Colombia implemented the WHO-FCTC in 2009, compliance with tobacco advertising and sales restrictions has been monitored through police inspections of retail establishments—including the types of shops our survey asks about (e.g., supermarkets, newsagents, sweet shops, and petrol stations) (59, 60). However, adolescents may still obtain tobacco products through informal means such as contraband cigarettes or unregulated street vendors, which are more difficult to monitor (60). To reflect this context, Bogotá participants answered five additional items about exposure to advertising in local neighborhood shops (e.g., street booths or candy stands, neighborhood stores, cigar and liquor stores, vending machines, and bars or restaurants). On average, Bogotá participants reported seeing tobacco advertisements in 2.11 formal shop types and in a further 2.97 informal shop types, indicating that unregulated retail channels remain a key source of exposure.

Most of the significant mediating effects described how NI participants showed greater anti-smoking outcomes compared to Bogotá at follow-up, controlling for baseline. However, PBC—specifically, the belief that quitting would be easy—was a significant mediator that increased self-report anti-smoking behavior among Bogotá participants. Conversely, higher PBC that it would be easy to quit smoking was negatively associated with smoking knowledge, producing a significant indirect effect that increased knowledge among NI schools (in the opposite direction to its mediating effect on self-report behavior). One explanation may be that pupils who report never having smoked underestimate how addiction could affect their ability to quit, whereas pupils with greater smoking knowledge are more aware of nicotine’s addictive nature.

In models with experimental donations to ASSIST/Dead Cool and attitudes towards smoking as the outcome variables, the mediators exerted suppressive effects, suggesting there were unmeasured mediators acting in the opposite direction to increase experimental donations and anti-smoking attitudes in Bogotá. Future research should consider additional mediating constructs to better capture these behavior change processes, particularly in LMIC contexts. For example, our study’s qualitative findings identified contextual differences that influenced how the interventions operated. Pupils in Bogotá reported greater access to cigarettes through family members, peers, and unsafe neighborhood environments, and mentioned obtaining e-cigarettes through social media “giveaways.” By contrast, NI pupils reported more protective resources, such as prior school-based smoking education and anti-smoking advocacy through community groups (36). Given that smoking can become a strong, antagonistic habit reinforced by environmental cues, research on how these cues trigger initiation and habit formation, may be particularly relevant for smoking prevention and cessation strategies (134–138).

Cues in the physical environment and exposure to advertising are also closely linked to the political and regulatory context. It is therefore essential to consider differences in tobacco control environments when culturally adapting intervention programs (9). Colombia has recently introduced legislation regulating ENDS and other nicotine substitutes, including e-cigarettes (63), and the UK government has proposed similar measures extending tobacco control regulations to vaping and specific outdoor spaces (55, 56). These developments have made smoking, vaping, and related advertising increasingly prominent public issues. As tobacco control policies and societal smoking norms evolve, the behavioral mechanisms underlying peer influence in adolescent smoking may also change. It may be particularly important to monitor these processes in LMICs undergoing the initial stages of smoking denormalization, or in countries tightening their tobacco control measures (28, 29). The importance of social norms and social influences in adolescent smoking initiation is well documented (7, 11–13, 21). However, future research in this area should integrate social norms and social influence theories with broader behavior change frameworks that address intrapersonal, social, and environmental factors, and account for the wider cultural and political context (139, 140). Interventionists should also measure relevant mediators to capture these mechanisms and provide testable pathways of intervention effects.

### 4.3 Findings of suppressor mediation effects

In several mediation models comparing intervention mechanisms for ASSIST versus Dead Cool and NI versus Bogotá, we observed suppressor mediation effects, where specific mediators operated in the opposite direction to the total effect. In some cases, these patterns were partially explained by our measured variables, where we observed multiple mediators acting in opposing directions. For example, among ASSIST participants, decreased exposure to advertising in the media mediated an increase in experimental donations compared to Dead Cool, whereas an additional indirect pathway via perceived social risks favored increased donations for Dead Cool. A similar pattern emerged for anti-smoking attitudes, where decreased exposure to advertising in the media mediated higher anti-smoking attitudes for both the ASSIST PS and non-PS, but this operated counter to several other mediators—knowledge, descriptive smoking norms, perceived physical risks, and exposure to advertising in shops—which collectively mediated higher anti-smoking attitudes for Dead Cool. When comparing NI and Bogotá, we also identified mediators acting in opposite directions for self-report smoking behavior, with self-efficacy increasing anti-smoking behavior in NI and PBC that it would be easy to quit smoking increasing anti-smoking behavior in Bogotá.

On the other hand, some of the observed suppressor effects are likely attributable to omitted mediator variables. For example, intentions, attitudes, and opportunity self-efficacy all significantly mediated increases in self-report anti-smoking behavior in Dead Cool schools compared to ASSIST. However, the non-significant total effect of intervention on self-report smoking behavior suggests that additional, unmeasured mediators were acting in the opposite direction to enhance anti-smoking behavior in ASSIST schools. Consistent with this, we observed evidence that the ASSIST PS improved their self-report smoking behavior compared to Dead Cool. Although the diffusion of these benefits to the wider school cohort appears too limited to produce a detectable improvement for the ASSIST non-PS versus Dead Cool, it is probable that the ASSIST PS did exert some positive influence. This pattern suggests that the unmeasured mediators may relate specifically to the peer-led and diffusion-based nature of the ASSIST program (7, 37, 65, 66). Since the peer supporters’ conversations and social influence are central to ASSIST’s mechanism of change, future evaluations would benefit from incorporating measures that capture peer-led processes more directly, enabling clearer identification of the pathways through which peer supporters shape behavioral outcomes. Some specific examples of mediators that could be measured include peer-educator self-efficacy, perceived social support, and the frequency or reach of peer-led conversations.

In models with experimental donations to ASSIST/Dead Cool and attitudes towards smoking as outcomes, we observed suppressor effects which suggested the presence of unmeasured mediators operating in a protective direction in Bogotá, increasing donations and strengthening anti-smoking attitudes independently of the measured pathways. These patterns likely reflect broader cultural, normative, and structural influences that shaped how adolescents interpreted and responded to the interventions (141). For example, our qualitative findings highlighted several contextual differences: pupils in Bogotá reported greater access to cigarettes through family members, peers, unsafe neighborhood environments, and social media “giveaways,” whereas NI pupils described more protective resources, including prior school-based smoking education and community anti-smoking advocacy (36). These contextual factors may influence adolescents’ risk perception, moral evaluation of smoking, and responsiveness to interventions in ways not captured by the measured mediators. In Bogotá, intervention exposure may have activated additional processes—such as heightened collective concern about smoking-related harms, stronger moral or social disapproval of smoking, or increased critical awareness of tobacco industry practices—that reinforced anti-smoking attitudes but were not directly measured. These mechanisms are consistent with social-ecological and critical consciousness frameworks, which emphasize that health messaging in higher-risk or more unequal environments can catalyze protective attitudinal shifts through pathways beyond individual-level cognition (139, 140, 142–144). Differences in tobacco control environments may further shape these mechanisms, particularly as smoking and vaping becomes increasingly denormalized following recent ENDS regulations in both the UK and Colombia (9, 28, 29, 55, 56, 63). Future research should therefore integrate social norms theories with broader behavior change frameworks—incorporating intrapersonal, social, environmental, cultural, and political determinants—to more fully capture intervention mechanisms across sociocultural contexts, measuring mediators that account for these processes (139, 140). For example, studies could incorporate culturally-specific mediators capturing community-level norms, family communication patterns, neighborhood safety perceptions, informal access to tobacco products, perceived injustice or exploitation by tobacco companies, collective efficacy, and moral norm activation.

### 4.4 Sensitivity analyses comparing MECHANISMS schools with the Northern Ireland control group from the Dead Cool study

When comparing MECHANISMS schools with the NI control group from the Dead Cool RCT, most of the observed significant effects were more pro-smoking for MECHANISMS. It is important to note that there was a five-year gap between data collection for the NI control group (2014) and for MECHANISMS participants (2019). Although MECHANISMS schools were randomized to the ASSIST or Dead Cool interventions in NI and Bogotá, there was no randomization between MECHANISMS schools and the NI control group. As outlined in the Introduction, this means that differences between the two cohorts may reflect long-term national trends in smoking and vaping, short-term developmental changes that typically occur over a school semester, or both, rather than intervention effects.

Research shows that national trends in adolescent smoking and vaping behaviors can shift substantially over time (145). In NI, the proportion of current adolescent smokers decreased from 5.0% in 2013 to 3.9% in 2019, and further to 2.2% in 2022. Over the same period, the prevalence of adolescent current e-cigarette users increased from 4.9% in 2016 to 5.7% in 2019, and to 9.2% in 2022 (46). There has also been a notable shift in the pattern of use, with vaping increasingly acting as a precursor to cigarette smoking by 2019 (47). In Colombia, current cigarette consumption rates for adolescents aged 12–18 years decreased from 12.3% in 2011 to 7.6% in 2016, and to 4.5% in 2022 (31). Whilst national data suggests that adolescent smoking rates have generally declined in high-income countries, they have remained high or continued to rise in some LMICs (1, 145). Previous studies have also shown substantial variation in smoking behavior and intentions across adolescence (146–148). Adolescent smoking onset can increase rapidly, even over follow-ups shorter than one year (146). Our pre- and post-intervention measures were also collected during a developmental stage when contemplation and initiation of risk behaviors, including smoking, naturally increase as part of adolescence (146–149).

In contrast, exposure to tobacco advertising in shops was lower at follow-up (controlling for baseline) for ASSIST participants in NI, and for both ASSIST and Dead Cool participants in Bogotá, compared with the NI control group. This difference may be partly explained by changes in UK tobacco legislation between 2014, when the original Dead Cool study was conducted, and 2019, when MECHANISMS data collection occurred. For example, standardized tobacco packaging was only fully implemented in the UK in May 2017, and prior to the full display ban in April 2015, tobacco products could still be openly displayed in some types of shop (54, 150). Consequently, participants in the 2014 NI control group were probably more exposed to visible tobacco advertising and branding in retail environments than MECHANISMS participants in 2019. These comparisons therefore provide contextual information about how changes in outcomes over a school semester differed from an earlier cohort not exposed to either intervention, rather than evidence of intervention effects.

### 4.5 Strengths and limitations

Study strengths include the large sample size, and inclusion of data collected in two settings with different norms, culture, regulatory contexts, and health behavior patterns. All study materials were thoroughly culturally adapted and translated into Spanish language before implementing the programs in Bogotá (9). The study included a wide range of smoking and vaping-related outcomes to provide richer insights into the intervention mechanisms, including both self-report and objective measures of smoking behavior, and norms assessed using self-report and experimental methods. This is the first study to apply experimental methods from behavioral economics and game theory—which mitigate social desirability biases associated with self-report measures—to study norms for adolescent smoking and vaping (1, 5). The participation rates were high across MECHANISMS schools (93.1%), and completion rates for the experiments (93.1–94.6%) and survey (90.0–94.8%) were high at both timepoints. Therefore, the impact of missing data should be minimal, although our SEMs used FIML to address missing data.

All self-report mediators and behavioral outcomes were measured using previously validated instruments (1). To ensure conceptual equivalence across settings, all study instruments underwent a rigorous cultural adaptation process, including translation and back-translation into Spanish and pre-testing in pilot studies in both NI and Bogotá (9). Multiple-item scales were modelled as latent variables in SEMs, which offers advantages over approaches based on observed scale scores by accommodating statistical assumptions and adjusting for measurement error (151). Comprehensive measurement invariance CFA models demonstrated good construct and factorial validity, as well as longitudinal invariance and measurement equivalence across intervention groups and settings (5). These findings provide strong evidence that the instruments measured the same underlying constructs in both countries, supporting the validity of the cross-setting comparisons.

Our mediation analysis followed best practice. For example, intervention logic models and mediation analyses were pre-specified in the study protocol (1), mediator models were adjusted for baseline values, and bias-corrected bootstrap CIs were used to assess the significance of indirect effects (114, 115). We also estimated multiple mediator models, which allow specific indirect effects—through one mediator—to be calculated conditional on the presence of other mediators and reduce the risk of omitted variable bias (113). Since there were many potential mediators, we used the CMF method to select mediators for each outcome (117). The CMF method has shown improved performance over previous exploratory mediation approaches when many potential mediators are present (117). The multiple mediator models also included mediators with significant indirect effects in single mediator models (*p* < 0.05).

Some scholars have noted limitations of using mediation analysis to guide intervention adaptation or targeting. Our mediation models and SEMs estimate indirect and total effects based on population averages. In causal mediation terminology these correspond to *“interventional effects,”* which rely on population-level interventions and can be estimated within RCTs as population-average effects (152, 153). Thus, they overcome some of the limitations of *“natural effects,”* which are defined at the individual level and rely on empirically untestable “cross-world independence” assumptions (152, 153). Nevertheless, these average indirect and total effects may not apply uniformly across all individuals or subgroups. Recent methodological literature has therefore called for more complex designs that investigate effect heterogeneity, including mediated-moderation or moderated-mediation (moderation of the predictor-mediator or mediator-outcome paths by a third variable), baseline-target moderated-mediation (where intervention impact varies by baseline levels of the target variable), assessments of effect transportability and effect modifiers (related to external validity), and the “operating conditions” framework (investigating links between intervention mechanisms and moderators) (154–160). We did not incorporate a moderated-mediation approach in our models because of their complexity and the risk of model instability. Our primary aim was to investigate intervention mechanisms operating in school-based smoking prevention programs delivered to entire school year groups of adolescents. Moreover, the causal mediation literature highlights that whilst mediation implies mechanism, mechanism does not necessarily imply mediation (161). Therefore, some of our targets or mediators may have influenced the smoking outcomes through non-mediational pathways.

The MECHANISMS study presented distinct implementation challenges inherent in conducting a complex, multidisciplinary project across two culturally and structurally diverse research settings. Our study teams were required to balance appropriate cultural adaptation of the study tools and intervention materials in line with local norms, school structures, and Colombian tobacco control regulations, while maintaining fidelity to each program’s core components and ensuring standardization of research procedures across settings. A rigorous cultural adaptation process was undertaken before implementation in Bogotá, and findings from the adaptation work, pilot phases, and fidelity monitoring indicated that the core content and themes of both interventions were delivered as intended in each setting (9). This supports the interpretation that the cross-setting differences observed in this paper are more likely to reflect contextual influences and intervention mechanisms rather than inconsistencies in delivery. Standardization of study procedures was facilitated through quarterly study meetings, regular online coordination, and research visits. As expected, given the contrasting settings, significant baseline differences were observed across many outcomes and participant characteristics. Covariate selection in our models was guided systematically using DAGs (92, 93), and our models were adjusted for established correlates of adolescent smoking—gender, age, ethnicity, SES, and baseline values (35, 162–169). However, we acknowledge that unmeasured or residual confounding may remain (170, 171).

Some scholars may reasonably question the appropriateness of comparing outcomes or intervention mechanisms across settings that differ markedly in their structural, cultural, and demographic characteristics. In our analyses comparing intervention mechanisms between NI and Bogotá, setting is explicitly conceptualized as the primary exposure of interest. In this context, “setting” represents a composite of contextual, demographic, cultural, and structural characteristics rather than a single manipulable factor, and we do not assume that the two settings are inherently comparable. Similar studies have used standard SEM and regression-based approaches to make comparisons across small numbers of cultural or national settings, including previous MECHANISMS publications (32, 34, 35, 172–177). Adjustments for gender, age, ethnicity, and SES were undertaken to reduce confounding due to compositional differences between samples, allowing the estimated direct and indirect effects to more clearly reflect contextual influences beyond these core individual-level characteristics. In our models examining intervention effects, setting was additionally adjusted for using fixed effects, which accounts for all time-invariant between-setting differences. This approach is recommended in small-cluster contexts where multilevel random-effects models are not statistically appropriate as the variance components cannot be reliably estimated (178, 179). We acknowledge the potential for residual confounding by unmeasured contextual characteristics, such as broader policy environments, educational system structures, or cultural norms not captured by the measured mediators. Accordingly, our mediation estimates should be interpreted as explanatory rather than strictly causal, and the findings should be viewed as context-informed insights into mechanisms underlying the cross-setting differences in outcomes.

The MECHANISMS study was designed to be mechanisms-focused rather than offering a randomized head-to-head comparison of the effectiveness of ASSIST and Dead Cool, which were both previously evaluated in the UK (7, 8). The primary aim was to understand how adolescent smoking prevention interventions work, for whom, and why, using a theory-based rather than an effectiveness-focused perspective. While prior research has examined the effectiveness of adolescent smoking prevention interventions grounded in diverse behavior change theories, evaluations which are focused only on effectiveness are insufficient to inform decisions about implementation across varied contexts (180). In contrast, theory-driven evaluation emphasizes understanding how interventions operate, including the mechanisms through which they influence individuals and settings (181). Despite this, the mechanisms by which these interventions produce behavioral change across contexts remain poorly understood, and pathways involving social networks and social norms are under-investigated (182, 183). Mechanisms-focused studies such as ours, which aim to address this gap, are essential for informing real-world public health implementation and decision-making across diverse settings (180).

Our study’s experiment assessed injunctive and descriptive norms for both smoking and vaping, and our main models included latent variables that combined these norms. A potential criticism of this approach is that combining smoking and vaping norms could obscure intervention mechanisms, given evidence that e-cigarette norms and peer dynamics can differ substantially from those associated with traditional smoking. For example, research shows that young people hold more positive norms around e-cigarettes than conventional cigarettes, often perceiving them as more socially acceptable, less stigmatized, and less harmful (184–187). In the MECHANISMS study, we considered it important to measure norms related to both smoking and vaping, particularly in light of the increasing popularity of e-cigarettes among adolescents (17, 18). For adolescents, smoking and vaping are highly interconnected behaviors. Whilst adults more often use e-cigarettes as a smoking cessation aid, adolescents tend to experiment with e-cigarettes in similar ways to traditional cigarettes. Young people who use e-cigarettes are also more likely to start smoking (17, 19, 20). When we re-estimated our SEMs separately for smoking-specific and vaping-specific norms, the results were consistent with the combined models, supporting the validity of our approach to modelling smoking and vaping norms together.

The MECHANISMS study was funded as a proof-of-concept study to compare norms-based mechanisms in the ASSIST and Dead Cool programs and did not include a control group in its design (1). Although we incorporated control group data from the original Dead Cool study to provide additional context in a sensitivity analysis, schools were not randomized between the MECHANISMS interventions and the NI control group. This means the historical control data cannot be interpreted as a true counterfactual. In addition, there was a five-year gap between data collection for the NI control group (2014) and the MECHANISMS cohort (2019), during which population-level adolescent smoking and e-cigarette use patterns changed substantially. These temporal and design differences introduce the possibility of bias due to secular trends and unmeasured confounding. As such, the historical control data should be viewed as offering a broad benchmark for contextualizing short-term changes in outcomes rather than providing a basis for causal inference. Nonetheless, for outcomes measured identically across studies, the 2014 cohort allowed us to explore whether the short-term, within-cohort changes observed in MECHANISMS were broadly consistent with expected background patterns among similarly aged pupils who were not exposed to either intervention.

Our study has several other limitations. The MECHANISMS study included a relatively small sample of schools. Therefore, we are cautious in generalizing our findings to other schools in NI and Bogotá. However, we endeavored to recruit schools with a range of deprivation levels and mixed gender. Our results should be interpreted with caution due to multiple testing. However, no consensus exists on adjustment for multiple testing, and several prominent researchers have argued that adjustment is not always desirable or appropriate (188–190). In our results tables, we have highlighted which results would have remained significant at *p* < 0.05 after applying Holm-Bonferroni corrections (87). We did not cross-validate our CFA models using an independent sample. Due to the complexity of our models, we were reluctant to decrease power by reducing the sample size. Two of our latent variables were measured with only two indicators as our study’s assessment of experimentally measured descriptive smoking/vaping norms and injunctive vaping norms included two items each. The MECHANISMS study was not specifically powered to detect changes in outcomes between baseline and follow-up, differences between the intervention groups or settings, or mediated effects (1). Post-hoc power analyses indicated that power for some analyses was low.

### 4.6 Implications for future research

The eight-week multi-component Dead Cool program—delivered to all pupils within classrooms and comprising weekly individual and group-based exercises—was effective in targeting adolescents’ smoking-related outcomes via the hypothesized mediating constructs. Whilst the ASSIST program improved knowledge about smoking, self-report smoking behavior, and perceived addiction risks among the ASSIST PS who attended the training course, these improvements were not transmitted to the wider school year groups. Intervention studies based on peer education and diffusion should explore strategies to improve and evaluate the effectiveness of the intervention communication channels—specifically, the informal peer-to-peer conversations within school year groups—because they are crucial to the diffusion process. One promising strategy is the use of prompts and cues to support peer supporters in initiating conversations. Social prompts could include daily text messages or emails from ASSIST trainers reminding peer supporters to engage in discussions about smoking. Environmental prompts might involve displaying posters featuring key facts learned during the training within school spaces. Evidence suggests that such prompting strategies can successfully promote healthy behaviors and engagement in interventions (125–127). Future adaptations might also use alternative social network parameters to identify the most influential pupils within friendship networks as peer supporters (35), or increase the proportion of pupils recruited. Future peer-led intervention studies should also investigate mediators that explain the effectiveness of peer supporters in influencing their peers’ attitudes and behaviors.

Our findings support using social norms intervention strategies in LMICs. Tobacco education is not yet formally integrated in Colombia’s school curriculum. More widespread implementation of school-based programs may be an appealing and effective way to target adolescent smoking prevention in LMICs and should be considered in ongoing tobacco control efforts (22, 24, 133). Whilst WTP to support anti-smoking norms, self-report descriptive norms and exposure to advertising in shops were more anti-smoking in Bogotá, most other targeted mediators and outcomes were more anti-smoking in NI at follow-up. Several of our multiple mediator models also showed suppressive mediation effects, which could indicate the presence of unmeasured mediators acting to increase WTP and anti-smoking attitudes in Bogotá. Future research should consider including additional mediating constructs that can both describe and drive behavior change during smoking prevention interventions, particularly in LMIC contexts. Behavior change theories that integrate factors across intrapersonal, social and environmental levels—whilst accounting for the wider cultural and political context—should be explored in combination with social norms and social influence theories when designing adolescent smoking prevention interventions (139, 140). Researchers should also measure relevant mediators to capture these mechanisms empirically and enable formal testing of theoretical pathways.

Research on environmental cues and how they trigger habit formation, may be particularly relevant for smoking prevention and cessation (134–138). Cues in the physical environment and exposure to tobacco advertising are closely linked to the political and regulatory context of each setting. Both the UK and Colombia have recently introduced, or are in the process of introducing, more stringent tobacco and e-cigarette control legislation, making smoking, vaping, and advertising highly prominent topics in the media and public discourse. Peer processes, such as social influence and diffusion of norms, may evolve as national tobacco control policies, public attitudes, and societal smoking norms change. Monitoring these dynamics will be especially important in LMICs that are newly implementing or tightening tobacco control measures (28, 29).

## 5 Conclusion

This study compared intervention mechanisms between two school-based smoking prevention programs—ASSIST and Dead Cool—implemented with adolescents in NI and Bogotá as part of the MECHANISMS study (1). Using multiple mediator models, we examined the hypothesized pathways through which each intervention influenced smoking-related outcomes. The eight-week, multi-component, skills-based Dead Cool program, delivered to all pupils in classroom settings, was effective in targeting adolescents’ smoking-related outcomes through the hypothesized mediating constructs. The ASSIST program, which operates through peer education and diffusion, improved knowledge about smoking, self-report smoking behavior, and perceived addiction risks among the peer supporters who attended the training course compared with Dead Cool. However, these improvements did not appear to be effectively diffused to the wider school year groups through informal peer conversations, suggesting limited transmission of intervention effects. Future research should investigate how to optimize and evaluate the communication channels that determine the effectiveness of peer education and diffusion programs (e.g., the conversations between ASSIST peer supporters and their school friends about smoking).

Whilst pupils’ WTP to support anti-smoking norms, self-report descriptive norms, and exposure to advertising in shops were more anti-smoking at follow-up (controlling for baseline) in Bogotá compared with NI, most other targeted mediators and outcomes were more anti-smoking in NI. Our findings reinforce the potential of social norms–based strategies for smoking prevention in LMICs. Smoking prevention education is not yet formally integrated in the school curriculum in Colombia and many other LMICs. More widespread implementation of school-based programs may be an appealing and effective way to target adolescent smoking prevention and should be considered in ongoing tobacco control efforts.

Several significant mediators demonstrated suppressive effects—acting in the opposite direction to the total effect of setting or intervention group on the outcome—suggesting the presence of unmeasured mediating mechanisms. Future research should consider additional mediating constructs which may be effective targets for smoking prevention, particularly in LMICs. Integrating theories of social influence and social norms with behavior change theories that span intrapersonal, social, and environmental factors—while accounting for cultural and political contexts—may enhance intervention design and effectiveness. As tobacco control policies and societal smoking norms continue to evolve, it will be essential to monitor how the norms-based mechanisms of behavior change—and thus the most relevant and effective prevention strategies—shift over time, particularly in LMICs that are introducing comprehensive tobacco control measures for the first time.

## Data Availability

The datasets presented in this article are not readily available because participants were informed that no-one outside of the research team would have access to the research data when they signed their consent forms. Examples of the syntax used to generate the results have been provided in the Supplementary Materials. For further information about the study datasets and analytic code, please contact the corresponding authors. Requests to access the datasets should be directed to JM (jmurray39@qub.ac.uk) or RH (ruth.hunter@qub.ac.uk).

## Footnotes

1 The self-efficacy opportunity subscale was kept in separate single mediator models as there were convergence issues when it was combined with other mediators.

2 The “proportion mediated” is calculated as the ratio of each specific indirect effect to the total effect. Negative values indicate suppressor mediation, where the mediator operates in the opposite direction to the total effect. Large negative values (e.g., < −100%) can occur when the total effect is very small or non-significant, reflecting opposing mediating pathways that inflate the negative indirect effect relative to the total effect. Large positive values (e.g., >100%) also indicate suppression is present. In this case, the mediator acts in the same direction as the total effect, but other (measured or unmeasured) mediators act in the opposite direction, reducing the total effect to its observed magnitude.
