# Supplementary material for: Mediation analysis investigating the mechanisms of two school-based smoking prevention interventions in adolescents from Northern Ireland and Bogotá
Source: Front Public Health. 2026 Mar 17;14:1758481. doi: 10.3389/fpubh.2026.1758481 (PMC13036153; doi:10.3389/fpubh.2026.1758481)
Supplement: Supplementary file 1 [file Data_Sheet_1.docx]

Supplementary Material

Mediation analysis investigating the mechanisms of two school-based smoking prevention interventions in adolescents from Northern Ireland and Bogotá.

**Jennifer M. Murray*, Sharon C. Sánchez-Franco, Olga L. Sarmiento, Erik O. Kimbrough, Christopher Tate, Shannon C. Montgomery, Rajnish Kumar, Laura Dunne, Allen Thurston, Aideen Gildea, Abhijit Ramalingam, Erin L. Krupka, Felipe Montes, Huiyu Zhou, Laurence Moore, Linda Bauld, Blanca Llorente, Frank Kee, Ruth F. Hunter***

*** Correspondence:** Corresponding Authors: [jmurray39@qub.ac.uk](mailto:jmurray39@qub.ac.uk), [ruth.hunter@qub.ac.uk](mailto:ruth.hunter@qub.ac.uk)

**This file includes:**

Supplementary Methods.

Supplementary Figures S1 to S26.

Supplementary Tables S1 to S77.

The information in the Supplementary Methods has been previously published as supplementary information to earlier papers related to the MECHANISMS study (1–3).

**
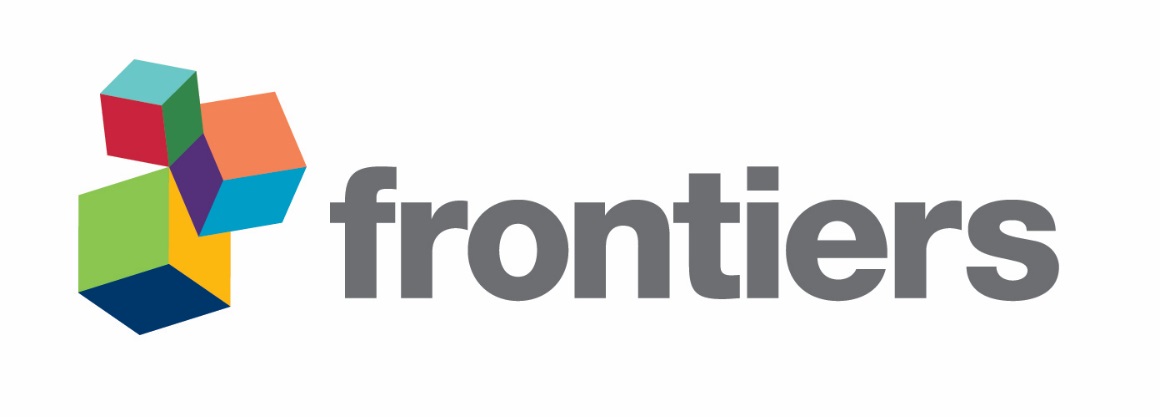
**

# Supplementary Methods

## MECHANISMS study procedures.

**Sample size calculation:** The study's power calculation was drawn from the work of Krupka et al., (2016) who studied ~200 Michigan university freshmen before and after a single semester (4). Using the basic Clauset community detection algorithm, with a sample size of ~200, and with data from two waves, a one standard deviation (SD) increase in risk preferences of an individual’s friends or social network was associated with an increase of 1/8th to 1/10th of a standard deviation (SD) of the same variable for the individual (5). Assuming that clustering of risk preferences is a reasonable proxy for clustering of social norms related to smoking that we have elicited in the MECHANISMS study, the power calculation estimated that a sample size of 300 would give over 80% power to detect, as statistically significant at the 5% level, a slope of 0.16 (i.e., an increase of 0.16 SDs in “y” [individual norms sensitivity], per SD increase in “x” [norms sensitivity of those in the individual’s social network]).

**School recruitment and assignment to intervention programs:** In Northern Ireland (NI), schools were recruited for the full phase of the MECHANISMS study between November 2018 and January 2019. Schools were recruited for the "A Stop Smoking in Schools Trial" (ASSIST) program as follows. Non-selective post-primary schools were selected from the list of secondary schools in NI as outlined by the Department of Education NI (6). Schools were prioritized if they were non-selective secondary education schools, mixed gender, had over 100 pupils in the year group, were of higher deprivation levels, and ranged in geographical location (urban, rural) and sector (controlled, maintained, integrated). Schools with over 100 pupils in Year 9 were identified using Year 8 enrolments during 2017 (7). Deprivation was measured using the Northern Ireland Multiple Deprivation Measure 2017 (NIMDM2017) (8). Principal invitations outlining the study procedures were sent to nine schools who met the criteria outlined above. Follow-up emails and phone-calls were conducted by a member of the study team, and a total of three schools were recruited to the ASSIST arm.

Schools were recruited for the Dead Cool program in NI as follows. Schools were prioritized if they were in more deprived areas and had higher school enrolment numbers. The Cancer Focus NI (CFNI) Post Primary School database was searched, and schools were eliminated if they were currently enrolled in the Dead Cool program or other CFNI smoking prevention programs. The remaining schools were contacted for expression of interest. After initial contact with the school was established, schools were eliminated if they had a class duration of less than 50 minutes (as this was deemed unsuitable for satisfactory completion of the Dead Cool program). The remaining schools were classified to ensure a good mix in terms of gender, geographical location (urban, rural) and sector (controlled, maintained, integrated). Detailed information regarding study procedures was sent to each school, resulting in a number of schools deselecting due to a range of issues (e.g., change of staff due to illness, unannounced school inspection, commitment to other initiatives). A total of three schools were enrolled to the Dead Cool arm of the study. The remaining schools were offered the Dead Cool program without taking part in the study.

In Bogotá, schools were recruited for the full phase of the MECHANISMS study between March and May 2019. The school recruitment strategy was as follows. First, a list of 40 private and public schools were prioritized based on health risks outlined by the Education and Health secretaries. Second, from this list, 13 schools were invited to participate according to the following inclusion criteria: (1) schools in urban area; (2) including boys and girls; (3) having enrolled between 90 and 150 students in 7th year (equivalent of Year 9 in NI). Third, only six schools accepted the invitation and were selected for the final sample. These schools were assigned randomly to each of the intervention arms by a member of the study team. Three schools were assigned to the ASSIST program, and the other three were assigned to the Dead Cool program.

**Ethical approval and study procedures:** Prior to the baseline assessment, each school was provided with teacher information sheets, pupil information sheets, parent/guardian information sheets, pupil consent forms, and parent/guardian opt-out forms. All pupils were required to complete written consent forms indicating whether they agreed or declined to participate. Parents/guardians who did not wish their child to take part were asked to return completed opt-out forms. In Bogotá, a parental opt-in procedure was used with written informed consent collected from parents/guardians at the start of the study. The consent procedures were fully approved by the ethics committees at the respective institutions. Pupils who consented to participate took part in a baseline assessment consisting of a series of game theory experiments and completion of a self-report survey. The fieldwork was conducted in NI schools between January and June 2019, and in Bogotá's schools between July and November 2019. Following the baseline assessment, each school took part in one of two previously tested smoking prevention interventions: ASSIST (N=3 schools in NI, N=3 Bogotá) or Dead Cool (N=3 NI, N=3 Bogotá) over a single school semester (approximately 10 weeks). Following intervention delivery in each school, all participants took part in a follow-up assessment, again completing the game theory experiments and a self-report survey. Participation in the study's experiments required a monetary payment to be made to each individual pupil. In NI the payment was made in cash, however due to Colombian ethical regulations the payment was made using gift cards to pupils in Bogotá. Ethics approval was granted from the School of Medicine, Dentistry and Biomedical Sciences Ethics Committee at Queen's University Belfast (QUB) on September 21, 2018 (reference 18:43) and from the Research Ethics Committee at Universidad de los Andes (UniAndes) on July 30, 2018 (reference 937/2018).

The study was implemented in Bogotá following a thorough cultural adaptation process, which involved translation and back‑translation of all study materials, intervention content, surveys, experiments, information sheets, and consent forms into Spanish. Content and delivery of these tools were adapted when needed according to the requirements of the local Research Ethics Committee and local legislation regarding research involving human subjects. For example, in Bogotá, information sheets were delivered to parents, teachers, and school administrators in personal meetings instead of by e-mail. Also, questions from parents and participants were handled in multiple presential meetings, before informed consent was collected. Parental opt-in consent was collected in Bogotá, whilst a parental opt-out consent procedure was used in NI. Game theory earnings were paid using gift cards instead of cash in Bogotá. As part of the cultural adaptation of the intervention programs, any images suggesting indirect promotion of tobacco consumption and substance-specific content were removed or replaced to fit with the national guidelines to prevent iatrogenic outcomes (9). These procedures were fully approved by the ethical review boards at the respective institutions. All instruments were pre-tested in a pilot study conducted before the full phase of the MECHANISMS study, which included a sample of 239 in Bogotá and 73 in Northern Ireland. Full details on the MECHANISMS study's cultural adaptation process and results can be found in our published cultural adaptation paper (10).

**Data collection:** The baseline assessment consisted of two separate sessions, held approximately one week apart, with each class in the school year group in each school. Sessions lasted approximately 50 minutes. Participating pupils completed the game theory experiments during the first session and the self-report survey during the second session. Experiments and surveys were collected on tablet computers using the platform Qualtrics (web-based platform in NI and offline version in Bogotá) (Qualtrics, Provo, Utah, USA). At the start of each session, participants were assured that any information provided would be treated as confidential. They were also instructed not to communicate with other participants and to direct any questions to a researcher. In NI, poster boards were used at computer stations to discourage communication between participants. In both countries, instructions were delivered onscreen with key portions read aloud by the experimenter. The experimenter read out introductory instructions at the start of the experiment, and at the start of Parts 1, 2 and 4. Pupils were invited to ask any questions. Dummy screens were inserted at the end of Parts 1 and 3, instructing pupils to wait until all of their classmates were ready to proceed to the next part so that instructions could be read together. Parts 2 and 3 were otherwise self-paced, and pupils were invited to raise their hands to have any further questions answered privately. The same procedures were used during the follow-up assessment.

**Interventions:** The 'A Stop Smoking in Schools Trial' (ASSIST) and Dead Cool programs have previously been evaluated in separate cluster randomized trials, and shown to effectively reduce rates of smoking initiation amongst adolescents (11,12). ASSIST is specifically designed to leverage peer influence whilst Dead Cool is based on conventional classroom pedagogy. The ASSIST program is based on the diffusion of innovations theory (13), and works on principles of peer education and diffusion. The ASSIST intervention is designed to train the most influential pupils in the school year group, nominated in a peer questionnaire completed by all participants prior to the baseline assessment, to use informal contacts with their peers (i.e., other pupils in their school year group) to encourage them not to smoke (11). Dead Cool is a skills-based program based on the theory of planned behavior (14). It includes training of schoolteachers in program delivery and provision of program resources (lesson plans, pupil work books, fact sheets and a DVD) to enhance pupils' knowledge of potential influences on smoking behavior from family, friends and the media (12,15).

**Participant and public involvement:** MECHANISMS study participants were first involved in the research during the baseline assessment (16). This paper uses participant data. Focus groups and interviews conducted with pupils and teachers during the Bogotá pilot were used to guide the cultural adaptation (10). Teachers and pupils provided information that was useful for addressing issues around adapting the intervention programs to the needs and context of the Colombian pupils (10). Otherwise, research participants and the public were not involved in deciding the research questions or outcome measures, or in the design, conduct, or reporting of the research. Research participants were involved in an online dissemination workshop in March 2021 during which they shared their experiences of taking part and their views on smoking-related issues. Research participants and their schoolteachers were also invited to attend this workshop to listen to researchers' talks disseminating the results of the study. Focus groups were conducted in each of the schools during which groups of participants shared their experiences of taking part in the smoking prevention programs, and completing the experiments, surveys and carbon monoxide measurements (17). Research participants were also involved in creating online videos to disseminate the research for "World No-Tobacco Day 2020" (18).

## Game theory and behavioral economics experiments.

The game theory experiments consisted of a series of incentivized tasks which were based on published works in the field of behavioral economics (19–21), and designed by the original producers (Kimbrough, Krupka) and other experts in the field (Kumar, Ramalingam). There were four parts to the experiment: (1) a Rule-Following (RF) task measuring each individual participant's sensitivity to the effects of social norms; (2) a series of co-ordination games attempting to elicit *injunctive* social norms unrelated and related to smoking and vaping behaviors; (3) a series of co-ordination games attempting to elicit *descriptive* social norms related to smoking and vaping behaviors; (4) a willingness-to-pay task designed to measure each individual participant's support for cultivating anti-smoking norms. These are outlined in more detail below. The full experimental protocol is available below.

At the start of experimental sessions, participants were informed that they would receive a participation fee of £5.00 (NI; *COP* $5.000 in Bogotá), and that they could earn money in each part of the experiment (maximum £30 in NI, *COP* $50.000 in Bogotá) depending on the answers they provided and those provided by other pupils in their school year group. They were told that the researchers would determine their payment by performing two sets of randomizations for each part of the experiment: (1) to determine whether payment was based on answers provided at baseline or follow-up; (2) to determine which question of each part would result in payment.

**Part 1: Identifying individual-level norms sensitivity.**

Part 1 of the experiment consisted of an individual decision task (a variant of the RF Task) measuring participants' preferences for following established rules and social norms, without peer interaction (19,20). The task instructs participants to follow an explicitly stated arbitrary rule when doing so imposes explicit monetary costs directly proportional to the degree of rule-following. We employed the version of the RF task introduced by Kimbrough and Vostroknutov (2018) (20). Specifically, participants were asked to sequentially allocate 50 balls across two buckets (one blue and one yellow). They were told that "The rule is to put the balls in the blue bucket". They were also informed that they would receive £0.05 (NI; *COP* $100 Bogotá) for every ball they put in the blue bucket and £0.10 (NI; *COP* $200 Bogotá) for every ball they put in the yellow bucket. Lastly, they were informed that they would be given five minutes to allocate the 50 balls between the two buckets and that any balls which were not allocated by the end of the five minutes were worth nothing.[[1]](#footnote-1) No other information was provided. Therefore, assuming a participant allocated all 50 balls, the minimum amount that he/she could earn was £2.50 (NI; *COP* $5.000 Bogotá) if he/she followed the rule completely and allocated all 50 balls to the blue bucket. The maximum amount that could be earned was £5.00 (NI; *COP* $10.000 in Bogotá) if he/she completely ignored the rule and allocated all 50 balls to the yellow bucket. The central premise is that the more a participant cares intrinsically about rule-following the more willing he/she will be to incur the costs of doing so (19). The extent of rule-following in the RF task provides a measure of individual norm-following proclivity, and this norm sensitivity measure has been shown to correlate with willingness to follow norms of cooperation, reciprocity and prosocial behavior across decision contexts (19). To avoid introducing any potential biases due to preference for bucket placement, participants were randomized to a version of the RF task with the blue bucket on the left (n=621 baseline, n=616 follow-up), or a version with the blue bucket on the right (n=650 baseline, n=635 follow-up).

**Parts 2-3: Measuring injunctive and descriptive social norms.**

Parts 2 and 3 of the experiment consisted of a series of incentivized co-ordination games which used methods employed by Krupka and Weber to elicit injunctive and descriptive social norms around smoking and vaping (21). Injunctive norms reflect shared beliefs among members of a population about what actions people *ought* *to* take; descriptive norms reflect shared beliefs among members of a population about what actions people *actually do* take (21).

In Part 2, participants were asked to rate the social appropriateness of various actions that others might take on a six-point Likert scale: "extremely socially inappropriate", "very socially inappropriate", "somewhat socially inappropriate", "somewhat socially appropriate", "very socially appropriate", "extremely socially appropriate". Situation 1 aimed to elicit pro-sociality injunctive norms by asking participants to co-ordinate with others in their school year group to rate the social appropriateness of a range of actions one might take in a standard dictator game. The dictator game is commonly used as a measure of social preferences, in particular, altruism. Such norms are unlikely to be affected by interventions targeted at altering smoking behavior. Eight items (situations 2-9) were asked to assess smoking- and vaping-related injunctive social norms. In Part 3, participants were asked to estimate the proportion of peers in their school year group who would be accepting of certain behaviors on a six-point Likert scale: "none of my peers", "only a few of my peers", "some of my peers", "a lot of my peers", "most of my peers", "all of my peers". Two items were asked to assess smoking- and vaping-related descriptive social norms.

The principal feature of this part of the experiment is that participants are provided with incentives to *match* their ratings/estimates to other participants' in their school year group on the day as opposed to providing personal opinions. For example, participants are informed that they will receive £10 (NI; *COP* $15.000 Bogotá) if the answer they provide for a randomly selected question matches the most common answer in the school year group. Assuming that a norm exists, and in the absence of peer interaction, participants attempting to match others' responses in order to win the incentive will anticipate the extent to which others will rate an action as socially appropriate or inappropriate (or anticipate the extent to which others will estimate that a large or small proportion of their peers would be accepting of certain behaviors), and respond accordingly.[[2]](#footnote-2) Therefore, in Part 2 of the experiment participants play a co-ordination game in which the incentive elicits an empirical measure of injunctive social norms as collective perceptions of the social appropriateness of various behaviors. In Part 3 they play a co-ordination game in which the incentive elicits an empirical measure of descriptive social norms as collective perceptions of the rate of acceptance of certain behaviors.

As proposed by the original authors, the components elicited in Parts 1-3 of the experiment can be examined within the context of a norm-dependent utility framework to further our understanding of how the existence of social norms, and individuals' norms sensitivities, can influence behavior in social settings (19–21). Within this framework, behavioral heterogeneity in a given social context is proposed as being related to the fact that people suffer disutility from violating norms and that those individuals differ in sensitivity to own-norm violations.

u(a_k_) = V {ᴫ(a_k_)} + γN(a_k_) (1)

In the above framework (1), social norms are modelled quantitatively, such that a decision maker’s "pay-off", u(a_k_), from each action, V {ᴫ(a_k_)}, is traded off against the normative appropriateness of each action according to the parameter γ≥0, representing the degree to which the individual cares about adhering to social norms, with the function N capturing the social norm. N_g_(a_k_) denotes the social norms for group g, which are estimated from the co-ordination games in Parts 2 and 3 of the experiment. γ is the parameter reflecting individual sensitivity to the norm, estimated using the total number of balls allocated to the blue "rule-following" bucket in the RF task of Part 1.

**Part 4: Measuring willingness to pay to support anti-smoking norms.**

Part 4 of the experiment gives us a chance to test the implications of this model. Participants were given an endowment of £5 (NI; *COP* $10.000 Bogotá) and asked to decide how much of the £5 they wanted to donate to the organizations responsible for ASSIST/Dead Cool, depending on which program their school was taking part in, and how much they wanted to keep for themselves. They were provided with a description of ASSIST/Dead Cool as "a smoking prevention program which aims to prevent the uptake of smoking among adolescents your age". Therefore, in the same way that a willingness to incur a cost to follow the rule in the RF task reveals a respect for following norms in general, the extent of a participant's willingness to incur a cost to make a higher donation to a program whose aim is to encourage smoking reduction by others reveals their support for creating anti-smoking norms. Since a donation may be taken as revealing a participant's belief that such smoking prevention programs are normatively appealing and effective, this task provides evidence for the behavioral impact of an injunctive anti-smoking social norm.

To connect this to the model (1), we need only assume that V is increasing in the participant’s own payoff; then when facing the decision about whether to donate to the anti-smoking intervention charity, subjects trade off their own higher payoff from keeping the money for themselves against the normative appropriateness of donating to help prevent smoking. The theory implies that when norms are stronger, or an individual’s γ is larger, the amount donated will be higher.

Further details on the smoking- and vaping-related scenarios assessed in Parts 2 and 3 of the experiment and numerical coding of responses are provided in Supplementary Table S3. Responses to the experiment items from Part 2 were coded such that numerical responses ran between -1 (extremely socially inappropriate) and +1 (extremely socially appropriate) following procedures adopted in Krupka and Weber (2013) (21). Similarly, responses to experiment items from Part 3 were coded such that numerical responses ran between -1 (none of my peers) and +1 (all of my peers).

## Statistical analysis: Latent variable modelling and measurement invariance confirmatory factor analyses.

In structural equation models (SEMs), the following outcomes and mediators were treated as latent variables (i.e., a variable that is not directly observed but is rather inferred, through a mathematical model, from other variables or "indicators" that can be directly observed or measured (22)): attitudes towards smoking (measured from items 'AT1', 'AT3' to 'AT12', item 'AT2' was excluded due to poor factor loading), experimentally measured injunctive smoking/vaping norms ('P2S2' to 'P2S9'), experimentally measured injunctive smoking norms ('P2S2' to 'P2S6' and 'P2S9'), experimentally measured injunctive vaping norms ('P2S7' to 'P2S8'), experimentally measured descriptive smoking/vaping norms ('P3Q1' to 'P3Q2'), self-report injunctive smoking norms ('IN1' to 'IN7'), self-report descriptive smoking norms scale 1 ('DN1.1' to 'DN1.5'), self-report descriptive smoking norms scale 2 ('DN2.1' to 'DN2.3'), self-efficacy (emotional; 'SEE1' to 'SEE9'), self-efficacy (friends; 'SEF1' to 'SEF9'), self-efficacy (opportunity; 'SEO1' to 'SEO11'), perceived physical risks ('RP1' to 'RP7'), perceived social risks ('RS1' to 'RS3'), perceived addiction risks ('RA1' to 'RA3'), and perceived benefits ('BE1' to 'BE5'). The following outcomes were treated as observed variables in SEMs: experimental donations to ASSIST/Dead Cool, self-report smoking behavior, objectively measured smoking behavior, smoking intentions, susceptibility, knowledge, perceived behavioral control (easy to quit smoking), perceived behavioral control (to avoid smoking), exposure to advertising in the media, and exposure to advertising in shops (Supplementary Table S3).

Measurement invariance across time (i.e., between baseline and follow-up) for the latent variables was examined with longitudinal measurement invariance confirmatory factor analysis (CFA) models, using procedures described in Mackinnon et al., (2022) (23). CFAs were specified with the 'lavaan' package in R (24), using maximum likelihood estimation with robust (Huber-White) standard errors (SEs; MLR estimator), and imputation of missing data using full information maximum likelihood (FIML) (25,26). CFAs were constructed examining configural (i.e., whether the constructs have the same pattern of free and fixed loadings across timepoints), weak or metric (i.e., whether the item loadings on the factors are equivalent across timepoints, meaning that the latent factor has the same interpretation), strong or scalar (i.e., whether the item intercepts are equivalent across timepoints, meaning that latent means can be compared), and strict or residual (i.e., whether the item residuals are equivalent across timepoints) measurement invariance for each latent variable (27). Latent variables were scaled by freeing the first indicator (in the configural model) and constraining the variance of each latent variable to 1[[3]](#footnote-3), which makes it easier to constrain the factor loadings to equality in the later models (23). For all models, unstandardized ("std.lv", which standardizes all latent variables in the model only) and standardized ("std.all", which standardizes all latent and observed variables in the model) parameter estimates were extracted.

Goodness-of-fit statistics were extracted for each model, including: the model chi-square test statistic, the Comparative Fit Index (CFI), the Tucker-Lewis Index (TLI), the Root Mean Square Error of Approximation (RMSEA), the Standardized Root Mean Square Residual (SRMR), and three parsimony based fit indices (the Akaike Information Criterion [AIC], the Bayesian Information Criterion [BIC], and the adjusted BIC). For the model chi-square test, p>0.05 indicates good model fit, but the test can be overly influenced by sample size, correlations, variance unrelated to the model, and multivariate non-normality, and so models were not rejected based on the chi-square statistic (28,29). CFI values of ≥0.96, TLI values of ≥0.95, RMSEA values of ≤0.06, and SRMR values of ≤0.08 indicated good fit (28,29). Our cutoff values for 'good fit' were mostly based on the strict criteria presented in Hu and Bentler (1999) and Hooper et al., (2008) (28,29). We also determined that CFI and TLI values of ≥0.90, RMSEA values of ≤0.08, and SRMR values of ≤0.09 indicated acceptable fit (23,28). For example, the two-index presentation strategy presented in Hu and Bentler (1999) and Hooper et al., (2008) suggests that SRMR values of ≤0.09 are acceptable when combined with RMSEA values of ≤0.06 or CFI values of ≥0.96 (28,29). Lower values of the AIC, BIC, and adjusted BIC indicate a more parsimonious model, with differences of ≥6 indicating strong evidence of model difference (23,30).

Measurement invariance was examined with three sets of comparisons: (1) metric (weak) versus configural CFA models; (2) scalar (strong) versus metric CFA models, and; (3) residual (strict) versus scalar CFA models. Scaled chi-square difference tests (i.e., log-likelihood ratio tests) were calculated using the Satorra and Bentler (2001) method (31). The CFI, TLI, RMSEA, SRMR, AIC, BIC, and adjusted BIC were compared by calculating the differences in the indices between the CFA models. Non-significant scaled chi-square difference tests (p>0.05) indicate model fit is not worse (for metric compared to configural, scalar compared to metric, or residual compared to scalar CFA models), and that measurement invariance is present (weak, strong, or strict measurement invariance, respectively) (27). However, log-likelihood ratio tests tend to be overly sensitive in the same ways that chi-square tests for model fit are (23,27). Therefore, we did not base our assessment of measurement invariance on the outcome of the log-likelihood ratio tests. We based our assessment of measurement invariance on changes in the model CFIs, TLIs, RMSEAs, and SRMRs (27). In particular, the CFI appears to be the best supported alternative criterion for determining measurement invariance (32,33). Decreases in the CFI and TLI of no more than 0.010, increases in the RMSEA of no more than 0.015, and increases in the SRMR of no more than 0.030 (for metric invariance) or 0.015 (for scalar or residual invariance), indicated that measurement invariance was present (27,32,33).

Complete or partial measurement invariance was considered sufficient (e.g., some authors have argued that only two indicators are needed to be invariant to make meaningful comparisons) (34). Subsequent SEMs were run with the complete or partial longitudinal measurement invariance constraints included in the model (35,36). We did not reject measurement invariance models based on the parsimony fit indices, unlike Mackinnon et al., (2022) who chose the BIC as an a-priori criterion (23)[[4]](#footnote-4). However, in cases where there were significant increases in the BIC between the scalar and residual models (≥6), we decided to run subsequent SEMs with the scalar measurement invariance constraints included in the model only (23,30).

We also conducted CFAs examining configural, weak or metric, and strong or scalar measurement invariance between settings (NI and Bogotá) and intervention groups (Dead Cool and ASSIST). Complete or partial scalar measurement invariance is required to compare latent means between groups (27). These CFA models were conducted with the latent variables at baseline and follow-up and including the complete or partial longitudinal measurement invariance constraints. We used the same criteria as for the longitudinal measurement invariance CFAs, based on the CFI, TLI, RMSEA, and SRMR. Where some of the indices or changes in the indices between measurement invariance models fell outside of the acceptable range, we based our decision on the CFI and on whether the changes in most indices were acceptable (23,32,33).

## English and Spanish language versions of the experimental protocol.


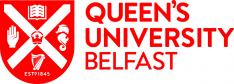


**Experimental Instructions**

**General information**

This is a study about decision-making. You will be paid a fee of £5 for taking part, as outlined below. In addition, you may receive some extra money based on your choices and the choices made by others during the study.

If you have any questions during the session, please raise your hand and wait for a researcher to come to you.  Please do not talk or try to communicate with other participants during the experiment.  It is important that everyone taking part makes his or her own decisions.

This is an on-going study, which has received funding from the UK Medical Research Council to cover all current and future costs. You can be certain that all participants who complete the study will be paid as described in the instructions. If you have any concerns, please contact:

**Dr. Ruth Hunter**
Centre for Public Health/UKCRC Centre of Excellence for Public Health (NI)
School of Medicine, Dentistry and Biomedical Sciences
Institute of Clinical Science B, Royal Victoria Hospital
Grosvenor Road, Belfast, BT 12 6BJ
E-mail: ruth.hunter@qub.ac.uk; 
Tel: +44 (0) 28 90978944
 
**There are four parts to today’s study.**

**You can earn money in each part.**

Your earnings from today will **not** be paid to you today. We will come back to your school at the end of the program in ten weeks’ time. At that time, we would like you to participate in another study. There will be four parts to that study, and you can earn money in each part of that study too.

After you have participated in the study at the end of the program we will determine for each part whether you receive earnings from today or from the study at the end of the program. For each part, we will toss a coin to determine this. We will record your choices in both today’s study and the study at the end of the program. You will be able to review your choices from both experiments when you learn your payment, if you wish.

**Part 1**

In Part 1 of this study, you will decide how to allocate 50 balls between two buckets. Your task is to put each of the balls, one-by-one, into one of the two buckets: the blue bucket or the yellow bucket. The balls will appear to the left-hand side of your screen, and you can allocate each ball by clicking and dragging it to the bucket of your choice. For each ball you put in the blue bucket, you will receive 5 pence, and for each ball you put in the yellow bucket, you will receive 10 pence.

The rule is to put the balls in the blue bucket.

Once the experiment begins, you will have 5 minutes to put the balls into the buckets. When you are finished, please click on the next button and wait quietly for further instructions from the experimenter. Any balls that have not been placed in a bucket at the end of the 5 minutes are worth nothing. Your earnings from Part 1 will be based on your decisions: it is the sum of earnings from the blue and yellow buckets.

This is the end of the instructions for Part 1. If you have any questions, please raise your hand and a researcher will answer them privately. Otherwise, please wait quietly until all of your classmates are ready and click on the next button to begin the experiment.


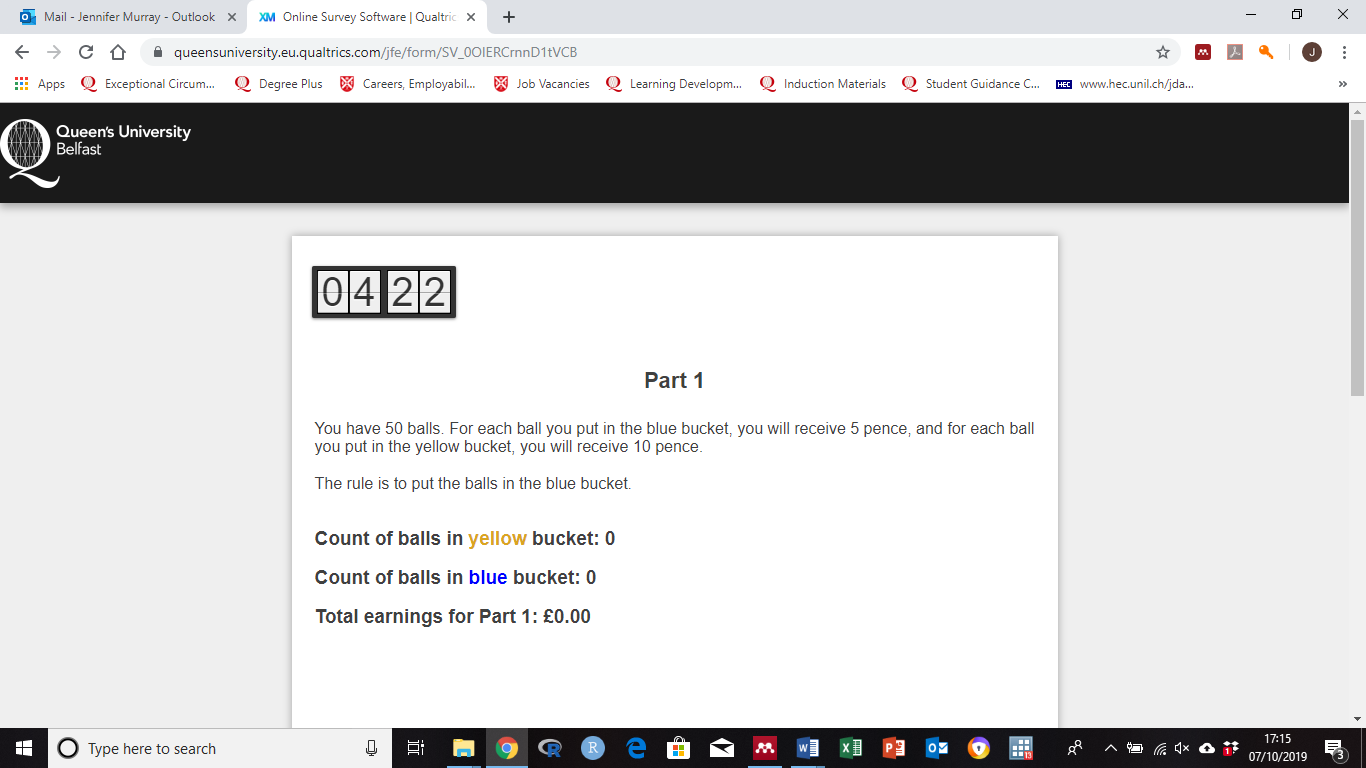


**The 50 balls can be re-located individually to either the blue or yellow bucket by mouse click and drag-and-drop.**

**Updated as balls are dragged in or out of the yellow bucket.**

**Updated as balls are dragged in or out of the blue bucket.**

**Timer indicating five-minute count-down for completing Rule-Following task.**


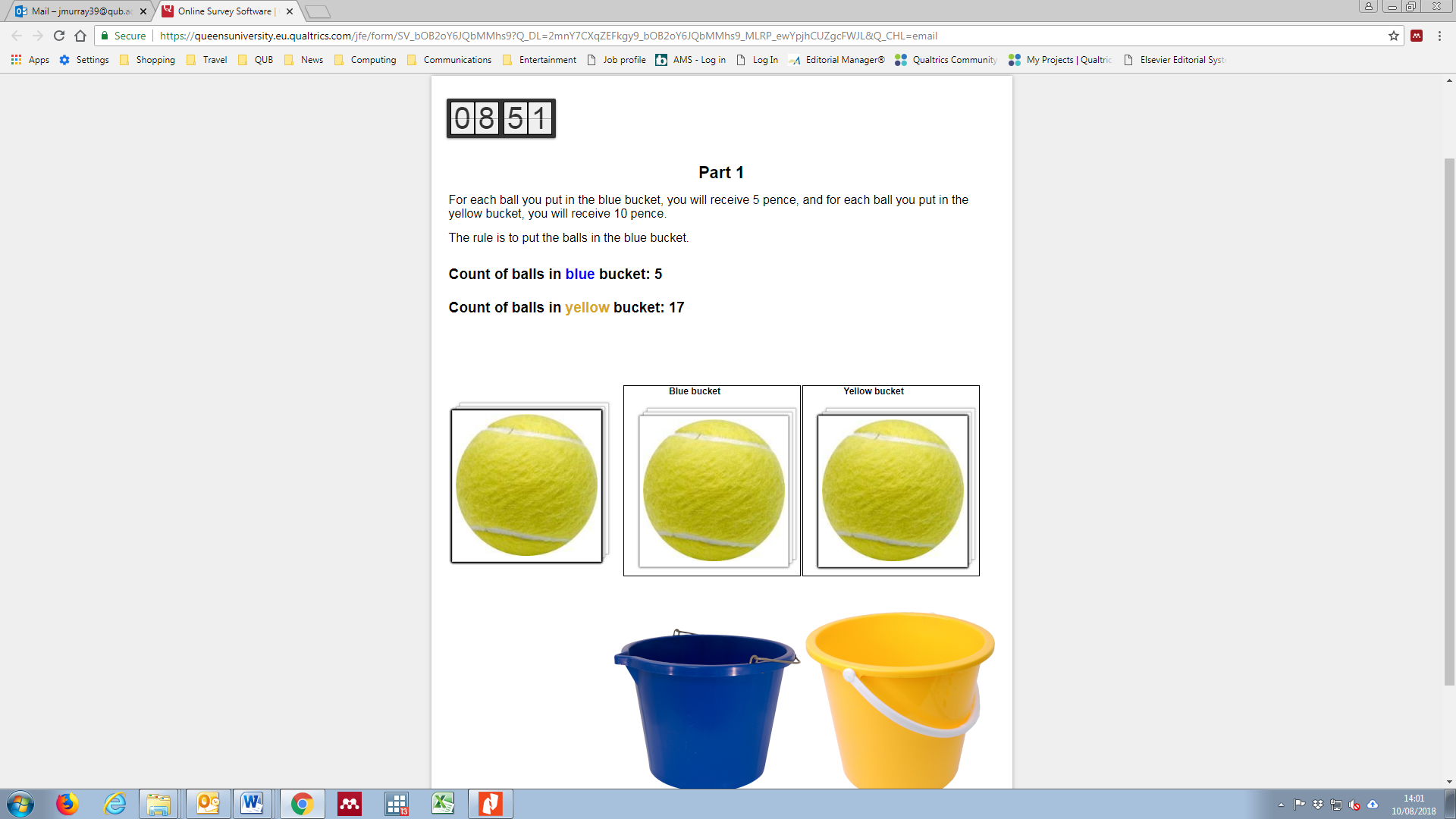


**N.B. Participants were randomised to this version of the experiment or to a version that had the buckets in reverse order to overcome any potential bias due to positioning of buckets.**

**Part 2**

On the following screens, you will read descriptions of a series of situations. These descriptions correspond to situations in which one person must make a decision or has taken an action. For each situation, you will be given a description of the decision faced or action taken by this person.

After you read the description of the situation, you will be asked to evaluate the decision or action taken. You will be asked to decide whether taking that decision or action would be "socially appropriate" and "consistent with moral or proper social behaviour" or "socially inappropriate" and "inconsistent with moral or proper social behaviour". By socially appropriate, we mean behaviour that most people in your school year group agree is the "correct" or "ethical" thing to do. Another way to think about what we mean is that if the person in the situation were to select a socially inappropriate choice, then someone else in your school year group might be angry with that person for doing so.

In each of your responses, we would like you to answer as truthfully as possible, based on your opinions of what constitutes socially appropriate or socially inappropriate behaviour.

To give you an idea of how the experiment will proceed, we will go through an example and show you how you will indicate your responses. On the next screen you will see an example of a situation.

**Part 2**

**Example Situation**

A person is at a local coffee shop near school. While there, the person notices that someone has left a wallet at one of the tables. The person must decide what to do. This person has four possible choices: take the wallet, ask others nearby if the wallet belongs to them, leave the wallet where it is, or give the wallet to the shop manager. The person can choose one of these four options.

The table below presents a list of the possible choices available to this person. For each of the choices, you will be asked to indicate whether you believe choosing that option is extremely socially inappropriate, very socially inappropriate, somewhat socially inappropriate, somewhat socially appropriate, very socially appropriate, or extremely socially appropriate. To indicate your response, you would select the corresponding option.

**The person's choice...**

|  | Extremely socially inappropriate | Very socially inappropriate | Somewhat socially inappropriate | Somewhat socially appropriate | Very socially appropriate | Extremely socially appropriate |
| --- | --- | --- | --- | --- | --- | --- |
| Take the wallet |  |  |  |  |  |  |
| Ask others nearby if the wallet belongs to them |  |  |  |  |  |  |
| Leave the wallet where it is |  |  |  |  |  |  |
| Give the wallet to the shop manager |  |  |  |  |  |  |

**Please make sure that you have placed one tick in each row.**

If this were one of the situations for this study, you would consider each of the possible choices above and, for that choice, indicate the extent to which you believe taking that action would be socially appropriate" and "consistent with moral or proper social behaviour" or "socially inappropriate" and "inconsistent with moral or proper social behaviour".  Recall that by socially appropriate we mean behaviour that most people agree is the "correct" or "ethical" thing to do.

**Part 2**

For example, suppose you thought that taking the wallet was extremely socially inappropriate, asking others nearby if the wallet belongs to them was somewhat socially appropriate, leaving the wallet where it is was somewhat socially inappropriate, and giving the wallet to the shop manager was extremely socially appropriate.  Then you would indicate your responses as follows:


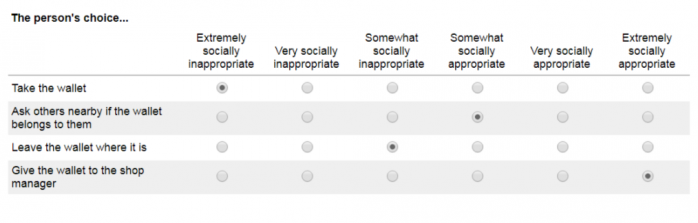
Are there any questions about this example situation or about how to indicate your responses?  On the following pages, there are several situations, all dealing with decisions that another person might have to make. You will indicate your appropriateness rating by selecting the corresponding option. At the end of the experiment today, we will randomly select one of the situations. For this situation, we will also randomly select one of the possible choices that Individual A could make. Thus, we will select both a situation and one possible choice at random. For the choice selected, we will find out which response was selected by most people in your school year group today. If you give the same response as that most frequently given by other people in your school year group, then your earning from Part 2 will be £10.  This amount will be paid to you, in cash, at the conclusion of the study in ten weeks. For instance, if we were to select the example situation above and the possible choice "Leave the wallet where it is", and if your response had been "somewhat socially inappropriate", then your earning from Part 2 would be £10, if this was the response selected by most other people in your school year group today. Otherwise your earning from Part 2 would be £0.

You are now going to complete some similar questions to this example on your own. You can go at your own pace. ***If you have any questions from this point on, please raise your hand and wait for the researcher to come to you.***

**Part 2**

**Situation 1**

Consider two hypothetical individuals from your school year group – Individual A and Individual B. Suppose that Individual A is randomly paired with another person in your school year group, Individual B in an experiment. The pairing is anonymous, meaning that neither individual will ever know the identity of the other individual with whom he or she is paired.

In this hypothetical experiment, Individual A will make a choice, the researcher will record this choice, and then both individuals will be informed of the choice and paid money based on the choice made by Individual A, as well as a small participation fee. Suppose that neither individual will receive any other money for participating in the experiment.

In each pair, Individual A will receive £10.  Individual A will then have the opportunity to give any amount of his or her £10 to Individual B. That is, Individual A can give any of the £10 he or she receives to Individual B. For instance, Individual A may decide to give £0 to Individual B and keep £10 for him or herself. Or Individual A may decide to give £10 to Individual B and keep £0 for him or herself. Individual A may also choose to give any other amount between £0 and £10 to Individual B. This choice will determine how much money each will receive, privately and in cash, at the end of the experiment.

The table below gives a list of the possible choices available to Individual A. For each of the choices, please indicate whether you believe choosing that option is extremely socially inappropriate, very socially inappropriate, somewhat socially inappropriate, somewhat socially appropriate, very socially appropriate, or extremely socially appropriate. To indicate your response, please select the corresponding option.

**Remember that you will earn money (£10) if your response to a randomly selected question is the same as the most common response provided in your school year group today.**

**Individual A's choice...**

|  | Extremely socially inappropriate | Very socially inappropriate | Somewhat socially inappropriate | Somewhat socially appropriate | Very socially appropriate | Extremely socially appropriate |
| --- | --- | --- | --- | --- | --- | --- |
| Give £0 to Individual B (Individual A gets £10, Individual B gets £0) |  |  |  |  |  |  |
| Give £1 to Individual B (Individual A gets £9, Individual B gets £1) |  |  |  |  |  |  |
| Give £2 to Individual B (Individual A gets £8, Individual B gets £2) |  |  |  |  |  |  |
| Give £3 to Individual B (Individual A gets £7, Individual B gets £3) |  |  |  |  |  |  |
| Give £4 to Individual B (Individual A gets £6, Individual B gets £4) |  |  |  |  |  |  |
| Give £5 to Individual B (Individual A gets £5, Individual B gets £5) |  |  |  |  |  |  |
| Give £6 to Individual B (Individual A gets £4, Individual B gets £6) |  |  |  |  |  |  |
| Give £7 to Individual B (Individual A gets £3, Individual B gets £7) |  |  |  |  |  |  |
| Give £8 to Individual B (Individual A gets £2, Individual B gets £8) |  |  |  |  |  |  |
| Give £9 to Individual B (Individual A gets £1, Individual B gets £9) |  |  |  |  |  |  |
| Give £10 to Individual B (Individual A gets £0, Individual B gets £10) |  |  |  |  |  |  |

**If you have any questions, please raise your hand and wait for the experimenter.**

**Part 2**

**Situation 2**

**A parent is smoking in their own home in front of their children who are under the age of 5.**

Please indicate whether you believe the parent smoking at home in front of their young children is extremely socially inappropriate, very socially inappropriate, somewhat socially inappropriate, somewhat socially appropriate, very socially appropriate, or extremely socially appropriate. To indicate your response, please select the corresponding option.

**Remember that you will earn money (£10) if your response to a randomly selected question is the same as the most common response provided in your school year group today.**

**A parent smoking at home in front of their young children.**

- Extremely socially inappropriate
- Very socially inappropriate
- Somewhat socially inappropriate
- Somewhat socially appropriate
- Very socially appropriate
- Extremely socially appropriate

**Part 2**

**Situation 3**

**An adult is smoking in a car with children under the age of 16 in the car.**

Please indicate whether you believe the adult smoking in the car with children in the car is extremely socially inappropriate, very socially inappropriate, somewhat socially inappropriate, somewhat socially appropriate, very socially appropriate, or extremely socially appropriate. To indicate your response, please select the corresponding option.

**Remember that you will earn money (£10) if your response to a randomly selected question is the same as the most common response provided in your school year group today.**

**Adult smoking in a car with children on board.**

- Extremely socially inappropriate
- Very socially inappropriate
- Somewhat socially inappropriate
- Somewhat socially appropriate
- Very socially appropriate
- Extremely socially appropriate

**Part 2**

**Situation 4**

**Someone sells cigarettes to a teenager who looks younger than 16 without requesting proof of age.**

Please indicate whether you believe someone selling cigarettes without proof of age is extremely socially inappropriate, very socially inappropriate, somewhat socially inappropriate, somewhat socially appropriate, very socially appropriate, or extremely socially appropriate. To indicate your response, please select the corresponding option.

**Remember that you will earn money (£10) if your response to a randomly selected question is the same as the most common response provided in your school year group today.**

**Someone selling cigarettes without proof of age.**

- Extremely socially inappropriate
- Very socially inappropriate
- Somewhat socially inappropriate
- Somewhat socially appropriate
- Very socially appropriate
- Extremely socially appropriate

**Part 2**

**Situation 5**

**In a recent superhero movie the lead actor is seen smoking in the opening scene.**

Please indicate whether you believe the movie showing the lead character smoking is extremely socially inappropriate, very socially inappropriate, somewhat socially inappropriate, somewhat socially appropriate, very socially appropriate, or extremely socially appropriate. To indicate your response, please select the corresponding option.

**Remember that you will earn money (£10) if your response to a randomly selected question is the same as the most common response provided in your school year group today.**

**A movie showing the lead character smoking.**

- Extremely socially inappropriate
- Very socially inappropriate
- Somewhat socially inappropriate
- Somewhat socially appropriate
- Very socially appropriate
- Extremely socially appropriate

**Part 2**

**Situation 6**

**An older student in your school is smoking outside school, for example, at a bus stop.**

Please indicate whether you believe an older student smoking outside school is extremely socially inappropriate, very socially inappropriate, somewhat socially inappropriate, somewhat socially appropriate, very socially appropriate, or extremely socially appropriate. To indicate your response, please select the corresponding option.

**Remember that you will earn money (£10) if your response to a randomly selected question is the same as the most common response provided in your school year group today.**

**An older student from your school smoking outside school.**

- Extremely socially inappropriate
- Very socially inappropriate
- Somewhat socially inappropriate
- Somewhat socially appropriate
- Very socially appropriate
- Extremely socially appropriate

**Part 2**

**Situation 7**

**A pupil from your school is using an e-cigarette while walking to school.**

Please indicate whether you believe the pupil using an e-cigarette is extremely socially inappropriate, very socially inappropriate, somewhat socially inappropriate, somewhat socially appropriate, very socially appropriate, or extremely socially appropriate. To indicate your response, please select the corresponding option.

**Remember that you will earn money (£10) if your response to a randomly selected question is the same as the most common response provided in your school year group today.**

**A school student smoking an e-cigarette.**

- Extremely socially inappropriate
- Very socially inappropriate
- Somewhat socially inappropriate
- Somewhat socially appropriate
- Very socially appropriate
- Extremely socially appropriate

**Part 2**

**Situation 8**

**A pupil from your school shares a photograph of him/herself using an e-cigarette on social media (e.g. Facebook, Instagram).**

Please indicate whether you believe the pupil sharing an image of e-cigarette use is extremely socially inappropriate, very socially inappropriate, somewhat socially inappropriate, somewhat socially appropriate, very socially appropriate, or extremely socially appropriate. To indicate your response, please select the corresponding option.

**Remember that you will earn money (£10) if your response to a randomly selected question is the same as the most common response provided in your school year group today.**

**A student sharing a photo of his/her e-cigarette use.**

- Extremely socially inappropriate
- Very socially inappropriate
- Somewhat socially inappropriate
- Somewhat socially appropriate
- Very socially appropriate
- Extremely socially appropriate

**Part 2**

**Situation 9**

**A pupil from your school is chewing tobacco.**

Please indicate whether you believe the pupil chewing tobacco is extremely socially inappropriate, very socially inappropriate, somewhat socially inappropriate, somewhat socially appropriate, very socially appropriate, or extremely socially appropriate. To indicate your response, please select the corresponding option.

**Remember that you will earn money (£10) if your response to a randomly selected question is the same as the most common response provided in your school year group today.**

**A school pupil chewing tobacco.**

- Extremely socially inappropriate
- Very socially inappropriate
- Somewhat socially inappropriate
- Somewhat socially appropriate
- Very socially appropriate
- Extremely socially appropriate

**This is the end of Part 2 of the experiment.**

In Part 3 of today's experiment you will be asked some questions about the behaviour of your peers. By peers, we mean “other students in your school year group”. After today we will randomly select a question from part 3. If you give the same response as that most frequently given by other people in your school year group, then your earning from Part 3 will be £10. This amount will be paid to you, in cash, at the conclusion of the study in ten weeks. Please click on the next button when you are ready to proceed.

**Part 3**

**Question 1**

**What share of your school year group would be accepting of one of their close friends smoking?**

*Please indicate what proportion of students in your school year group (your peers) you believe would be accepting of one of their close friends smoking: All of my peers; most of my peers; a lot of my peers; some of my peers; only a few of my peers; none of my peers. To indicate your response, please select the corresponding option.*

**Remember that you will earn money (£10) if your response to a randomly selected question is the same as the most common response provided in your school year group today.**

**The proportion of my peers who would be accepting of a close friend smoking.**

- All of my peers
- Most of my peers
- A lot of my peers
- Some of my peers
- Only a few of my peers
- None of my peers

**Part 3**

**Question 2**

**What share of your school year group would be accepting of one of their close friends vaping (using an e-cigarette)?**

Please indicate what proportion of students *in your school year group (your peers)* you believe would be accepting of one of their close friends using an e-cigarette: All of my peers; most of my peers; a lot of my peers; some of my peers; only a few of my peers; none of my peers. To indicate your response, please select the corresponding option.

**Remember that you will earn money (£10) if your response to a randomly selected question is the same as the most common response provided in your school year group today.**

**The proportion of my peers who would be accepting of a close friend vaping.**

- All of my peers
- Most of my peers
- A lot of my peers
- Some of my peers
- Only a few of my peers
- None of my peers

**This is the end of Part 3 of the experiment.**

Please wait for the experimenter to tell you when to proceed to Part 4.

**Part 4**

You will be given 10 virtual tokens. Each token is worth 50 pence. That means you will receive tokens worth £5.

You will then have the opportunity to give any amount of your £5 to the ASSIST Program.

ASSIST is a smoking prevention program which aims to prevent the uptake of smoking among adolescents your age.

You can give any of the £5 you receive to ASSIST. For instance, you may decide to give £0 to ASSIST and keep £5 for yourself. Or you may decide to give £5 to ASSIST and keep £0 for yourself. You may also choose to give any other amount between £0 and £5 to ASSIST.

The value of any tokens you do not give to ASSIST will be your earnings for this Part. That is, each token that you do not give to ASSIST will increase your own payment for Part 4 by 50 pence.

**How many tokens do you want to give to ASSIST?**

- 0 (you earn £5.00)
- 1 (you earn £4.50)
- 2 (you earn £4.00)
- 3 (you earn £3.50)
- 4 (you earn £3.00)
- 5 (you earn £2.50)
- 6 (you earn £2.00)
- 7 (you earn £1.50)
- 8 (you earn £1.00)
- 9 (you earn £0.50)
- 10 (you earn £0.00)

**
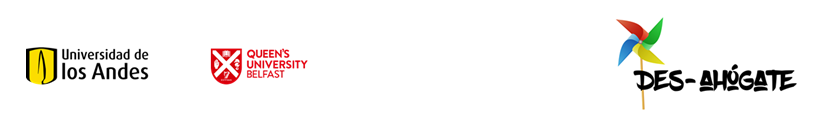
**

**Instrucciones para los experimentos**

**Información general**

Este es un estudio sobre toma de decisiones. Por participar te daremos un monto de $ 5.000 en una tarjeta de regalo. Además, puedes recibir un dinero extra en esta tarjeta en función de tus elecciones y las decisiones tomadas por otros durante el estudio.

Si tienes alguna pregunta durante la sesión, levanta la mano y espera a la persona encargada. Es muy importante que no hables ni trates de comunicarte con otros estudiantes durante el experimento. También es importante que todos los participantes tomen sus propias decisiones.

Este es un estudio que recibe financiación del Consejo de Investigación Médica del Reino Unido y cubrirá todos los gastos actuales y futuros. Por eso, puedes estar seguro de que a todos los estudiantes que participan se les pagará como se describe en las instrucciones en una tarjeta de regalo. Si tienes alguna duda, comunícate con el encargado en el salón o con Sharon Sánchez en la Facultad de Medicina de la Universidad de los Andes al teléfono 3394949 ext.3803 o al correo sc.sanchez@uniandes.edu.co.

**Hay cuatro partes en el estudio de hoy. Puedes ganar dinero en cada parte.**

Tus ganancias NO se pagarán hoy. La tarjeta de regalo será entregada al final del programa en 16 semanas. En ese momento volveremos para realizar otro estudio similar a este en el que podrás ganar dinero en cada parte. Tus ganancias dependen de tus respuestas en la sesión de experimentos de hoy y en la sesión del final del programa. Ten en cuenta que recibirás ganancias solo por una de las sesiones de experimentos, que se elegirá al azar lanzando una moneda. Para poder determinar tus ganancias, registraremos tus respuestas tanto en la sesión de hoy como en la sesión al final del programa. Si lo deseas, al final podrás verificar que el pago asignado corresponde a las respuestas que realizaste durante las dos sesiones.

**Parte 1**

En la parte 1 de este estudio, decidirás cómo encestar 50 pelotas entre dos baldes. Tu tarea es colocar cada una de las pelotas, una a una, en uno de los dos baldes: el balde azul o el balde amarillo. Las pelotas aparecerán en la parte izquierda de la pantalla, y puedes encestar cada pelota haciendo clic y arrastrándola al balde de tu elección. Por cada pelota que pongas en el balde azul, recibirás $100, y por cada pelota que pongas en el balde amarillo, recibirás $200.

La regla es poner las pelotas en el balde azul.

Una vez que comience el experimento, tendrás 5 minutos para poner las pelotas en los baldes. Cuando hayas terminado, espera en silencio hasta que el tiempo se acabe. Las bolas que no hayan sido encestadas en ningún balde no valen nada. Tus ganancias de la parte 1 se basarán en tus decisiones: es la suma de los pagos correspondientes a las pelotas que se encuentren en los baldes azules y amarillos.

Este es el final de las instrucciones para la parte 1. Si tienes alguna pregunta, levanta la mano y el encargado las contestará en privado. De lo contrario, espera en silencio hasta que todo el mundo haya terminado. **Solo cuando el encargado lo indique**, da clic en la siguiente pestaña para comenzar el experimento.

**Parte 1**

Tienes 50 pelotas. Por cada pelota que pongas en el balde azul, recibirás $100, y por cada bola que pongas en el balde amarillo, recibirás $200.


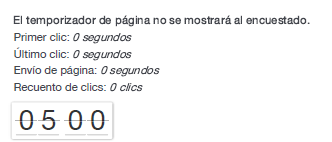


La regla es poner las bolas en el balde azul.

Arrastra y suelta cada pelota dentro del espacio correspondiente. Clasifique los elementos arrastrándolos y colocándolos en su lugar.


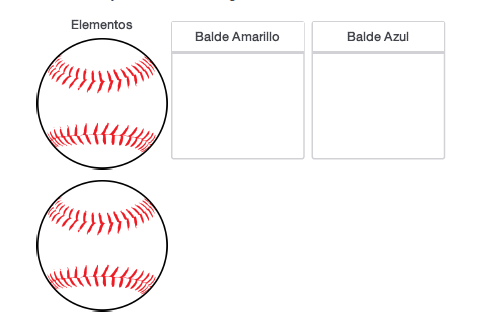


**Este es el final de la Parte 1 del experimento.**

Por favor, espera a que el encargado te indique cuándo empezar con la parte 2.

**Parte 2**

En las siguientes pantallas, se describirán varias situaciones. En estas situaciones una persona debe tomar una decisión o ha realizado una acción. Para cada situación, se describe la decisión o acción tomada por esta persona.

Después de leer la situación, se te pedirá que evalúes la decisión o acción tomada por esa persona. Debes decidir si lo que la persona hace sería “socialmente apropiado” y “consistente con un comportamiento moralmente aceptable o socialmente apropiado” o si el comportamiento de la persona sería “socialmente inapropiado” e “incompatible con una conducta moralmente aceptable o socialmente apropiada”. Por socialmente apropiado, nos referimos al comportamiento que la mayoría de las personas en tu grado escolar está de acuerdo que es lo “correcto” o “ético”. Otra forma de pensar sobre lo que queremos decir es que, si la persona en la situación descrita elije una opción socialmente inapropiada, alguien más en el grado escolar podría estar enojado con esa persona por hacerlo.

En cada una de tus respuestas, nos gustaría que respondas lo más sinceramente posible, en función de tus opiniones sobre lo que es un comportamiento socialmente apropiado o socialmente inapropiado.

Para explicarte cómo procederá el experimento, veremos un ejemplo y te mostraremos cómo podrías indicar tus respuestas. Recuerda que este es solo un ejemplo, tú podrás marcar cada respuesta de acuerdo con lo que opines. No hay respuestas buenas y malas. En la siguiente pantalla, verás un ejemplo de una situación y el encargado del salón lo explicará para todos.

**Parte 2**

**Ejemplo de situación.**

Una persona está en una cafetería cerca del colegio. Mientras está allí, la persona se da cuenta de que alguien ha dejado una billetera en una de las mesas. La persona debe decidir qué hacer. Esta persona tiene cuatro posibles opciones: llevarse la billetera, preguntar a otras personas que estén cerca si la billetera es suya, dejar la billetera donde está o darle la billetera al administrador de la tienda. La persona debe elegir una de las cuatro opciones.

*La siguiente tabla presenta una lista de las posibles opciones disponibles para esta persona. Para cada una de las opciones, debes indicar si crees que elegir esa opción es extremadamente inapropiado, socialmente muy inapropiado, socialmente algo inapropiado, socialmente algo apropiado, socialmente muy apropiado, extremadamente apropiado. Para indicar tu respuesta, marca la casilla correspondiente a la opción deseada:*

| **La persona elige:** | *Extremadamente inapropiado* | *Socialmente muy inapropiado* | *Socialmente algo inapropiado* | *Socialmente algo apropiado* | *Socialmente muy apropiado* | *Extremadamente apropiado* |
| --- | --- | --- | --- | --- | --- | --- |
| Llevarse la billetera | 🞆 | 🞆 | 🞆 | 🞆 | 🞆 | 🞆 |
| Preguntar a personas cercanas si la billetera es suya. | 🞆 | 🞆 | 🞆 | 🞆 | 🞆 | 🞆 |
| Dejar la billetera donde está | 🞆 | 🞆 | 🞆 | 🞆 | 🞆 | 🞆 |
| Darle la billetera al administrador de la tienda. | 🞆 | 🞆 | 🞆 | 🞆 | 🞆 | 🞆 |

**Por favor, asegúrate de seleccionar la opción deseada**

Si esta fuera una de las situaciones para este estudio, tú considerarías cada una de las posibles opciones anteriores y, para esa elección, indicarías hasta qué punto crees que tomar esa acción sería “socialmente apropiado” y “consistente con un comportamiento moralmente aceptable o socialmente apropiado” o si el comportamiento de la persona sería “socialmente inapropiado” e “incompatible con una conducta moralmente aceptable o socialmente apropiada. Recuerda que por “socialmente apropiado” nos referimos al comportamiento con el que la mayoría de las personas está de acuerdo, es lo “correcto” o “ético”.

**Parte 2**

Por ejemplo, supongamos que pensaste que tomar la billetera era *Extremadamente inapropiado*, preguntar a otras personas cercanas si la billetera era suya era *socialmente algo apropiado*, dejar la billetera donde estaba era *socialmente algo inapropiado* y darle la billetera al administrador de la tienda era *Extremadamente apropiado*. Entonces, tu habrías indicado tus respuestas de la siguiente manera:


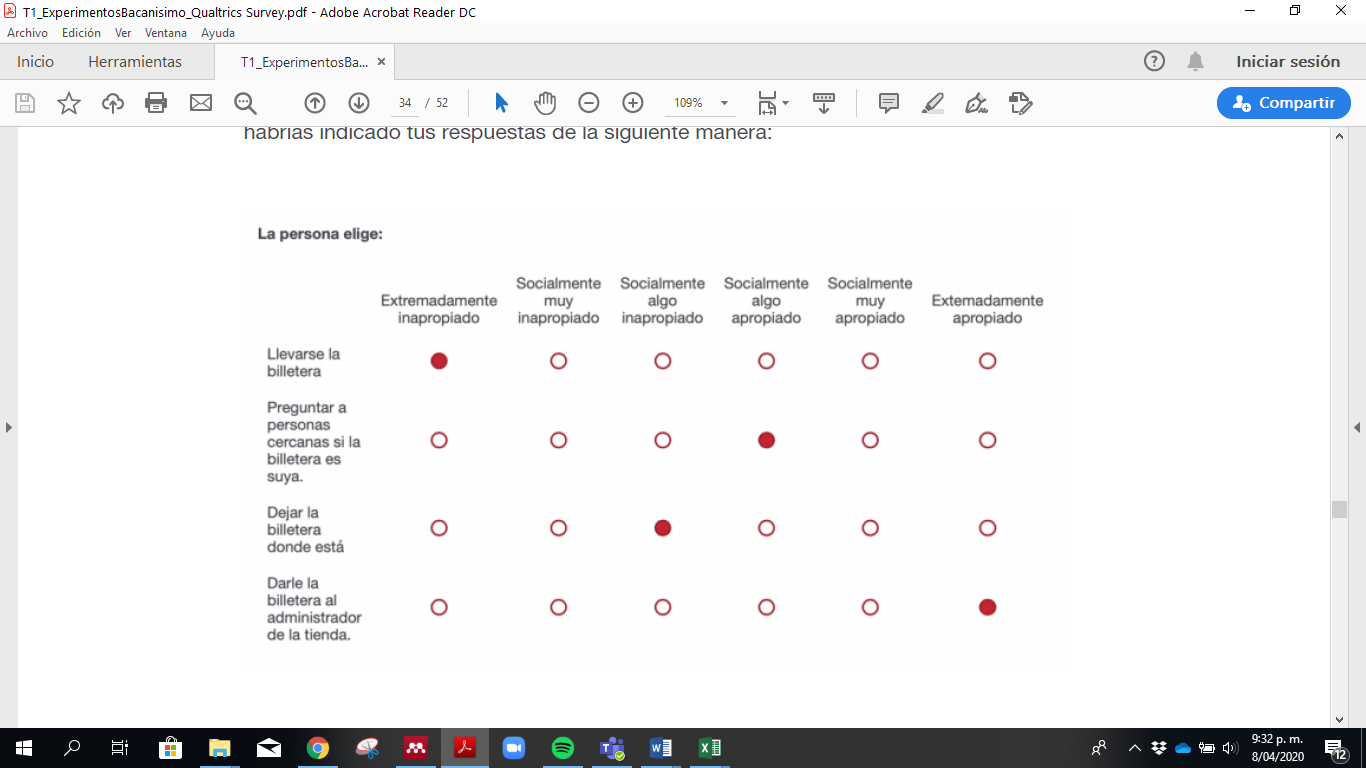


¿Hay alguna pregunta sobre este ejemplo o sobre cómo indicar tus respuestas? En las siguientes páginas, hay varias situaciones, todas relacionadas con decisiones que una persona podría tener que hacer.

Para cada situación, podrás marcar qué tan idóneo te parece cada acción en el recuadro correspondiente.

Al final del experimento de hoy, con un juego de lotería seleccionaremos al azar una de las situaciones. Para esta situación, también seleccionaremos al azar una de las opciones posibles que la persona podría hacer. Por lo tanto, seleccionaremos una situación y una posible elección al azar. Para la opción seleccionada, descubriremos qué respuesta fue seleccionada hoy por la mayoría de tus compañeros en el grado escolar.

Si tú escribes la misma respuesta que la mayoría de tus compañeros de grado, podrás recibir $15.000 por la parte 2 del experimento. Este premio se te entregará en la tarjeta de regalo al final del programa 16 semanas para EntreParceros. Por ejemplo, si en el ejemplo anterior, seleccionáramos al azar para premiar la opción “deje la billetera donde está”, y si tu respuesta fuese la misma que la mayoría de tus compañeros, entonces tu ganancia sería de $15.000, si esta fue la respuesta seleccionada por la mayoría de tus compañeros de grado el día de hoy. De lo contrario, su ganancia de la parte 2 sería $0.

Ahora, vas a completar algunas preguntas similares a este ejemplo por tu cuenta. Puedes ir a tu propio ritmo.

**Si tienes alguna pregunta a partir de este punto, levanta la mano y espera a que el encargado acuda.**

**Parte 2**

**Situación 1.**

Imagina dos personas hipotéticas de tu grado escolar que son ubicados en parejas al azar: Individuo A e Individuo B. El emparejamiento es anónimo, es decir, nadie sabe quién es la pareja de quién.

En este experimento hipotético, el individuo A hará una elección sobre dar dinero al otro, el encargado del curso registrará esta elección, y luego se la informará a ambas personas. Supongamos que ninguna persona recibirá ningún otro dinero diferente a lo que haga en esta situación hipotética.

En la pareja, el individuo A recibe $10.000. El individuo A tendrá entonces la oportunidad de dar cualquier cantidad de sus $10.000 al individuo B. Es decir, el individuo A puede darle al individuo B lo que quiera de los $10.000 que recibió. Por ejemplo, el individuo A puede decidir darle al individuo B $0 y mantener $10.000 para él o ella. O el individuo A puede decidir darle al individuo B los $10.000 y mantener $0 para él o ella. El individuo A también puede optar por dar cualquier otra cantidad entre $0 y $10.000 al individuo B.

Recuerda que tu respuesta sobre lo socialmente apropiado de las acciones del individuo A determinará la ganancia en tu tarjeta de regalo al final del estudio.

*La siguiente tabla presenta una lista de las posibles opciones disponibles para esta persona. Para cada una de las opciones, por favor, indica si crees que elegir esa opción es extremadamente inapropiado, socialmente muy inapropiado, socialmente algo inapropiado, socialmente algo apropiado, socialmente muy apropiado, extremadamente apropiado. Para indicar tu respuesta, marca la casilla correspondiente.*

Recuerda que ganarás un premio ($15.000) en la tarjeta de regalo si tu respuesta a una pregunta seleccionada al azar coincide con la respuesta más común brindada hoy por tus compañeros del grado escolar. Es decir, **para que sea más probable ganar el premio, debes responder según lo que tú crees que la mayoría de tus compañeros piensan.**

El individuo A elige:

| El individuo A elige: | *Extremadamente inapropiado* | *Socialmente muy inapropiado* | *Socialmente algo inapropiado* | *Socialmente algo apropiado* | *Socialmente muy apropiado* | *Extremadamente apropiado* |
| --- | --- | --- | --- | --- | --- | --- |
| Darle $0 al individuo B  (Individuo A obtiene $10.000, Individuo B obtiene $0) | 🞆 | 🞆 | 🞆 | 🞆 | 🞆 | 🞆 |
| Darle $1.000 al individuo B  (Individuo A obtiene $9.000, Individuo B obtiene $1.000 | 🞆 | 🞆 | 🞆 | 🞆 | 🞆 | 🞆 |
| Darle $2.000 al individuo B  (Individuo A obtiene $8.000, Individuo B obtiene $2.000) | 🞆 | 🞆 | 🞆 | 🞆 | 🞆 | 🞆 |
| Darle $3.000 al individuo B  (Individuo A obtiene $7.000, Individuo B obtiene $3.000) | 🞆 | 🞆 | 🞆 | 🞆 | 🞆 | 🞆 |
| Darle $4.000 al individuo B  (Individuo A obtiene $6.000, Individuo B obtiene $4.000) | 🞆 | 🞆 | 🞆 | 🞆 | 🞆 | 🞆 |
| Darle $5.000 al individuo B  (Individuo A obtiene $5.000, Individuo B obtiene $5.000) | 🞆 | 🞆 | 🞆 | 🞆 | 🞆 | 🞆 |
| Darle $6.000 al individuo B  (Individuo A obtiene $4.000, Individuo B obtiene $6.000) | 🞆 | 🞆 | 🞆 | 🞆 | 🞆 | 🞆 |
| Darle $5.000 al individuo B  (Individuo A obtiene $3.000, Individuo B obtiene $7.000) | 🞆 | 🞆 | 🞆 | 🞆 | 🞆 | 🞆 |
| Darle $8.000 al individuo B  (Individuo A obtiene $2.000, Individuo B obtiene $8.000) | 🞆 | 🞆 | 🞆 | 🞆 | 🞆 | 🞆 |
| Darle $9.000 al individuo B  (Individuo A obtiene $1.000, Individuo B obtiene $9.000) | 🞆 | 🞆 | 🞆 | 🞆 | 🞆 | 🞆 |
| Darle $10.000 al individuo B  (Individuo A obtiene $0, Individuo B obtiene $10.000) | 🞆 | 🞆 | 🞆 | 🞆 | 🞆 | 🞆 |

**Si tienes alguna pregunta, por favor levanta la mano y espera por el encargado del salón.**

**Parte 2**

**Situación 2.**

Un padre o una madre fuma en su propia casa frente a sus hijos menores de 5 años.

*Indica si crees que el padre fumando en la casa delante de sus hijos pequeños es extremadamente inapropiado, socialmente muy inapropiado, socialmente algo inapropiado, socialmente algo apropiado, socialmente muy apropiado, extremadamente apropiado. Para indicar tu respuesta marca la casilla correspondiente.*

Recuerda que ganarás un premio ($15.000) en la tarjeta de regalo si tu respuesta a una pregunta seleccionada al azar coincide con la respuesta más común brindada hoy por tus compañeros del grado escolar. Es decir, **para que sea más probable ganar el premio, debes responder según lo que tú crees que la mayoría de tus compañeros piensan.**

Un padre o una madre fuma en su propia casa frente a sus hijos menores de 5 años.

| *Extremadamente inapropiado* | *Socialmente muy inapropiado* | *Socialmente algo inapropiado* | *Socialmente algo apropiado* | *Socialmente muy apropiado* | *Extremadamente apropiado* |
| --- | --- | --- | --- | --- | --- |
| 🞆 | 🞆 | 🞆 | 🞆 | 🞆 | 🞆 |

**Parte 2**

**Situación 3.**

Un adulto fuma en un carro con personas menores de 16 años en el auto.

*Indica si crees que un adulto fumando en un carro con niños menores de 16 años en el carro es extremadamente inapropiado, socialmente muy inapropiado, socialmente algo inapropiado, socialmente algo apropiado, socialmente muy apropiado, extremadamente apropiado. Para indicar tu respuesta marca la casilla correspondiente.*

Recuerda que ganarás un premio ($15.000) en la tarjeta de regalo si tu respuesta a una pregunta seleccionada al azar coincide con la respuesta más común brindada hoy por tus compañeros del grado escolar. Es decir, **para que sea más probable ganar el premio, debes responder según lo que tú crees que la mayoría de tus compañeros piensan.**

Un adulto fuma en un carro con personas menores de 16 años en el auto.

| *Extremadamente inapropiado* | *Socialmente muy inapropiado* | *Socialmente algo inapropiado* | *Socialmente algo apropiado* | *Socialmente muy apropiado* | *Extremadamente apropiado* |
| --- | --- | --- | --- | --- | --- |
| 🞆 | 🞆 | 🞆 | 🞆 | 🞆 | 🞆 |

**Parte 2**

**Situación 4.**

Alguien vende cigarrillos a un adolescente que parece tener menos de 16 años sin pedirle la cédula para comprobar su edad.

*Indica si crees que alguien venda cigarrillos a un adolescente que parece tener menos de 16 años sin solicitar prueba de edad es extremadamente inapropiado, socialmente muy inapropiado, socialmente algo inapropiado, socialmente algo apropiado, socialmente muy apropiado, extremadamente apropiado.*

Recuerda que ganarás un premio ($15.000) en la tarjeta de regalo si tu respuesta a una pregunta seleccionada al azar coincide con la respuesta más común brindada hoy por tus compañeros del grado escolar. Es decir, **para que sea más probable ganar el premio, debes responder según lo que tú crees que la mayoría de tus compañeros piensan.**

Alguien vende cigarrillos a un adolescente que parece tener menos de 16 años sin pedirle la cédula para comprobar su edad.

| *Extremadamente inapropiado* | *Socialmente muy inapropiado* | *Socialmente algo inapropiado* | *Socialmente algo apropiado* | *Socialmente muy apropiado* | *Extremadamente apropiado* |
| --- | --- | --- | --- | --- | --- |
| 🞆 | 🞆 | 🞆 | 🞆 | 🞆 | 🞆 |

**Parte 2**

**Situación 5.**

En una película reciente de superhéroes, se ve al actor principal fumando en la primera escena.

*Indica si crees que en una película mostrando al actor principal fumando en la primera escena* *es extremadamente inapropiado, socialmente muy inapropiado, socialmente algo inapropiado, socialmente algo apropiado, socialmente muy apropiado, extremadamente apropiado.*

Recuerda que ganarás un premio ($15.000) en la tarjeta de regalo si tu respuesta a una pregunta seleccionada al azar coincide con la respuesta más común brindada hoy por tus compañeros del grado escolar. Es decir, **para que sea más probable ganar el premio, debes responder según lo que tú crees que la mayoría de tus compañeros piensan.**

En una película reciente de superhéroes, se ve al actor principal fumando en la primera escena.

| *Extremadamente inapropiado* | *Socialmente muy inapropiado* | *Socialmente algo inapropiado* | *Socialmente algo apropiado* | *Socialmente muy apropiado* | *Extremadamente apropiado* |
| --- | --- | --- | --- | --- | --- |
| 🞆 | 🞆 | 🞆 | 🞆 | 🞆 | 🞆 |

**Parte 2**

**Situación 6.**

Un estudiante mayor de tu colegio está fumando fuera del colegio, por ejemplo, en un paradero de bus.

*Indica si crees que un estudiante mayor de tu colegio está fumando fuera del colegio es extremadamente inapropiado, socialmente muy inapropiado, socialmente algo inapropiado, socialmente algo apropiado, socialmente muy apropiado, extremadamente apropiado. Para indicar tu respuesta marca la casilla correspondiente.*

Recuerda que ganarás un premio ($15.000) en la tarjeta de regalo si tu respuesta a una pregunta seleccionada al azar coincide con la respuesta más común brindada hoy por tus compañeros del grado escolar. Es decir, **para que sea más probable ganar el premio, debes responder según lo que tú crees que la mayoría de tus compañeros piensan.**

Un estudiante mayor de tu colegio está fumando fuera del colegio, por ejemplo, en un paradero de bus.

| *Extremadamente inapropiado* | *Socialmente muy inapropiado* | *Socialmente algo inapropiado* | *Socialmente algo apropiado* | *Socialmente muy apropiado* | *Extremadamente apropiado* |
| --- | --- | --- | --- | --- | --- |
| 🞆 | 🞆 | 🞆 | 🞆 | 🞆 | 🞆 |

**Parte 2**

**Situación 7.**

Un estudiante de tu colegio usa un cigarrillo electrónico mientras camina hacia el colegio.

*Indica si crees que el alumno usando un cigarrillo electrónico mientras camina hacia el colegio* *es extremadamente inapropiado, socialmente muy inapropiado, socialmente algo inapropiado, socialmente algo apropiado, socialmente muy apropiado, extremadamente apropiado. Para indicar tu respuesta marca la casilla correspondiente.*

Recuerda que ganarás un premio ($15.000) en la tarjeta de regalo si tu respuesta a una pregunta seleccionada al azar coincide con la respuesta más común brindada hoy por tus compañeros del grado escolar. Es decir, **para que sea más probable ganar el premio, debes responder según lo que tú crees que la mayoría de tus compañeros piensan.**

Un estudiante de tu colegio usa un cigarrillo electrónico mientras camina hacia el colegio.

| *Extremadamente inapropiado* | *Socialmente muy inapropiado* | *Socialmente algo inapropiado* | *Socialmente algo apropiado* | *Socialmente muy apropiado* | *Extremadamente apropiado* |
| --- | --- | --- | --- | --- | --- |
| 🞆 | 🞆 | 🞆 | 🞆 | 🞆 | 🞆 |

**Parte 2**

**Situación 8.**

Un estudiante de tu colegio comparte una fotografía de sí mismo utilizando un cigarrillo electrónico en redes sociales (Ej. Facebook o Instagram).

*Indica si crees que un alumno de tu colegio compartiendo una fotografía de sí mismo utilizando un cigarrillo electrónico en redes sociales (Ej. Facebook o Instagram*) *es extremadamente inapropiado, socialmente muy inapropiado, socialmente algo inapropiado, socialmente algo apropiado, socialmente muy apropiado, extremadamente apropiado.*

Recuerda que ganarás un premio ($15.000) en la tarjeta de regalo si tu respuesta a una pregunta seleccionada al azar coincide con la respuesta más común brindada hoy por tus compañeros del grado escolar. Es decir, **para que sea más probable ganar el premio, debes responder según lo que tú crees que la mayoría de tus compañeros piensan.**

Un estudiante de tu colegio comparte una fotografía de sí mismo utilizando un cigarrillo electrónico en redes sociales (Ej. Facebook o Instagram).

| *Extremadamente inapropiado* | *Socialmente muy inapropiado* | *Socialmente algo inapropiado* | *Socialmente algo apropiado* | *Socialmente muy apropiado* | *Extremadamente apropiado* |
| --- | --- | --- | --- | --- | --- |
| 🞆 | 🞆 | 🞆 | 🞆 | 🞆 | 🞆 |

**Parte 2**

**Situación 9.**

Un estudiante de tu colegio está masticando tabaco.

*Indica si crees que un estudiante de tu colegio masticando tabaco es extremadamente inapropiado, socialmente muy inapropiado, socialmente algo inapropiado, socialmente algo apropiado, socialmente muy apropiado, extremadamente apropiado. Para indicar tu respuesta marca la casilla correspondiente.*

Recuerda que ganarás un premio ($15.000) en la tarjeta de regalo si tu respuesta a una pregunta seleccionada al azar coincide con la respuesta más común brindada hoy por tus compañeros del grado escolar. Es decir, **para que sea más probable ganar el premio, debes responder según lo que tú crees que la mayoría de tus compañeros piensan.**

Un estudiante de tu colegio está masticando tabaco.

| *Extremadamente inapropiado* | *Socialmente muy inapropiado* | *Socialmente algo inapropiado* | *Socialmente algo apropiado* | *Socialmente muy apropiado* | *Extremadamente apropiado* |
| --- | --- | --- | --- | --- | --- |
| 🞆 | 🞆 | 🞆 | 🞆 | 🞆 | 🞆 |

**Este es el final de la Parte 2 del experimento.**

En la Parte 3 del experimento de hoy responderás varias preguntas sobre el comportamiento de tus compañeros, es decir, otros estudiantes en tu mismo grado escolar. Al final del estudio, con un juego de lotería seleccionaremos al azar una de las situaciones. Para esta situación, también seleccionaremos al azar una de las opciones posibles. Por lo tanto, seleccionaremos una situación y una posible elección al azar. Para la opción seleccionada, descubriremos qué respuesta fue seleccionada por la mayoría de tus compañeros en el grado escolar.

Si tú escribes la misma respuesta que la mayoría de tus compañeros de grado, podrás recibir $15.000 por la parte 3 del experimento. Este premio se te entregará en la tarjeta de regalo al final del programa 16 semanas para EntreParceros.

Por favor, da click en el botón cuando estés listo para empezar con la Parte 3.

**Parte 3**

**Pregunta 1.**

¿Cuántos de tus compañeros del grado aceptarían que uno de sus amigos cercanos fumara?

*Indica qué proporción de estudiantes del grado crees que aceptaría que uno de sus amigos cercanos fumara: todos mis compañeros, la mayoría de mis compañeros, muchos de mis compañeros, algunos de mis compañeros, solo unos pocos de mis compañeros, ninguno de mis compañeros. Para indicar tu respuesta marca la casilla correspondiente.*

Recuerda que ganarás un premio ($15.000) en la tarjeta de regalo si tu respuesta a una pregunta seleccionada al azar coincide con la respuesta más común brindada hoy por tus compañeros del grado escolar. Es decir, **para que sea más probable ganar el premio, debes responder según lo que tú crees que la mayoría de tus compañeros piensan.**

¿Cuántos de tus compañeros del grado aceptarían que uno de sus amigos cercanos fumara?

| *Todos mis compañeros* | *La mayoría de mis compañeros* | *Muchos de mis compañeros* | *Algunos de mis compañeros* | *Solo unos pocos de mis compañeros* | *Ninguno de mis compañeros* |
| --- | --- | --- | --- | --- | --- |
| 🞆 | 🞆 | 🞆 | 🞆 | 🞆 | 🞆 |

**Parte 3**

**Pregunta 2.**

¿Cuántos de tus compañeros del grado aceptarían que uno de tus amigos cercanos usara un cigarrillo electrónico?

*Indica qué proporción de estudiantes del grado crees que aceptaría que uno de sus amigos cercanos usara un cigarrillo electrónico: todos mis compañeros, la mayoría de mis compañeros, muchos de mis compañeros, algunos de mis compañeros, solo unos pocos de mis compañeros, ninguno de mis compañeros. Para indicar tu respuesta marca la casilla correspondiente.*

Recuerda que ganarás un premio ($15.000) en la tarjeta de regalo si tu respuesta a una pregunta seleccionada al azar coincide con la respuesta más común brindada hoy por tus compañeros del grado escolar. Es decir, **para que sea más probable ganar el premio, debes responder según lo que tú crees que la mayoría de tus compañeros piensan.**

¿Cuántos de tus compañeros del grado aceptarían que uno de tus amigos cercanos usara un cigarrillo electrónico?

| *Todos mis compañeros* | *La mayoría de mis compañeros* | *Muchos de mis compañeros* | *Algunos de mis compañeros* | *Solo unos pocos de mis compañeros* | *Ninguno de mis compañeros* |
| --- | --- | --- | --- | --- | --- |
| 🞆 | 🞆 | 🞆 | 🞆 | 🞆 | 🞆 |

**Este es el final de la Parte 3 del experimento**

Por favor, espera a que el encargado del salón te indique que puedes seguir con la Parte 4.

**Parte 4.**

Se te darán 10 fichas virtuales equivalentes a $10.000. Es decir, recibirás 10 fichas de $1.000 cada una. A continuación, tendrás la oportunidad de dar cualquier cantidad de tus $10.000 al programa Des-ahógate [reemplazar con el nombre del programa asignado a la institución Des-ahógate – EntreParceros. El programa de prevención Des-ahógate EntreParceros pretende evitar el consumo de tabaco entre adolescentes de tu edad.

Puedes dar cualquier cantidad de los $10.000 que recibiste a Des-ahógate - EntreParceros. Por ejemplo, puedes decidir dar $0 a Des-ahógate EntreParceros y mantener $10.000 para ti. O puedes decidir dar $10.000 a Des-ahógate EntreParceros y mantener $0 para ti. También puedes optar por dar cualquier otra cantidad entre $0 y $10.000 a Des-ahógate EntreParceros.

La cantidad de dinero que no le des a Des-ahógate EntreParceros hará parte de tus ganancias en esta parte. Recuerda que cada ficha equivale a $1.000.

¿Cuántas **fichas** quieres donar a Des-ahógate EntreParceros?

| 🞆 | 0 (tú ganas $10.000) |
| --- | --- |
| 🞆 | 1 (tú ganas $9.000) |
| 🞆 | 2 (tú ganas $8.000) |
| 🞆 | 3 (tú ganas $7.000) |
| 🞆 | 4 (tú ganas $6.000) |
| 🞆 | 5 (tú ganas $5.000) |
| 🞆 | 6 (tú ganas $4.000) |
| 🞆 | 7 (tú ganas $3.000) |
| 🞆 | 8 (tú ganas $2.000) |
| 🞆 | 9 (tú ganas $1.000) |
| 🞆 | 10 (tú ganas $0) |

Gracias por participar de este experimento.

Tus respuestas serán guardas para determinar tus ganancias.

Recuerda que en 16 semanas semanas volveremos para hacer otro estudio igual a este.

## Example syntax for longitudinal measurement invariance confirmatory factor analysis models, including data from MECHANISMS schools.

**Configural measurement invariance model**

model_Attitude_Configural <- 'Attitude =~ NA*AT1 + AT3 + AT4 + AT5 + AT6 + AT7 + AT8 + AT9 + AT10 + AT11 + AT12

FU_Attitude =~ NA*FU_AT1 + FU_AT3 + FU_AT4 + FU_AT5 + FU_AT6 + FU_AT7 + FU_AT8 + FU_AT9 + FU_AT10 + FU_AT11 + FU_AT12

Attitude ~~ FU_Attitude

AT1 ~~ FU_AT1

AT3 ~~ FU_AT3

AT4 ~~ FU_AT4

AT5 ~~ FU_AT5

AT6 ~~ FU_AT6

AT7 ~~ FU_AT7

AT8 ~~ FU_AT8

AT9 ~~ FU_AT9

AT10 ~~ FU_AT10

AT11 ~~ FU_AT11

AT12 ~~ FU_AT12

AT4 ~~ a*AT6

FU_AT4 ~~ a*FU_AT6

AT7 ~~ b*AT8

FU_AT7 ~~ b*FU_AT8

AT11 ~~ c*AT12

FU_AT11 ~~ c*FU_AT12

AT1 + AT3 + AT4 + AT5 + AT6 + AT7 + AT8 + AT9 + AT10 + AT11 + AT12 ~ 1

FU_AT1 + FU_AT3 + FU_AT4 + FU_AT5 + FU_AT6 + FU_AT7 + FU_AT8 + FU_AT9 + FU_AT10 + FU_AT11 + FU_AT12 ~ 1

Attitude ~~ 1*Attitude

FU_Attitude ~~ 1*FU_Attitude

AT1 ~~ AT1

AT3 ~~ AT3

AT4 ~~ AT4

AT5 ~~ AT5

AT6 ~~ AT6

AT7 ~~ AT7

AT8 ~~ AT8

AT9 ~~ AT9

AT10 ~~ AT10

AT11 ~~ AT11

AT12 ~~ AT12

FU_AT1 ~~ FU_AT1

FU_AT3 ~~ FU_AT3

FU_AT4 ~~ FU_AT4

FU_AT5 ~~ FU_AT5

FU_AT6 ~~ FU_AT6

FU_AT7 ~~ FU_AT7

FU_AT8 ~~ FU_AT8

FU_AT9 ~~ FU_AT9

FU_AT10 ~~ FU_AT10

FU_AT11 ~~ FU_AT11

FU_AT12 ~~ FU_AT12'

**Weak/metric measurement invariance model**

model_Attitude_Weak <- 'Attitude =~ a*AT1 + b*AT3 + c*AT4 + d*AT5 + e*AT6 + f*AT7 + g*AT8 + h*AT9 + i*AT10 + j*AT11 + k*AT12

FU_Attitude =~ a*FU_AT1 + b*FU_AT3 + c*FU_AT4 + d*FU_AT5 + e*FU_AT6 + f*FU_AT7 + g*FU_AT8 + h*FU_AT9 + i*FU_AT10 + j*FU_AT11 + k*FU_AT12

Attitude ~~ FU_Attitude

AT1 ~~ FU_AT1

AT3 ~~ FU_AT3

AT4 ~~ FU_AT4

AT5 ~~ FU_AT5

AT6 ~~ FU_AT6

AT7 ~~ FU_AT7

AT8 ~~ FU_AT8

AT9 ~~ FU_AT9

AT10 ~~ FU_AT10

AT11 ~~ FU_AT11

AT12 ~~ FU_AT12

AT4 ~~ l*AT6

FU_AT4 ~~ l*FU_AT6

AT7 ~~ m*AT8

FU_AT7 ~~ m*FU_AT8

AT11 ~~ n*AT12

FU_AT11 ~~ n*FU_AT12

AT1 + AT3 + AT4 + AT5 + AT6 + AT7 + AT8 + AT9 + AT10 + AT11 + AT12 ~ 1

FU_AT1 + FU_AT3 + FU_AT4 + FU_AT5 + FU_AT6 + FU_AT7 + FU_AT8 + FU_AT9 + FU_AT10 + FU_AT11 + FU_AT12 ~ 1

Attitude ~~ 1*Attitude

FU_Attitude ~~ 1*FU_Attitude

AT1 ~~ AT1

AT3 ~~ AT3

AT4 ~~ AT4

AT5 ~~ AT5

AT6 ~~ AT6

AT7 ~~ AT7

AT8 ~~ AT8

AT9 ~~ AT9

AT10 ~~ AT10

AT11 ~~ AT11

AT12 ~~ AT12

FU_AT1 ~~ FU_AT1

FU_AT3 ~~ FU_AT3

FU_AT4 ~~ FU_AT4

FU_AT5 ~~ FU_AT5

FU_AT6 ~~ FU_AT6

FU_AT7 ~~ FU_AT7

FU_AT8 ~~ FU_AT8

FU_AT9 ~~ FU_AT9

FU_AT10 ~~ FU_AT10

FU_AT11 ~~ FU_AT11

FU_AT12 ~~ FU_AT12'

**Strong/scalar measurement invariance model**

model_Attitude_Strong <- 'Attitude =~ a*AT1 + b*AT3 + c*AT4 + d*AT5 + e*AT6 + f*AT7 + g*AT8 + h*AT9 + i*AT10 + j*AT11 + k*AT12

FU_Attitude =~ a*FU_AT1 + b*FU_AT3 + c*FU_AT4 + d*FU_AT5 + e*FU_AT6 + f*FU_AT7 + g*FU_AT8 + h*FU_AT9 + i*FU_AT10 + j*FU_AT11 + k*FU_AT12

Attitude ~~ FU_Attitude

AT1 ~~ FU_AT1

AT3 ~~ FU_AT3

AT4 ~~ FU_AT4

AT5 ~~ FU_AT5

AT6 ~~ FU_AT6

AT7 ~~ FU_AT7

AT8 ~~ FU_AT8

AT9 ~~ FU_AT9

AT10 ~~ FU_AT10

AT11 ~~ FU_AT11

AT12 ~~ FU_AT12

AT4 ~~ l*AT6

FU_AT4 ~~ l*FU_AT6

AT7 ~~ m*AT8

FU_AT7 ~~ m*FU_AT8

AT11 ~~ n*AT12

FU_AT11 ~~ n*FU_AT12

AT1 + FU_AT1 ~ o*1

AT3 + FU_AT3 ~ p*1

AT4 + FU_AT4 ~ q*1

AT5 + FU_AT5 ~ r*1

AT6 + FU_AT6 ~ s*1

AT7 + FU_AT7 ~ t*1

AT8 + FU_AT8 ~ u*1

AT9 + FU_AT9 ~ v*1

AT10 + FU_AT10 ~ w*1

AT11 + FU_AT11 ~ x*1

AT12 + FU_AT12 ~ y*1

Attitude ~~ 1*Attitude

FU_Attitude ~~ 1*FU_Attitude

AT1 ~~ AT1

AT3 ~~ AT3

AT4 ~~ AT4

AT5 ~~ AT5

AT6 ~~ AT6

AT7 ~~ AT7

AT8 ~~ AT8

AT9 ~~ AT9

AT10 ~~ AT10

AT11 ~~ AT11

AT12 ~~ AT12

FU_AT1 ~~ FU_AT1

FU_AT3 ~~ FU_AT3

FU_AT4 ~~ FU_AT4

FU_AT5 ~~ FU_AT5

FU_AT6 ~~ FU_AT6

FU_AT7 ~~ FU_AT7

FU_AT8 ~~ FU_AT8

FU_AT9 ~~ FU_AT9

FU_AT10 ~~ FU_AT10

FU_AT11 ~~ FU_AT11

FU_AT12 ~~ FU_AT12'

**Strict/residual measurement invariance model**

model_Attitude_Strict <- 'Attitude =~ a*AT1 + b*AT3 + c*AT4 + d*AT5 + e*AT6 + f*AT7 + g*AT8 + h*AT9 + i*AT10 + j*AT11 + k*AT12

FU_Attitude =~ a*FU_AT1 + b*FU_AT3 + c*FU_AT4 + d*FU_AT5 + e*FU_AT6 + f*FU_AT7 + g*FU_AT8 + h*FU_AT9 + i*FU_AT10 + j*FU_AT11 + k*FU_AT12

Attitude ~~ FU_Attitude

AT1 ~~ FU_AT1

AT3 ~~ FU_AT3

AT4 ~~ FU_AT4

AT5 ~~ FU_AT5

AT6 ~~ FU_AT6

AT7 ~~ FU_AT7

AT8 ~~ FU_AT8

AT9 ~~ FU_AT9

AT10 ~~ FU_AT10

AT11 ~~ FU_AT11

AT12 ~~ FU_AT12

AT4 ~~ l*AT6

FU_AT4 ~~ l*FU_AT6

AT7 ~~ m*AT8

FU_AT7 ~~ m*FU_AT8

AT11 ~~ n*AT12

FU_AT11 ~~ n*FU_AT12

AT1 + FU_AT1 ~ o*1

AT3 + FU_AT3 ~ p*1

AT4 + FU_AT4 ~ q*1

AT5 + FU_AT5 ~ r*1

AT6 + FU_AT6 ~ s*1

AT7 + FU_AT7 ~ t*1

AT8 + FU_AT8 ~ u*1

AT9 + FU_AT9 ~ v*1

AT10 + FU_AT10 ~ w*1

AT11 + FU_AT11 ~ x*1

AT12 + FU_AT12 ~ y*1

Attitude ~~ 1*Attitude

FU_Attitude ~~ 1*FU_Attitude

AT1 ~~ z*AT1

AT3 ~~ aa*AT3

AT4 ~~ ab*AT4

AT5 ~~ ac*AT5

AT6 ~~ ad*AT6

AT7 ~~ ae*AT7

AT8 ~~ af*AT8

AT9 ~~ ag*AT9

AT10 ~~ ah*AT10

AT11 ~~ ai*AT11

AT12 ~~ aj*AT12

FU_AT1 ~~ z*FU_AT1

FU_AT3 ~~ aa*FU_AT3

FU_AT4 ~~ ab*FU_AT4

FU_AT5 ~~ ac*FU_AT5

FU_AT6 ~~ ad*FU_AT6

FU_AT7 ~~ ae*FU_AT7

FU_AT8 ~~ af*FU_AT8

FU_AT9 ~~ ag*FU_AT9

FU_AT10 ~~ ah*FU_AT10

FU_AT11 ~~ ai*FU_AT11

FU_AT12 ~~ aj*FU_AT12'

**Estimates and model fit statistics for each model**

model_Attitude_Configural_fit <- cfa(model = model_Attitude_Configural, data = data, std.lv = TRUE, estimator="MLR", missing="fiml", orthogonal = FALSE)

summary(model_Attitude_Configural_fit, fit.measures=TRUE, standardized=TRUE, rsquare=TRUE)

parameterEstimates(model_Attitude_Configural_fit, level=0.95, standardized = TRUE)

standardizedSolution(model_Attitude_Configural_fit, level=0.95, type = "std.all")

model_Attitude_Configural_fitMeasures <- fitMeasures(model_Attitude_Configural_fit)

model_Attitude_Weak_fit <- cfa(model = model_Attitude_Weak, data = data, std.lv = TRUE, estimator="MLR", missing="fiml", orthogonal = FALSE)

summary(model_Attitude_Weak_fit, fit.measures=TRUE, standardized=TRUE, rsquare=TRUE)

parameterEstimates(model_Attitude_Weak_fit, level=0.95, standardized = TRUE)

standardizedSolution(model_Attitude_Weak_fit, level=0.95, type = "std.all")

model_Attitude_Weak_fitMeasures <- fitMeasures(model_Attitude_Weak_fit)

model_Attitude_Strong_fit <- cfa(model = model_Attitude_Strong, data = data, std.lv = TRUE, estimator="MLR", missing="fiml", orthogonal = FALSE)

summary(model_Attitude_Strong_fit, fit.measures=TRUE, standardized=TRUE, rsquare=TRUE)

parameterEstimates(model_Attitude_Strong_fit, level=0.95, standardized = TRUE)

standardizedSolution(model_Attitude_Strong_fit, level=0.95, type = "std.all")

model_Attitude_Strong_fitMeasures <- fitMeasures(model_Attitude_Strong_fit)

model_Attitude_Strict_fit <- cfa(model = model_Attitude_Strict, data = data, std.lv = TRUE, estimator="MLR", missing="fiml", orthogonal = FALSE)

summary(model_Attitude_Strict_fit, fit.measures=TRUE, standardized=TRUE, rsquare=TRUE)

parameterEstimates(model_Attitude_Strict_fit, level=0.95, standardized = TRUE)

standardizedSolution(model_Attitude_Strict_fit, level=0.95, type = "std.all")

model_Attitude_Strict_fitMeasures <- fitMeasures(model_Attitude_Strict_fit)

**Scaled chi-square difference tests (log likelihood ratio tests) comparing the models**

anova(model_Attitude_Configural_fit, model_Attitude_Weak_fit, model_Attitude_Strong_fit, model_Attitude_Strict_fit)

**Comparison of Weak/metric measurement invariance model with Configural measurement invariance model**

anova(model_Attitude_Configural_fit, model_Attitude_Weak_fit)

model_Attitude_Weak_fitMeasures[["cfi.robust"]]-model_Attitude_Configural_fitMeasures[["cfi.robust"]]

model_Attitude_Weak_fitMeasures[["tli.robust"]]-model_Attitude_Configural_fitMeasures[["tli.robust"]]

model_Attitude_Weak_fitMeasures[["rmsea.robust"]]-model_Attitude_Configural_fitMeasures[["rmsea.robust"]]

model_Attitude_Weak_fitMeasures[["srmr"]]-model_Attitude_Configural_fitMeasures[["srmr"]]

model_Attitude_Weak_fitMeasures[["aic"]]-model_Attitude_Configural_fitMeasures[["aic"]]

model_Attitude_Weak_fitMeasures[["bic"]]-model_Attitude_Configural_fitMeasures[["bic"]]

model_Attitude_Weak_fitMeasures[["bic2"]]-model_Attitude_Configural_fitMeasures[["bic2"]]

**Comparison of Strong/scalar measurement invariance model with Weak/metric measurement invariance model**

anova(model_Attitude_Weak_fit, model_Attitude_Strong_fit)

model_Attitude_Strong_fitMeasures[["cfi.robust"]]-model_Attitude_Weak_fitMeasures[["cfi.robust"]]

model_Attitude_Strong_fitMeasures[["tli.robust"]]-model_Attitude_Weak_fitMeasures[["tli.robust"]]

model_Attitude_Strong_fitMeasures[["rmsea.robust"]]-model_Attitude_Weak_fitMeasures[["rmsea.robust"]]

model_Attitude_Strong_fitMeasures[["srmr"]]-model_Attitude_Weak_fitMeasures[["srmr"]]

model_Attitude_Strong_fitMeasures[["aic"]]-model_Attitude_Weak_fitMeasures[["aic"]]

model_Attitude_Strong_fitMeasures[["bic"]]-model_Attitude_Weak_fitMeasures[["bic"]]

model_Attitude_Strong_fitMeasures[["bic2"]]-model_Attitude_Weak_fitMeasures[["bic2"]]

**Comparison of Strict/residual measurement invariance model with Strong/scalar measurement invariance model**

anova(model_Attitude_Strong_fit, model_Attitude_Strict_fit)

model_Attitude_Strict_fitMeasures[["cfi.robust"]]-model_Attitude_Strong_fitMeasures[["cfi.robust"]]

model_Attitude_Strict_fitMeasures[["tli.robust"]]-model_Attitude_Strong_fitMeasures[["tli.robust"]]

model_Attitude_Strict_fitMeasures[["rmsea.robust"]]-model_Attitude_Strong_fitMeasures[["rmsea.robust"]]

model_Attitude_Strict_fitMeasures[["srmr"]]-model_Attitude_Strong_fitMeasures[["srmr"]]

model_Attitude_Strict_fitMeasures[["aic"]]-model_Attitude_Strong_fitMeasures[["aic"]]

model_Attitude_Strict_fitMeasures[["bic"]]-model_Attitude_Strong_fitMeasures[["bic"]]

model_Attitude_Strict_fitMeasures[["bic2"]]-model_Attitude_Strong_fitMeasures[["bic2"]]

**Key**

| model_Attitude_Configural | CFA model examining configural measurement invariance (i.e., whether the constructs have the same pattern of free and fixed loadings across timepoints). |
| --- | --- |
| model_Attitude_Weak | CFA model examining weak/metric measurement invariance (i.e., whether the item loadings on the factors are equivalent across timepoints, meaning that the latent factor has the same interpretation at baseline and follow-up). |
| model_Attitude_Strong | CFA model examining strong/scalar measurement invariance (i.e., whether the item loadings and intercepts are equivalent across timepoints, meaning that latent means can be compared between baseline and follow-up). |
| model_Attitude_Strict | CFA model examining strict/residual measurement invariance (i.e., whether the item loadings, intercepts, and residual variances are equivalent across timepoints). |
| Attitude | Latent variable representing attitudes towards smoking at baseline, derived from items AT1 to AT12, excluding AT2. |
| FU_Attitude | Latent variable representing attitudes towards smoking at follow-up, derived from items FU_AT1 to FU_AT12, excluding FU_AT2. |
| AT1 | Participants' observed scores for item AT1 at baseline (similar definitions apply for items AT3 to AT12). |
| FU_AT1 | Participants' observed scores for item AT1 at follow-up (similar definitions apply for items FU_AT3 to FU_AT12). |
| =~ | Latent variable operator, indicates “is measured by” (lavaan package) (24). |
| ~~ | Covariance operator, indicates “is correlated with” (lavaan package) (24). |
| ~1 | Intercept operator, defines the intercept (lavaan package) (24). |
| * | Used to define parameter labels and specify constraints. E.g., “a*AT1” and “a*FU_AT1” indicates “constrain the estimated factor loadings for AT1 and FU_AT1 to be equal”. |
| NA* | By default, the first factor loading would be constrained to 1 whilst the variance would be unconstrained. We invert these settings by specifying 'NA*' before the first indicator of each latent factor in the configural model and constraining the variance of each latent variable to 1. This is similar to specifying "std.lv=TRUE" in the call of the 'cfa' function (see below). This allows us to more easily constrain the factor loadings to equality in the weak/metric, strong/scalar, and strict/residual models. |
| cfa | Function to fit a confirmatory factor analysis (CFA) model (lavaan package) (24). |
| std.lv | Unstandardized parameter estimates (standardizes all latent variables in the model only). |
| std.all | Standardized parameter estimates (standardizes all latent and observed variables in the model). |
| MLR | Robust maximum-likelihood estimator. |
| fiml | Full-information maximum likelihood. |
| orthogonal | Specifying “orthogonal = FALSE” in the “cfa” call indicates that latent variables should be allowed to covary in the model when no other relationship is specified between them. |
| summary | Function to return a summary of the estimated parameters and model fit statistics (lavaan package) (24). |
| parameterEstimates | Function to return the unstandardized parameter estimates, standard errors, p-values, and confidence intervals (lavaan package) (24). |
| standardizedSolution | Function to return the standardized parameter estimates, standard errors, p-values, and confidence intervals (lavaan package) (24). |
| fitMeasures | Function to return the model fit statistics for each CFA model (lavaan package) (24). |
| anova | Function to perform an analysis of variance (ANOVA) test. Used to perform the log likelihood ratio tests comparing the chi-square test statistic (overall model fit) between configural, weak/metric, strong/scalar, and strict/residual measurement invariance CFA models (scaled chi-square difference tests). |
| cfi.robust | Robust Comparative Fit Index (CFI). |
| tli.robust | Robust Tucker-Lewis Index (TLI). |
| rmsea.robust | Robust Root Mean Square Error of Approximation (RMSEA). |
| srmr | Standardized Root Mean Square Residual (SRMR). |
| aic | Akaike Information Criterion (AIC). |
| bic | Bayesian Information Criterion (BIC). |
| bic2 | Sample size adjusted Bayesian Information Criterion (BIC). |

Note: The results of these models are reported at the top of Supplementary Table S7.

## Example syntax for confirmatory factor analysis models examining measurement invariance across settings or intervention groups, including data from MECHANISMS schools.

**Configural measurement invariance model**

model_Attitude_Configural <- 'Attitude =~ c(a1,a2)*AT1 + c(b1,b2)*AT3 + c(c1,c2)*AT4 + c(d1,d2)*AT5 + c(e1,e2)*AT6 + c(f1,f2)*AT7 + c(g1,g2)*AT8 + c(h1,h2)*AT9 + c(i1,i2)*AT10 + c(j1,j2)*AT11 + c(k1,k2)*AT12

FU_Attitude =~ c(a1,a2)*FU_AT1 + c(b1,b2)*FU_AT3 + c(c1,c2)*FU_AT4 + c(d1,d2)*FU_AT5 + c(e1,e2)*FU_AT6 + c(f1,f2)*FU_AT7 + c(g1,g2)*FU_AT8 + c(h1,h2)*FU_AT9 + c(i1,i2)*FU_AT10 + c(j1,j2)*FU_AT11 + c(k1,k2)*FU_AT12

Attitude ~~ FU_Attitude

AT1 ~~ FU_AT1

AT3 ~~ FU_AT3

AT4 ~~ FU_AT4

AT5 ~~ FU_AT5

AT6 ~~ FU_AT6

AT7 ~~ FU_AT7

AT8 ~~ FU_AT8

AT9 ~~ FU_AT9

AT10 ~~ FU_AT10

AT11 ~~ FU_AT11

AT12 ~~ FU_AT12

AT4 ~~ c(l1,l2)*AT6

FU_AT4 ~~ c(l1,l2)*FU_AT6

AT7 ~~ c(m1,m2)*AT8

FU_AT7 ~~ c(m1,m2)*FU_AT8

AT11 ~~ c(n1,n2)*AT12

FU_AT11 ~~ c(n1,n2)*FU_AT12

AT1 + FU_AT1 ~ c(o1,o2)*1

AT3 + FU_AT3 ~ c(p1,p2)*1

AT4 + FU_AT4 ~ c(q1,q2)*1

AT5 + FU_AT5 ~ c(r1,r2)*1

AT6 + FU_AT6 ~ c(s1,s2)*1

AT7 + FU_AT7 ~ c(t1,t2)*1

AT8 + FU_AT8 ~ c(u1,u2)*1

AT9 + FU_AT9 ~ c(v1,v2)*1

AT10 + FU_AT10 ~ c(w1,w2)*1

AT11 + FU_AT11 ~ c(x1,x2)*1

AT12 + FU_AT12 ~ c(y1,y2)*1

Attitude ~~ 1*Attitude

FU_Attitude ~~ 1*FU_Attitude

AT1 ~~ c(z1,z2)*AT1

AT3 ~~ c(aa1,aa2)*AT3

AT4 ~~ c(ab1,ab2)*AT4

AT5 ~~ c(ac1,ac2)*AT5

AT6 ~~ c(ad1,ad2)*AT6

AT7 ~~ c(ae1,ae2)*AT7

AT8 ~~ c(af1,af2)*AT8

AT9 ~~ c(ag1,ag2)*AT9

AT10 ~~ c(ah1,ah2)*AT10

AT11 ~~ c(ai1,ai2)*AT11

AT12 ~~ c(aj1,aj2)*AT12

FU_AT1 ~~ c(z1,z2)*FU_AT1

FU_AT3 ~~ c(aa1,aa2)*FU_AT3

FU_AT4 ~~ c(ab1,ab2)*FU_AT4

FU_AT5 ~~ c(ac1,ac2)*FU_AT5

FU_AT6 ~~ c(ad1,ad2)*FU_AT6

FU_AT7 ~~ c(ae1,ae2)*FU_AT7

FU_AT8 ~~ c(af1,af2)*FU_AT8

FU_AT9 ~~ c(ag1,ag2)*FU_AT9

FU_AT10 ~~ c(ah1,ah2)*FU_AT10

FU_AT11 ~~ c(ai1,ai2)*FU_AT11

FU_AT12 ~~ c(aj1,aj2)*FU_AT12'

**Weak/metric measurement invariance model**

model_Attitude_Weak <- 'Attitude =~ a*AT1 + b*AT3 + c*AT4 + d*AT5 + e*AT6 + f*AT7 + g*AT8 + h*AT9 + i*AT10 + j*AT11 + k*AT12

FU_Attitude =~ a*FU_AT1 + b*FU_AT3 + c*FU_AT4 + d*FU_AT5 + e*FU_AT6 + f*FU_AT7 + g*FU_AT8 + h*FU_AT9 + i*FU_AT10 + j*FU_AT11 + k*FU_AT12

Attitude ~~ FU_Attitude

AT1 ~~ FU_AT1

AT3 ~~ FU_AT3

AT4 ~~ FU_AT4

AT5 ~~ FU_AT5

AT6 ~~ FU_AT6

AT7 ~~ FU_AT7

AT8 ~~ FU_AT8

AT9 ~~ FU_AT9

AT10 ~~ FU_AT10

AT11 ~~ FU_AT11

AT12 ~~ FU_AT12

AT4 ~~ c(l1,l2)*AT6

FU_AT4 ~~ c(l1,l2)*FU_AT6

AT7 ~~ c(m1,m2)*AT8

FU_AT7 ~~ c(m1,m2)*FU_AT8

AT11 ~~ c(n1,n2)*AT12

FU_AT11 ~~ c(n1,n2)*FU_AT12

AT1 + FU_AT1 ~ c(o1,o2)*1

AT3 + FU_AT3 ~ c(p1,p2)*1

AT4 + FU_AT4 ~ c(q1,q2)*1

AT5 + FU_AT5 ~ c(r1,r2)*1

AT6 + FU_AT6 ~ c(s1,s2)*1

AT7 + FU_AT7 ~ c(t1,t2)*1

AT8 + FU_AT8 ~ c(u1,u2)*1

AT9 + FU_AT9 ~ c(v1,v2)*1

AT10 + FU_AT10 ~ c(w1,w2)*1

AT11 + FU_AT11 ~ c(x1,x2)*1

AT12 + FU_AT12 ~ c(y1,y2)*1

Attitude ~~ 1*Attitude

FU_Attitude ~~ 1*FU_Attitude

AT1 ~~ c(z1,z2)*AT1

AT3 ~~ c(aa1,aa2)*AT3

AT4 ~~ c(ab1,ab2)*AT4

AT5 ~~ c(ac1,ac2)*AT5

AT6 ~~ c(ad1,ad2)*AT6

AT7 ~~ c(ae1,ae2)*AT7

AT8 ~~ c(af1,af2)*AT8

AT9 ~~ c(ag1,ag2)*AT9

AT10 ~~ c(ah1,ah2)*AT10

AT11 ~~ c(ai1,ai2)*AT11

AT12 ~~ c(aj1,aj2)*AT12

FU_AT1 ~~ c(z1,z2)*FU_AT1

FU_AT3 ~~ c(aa1,aa2)*FU_AT3

FU_AT4 ~~ c(ab1,ab2)*FU_AT4

FU_AT5 ~~ c(ac1,ac2)*FU_AT5

FU_AT6 ~~ c(ad1,ad2)*FU_AT6

FU_AT7 ~~ c(ae1,ae2)*FU_AT7

FU_AT8 ~~ c(af1,af2)*FU_AT8

FU_AT9 ~~ c(ag1,ag2)*FU_AT9

FU_AT10 ~~ c(ah1,ah2)*FU_AT10

FU_AT11 ~~ c(ai1,ai2)*FU_AT11

FU_AT12 ~~ c(aj1,aj2)*FU_AT12'

**Strong/scalar measurement invariance model**

model_Attitude_Strong <- 'Attitude =~ a*AT1 + b*AT3 + c*AT4 + d*AT5 + e*AT6 + f*AT7 + g*AT8 + h*AT9 + i*AT10 + j*AT11 + k*AT12

FU_Attitude =~ a*FU_AT1 + b*FU_AT3 + c*FU_AT4 + d*FU_AT5 + e*FU_AT6 + f*FU_AT7 + g*FU_AT8 + h*FU_AT9 + i*FU_AT10 + j*FU_AT11 + k*FU_AT12

Attitude ~~ FU_Attitude

AT1 ~~ FU_AT1

AT3 ~~ FU_AT3

AT4 ~~ FU_AT4

AT5 ~~ FU_AT5

AT6 ~~ FU_AT6

AT7 ~~ FU_AT7

AT8 ~~ FU_AT8

AT9 ~~ FU_AT9

AT10 ~~ FU_AT10

AT11 ~~ FU_AT11

AT12 ~~ FU_AT12

AT4 ~~ c(l1,l2)*AT6

FU_AT4 ~~ c(l1,l2)*FU_AT6

AT7 ~~ c(m1,m2)*AT8

FU_AT7 ~~ c(m1,m2)*FU_AT8

AT11 ~~ c(n1,n2)*AT12

FU_AT11 ~~ c(n1,n2)*FU_AT12

AT1 + FU_AT1 ~ o*1

AT3 + FU_AT3 ~ p*1

AT4 + FU_AT4 ~ q*1

AT5 + FU_AT5 ~ r*1

AT6 + FU_AT6 ~ s*1

AT7 + FU_AT7 ~ t*1

AT8 + FU_AT8 ~ u*1

AT9 + FU_AT9 ~ v*1

AT10 + FU_AT10 ~ w*1

AT11 + FU_AT11 ~ x*1

AT12 + FU_AT12 ~ y*1

Attitude ~~ 1*Attitude

FU_Attitude ~~ 1*FU_Attitude

AT1 ~~ c(z1,z2)*AT1

AT3 ~~ c(aa1,aa2)*AT3

AT4 ~~ c(ab1,ab2)*AT4

AT5 ~~ c(ac1,ac2)*AT5

AT6 ~~ c(ad1,ad2)*AT6

AT7 ~~ c(ae1,ae2)*AT7

AT8 ~~ c(af1,af2)*AT8

AT9 ~~ c(ag1,ag2)*AT9

AT10 ~~ c(ah1,ah2)*AT10

AT11 ~~ c(ai1,ai2)*AT11

AT12 ~~ c(aj1,aj2)*AT12

FU_AT1 ~~ c(z1,z2)*FU_AT1

FU_AT3 ~~ c(aa1,aa2)*FU_AT3

FU_AT4 ~~ c(ab1,ab2)*FU_AT4

FU_AT5 ~~ c(ac1,ac2)*FU_AT5

FU_AT6 ~~ c(ad1,ad2)*FU_AT6

FU_AT7 ~~ c(ae1,ae2)*FU_AT7

FU_AT8 ~~ c(af1,af2)*FU_AT8

FU_AT9 ~~ c(ag1,ag2)*FU_AT9

FU_AT10 ~~ c(ah1,ah2)*FU_AT10

FU_AT11 ~~ c(ai1,ai2)*FU_AT11

FU_AT12 ~~ c(aj1,aj2)*FU_AT12'

**Estimates and model fit statistics for each model**

model_Attitude_Configural_fit <- cfa(model = model_Attitude_Configural, data = data, std.lv = TRUE, estimator="MLR", missing="fiml", orthogonal = FALSE, group = "Setting")

summary(model_Attitude_Configural_fit, fit.measures=TRUE, standardized=TRUE, rsquare=TRUE)

parameterEstimates(model_Attitude_Configural_fit, level=0.95, standardized = TRUE)

standardizedSolution(model_Attitude_Configural_fit, level=0.95, type = "std.all")

model_Attitude_Configural_fitMeasures <- fitMeasures(model_Attitude_Configural_fit)

model_Attitude_Weak_fit <- cfa(model = model_Attitude_Weak, data = data, std.lv = TRUE, estimator="MLR", missing="fiml", orthogonal = FALSE, group = "Setting")

summary(model_Attitude_Weak_fit, fit.measures=TRUE, standardized=TRUE, rsquare=TRUE)

parameterEstimates(model_Attitude_Weak_fit, level=0.95, standardized = TRUE)

standardizedSolution(model_Attitude_Weak_fit, level=0.95, type = "std.all")

model_Attitude_Weak_fitMeasures <- fitMeasures(model_Attitude_Weak_fit)

model_Attitude_Strong_fit <- cfa(model = model_Attitude_Strong, data = data, std.lv = TRUE, estimator="MLR", missing="fiml", orthogonal = FALSE, group = "Setting")

summary(model_Attitude_Strong_fit, fit.measures=TRUE, standardized=TRUE, rsquare=TRUE)

parameterEstimates(model_Attitude_Strong_fit, level=0.95, standardized = TRUE)

standardizedSolution(model_Attitude_Strong_fit, level=0.95, type = "std.all")

model_Attitude_Strong_fitMeasures <- fitMeasures(model_Attitude_Strong_fit)

**Scaled chi-square difference tests (log likelihood ratio tests) comparing the models**

anova(model_Attitude_Configural_fit, model_Attitude_Weak_fit, model_Attitude_Strong_fit)

**Comparison of Weak/metric measurement invariance model with Configural measurement invariance model**

anova(model_Attitude_Configural_fit, model_Attitude_Weak_fit)

model_Attitude_Weak_fitMeasures[["cfi.robust"]]-model_Attitude_Configural_fitMeasures[["cfi.robust"]]

model_Attitude_Weak_fitMeasures[["tli.robust"]]-model_Attitude_Configural_fitMeasures[["tli.robust"]]

model_Attitude_Weak_fitMeasures[["rmsea.robust"]]-model_Attitude_Configural_fitMeasures[["rmsea.robust"]]

model_Attitude_Weak_fitMeasures[["srmr"]]-model_Attitude_Configural_fitMeasures[["srmr"]]

model_Attitude_Weak_fitMeasures[["aic"]]-model_Attitude_Configural_fitMeasures[["aic"]]

model_Attitude_Weak_fitMeasures[["bic"]]-model_Attitude_Configural_fitMeasures[["bic"]]

model_Attitude_Weak_fitMeasures[["bic2"]]-model_Attitude_Configural_fitMeasures[["bic2"]]

**Comparison of Strong/scalar measurement invariance model with Weak/metric measurement invariance model**

anova(model_Attitude_Weak_fit, model_Attitude_Strong_fit)

model_Attitude_Strong_fitMeasures[["cfi.robust"]]-model_Attitude_Weak_fitMeasures[["cfi.robust"]]

model_Attitude_Strong_fitMeasures[["tli.robust"]]-model_Attitude_Weak_fitMeasures[["tli.robust"]]

model_Attitude_Strong_fitMeasures[["rmsea.robust"]]-model_Attitude_Weak_fitMeasures[["rmsea.robust"]]

model_Attitude_Strong_fitMeasures[["srmr"]]-model_Attitude_Weak_fitMeasures[["srmr"]]

model_Attitude_Strong_fitMeasures[["aic"]]-model_Attitude_Weak_fitMeasures[["aic"]]

model_Attitude_Strong_fitMeasures[["bic"]]-model_Attitude_Weak_fitMeasures[["bic"]]

model_Attitude_Strong_fitMeasures[["bic2"]]-model_Attitude_Weak_fitMeasures[["bic2"]]

**Key**

| model_Attitude_Configural | CFA model examining configural measurement invariance (i.e., whether the constructs have the same pattern of free and fixed loadings between NI and Bogotá). |
| --- | --- |
| model_Attitude_Weak | CFA model examining weak/metric measurement invariance (i.e., whether the item loadings on the factors are equivalent between NI and Bogotá, meaning that the latent factor has the same interpretation for NI and Bogotá). |
| model_Attitude_Strong | CFA model examining strong/scalar measurement invariance (i.e., whether the item loadings and intercepts are equivalent between NI and Bogotá, meaning that latent means can be compared between NI and Bogotá). |
| Attitude | Latent variable representing attitudes towards smoking at baseline, derived from items AT1 to AT12, excluding AT2. |
| FU_Attitude | Latent variable representing attitudes towards smoking at follow-up, derived from items FU_AT1 to FU_AT12, excluding FU_AT2. |
| AT1 | Participants' observed scores for item AT1 at baseline (similar definitions apply for items AT3 to AT12). |
| FU_AT1 | Participants' observed scores for item AT1 at follow-up (similar definitions apply for items FU_AT3 to FU_AT12). |
| =~ | Latent variable operator, indicates “is measured by” (lavaan package) (24). |
| ~~ | Covariance operator, indicates “is correlated with” (lavaan package) (24). |
| ~1 | Intercept operator, defines the intercept (lavaan package) (24). |
| * | Used to define parameter labels and specify constraints. E.g., “a*AT1” and “a*FU_AT1” indicates “constrain the estimated factor loadings for AT1 and FU_AT1 to be equal in NI and Bogotá”. “c(a1,a2)*AT1” and “c(a1,a2)*FU_AT1” indicates “constrain the estimated factor loadings for AT1 and FU_AT1 to be equal but allow them to vary between NI and Bogotá”. |
| cfa | Function to fit a confirmatory factor analysis (CFA) model (lavaan package) (24). |
| std.lv | Unstandardized parameter estimates (standardizes all latent variables in the model only). |
| group | Used to specify the grouping variable. |
| std.all | Standardized parameter estimates (standardizes all latent and observed variables in the model). |
| MLR | Robust maximum-likelihood estimator. |
| fiml | Full-information maximum likelihood. |
| orthogonal | Specifying “orthogonal = FALSE” in the “cfa” call indicates that latent variables should be allowed to covary in the model when no other relationship is specified between them. |
| summary | Function to return a summary of the estimated parameters and model fit statistics (lavaan package) (24). |
| parameterEstimates | Function to return the unstandardized parameter estimates, standard errors, p-values, and confidence intervals (lavaan package) (24). |
| standardizedSolution | Function to return the standardized parameter estimates, standard errors, p-values, and confidence intervals (lavaan package) (24). |
| fitMeasures | Function to return the model fit statistics for each CFA model (lavaan package) (24). |
| anova | Function to perform an analysis of variance (ANOVA) test. Used to perform the log likelihood ratio tests comparing the chi-square test statistic (overall model fit) between configural, weak/metric, and strong/scalar measurement invariance CFA models (scaled chi-square difference tests). |
| cfi.robust | Robust Comparative Fit Index (CFI). |
| tli.robust | Robust Tucker-Lewis Index (TLI). |
| rmsea.robust | Robust Root Mean Square Error of Approximation (RMSEA). |
| srmr | Standardized Root Mean Square Residual (SRMR). |
| aic | Akaike Information Criterion (AIC). |
| bic | Bayesian Information Criterion (BIC). |
| bic2 | Sample size adjusted Bayesian Information Criterion (BIC). |

Note: The results of these models are reported at the top of Supplementary Table S9. The syntax also applies for the models reported at the top of Supplementary Table S11 when specifying group="Intervention" in the 'cfa' call.

## Example syntax for structural equation models with intervention group (ASSIST versus Dead Cool) and setting (Bogotá versus Northern Ireland) as predictor variables, including data from MECHANISMS schools.

model1 <- 'FU_Donation ~ Intervention + Setting + Donation + Gender + Age1 + Age2 + Ethnicity + SES1 + SES2'

set.seed(1234)

fit1 <- sem(model = model1, data = data, std.lv = TRUE, estimator="MLR", missing="fiml")

summary(fit1, fit.measures=TRUE, standardized=TRUE, rsquare=TRUE)

parameterEstimates(fit1, level=0.95, standardized = TRUE)

standardizedSolution(fit1, type="std.all", level=0.95, se=TRUE)

fitMeasures(fit1)

model2 <- 'FU_Susceptibility ~ Intervention + Setting + Susceptibility + Gender + Age1 + Age2 + Ethnicity + SES1 + SES2'

set.seed(1234)

fit2 <- sem(model = model2, data = data, std.lv = TRUE, ordered = c("FU_Susceptibility"), estimator="WLSMV")

summary(fit2, fit.measures=TRUE, standardized=TRUE, rsquare=TRUE)

parameterEstimates(fit2, level=0.95, standardized = TRUE)

standardizedSolution(fit2, type="std.all", level=0.95, se=TRUE)

fitMeasures(fit2)

**Key**

| FU_Donation | Participants' responses to the experimental outcome (Donation to ASSIST/Dead Cool) at follow-up. |
| --- | --- |
| Intervention | Dummy variable representing intervention group (0=Dead Cool, 1=ASSIST). |
| Setting | Dummy variable representing setting (0=Northern Ireland, 1=Bogotá). |
| Donation | Participants' responses to the experimental outcome (Donation to ASSIST/Dead Cool) at baseline. |
| Gender | Dummy variable representing participant gender (0=boy; 1=girl/prefer not to say). |
| Age1 | Dummy variable representing participant age (1=13 years; 0=otherwise). Base category=12 years or less. |
| Age2 | Dummy variable representing participant age (1=14 years or more; 0=otherwise). Base category=12 years or less. |
| Ethnicity | Dummy variable representing participant ethnicity (0=No ethnic minority; 1=ethnic minority). |
| SES1 | Dummy variable representing participant socio-economic status (1=Middle-Low/Middle [296.6<NIMDM2017≤593.2 in NI]; 0=otherwise). Base category=Informal settlement/Lowest/Low [NIMDM2017≤296.6 in NI]. |
| SES2 | Dummy variable representing participant socio-economic status (1=Middle-High/High [NIMDM2017>593.2 in NI]; 0=otherwise). Base category=Informal settlement/Lowest/Low [NIMDM2017≤296.6 in NI]. |
| FU_Susceptibility | Participants' responses to the smoking susceptibility outcome at follow-up (binary outcome variable). |
| Susceptibility | Participants' responses to the smoking susceptibility outcome at baseline. |
| ~ | Regression operator, indicates “is regressed on” (lavaan package) (24). |
| sem | Function to fit a structural equation model (lavaan package) (24). |
| std.lv | Unstandardized parameter estimates (standardizes all latent variables in the model only). |
| std.all | Standardized parameter estimates (standardizes all latent and observed variables in the model). |
| MLR | Robust maximum-likelihood estimator. |
| WLSMV | Weighted least square mean and variance adjusted estimator. |
| fiml | Full-information maximum likelihood. |
| ordered | Specifying “ordered = c("FU_Susceptibility")” in the “sem” call for “model2” indicates that “FU_Susceptibility” should be treated as a categorical outcome variable. |
| summary | Function to return a summary of the estimated parameters and model fit statistics (lavaan package) (24). |
| parameterEstimates | Function to return the unstandardized parameter estimates, standard errors, p-values, and confidence intervals (lavaan package) (24). |
| standardizedSolution | Function to return the standardized parameter estimates, standard errors, p-values, and confidence intervals (lavaan package) (24). |
| fitMeasures | Function to return the model fit statistics (lavaan package) (24). |

Note: The results of these models are reported in Table 3 and Supplementary Table S15.

## Example syntax for structural equation models with predictor variables comparing ASSIST non-peer supporters and ASSIST peer supporters separately with Dead Cool, including data from MECHANISMS schools.

model <- 'FU_Donation ~ Intervention1 + Intervention2 + Setting + Donation + Gender + Age1 + Age2 + Ethnicity + SES1 + SES2'

set.seed(1234)

fit <- sem(model = model, data = data, std.lv = TRUE, estimator="MLR", missing="fiml")

summary(fit, fit.measures=TRUE, standardized=TRUE, rsquare=TRUE)

parameterEstimates(fit, level=0.95, standardized = TRUE)

standardizedSolution(fit, type="std.all", level=0.95, se=TRUE)

fitMeasures(fit)

**Key**

| FU_Donation | Participants' responses to the experimental outcome (Donation to ASSIST/Dead Cool) at follow-up. |
| --- | --- |
| Intervention1 | Dummy variable representing intervention group (1=ASSIST non-peer supporters; 0=otherwise). Base category=Dead Cool. |
| Intervention2 | Dummy variable representing intervention group (1=ASSIST peer supporters; 0=otherwise). Base category=Dead Cool. |
| Setting | Dummy variable representing setting (0=Northern Ireland, 1=Bogotá). |
| Donation | Participants' responses to the experimental outcome (Donation to ASSIST/Dead Cool) at baseline. |
| Gender | Dummy variable representing participant gender (0=boy; 1=girl/prefer not to say). |
| Age1 | Dummy variable representing participant age (1=13 years; 0=otherwise). Base category=12 years or less. |
| Age2 | Dummy variable representing participant age (1=14 years or more; 0=otherwise). Base category=12 years or less. |
| Ethnicity | Dummy variable representing participant ethnicity (0=No ethnic minority; 1=ethnic minority). |
| SES1 | Dummy variable representing participant socio-economic status (1=Middle-Low/Middle [296.6<NIMDM2017≤593.2 in NI]; 0=otherwise). Base category=Informal settlement/Lowest/Low [NIMDM2017≤296.6 in NI]. |
| SES2 | Dummy variable representing participant socio-economic status (1=Middle-High/High [NIMDM2017>593.2 in NI]; 0=otherwise). Base category=Informal settlement/Lowest/Low [NIMDM2017≤296.6 in NI]. |
| ~ | Regression operator, indicates “is regressed on” (lavaan package) (24). |
| sem | Function to fit a structural equation model (lavaan package) (24). |
| std.lv | Unstandardized parameter estimates (standardizes all latent variables in the model only). |
| std.all | Standardized parameter estimates (standardizes all latent and observed variables in the model). |
| MLR | Robust maximum-likelihood estimator. |
| fiml | Full-information maximum likelihood. |
| summary | Function to return a summary of the estimated parameters and model fit statistics (lavaan package) (24). |
| parameterEstimates | Function to return the unstandardized parameter estimates, standard errors, p-values, and confidence intervals (lavaan package) (24). |
| standardizedSolution | Function to return the standardized parameter estimates, standard errors, p-values, and confidence intervals (lavaan package) (24). |
| fitMeasures | Function to return the model fit statistics (lavaan package) (24). |

Note: The results of this model are reported in the first row of Table 4 and Supplementary Table S16.

## Example syntax for structural equation models with intervention × setting interactions, including data from MECHANISMS schools.

model <- 'FU_Donation ~ Intervention + Setting + Interaction + Donation + Gender + Age1 + Age2 + Ethnicity + SES1 + SES2'

set.seed(1234)

fit <- sem(model = model, data = data, std.lv = TRUE, estimator="MLR", missing="fiml")

summary(fit, fit.measures=TRUE, standardized=TRUE, rsquare=TRUE)

parameterEstimates(fit, level=0.95, standardized = TRUE)

standardizedSolution(fit, type="std.all", level=0.95, se=TRUE)

fitMeasures(fit)

**Key**

| FU_Donation | Participants' responses to the experimental outcome (Donation to ASSIST/Dead Cool) at follow-up. |
| --- | --- |
| Intervention | Dummy variable representing intervention group (0=Dead Cool, 1=ASSIST). |
| Setting | Dummy variable representing setting (0=Northern Ireland, 1=Bogotá). |
| Interaction | Variable computed as the product of the Intervention and Setting variables. |
| Donation | Participants' responses to the experimental outcome (Donation to ASSIST/Dead Cool) at baseline. |
| Gender | Dummy variable representing participant gender (0=boy; 1=girl/prefer not to say). |
| Age1 | Dummy variable representing participant age (1=13 years; 0=otherwise). Base category=12 years or less. |
| Age2 | Dummy variable representing participant age (1=14 years or more; 0=otherwise). Base category=12 years or less. |
| Ethnicity | Dummy variable representing participant ethnicity (0=No ethnic minority; 1=ethnic minority). |
| SES1 | Dummy variable representing participant socio-economic status (1=Middle-Low/Middle [296.6<NIMDM2017≤593.2 in NI]; 0=otherwise). Base category=Informal settlement/Lowest/Low [NIMDM2017≤296.6 in NI]. |
| SES2 | Dummy variable representing participant socio-economic status (1=Middle-High/High [NIMDM2017>593.2 in NI]; 0=otherwise). Base category=Informal settlement/Lowest/Low [NIMDM2017≤296.6 in NI]. |
| ~ | Regression operator, indicates “is regressed on” (lavaan package) (24). |
| sem | Function to fit a structural equation model (lavaan package) (24). |
| std.lv | Unstandardized parameter estimates (standardizes all latent variables in the model only). |
| std.all | Standardized parameter estimates (standardizes all latent and observed variables in the model). |
| MLR | Robust maximum-likelihood estimator. |
| fiml | Full-information maximum likelihood. |
| summary | Function to return a summary of the estimated parameters and model fit statistics (lavaan package) (24). |
| parameterEstimates | Function to return the unstandardized parameter estimates, standard errors, p-values, and confidence intervals (lavaan package) (24). |
| standardizedSolution | Function to return the standardized parameter estimates, standard errors, p-values, and confidence intervals (lavaan package) (24). |
| fitMeasures | Function to return the model fit statistics (lavaan package) (24). |

Note: The results of this model are reported in Table 5 and Supplementary Table S17.

## Example syntax for structural equation models with intervention × setting interactions comparing ASSIST non-peer supporters and ASSIST peer supporters separately with Dead Cool, including data from MECHANISMS schools.

model <- 'FU_Donation ~ Intervention1 + Intervention2 + Setting + Interaction1 + Interaction2 + Donation + Gender + Age1 + Age2 + Ethnicity + SES1 + SES2'

set.seed(1234)

fit <- sem(model = model, data = data, std.lv = TRUE, estimator="MLR", missing="fiml")

summary(fit, fit.measures=TRUE, standardized=TRUE, rsquare=TRUE)

parameterEstimates(fit, level=0.95, standardized = TRUE)

standardizedSolution(fit, type="std.all", level=0.95, se=TRUE)

fitMeasures(fit)

**Key**

| FU_Donation | Participants' responses to the experimental outcome (Donation to ASSIST/Dead Cool) at follow-up. |
| --- | --- |
| Intervention1 | Dummy variable representing intervention group (1=ASSIST non-peer supporters; 0=otherwise). Base category=Dead Cool. |
| Intervention2 | Dummy variable representing intervention group (1=ASSIST peer supporters; 0=otherwise). Base category=Dead Cool. |
| Setting | Dummy variable representing setting (0=Northern Ireland, 1=Bogotá). |
| Interaction1 | Variable computed as the product of the Intervention1 and Setting variables. |
| Interaction2 | Variable computed as the product of the Intervention2 and Setting variables. |
| Donation | Participants' responses to the experimental outcome (Donation to ASSIST/Dead Cool) at baseline. |
| Gender | Dummy variable representing participant gender (0=boy; 1=girl/prefer not to say). |
| Age1 | Dummy variable representing participant age (1=13 years; 0=otherwise). Base category=12 years or less. |
| Age2 | Dummy variable representing participant age (1=14 years or more; 0=otherwise). Base category=12 years or less. |
| Ethnicity | Dummy variable representing participant ethnicity (0=No ethnic minority; 1=ethnic minority). |
| SES1 | Dummy variable representing participant socio-economic status (1=Middle-Low/Middle [296.6<NIMDM2017≤593.2 in NI]; 0=otherwise). Base category=Informal settlement/Lowest/Low [NIMDM2017≤296.6 in NI]. |
| SES2 | Dummy variable representing participant socio-economic status (1=Middle-High/High [NIMDM2017>593.2 in NI]; 0=otherwise). Base category=Informal settlement/Lowest/Low [NIMDM2017≤296.6 in NI]. |
| ~ | Regression operator, indicates “is regressed on” (lavaan package) (24). |
| sem | Function to fit a structural equation model (lavaan package) (24). |
| std.lv | Unstandardized parameter estimates (standardizes all latent variables in the model only). |
| std.all | Standardized parameter estimates (standardizes all latent and observed variables in the model). |
| MLR | Robust maximum-likelihood estimator. |
| fiml | Full-information maximum likelihood. |
| summary | Function to return a summary of the estimated parameters and model fit statistics (lavaan package) (24). |
| parameterEstimates | Function to return the unstandardized parameter estimates, standard errors, p-values, and confidence intervals (lavaan package) (24). |
| standardizedSolution | Function to return the standardized parameter estimates, standard errors, p-values, and confidence intervals (lavaan package) (24). |
| fitMeasures | Function to return the model fit statistics (lavaan package) (24). |

Note: The results of this model are reported in the first row of Table 6 and Supplementary Table S20.

## Example syntax for structural equation models including predictor variables comparing MECHANISMS setting/intervention groups with the Northern Ireland control group (sensitivity analyses).

model <- 'FU_Smoking ~ Group1 + Group2 + Group3 + Group4 + Smoking + Gender + Age1 + Age2 + Ethnicity + SES1 + SES2'

set.seed(1234)

fit <- sem(model = model, data = data, std.lv = TRUE, estimator="MLR", missing="fiml")

summary(fit, fit.measures=TRUE, standardized=TRUE, rsquare=TRUE)

parameterEstimates(fit, level=0.95, standardized = TRUE)

standardizedSolution(fit, type="std.all", level=0.95, se=TRUE)

fitMeasures(fit)

**Key**

| FU_Smoking | Participants' responses to self-report smoking behavior at follow-up. |
| --- | --- |
| Group1 | Dummy variable representing setting/intervention group (1=Northern Ireland Dead Cool; 0=otherwise). Base category=Northern Ireland Control group. |
| Group2 | Dummy variable representing setting/intervention group (1=Northern Ireland ASSIST; 0=otherwise). Base category=Northern Ireland Control group. |
| Group3 | Dummy variable representing setting/intervention group (1=Bogotá Dead Cool; 0=otherwise). Base category=Northern Ireland Control group. |
| Group4 | Dummy variable representing setting/intervention group (1=Bogotá ASSIST; 0=otherwise). Base category=Northern Ireland Control group. |
| Smoking | Participants' responses to self-report smoking behavior at baseline. |
| Gender | Dummy variable representing participant gender (0=boy; 1=girl/prefer not to say). |
| Age1 | Dummy variable representing participant age (1=13 years; 0=otherwise). Base category=12 years or less. |
| Age2 | Dummy variable representing participant age (1=14 years or more; 0=otherwise). Base category=12 years or less. |
| Ethnicity | Dummy variable representing participant ethnicity (0=No ethnic minority; 1=ethnic minority). |
| SES1 | Dummy variable representing participant socio-economic status (1=Middle-Low/Middle [296.6<NIMDM2017≤593.2 in NI]; 0=otherwise). Base category=Informal settlement/Lowest/Low [NIMDM2017≤296.6 in NI]. |
| SES2 | Dummy variable representing participant socio-economic status (1=Middle-High/High [NIMDM2017>593.2 in NI]; 0=otherwise). Base category=Informal settlement/Lowest/Low [NIMDM2017≤296.6 in NI]. |
| ~ | Regression operator, indicates “is regressed on” (lavaan package) (24). |
| sem | Function to fit a structural equation model (lavaan package) (24). |
| std.lv | Unstandardized parameter estimates (standardizes all latent variables in the model only). |
| std.all | Standardized parameter estimates (standardizes all latent and observed variables in the model). |
| MLR | Robust maximum-likelihood estimator. |
| fiml | Full-information maximum likelihood. |
| summary | Function to return a summary of the estimated parameters and model fit statistics (lavaan package) (24). |
| parameterEstimates | Function to return the unstandardized parameter estimates, standard errors, p-values, and confidence intervals (lavaan package) (24). |
| standardizedSolution | Function to return the standardized parameter estimates, standard errors, p-values, and confidence intervals (lavaan package) (24). |
| fitMeasures | Function to return the model fit statistics (lavaan package) (24). |

Note: The results of this model are reported in the first row of Table 10 and Supplementary Table S68.

## Example syntax for structural equation models including predictor variables comparing MECHANISMS setting/intervention groups with the Northern Ireland control group, with ASSIST non-peer supporters and ASSIST peer supporters analyzed separately (sensitivity analyses).

model <- 'FU_Smoking ~ Group1 + Group2 + Group3 + Group4 + Group5 + Group6 + Smoking + Gender + Age1 + Age2 + Ethnicity + SES1 + SES2'

set.seed(1234)

fit <- sem(model = model, data = data, std.lv = TRUE, estimator="MLR", missing="fiml")

summary(fit, fit.measures=TRUE, standardized=TRUE, rsquare=TRUE)

parameterEstimates(fit, level=0.95, standardized = TRUE)

standardizedSolution(fit, type="std.all", level=0.95, se=TRUE)

fitMeasures(fit)

**Key**

| FU_Smoking | Participants' responses to self-report smoking behavior at follow-up. |
| --- | --- |
| Group1 | Dummy variable representing setting/intervention group (1=Northern Ireland Dead Cool; 0=otherwise). Base category=Northern Ireland Control group. |
| Group2 | Dummy variable representing setting/intervention group (1=Northern Ireland ASSIST non-peer supporters; 0=otherwise). Base category=Northern Ireland Control group. |
| Group3 | Dummy variable representing setting/intervention group (1=Northern Ireland ASSIST peer supporters; 0=otherwise). Base category=Northern Ireland Control group. |
| Group4 | Dummy variable representing setting/intervention group (1=Bogotá Dead Cool; 0=otherwise). Base category=Northern Ireland Control group. |
| Group5 | Dummy variable representing setting/intervention group (1=Bogotá ASSIST non-peer supporters; 0=otherwise). Base category=Northern Ireland Control group. |
| Group6 | Dummy variable representing setting/intervention group (1=Bogotá ASSIST peer supporters; 0=otherwise). Base category=Northern Ireland Control group. |
| Smoking | Participants' responses to self-report smoking behavior at baseline. |
| Gender | Dummy variable representing participant gender (0=boy; 1=girl/prefer not to say). |
| Age1 | Dummy variable representing participant age (1=13 years; 0=otherwise). Base category=12 years or less. |
| Age2 | Dummy variable representing participant age (1=14 years or more; 0=otherwise). Base category=12 years or less. |
| Ethnicity | Dummy variable representing participant ethnicity (0=No ethnic minority; 1=ethnic minority). |
| SES1 | Dummy variable representing participant socio-economic status (1=Middle-Low/Middle [296.6<NIMDM2017≤593.2 in NI]; 0=otherwise). Base category=Informal settlement/Lowest/Low [NIMDM2017≤296.6 in NI]. |
| SES2 | Dummy variable representing participant socio-economic status (1=Middle-High/High [NIMDM2017>593.2 in NI]; 0=otherwise). Base category=Informal settlement/Lowest/Low [NIMDM2017≤296.6 in NI]. |
| ~ | Regression operator, indicates “is regressed on” (lavaan package) (24). |
| sem | Function to fit a structural equation model (lavaan package) (24). |
| std.lv | Unstandardized parameter estimates (standardizes all latent variables in the model only). |
| std.all | Standardized parameter estimates (standardizes all latent and observed variables in the model). |
| MLR | Robust maximum-likelihood estimator. |
| fiml | Full-information maximum likelihood. |
| summary | Function to return a summary of the estimated parameters and model fit statistics (lavaan package) (24). |
| parameterEstimates | Function to return the unstandardized parameter estimates, standard errors, p-values, and confidence intervals (lavaan package) (24). |
| standardizedSolution | Function to return the standardized parameter estimates, standard errors, p-values, and confidence intervals (lavaan package) (24). |
| fitMeasures | Function to return the model fit statistics (lavaan package) (24). |

Note: The results of this model are reported in the first row of Table 11 and Supplementary Table S69.

## Example syntax for power calculations for SEM predictor models using WebPower's "linear regression" method.

Example 1: wp.regression(n = nobs, p1 = 9, p2 = 7, f2 = max(0, EffectSize_f2), alpha = 0.05, power = NULL)

Example 2: wp.regression(n = nobs, p1 = 10, p2 = 7, f2 = max(0, EffectSize_f2), alpha = 0.05, power = NULL)

Example 3: wp.regression(n = nobs, p1 = 12, p2 = 7, f2 = max(0, EffectSize_f2), alpha = 0.05, power = NULL)

Example 4: wp.regression(n = nobs, p1 = 8, p2 = 7, f2 = max(0, EffectSize_f2), alpha = 0.05, power = NULL)

Example 5: wp.regression(n = nobs, p1 = 11, p2 = 7, f2 = max(0, EffectSize_f2), alpha = 0.05, power = NULL)

Example 6: wp.regression(n = nobs, p1 = 13, p2 = 7, f2 = max(0, EffectSize_f2), alpha = 0.05, power = NULL)

Example 7: wp.regression(n = 1000, p1 = 9, p2 = 7, f2 = NULL, alpha = c(0.01, 0.05), power = 0.8)

**Key**

| wp.regression | Function to calculate power using WebPower's "linear regression" method (WebPower package) (37). |
| --- | --- |
| nobs | Number of observations reported in SEM predictor model output. |
| p1 | Number of parameters in full SEM predictor model (including all predictors). |
| p2 | Number of parameters in reduced SEM predictor model (including baseline values of the outcome variable, Gender, Age1, Age2, Ethnicity, SES1, and SES2 as covariates only). |
| EffectSize_f2 | Effect size calculated from the R-squared statistics reported in the full and reduced SEM predictor model outputs as $\frac{R_{Full}^{2} -R_{Reduced}^{2}}{1 -R_{Full}^{2}}$. |

Note: Example 1 relates to power calculations for the models with observed outcome variables in Table 3 and Supplementary Table S21. Example 2 relates to power calculations for the models with observed outcome variables in Tables 4 and 5. Example 3 relates to power calculations for the models with observed outcome variables in Table 6. Example 4 relates to power calculations for the models with observed outcome variables in Supplementary Table S18. Example 5 relates to power calculations for the models with observed outcome variables in Table 10. Example 6 relates to power calculations for the models with observed outcome variables in Table 11. The results of Example 7 are reported in the first two rows of Supplementary Table S13. Example 7 relates to calculating the minimum effect size that can be detected at different sample sizes and significance levels with 80% power.

## Example syntax for power calculations for SEM predictor models using WebPower's "power analysis based on RMSEA" method.

Example 1: wp.sem.rmsea(n = nobs, df = dof, rmsea0 = RMSEA_Reduced, rmsea1 = RMSEA_Full, power = NULL, alpha = 0.05)

Example 2: wp.sem.rmsea(n = nobs, df = dof, rmsea0 = RMSEA_Reduced, rmsea1 = NULL, power = 0.8, alpha = c(0.01, 0.05))

**Key**

| wp.sem.rmsea | Function to calculate power using WebPower's "power analysis based on RMSEA" method (WebPower package) (37). |
| --- | --- |
| nobs | Number of observations reported in SEM predictor model output. |
| dof | Degrees of freedom for the chi-square test reported in SEM predictor model output. |
| RMSEA_Reduced | RMSEA from full SEM predictor model (including all predictors). |
| RMSEA_Full | RMSEA from reduced SEM predictor model (including baseline values of the outcome variable, Gender, Age1, Age2, Ethnicity, SES1, and SES2 as covariates only). |

Note: Example 1 relates to power calculations for the models with latent outcome variables in Tables 3 to 6, and Supplementary Tables S18 and S21. Example 2 relates to calculating the minimum effect size that can be detected at different sample sizes and significance levels with 80% power (reported in Supplementary Table S14).

## Example syntax for single mediator models with intervention group (ASSIST versus Dead Cool) as the predictor variable, including data from MECHANISMS schools.

model1 <- 'FU_Donation ~ B*FU_Smoking + CP*Intervention + Donation + Smoking + Setting + Gender + Age1 + Age2 + Ethnicity + SES1 + SES2

FU_Smoking ~ A*Intervention + Donation + Smoking + Setting + Gender + Age1 + Age2 + Ethnicity + SES1 + SES2

AB := A * B

C := CP + AB'

set.seed(1234)

fit1 <- sem(model = model1, data = data, std.lv = TRUE, estimator="ML", missing="fiml", se = "bootstrap", bootstrap = 10000, test = "yuan.bentler")

summary(fit1, fit.measures=TRUE, standardized=TRUE, rsquare=TRUE)

parameterEstimates(fit1, level=0.95, boot.ci.type = "bca.simple", standardized = TRUE)

standardizedSolution_boot_ci(fit1, level=0.95, type="std.all")

fitMeasures(fit1)

model2 <- 'FU_Susceptibility ~ B*FU_Knowledge + CP*Intervention + Susceptibility + Knowledge + Setting + Gender + Age1 + Age2 + Ethnicity + SES1 + SES2

FU_Knowledge ~ A*Intervention + Susceptibility + Knowledge + Setting + Gender + Age1 + Age2 + Ethnicity + SES1 + SES2

AB := A * B

C := CP + AB'

set.seed(1234)

fit2 <- sem(model = model2, data = data, std.lv = TRUE, ordered=c("FU_Susceptibility"), estimator="DWLS", se = "bootstrap", bootstrap = 10000, test = "scaled.shifted")

summary(fit2, fit.measures=TRUE, standardized=TRUE, rsquare=TRUE)

parameterEstimates(fit2, level=0.95, boot.ci.type = "bca.simple", standardized = TRUE)

standardizedSolution_boot_ci(fit2, level=0.95, type="std.all")

fitMeasures(fit2)

**Key**

| FU_Donation | Participants' responses to the experimental outcome (Donation to ASSIST/Dead Cool) at follow-up (outcome variable in model1). |
| --- | --- |
| FU_Smoking | Participants' responses to the self-report smoking behavior outcome at follow-up (mediator variable in model1). |
| Intervention | Dummy variable representing intervention group (0=Dead Cool, 1=ASSIST; predictor variable). |
| Donation | Participants' responses to the experimental outcome (Donation to ASSIST/Dead Cool) at baseline. |
| Smoking | Participants' responses to the self-report smoking behavior outcome at baseline. |
| Setting | Dummy variable representing setting (0=Northern Ireland, 1=Bogotá). |
| Gender | Dummy variable representing participant gender (0=boy; 1=girl/prefer not to say). |
| Age1 | Dummy variable representing participant age (1=13 years; 0=otherwise). Base category=12 years or less. |
| Age2 | Dummy variable representing participant age (1=14 years or more; 0=otherwise). Base category=12 years or less. |
| Ethnicity | Dummy variable representing participant ethnicity (0=No ethnic minority; 1=ethnic minority). |
| SES1 | Dummy variable representing participant socio-economic status (1=Middle-Low/Middle [296.6<NIMDM2017≤593.2 in NI]; 0=otherwise). Base category=Informal settlement/Lowest/Low [NIMDM2017≤296.6 in NI]. |
| SES2 | Dummy variable representing participant socio-economic status (1=Middle-High/High [NIMDM2017>593.2 in NI]; 0=otherwise). Base category=Informal settlement/Lowest/Low [NIMDM2017≤296.6 in NI]. |
| FU_Susceptibility | Participants' responses to the smoking susceptibility outcome at follow-up (binary outcome variable in model2). |
| FU_Knowledge | Participants' responses to the self-report knowledge of smoking outcome at follow-up (mediator variable in model2). |
| Susceptibility | Participants' responses to the smoking susceptibility outcome at baseline. |
| Knowledge | Participants' responses to the self-report knowledge of smoking outcome at baseline. |
| ~ | Regression operator, indicates “is regressed on” (lavaan package) (24). |
| := | Defined parameter operator (lavaan package) (24). |
| A | Coefficient representing the predictor variable (Intervention) effect on the mediator variable (FU_Smoking in model1, FU_Knowledge in model2). |
| B | Coefficient representing the association of the mediator variable (FU_Smoking in model1, FU_Knowledge in model2) with the outcome variable (FU_Donation in model1, FU_Susceptibility in model2). |
| AB | Coefficient representing the indirect effect of the predictor variable on the outcome variable via the mediator (i.e., the product of the A and B coefficients). |
| CP | Coefficient representing the direct effect (i.e., the effect of the predictor variable on the outcome variable adjusted for the mediator). |
| C | Coefficient representing the total effect (i.e., the effect of the predictor variable on the outcome variable unadjusted for the mediator). |
| sem | Function to fit a structural equation model (lavaan package) (24). |
| test = "yuan.bentler" | Requests the robust versions of the model fit statistics, similar to the robust maximum likelihood (MLR) estimator. |
| test = "scaled.shifted" | Requests the robust versions of the model fit statistics, similar to the weighted least square mean and variance adjusted (WLSMV) estimator. |
| std.lv | Unstandardized parameter estimates (standardizes all latent variables in the model only). |
| std.all | Standardized parameter estimates (standardizes all latent and observed variables in the model). |
| ML | Maximum-likelihood estimator. |
| DWLS | Diagonally weighted least squares estimator. |
| fiml | Full-information maximum likelihood. |
| ordered | Specifying “ordered = c("FU_Susceptibility")” in the “sem” call for “model2” indicates that “FU_Susceptibility” should be treated as a categorical outcome variable. |
| parameterEstimates | Function to report parameter estimates for a structural equation model (lavaan package) (24). In this example, bias-corrected bootstrap standard errors are requested (with 10,000 iterations). |
| standardizedSolution_boot_ci | Function to form bootstrap confidence intervals for the standardized solution (lavaan package) (24). |
| fitMeasures | Function to return the model fit statistics (lavaan package) (24). |

Note: The results of model1 are reported in the first row of Supplementary Tables S24 and S25. The results of model2 are reported in the first row of Supplementary Tables S32 and S33.

## Example syntax for single mediator models with predictor variables comparing ASSIST non-peer supporters and ASSIST peer supporters separately with Dead Cool, including data from MECHANISMS schools.

model <- 'FU_Donation ~ B*FU_Smoking + CP1*Intervention1 + CP2*Intervention2 + Donation + Smoking + Setting + Gender + Age1 + Age2 + Ethnicity + SES1 + SES2

FU_Smoking ~ A1*Intervention1 + A2*Intervention2 + Donation + Smoking + Setting + Gender + Age1 + Age2 + Ethnicity + SES1 + SES2

A1B := A1 * B

A2B := A2 * B

C1 := CP1 + A1B

C2 := CP2 + A2B

AB := A1B + A2B

CP := CP1 + CP2

C := C1 + C2'

set.seed(1234)

fit <- sem(model = model, data = data, std.lv = TRUE, estimator="ML", missing="fiml", se = "bootstrap", bootstrap = 10000, test = "yuan.bentler")

summary(fit, fit.measures=TRUE, standardized=TRUE, rsquare=TRUE)

parameterEstimates(fit, level=0.95, boot.ci.type = "bca.simple", standardized = TRUE)

standardizedSolution_boot_ci(fit, level=0.95, type="std.all")

fitMeasures(fit)

**Key**

| FU_Donation | Participants' responses to the experimental outcome (Donation to ASSIST/Dead Cool) at follow-up (outcome variable). |
| --- | --- |
| FU_Smoking | Participants' responses to the self-report smoking behavior outcome at follow-up (mediator variable). |
| Intervention1 | Dummy variable representing intervention group (1=ASSIST non-peer supporters; 0=otherwise). Base category=Dead Cool (predictor variable 1). |
| Intervention2 | Dummy variable representing intervention group (1=ASSIST peer supporters; 0=otherwise). Base category=Dead Cool (predictor variable 2). |
| Donation | Participants' responses to the experimental outcome (Donation to ASSIST/Dead Cool) at baseline. |
| Smoking | Participants' responses to the self-report smoking behavior outcome at baseline. |
| Setting | Dummy variable representing setting (0=Northern Ireland, 1=Bogotá). |
| Gender | Dummy variable representing participant gender (0=boy; 1=girl/prefer not to say). |
| Age1 | Dummy variable representing participant age (1=13 years; 0=otherwise). Base category=12 years or less. |
| Age2 | Dummy variable representing participant age (1=14 years or more; 0=otherwise). Base category=12 years or less. |
| Ethnicity | Dummy variable representing participant ethnicity (0=No ethnic minority; 1=ethnic minority). |
| SES1 | Dummy variable representing participant socio-economic status (1=Middle-Low/Middle [296.6<NIMDM2017≤593.2 in NI]; 0=otherwise). Base category=Informal settlement/Lowest/Low [NIMDM2017≤296.6 in NI]. |
| SES2 | Dummy variable representing participant socio-economic status (1=Middle-High/High [NIMDM2017>593.2 in NI]; 0=otherwise). Base category=Informal settlement/Lowest/Low [NIMDM2017≤296.6 in NI]. |
| ~ | Regression operator, indicates “is regressed on” (lavaan package) (24). |
| := | Defined parameter operator (lavaan package) (24). |
| A1 | Coefficient representing the effect of ASSIST non-peer supporters compared to Dead Cool on the mediator variable (FU_Smoking). |
| A2 | Coefficient representing the effect of ASSIST peer supporters compared to Dead Cool on the mediator variable (FU_Smoking). |
| B | Coefficient representing the association of the mediator variable (FU_Smoking) with the outcome variable (FU_Donation). |
| A1B | The indirect effect of ASSIST non-peer supporters compared to Dead Cool on the outcome variable via the mediator (i.e., the product of the A1 and B coefficients). |
| A2B | The indirect effect of ASSIST peer supporters compared to Dead Cool on the outcome variable via the mediator (i.e., the product of the A2 and B coefficients). |
| AB | The total indirect effect of ASSIST non-peer supporters and ASSIST peer supporters compared to Dead Cool on the outcome variable via the mediator (i.e., the sum of A1B and A2B). |
| CP1 | Coefficient representing the direct effect of ASSIST non-peer supporters compared to Dead Cool (i.e., the effect of predictor variable 1 on the outcome variable adjusted for the mediator). |
| CP2 | Coefficient representing the direct effect of ASSIST peer supporters compared to Dead Cool (i.e., the effect of the predictor variable 2 on the outcome variable adjusted for the mediator). |
| CP | The direct effect of ASSIST non-peer supporters and ASSIST peer supporters compared to Dead Cool on the outcome variable adjusted for the mediator (i.e., the sum of CP1 and CP2). |
| C1 | Coefficient representing the total effect of ASSIST non-peer supporters compared to Dead Cool (i.e., the effect of predictor variable 1 on the outcome variable unadjusted for the mediator). |
| C2 | Coefficient representing the total effect of ASSIST peer supporters compared to Dead Cool (i.e., the effect of the predictor variable 2 on the outcome variable unadjusted for the mediator). |
| C | The total effect of ASSIST non-peer supporters and ASSIST peer supporters compared to Dead Cool on the outcome variable unadjusted for the mediator (i.e., the sum of C1 and C2). |
| sem | Function to fit a structural equation model (lavaan package) (24). |
| test = "yuan.bentler" | Requests the robust versions of the model fit statistics, similar to the robust maximum likelihood (MLR) estimator. |
| std.lv | Unstandardized parameter estimates (standardizes all latent variables in the model only). |
| std.all | Standardized parameter estimates (standardizes all latent and observed variables in the model). |
| ML | Maximum-likelihood estimator. |
| fiml | Full-information maximum likelihood. |
| parameterEstimates | Function to report parameter estimates for a structural equation model (lavaan package) (24). In this example, bias-corrected bootstrap standard errors are requested (with 10,000 iterations). |
| standardizedSolution_boot_ci | Function to form bootstrap confidence intervals for the standardized solution (lavaan package) (24). |
| fitMeasures | Function to return the model fit statistics (lavaan package) (24). |

Note: The results of this model are reported in the first row of Supplementary Tables S39 and S40.

## Example syntax for single mediator models including predictor variables comparing MECHANISMS setting/intervention groups with the Northern Ireland control group (sensitivity analyses).

model <- 'FU_Smoking ~ B*FU_Intentions + CP1*Group1 + CP2*Group2 + CP3*Group3 + CP4*Group4 + Smoking + Intentions + Gender + Age1 + Age2 + Ethnicity + SES1 + SES2

FU_Intentions ~ A1*Group1 + A2*Group2 + A3*Group3 + A4*Group4 + Smoking + Intentions + Gender + Age1 + Age2 + Ethnicity + SES1 + SES2

A1B := A1 * B

A2B := A2 * B

A3B := A3 * B

A4B := A4 * B

C1 := CP1 + A1B

C2 := CP2 + A2B

C3 := CP3 + A3B

C4 := CP4 + A4B

AB := A1B + A2B + A3B + A4B

CP := CP1 + CP2 + CP3 + CP4

C := C1 + C2 + C3 + C4'

set.seed(1234)

fit <- sem(model = model, data = data, std.lv = TRUE, estimator="ML", missing="fiml", se = "bootstrap", bootstrap = 10000, test = "yuan.bentler")

summary(fit, fit.measures=TRUE, standardized=TRUE, rsquare=TRUE)

parameterEstimates(fit, level=0.95, boot.ci.type = "bca.simple", standardized = TRUE)

standardizedSolution_boot_ci(fit, level=0.95, type="std.all")

fitMeasures(fit)

**Key**

| FU_Smoking | Participants' responses to the self-report smoking behavior outcome at follow-up (outcome variable). |
| --- | --- |
| FU_Intentions | Participants' responses to the self-report smoking intentions outcome at follow-up (mediator variable). |
| Group1 | Dummy variable representing setting/intervention group (1=Northern Ireland Dead Cool; 0=otherwise). Base category=Northern Ireland Control group (predictor variable 1). |
| Group2 | Dummy variable representing setting/intervention group (1=Northern Ireland ASSIST; 0=otherwise). Base category=Northern Ireland Control group (predictor variable 2). |
| Group3 | Dummy variable representing setting/intervention group (1=Bogotá Dead Cool; 0=otherwise). Base category=Northern Ireland Control group (predictor variable 3). |
| Group4 | Dummy variable representing setting/intervention group (1=Bogotá ASSIST; 0=otherwise). Base category=Northern Ireland Control group (predictor variable 4). |
| Smoking | Participants' responses to the self-report smoking behavior outcome at baseline. |
| Intentions | Participants' responses to the self-report smoking intentions outcome at baseline. |
| Gender | Dummy variable representing participant gender (0=boy; 1=girl/prefer not to say). |
| Age1 | Dummy variable representing participant age (1=13 years; 0=otherwise). Base category=12 years or less. |
| Age2 | Dummy variable representing participant age (1=14 years or more; 0=otherwise). Base category=12 years or less. |
| Ethnicity | Dummy variable representing participant ethnicity (0=No ethnic minority; 1=ethnic minority). |
| SES1 | Dummy variable representing participant socio-economic status (1=Middle-Low/Middle [296.6<NIMDM2017≤593.2 in NI]; 0=otherwise). Base category=Informal settlement/Lowest/Low [NIMDM2017≤296.6 in NI]. |
| SES2 | Dummy variable representing participant socio-economic status (1=Middle-High/High [NIMDM2017>593.2 in NI]; 0=otherwise). Base category=Informal settlement/Lowest/Low [NIMDM2017≤296.6 in NI]. |
| ~ | Regression operator, indicates “is regressed on” (lavaan package) (24). |
| := | Defined parameter operator (lavaan package) (24). |
| A1 | Coefficient representing the effect of Northern Ireland Dead Cool compared to the Northern Ireland Control group on the mediator variable (FU_Intentions). |
| A2 | Coefficient representing the effect of Northern Ireland ASSIST compared to the Northern Ireland Control group on the mediator variable (FU_Intentions). |
| A3 | Coefficient representing the effect of Bogotá Dead Cool compared to the Northern Ireland Control group on the mediator variable (FU_Intentions). |
| A4 | Coefficient representing the effect of Bogotá ASSIST compared to the Northern Ireland Control group on the mediator variable (FU_Intentions). |
| B | Coefficient representing the association of the mediator variable (FU_Intentions) with the outcome variable (FU_Smoking). |
| A1B | The indirect effect of Northern Ireland Dead Cool compared to the Northern Ireland Control group on the outcome variable via the mediator (i.e., the product of the A1 and B coefficients). |
| A2B | The indirect effect of Northern Ireland ASSIST compared to the Northern Ireland Control group on the outcome variable via the mediator (i.e., the product of the A2 and B coefficients). |
| A3B | The indirect effect of Bogotá Dead Cool compared to the Northern Ireland Control group on the outcome variable via the mediator (i.e., the product of the A3 and B coefficients). |
| A4B | The indirect effect of Bogotá ASSIST compared to the Northern Ireland Control group on the outcome variable via the mediator (i.e., the product of the A4 and B coefficients). |
| AB | The total indirect effect of Northern Ireland Dead Cool, Northern Ireland ASSIST, Bogotá Dead Cool, and Bogotá ASSIST compared to the Northern Ireland Control group on the outcome variable via the mediator (i.e., the sum of A1B, A2B, A3B, and A4B). |
| CP1 | Coefficient representing the direct effect of Northern Ireland Dead Cool compared to the Northern Ireland Control group (i.e., the effect of predictor variable 1 on the outcome variable adjusted for the mediator). |
| CP2 | Coefficient representing the direct effect of Northern Ireland ASSIST compared to the Northern Ireland Control group (i.e., the effect of predictor variable 2 on the outcome variable adjusted for the mediator). |
| CP3 | Coefficient representing the direct effect of Bogotá Dead Cool compared to the Northern Ireland Control group (i.e., the effect of predictor variable 3 on the outcome variable adjusted for the mediator). |
| CP4 | Coefficient representing the direct effect of Bogotá ASSIST compared to the Northern Ireland Control group (i.e., the effect of predictor variable 4 on the outcome variable adjusted for the mediator). |
| CP | The direct effect of Northern Ireland Dead Cool, Northern Ireland ASSIST, Bogotá Dead Cool, and Bogotá ASSIST compared to the Northern Ireland Control group on the outcome variable adjusted for the mediator (i.e., the sum of CP1, CP2, CP3, and CP4). |
| C1 | Coefficient representing the total effect of Northern Ireland Dead Cool compared to the Northern Ireland Control group (i.e., the effect of predictor variable 1 on the outcome variable unadjusted for the mediator). |
| C2 | Coefficient representing the total effect of Northern Ireland ASSIST compared to the Northern Ireland Control group (i.e., the effect of predictor variable 2 on the outcome variable unadjusted for the mediator). |
| C3 | Coefficient representing the total effect of Bogotá Dead Cool compared to the Northern Ireland Control group (i.e., the effect of predictor variable 3 on the outcome variable unadjusted for the mediator). |
| C4 | Coefficient representing the total effect of Bogotá ASSIST compared to the Northern Ireland Control group (i.e., the effect of predictor variable 4 on the outcome variable unadjusted for the mediator). |
| C | The total effect of Northern Ireland Dead Cool, Northern Ireland ASSIST, Bogotá Dead Cool, and Bogotá ASSIST compared to the Northern Ireland Control group on the outcome variable unadjusted for the mediator (i.e., the sum of C1, C2, C3, and C4). |
| sem | Function to fit a structural equation model (lavaan package) (24). |
| test = "yuan.bentler" | Requests the robust versions of the model fit statistics, similar to the robust maximum likelihood (MLR) estimator. |
| std.lv | Unstandardized parameter estimates (standardizes all latent variables in the model only). |
| std.all | Standardized parameter estimates (standardizes all latent and observed variables in the model). |
| ML | Maximum-likelihood estimator. |
| fiml | Full-information maximum likelihood. |
| parameterEstimates | Function to report parameter estimates for a structural equation model (lavaan package) (24). In this example, bias-corrected bootstrap standard errors are requested (with 10,000 iterations). |
| standardizedSolution_boot_ci | Function to form bootstrap confidence intervals for the standardized solution (lavaan package) (24). |
| fitMeasures | Function to return the model fit statistics (lavaan package) (24). |

Note: The results of this model are reported at the top of Supplementary Tables S72 and S73.

## Example syntax for single mediator models including predictor variables comparing MECHANISMS setting/intervention groups with the Northern Ireland control group, with ASSIST non-peer supporters and ASSIST peer supporters analyzed separately (sensitivity analyses).

model <- 'FU_Smoking ~ B*FU_Intentions + CP1*Group1 + CP2*Group2 + CP3*Group3 + CP4*Group4 + CP5*Group5 + CP6*Group6 + Smoking + Intentions + Gender + Age1 + Age2 + Ethnicity + SES1 + SES2

FU_Intentions ~ A1*Group1 + A2*Group2 + A3*Group3 + A4*Group4 + A5*Group5 + A6*Group6 + Smoking + Intentions + Gender + Age1 + Age2 + Ethnicity + SES1 + SES2

A1B := A1 * B

A2B := A2 * B

A3B := A3 * B

A4B := A4 * B

A5B := A5 * B

A6B := A6 * B

C1 := CP1 + A1B

C2 := CP2 + A2B

C3 := CP3 + A3B

C4 := CP4 + A4B

C5 := CP5 + A5B

C6 := CP6 + A6B

AB := A1B + A2B + A3B + A4B + A5B + A6B

CP := CP1 + CP2 + CP3 + CP4 + CP5 + CP6

C := C1 + C2 + C3 + C4 + C5 + C6'

set.seed(1234)

fit <- sem(model = model, data = data, std.lv = TRUE, estimator="ML", missing="fiml", se = "bootstrap", bootstrap = 10000, test = "yuan.bentler")

summary(fit, fit.measures=TRUE, standardized=TRUE, rsquare=TRUE)

parameterEstimates(fit, level=0.95, boot.ci.type = "bca.simple", standardized = TRUE)

standardizedSolution_boot_ci(fit, level=0.95, type="std.all")

fitMeasures(fit)

**Key**

| FU_Smoking | Participants' responses to the self-report smoking behavior outcome at follow-up (outcome variable). |
| --- | --- |
| FU_Intentions | Participants' responses to the self-report smoking intentions outcome at follow-up (mediator variable). |
| Group1 | Dummy variable representing setting/intervention group (1=Northern Ireland Dead Cool; 0=otherwise). Base category=Northern Ireland Control group (predictor variable 1). |
| Group2 | Dummy variable representing setting/intervention group (1=Northern Ireland ASSIST non-peer supporters; 0=otherwise). Base category=Northern Ireland Control group (predictor variable 2). |
| Group3 | Dummy variable representing setting/intervention group (1=Northern Ireland ASSIST peer supporters; 0=otherwise). Base category=Northern Ireland Control group (predictor variable 3). |
| Group4 | Dummy variable representing setting/intervention group (1=Bogotá Dead Cool; 0=otherwise). Base category=Northern Ireland Control group (predictor variable 4). |
| Group5 | Dummy variable representing setting/intervention group (1=Bogotá ASSIST non-peer supporters; 0=otherwise). Base category=Northern Ireland Control group (predictor variable 5). |
| Group6 | Dummy variable representing setting/intervention group (1=Bogotá ASSIST peer supporters; 0=otherwise). Base category=Northern Ireland Control group (predictor variable 6). |
| Smoking | Participants' responses to the self-report smoking behavior outcome at baseline. |
| Intentions | Participants' responses to the self-report smoking intentions outcome at baseline. |
| Gender | Dummy variable representing participant gender (0=boy; 1=girl/prefer not to say). |
| Age1 | Dummy variable representing participant age (1=13 years; 0=otherwise). Base category=12 years or less. |
| Age2 | Dummy variable representing participant age (1=14 years or more; 0=otherwise). Base category=12 years or less. |
| Ethnicity | Dummy variable representing participant ethnicity (0=No ethnic minority; 1=ethnic minority). |
| SES1 | Dummy variable representing participant socio-economic status (1=Middle-Low/Middle [296.6<NIMDM2017≤593.2 in NI]; 0=otherwise). Base category=Informal settlement/Lowest/Low [NIMDM2017≤296.6 in NI]. |
| SES2 | Dummy variable representing participant socio-economic status (1=Middle-High/High [NIMDM2017>593.2 in NI]; 0=otherwise). Base category=Informal settlement/Lowest/Low [NIMDM2017≤296.6 in NI]. |
| ~ | Regression operator, indicates “is regressed on” (lavaan package) (24). |
| := | Defined parameter operator (lavaan package) (24). |
| A1 | Coefficient representing the effect of Northern Ireland Dead Cool compared to the Northern Ireland Control group on the mediator variable (FU_Intentions). |
| A2 | Coefficient representing the effect of Northern Ireland ASSIST non-peer supporters compared to the Northern Ireland Control group on the mediator variable (FU_Intentions). |
| A3 | Coefficient representing the effect of Northern Ireland ASSIST peer supporters compared to the Northern Ireland Control group on the mediator variable (FU_Intentions). |
| A4 | Coefficient representing the effect of Bogotá Dead Cool compared to the Northern Ireland Control group on the mediator variable (FU_Intentions). |
| A5 | Coefficient representing the effect of Bogotá ASSIST non-peer supporters compared to the Northern Ireland Control group on the mediator variable (FU_Intentions). |
| A6 | Coefficient representing the effect of Bogotá ASSIST peer supporters compared to the Northern Ireland Control group on the mediator variable (FU_Intentions). |
| B | Coefficient representing the association of the mediator variable (FU_Intentions) with the outcome variable (FU_Smoking). |
| A1B | The indirect effect of Northern Ireland Dead Cool compared to the Northern Ireland Control group on the outcome variable via the mediator (i.e., the product of the A1 and B coefficients). |
| A2B | The indirect effect of Northern Ireland ASSIST non-peer supporters compared to the Northern Ireland Control group on the outcome variable via the mediator (i.e., the product of the A2 and B coefficients). |
| A3B | The indirect effect of Northern Ireland ASSIST peer supporters compared to the Northern Ireland Control group on the outcome variable via the mediator (i.e., the product of the A3 and B coefficients). |
| A4B | The indirect effect of Bogotá Dead Cool compared to the Northern Ireland Control group on the outcome variable via the mediator (i.e., the product of the A4 and B coefficients). |
| A5B | The indirect effect of Bogotá ASSIST non-peer supporters compared to the Northern Ireland Control group on the outcome variable via the mediator (i.e., the product of the A5 and B coefficients). |
| A6B | The indirect effect of Bogotá ASSIST peer supporters compared to the Northern Ireland Control group on the outcome variable via the mediator (i.e., the product of the A6 and B coefficients). |
| AB | The total indirect effect of Northern Ireland Dead Cool, Northern Ireland ASSIST non-peer supporters, Northern Ireland ASSIST peer supporters, Bogotá Dead Cool, Bogotá ASSIST non-peer supporters, and Bogotá ASSIST peer supporters compared to the Northern Ireland Control group on the outcome variable via the mediator (i.e., the sum of A1B, A2B, A3B, A4B, A5B, and A6B). |
| CP1 | Coefficient representing the direct effect of Northern Ireland Dead Cool compared to the Northern Ireland Control group (i.e., the effect of predictor variable 1 on the outcome variable adjusted for the mediator). |
| CP2 | Coefficient representing the direct effect of Northern Ireland ASSIST non-peer supporters compared to the Northern Ireland Control group (i.e., the effect of predictor variable 2 on the outcome variable adjusted for the mediator). |
| CP3 | Coefficient representing the direct effect of Northern Ireland ASSIST peer supporters compared to the Northern Ireland Control group (i.e., the effect of predictor variable 3 on the outcome variable adjusted for the mediator). |
| CP4 | Coefficient representing the direct effect of Bogotá Dead Cool compared to the Northern Ireland Control group (i.e., the effect of predictor variable 4 on the outcome variable adjusted for the mediator). |
| CP5 | Coefficient representing the direct effect of Bogotá ASSIST non-peer supporters compared to the Northern Ireland Control group (i.e., the effect of predictor variable 5 on the outcome variable adjusted for the mediator). |
| CP6 | Coefficient representing the direct effect of Bogotá ASSIST peer supporters compared to the Northern Ireland Control group (i.e., the effect of predictor variable 6 on the outcome variable adjusted for the mediator). |
| CP | The direct effect of Northern Ireland Dead Cool, Northern Ireland ASSIST non-peer supporters, Northern Ireland ASSIST peer supporters, Bogotá Dead Cool, Bogotá ASSIST non-peer supporters, and Bogotá ASSIST peer supporters compared to the Northern Ireland Control group on the outcome variable adjusted for the mediator (i.e., the sum of CP1, CP2, CP3, CP4, CP5, and CP6). |
| C1 | Coefficient representing the total effect of Northern Ireland Dead Cool compared to the Northern Ireland Control group (i.e., the effect of predictor variable 1 on the outcome variable unadjusted for the mediator). |
| C2 | Coefficient representing the total effect of Northern Ireland ASSIST non-peer supporters compared to the Northern Ireland Control group (i.e., the effect of predictor variable 2 on the outcome variable unadjusted for the mediator). |
| C3 | Coefficient representing the total effect of Northern Ireland ASSIST peer supporters compared to the Northern Ireland Control group (i.e., the effect of predictor variable 3 on the outcome variable unadjusted for the mediator). |
| C4 | Coefficient representing the total effect of Bogotá Dead Cool compared to the Northern Ireland Control group (i.e., the effect of predictor variable 4 on the outcome variable unadjusted for the mediator). |
| C5 | Coefficient representing the total effect of Bogotá ASSIST non-peer supporters compared to the Northern Ireland Control group (i.e., the effect of predictor variable 5 on the outcome variable unadjusted for the mediator). |
| C6 | Coefficient representing the total effect of Bogotá ASSIST peer supporters compared to the Northern Ireland Control group (i.e., the effect of predictor variable 6 on the outcome variable unadjusted for the mediator). |
| C | The total effect of Northern Ireland Dead Cool, Northern Ireland ASSIST non-peer supporters, Northern Ireland ASSIST peer supporters, Bogotá Dead Cool, Bogotá ASSIST non-peer supporters, and Bogotá ASSIST peer supporters compared to the Northern Ireland Control group on the outcome variable unadjusted for the mediator (i.e., the sum of C1, C2, C3, C4, C5, and C6). |
| sem | Function to fit a structural equation model (lavaan package) (24). |
| test = "yuan.bentler" | Requests the robust versions of the model fit statistics, similar to the robust maximum likelihood (MLR) estimator. |
| std.lv | Unstandardized parameter estimates (standardizes all latent variables in the model only). |
| std.all | Standardized parameter estimates (standardizes all latent and observed variables in the model). |
| ML | Maximum-likelihood estimator. |
| fiml | Full-information maximum likelihood. |
| parameterEstimates | Function to report parameter estimates for a structural equation model (lavaan package) (24). In this example, bias-corrected bootstrap standard errors are requested (with 10,000 iterations). |
| standardizedSolution_boot_ci | Function to form bootstrap confidence intervals for the standardized solution (lavaan package) (24). |
| fitMeasures | Function to return the model fit statistics (lavaan package) (24). |

Note: The results of this model are reported at the top of Supplementary Tables S76 and S77.

## Example syntax for the 'Coordinate-wise Mediation Filter' (CMF) used to select mediators to include in the multiple mediator models.

Example 1: cmfilter <- cmf(x=Predictor1, M=Mediators1, y=Outcome1, decisionFunction = "prodcoef", maxIter = 10000, nStarts = 10000, pb = TRUE, p.value=0.1)

screeplot(cmfilter)

summary(cmfilter)

Example 2: cmfilter <- cmf(x=Predictor2, M=Mediators2, y=Outcome2, decisionFunction = "prodcoef", maxIter = 10000, nStarts = 10000, pb = TRUE, p.value=0.1)

screeplot(cmfilter)

summary(cmfilter)

Example 3: cmfilter <- cmf(x=Predictor3, M=Mediators3, y=Outcome3, decisionFunction = "prodcoef", maxIter = 10000, nStarts = 10000, pb = TRUE, p.value=0.1)

screeplot(cmfilter)

summary(cmfilter)

Example 4: cmfilter <- cmf(x=Predictor4, M=Mediators4, y=Outcome4, decisionFunction = "prodcoef", maxIter = 10000, nStarts = 10000, pb = TRUE, p.value=0.1)

screeplot(cmfilter)

summary(cmfilter)

**Key**

| cmf | Function to run the 'Coordinate-wise Mediation Filter' (cmfilter package) (38). |
| --- | --- |
| Predictor1 | Vector containing the predictor variable (Intervention). Intervention is a dummy variable indicating ASSIST versus Dead Cool. |
| Mediators1 | Matrix containing the potential mediator variables (residualized with respect to their intercepts, baseline values, Gender, Age1, Age2, Ethnicity, SES1, SES2, and Setting). |
| Outcome1 | Vector containing the outcome variable (residualized with respect to its intercept, baseline values, Gender, Age1, Age2, Ethnicity, SES1, SES2, and Setting). |
| Predictor2 | Vector containing the predictor variable (Intervention1). Intervention1 is a dummy variable indicating ASSIST non-peer supporters. |
| Mediators2 | Matrix containing the potential mediator variables (residualized with respect to their intercepts, baseline values, Gender, Age1, Age2, Ethnicity, SES1, SES2, Setting, and Intervention2). Intervention2 is a dummy variable indicating ASSIST peer supporters. |
| Outcome2 | Vector containing the outcome variable (residualized with respect to its intercept, baseline values, Gender, Age1, Age2, Ethnicity, SES1, SES2, Setting, and Intervention2). |
| Predictor3 | Vector containing the predictor variable (Intervention2). |
| Mediators3 | Matrix containing the potential mediator variables (residualized with respect to their intercepts, baseline values, Gender, Age1, Age2, Ethnicity, SES1, SES2, Setting, and Intervention1). |
| Outcome3 | Vector containing the outcome variable (residualized with respect to its intercept, baseline values, Gender, Age1, Age2, Ethnicity, SES1, SES2, Setting, and Intervention1). |
| Predictor4 | Vector containing the predictor variable (Setting). Setting is a dummy variable indicating Bogotá versus Northern Ireland. |
| Mediators4 | Matrix containing the potential mediator variables (residualized with respect to their intercepts, baseline values, Gender, Age1, Age2, Ethnicity, SES1, SES2, and Intervention). |
| Outcome4 | Vector containing the outcome variable (residualized with respect to its intercept, baseline values, Gender, Age1, Age2, Ethnicity, SES1, SES2, and Intervention). |
| prodcoef | Product of coefficients. |
| maxIter | Indicates number of iterations. |
| nStarts | Indicates number of random starts. |

Note: Prior to running the CMF algorithms, standardized residualized scores on mediators and outcome variables were created. For models with intervention as the predictor variable, mediators and outcome variables were residualized with respect to their intercept, baseline values, gender, age, ethnicity, socio-economic status, and setting. For models with setting as the predictor variable, mediators and outcome variables were residualized with respect to their intercept, baseline values, gender, age, ethnicity, socio-economic status, and intervention. In the CMFs, observed scale averages were used as approximations for mediators and outcomes treated as latent variables in SEMs. Example 1 pertains to the multiple mediator models with intervention group (ASSIST versus Dead Cool) as the predictor variable, reported in Table 7 and Supplementary Table S23. Examples 2 and 3 pertain to the multiple mediator models with predictor variables comparing ASSIST non-peer supporters and ASSIST peer supporters separately with Dead Cool, reported in Table 8 and Supplementary Table S38. Example 4 pertains to the multiple mediator models with setting (Bogotá versus Northern Ireland) as the predictor variable, reported in Table 9 and Supplementary Table S53.

## Example syntax for multiple mediator models with intervention group (ASSIST versus Dead Cool) as the predictor variable, including data from MECHANISMS schools.

model1 <- 'RiskSocial =~ a*RS1 + b*RS2 + c*RS3

FU_RiskSocial =~ a*FU_RS1 + b*FU_RS2 + c*FU_RS3

RS1 ~~ FU_RS1

RS2 ~~ FU_RS2

RS3 ~~ FU_RS3

RS1 + FU_RS1 ~ d*1

RS2 + FU_RS2 ~ 1

RS3 + FU_RS3 ~ e*1

RiskSocial ~~ 1*RiskSocial

FU_RiskSocial ~~ 1*FU_RiskSocial

RS1 ~~ f*RS1

RS2 ~~ g*RS2

RS3 ~~ h*RS3

FU_RS1 ~~ f*FU_RS1

FU_RS2 ~~ g*FU_RS2

FU_RS3 ~~ h*FU_RS3

FU_Donation ~ B1*FU_RiskSocial + B2*FU_AdvertMedia + CP*Intervention + Setting + Donation + RiskSocial + AdvertMedia + Gender + Age1 + Age2 + Ethnicity + SES1 + SES2

FU_RiskSocial ~ A1*Intervention + Setting + Donation + RiskSocial + AdvertMedia + Gender + Age1 + Age2 + Ethnicity + SES1 + SES2

FU_AdvertMedia ~ A2*Intervention + Setting + Donation + RiskSocial + AdvertMedia + Gender + Age1 + Age2 + Ethnicity + SES1 + SES2

A1B1 := A1 * B1

A2B2 := A2 * B2

AB := A1B1 + A2B2

C := CP + AB'

set.seed(1234)

fit1 <- sem(model = model1, data = data, std.lv = TRUE, estimator="ML", missing="fiml", se = "bootstrap", bootstrap = 10000, test = "yuan.bentler")

summary(fit1, fit.measures=TRUE, standardized=TRUE, rsquare=TRUE)

parameterEstimates(fit1, level=0.95, boot.ci.type = "bca.simple", standardized = TRUE)

standardizedSolution_boot_ci(fit1, level=0.95, type="std.all")

fitMeasures(fit1)

model2 <- 'Attitude =~ a*AT1 + b*AT3 + c*AT4 + d*AT5 + e*AT6 + f*AT7 + g*AT8 + h*AT9 + i*AT10 + j*AT11 + k*AT12

FU_Attitude =~ a*FU_AT1 + b*FU_AT3 + c*FU_AT4 + d*FU_AT5 + e*FU_AT6 + f*FU_AT7 + g*FU_AT8 + h*FU_AT9 + i*FU_AT10 + j*FU_AT11 + k*FU_AT12

AT1 ~~ FU_AT1

AT3 ~~ FU_AT3

AT4 ~~ FU_AT4

AT5 ~~ FU_AT5

AT6 ~~ FU_AT6

AT7 ~~ FU_AT7

AT8 ~~ FU_AT8

AT9 ~~ FU_AT9

AT10 ~~ FU_AT10

AT11 ~~ FU_AT11

AT12 ~~ FU_AT12

AT4 ~~ l*AT6

FU_AT4 ~~ l*FU_AT6

AT7 ~~ m*AT8

FU_AT7 ~~ m*FU_AT8

AT11 ~~ n*AT12

FU_AT11 ~~ n*FU_AT12

AT1 + FU_AT1 ~ o*1

AT3 + FU_AT3 ~ p*1

AT4 + FU_AT4 ~ q*1

AT5 + FU_AT5 ~ r*1

AT6 + FU_AT6 ~ s*1

AT7 + FU_AT7 ~ t*1

AT8 + FU_AT8 ~ u*1

AT9 + FU_AT9 ~ v*1

AT10 + FU_AT10 ~ w*1

AT11 + FU_AT11 ~ x*1

AT12 + FU_AT12 ~ y*1

Attitude ~~ 1*Attitude

FU_Attitude ~~ 1*FU_Attitude

AT1 ~~ z*AT1

AT3 ~~ aa*AT3

AT4 ~~ ab*AT4

AT5 ~~ ac*AT5

AT6 ~~ ad*AT6

AT7 ~~ ae*AT7

AT8 ~~ af*AT8

AT9 ~~ ag*AT9

AT10 ~~ ah*AT10

AT11 ~~ ai*AT11

AT12 ~~ aj*AT12

FU_AT1 ~~ z*FU_AT1

FU_AT3 ~~ aa*FU_AT3

FU_AT4 ~~ ab*FU_AT4

FU_AT5 ~~ ac*FU_AT5

FU_AT6 ~~ ad*FU_AT6

FU_AT7 ~~ ae*FU_AT7

FU_AT8 ~~ af*FU_AT8

FU_AT9 ~~ ag*FU_AT9

FU_AT10 ~~ ah*FU_AT10

FU_AT11 ~~ ai*FU_AT11

FU_AT12 ~~ aj*FU_AT12

ExptInj =~ ak*P2S2 + al*P2S3 + am*P2S4 + an*P2S5 + ao*P2S6 + ap*P2S7 + aq*P2S8 + ar*P2S9

FU_ExptInj =~ ak*FU_P2S2 + al*FU_P2S3 + am*FU_P2S4 + an*FU_P2S5 + ao*FU_P2S6 + ap*FU_P2S7 + aq*FU_P2S8 + ar*FU_P2S9

P2S2 ~~ FU_P2S2

P2S3 ~~ FU_P2S3

P2S4 ~~ FU_P2S4

P2S5 ~~ FU_P2S5

P2S6 ~~ FU_P2S6

P2S7 ~~ FU_P2S7

P2S8 ~~ FU_P2S8

P2S9 ~~ FU_P2S9

P2S2 ~~ as*P2S3

FU_P2S2 ~~as*FU_P2S3

P2S2 ~~ at*P2S4

FU_P2S2 ~~ at*FU_P2S4

P2S7 ~~ au*P2S8

FU_P2S7 ~~ au*FU_P2S8

P2S2 + FU_P2S2 ~ av*1

P2S3 + FU_P2S3 ~ aw*1

P2S4 + FU_P2S4 ~ ax*1

P2S5 + FU_P2S5 ~ ay*1

P2S6 + FU_P2S6 ~ az*1

P2S7 + FU_P2S7 ~ ba*1

P2S8 + FU_P2S8 ~ bb*1

P2S9 + FU_P2S9 ~ bc*1

ExptInj ~~ 1*ExptInj

FU_ExptInj ~~ 1*FU_ExptInj

P2S2 ~~ bd*P2S2

P2S3 ~~ be*P2S3

P2S4 ~~ bf*P2S4

P2S5 ~~ bg*P2S5

P2S6 ~~ bh*P2S6

P2S7 ~~ bi*P2S7

P2S8 ~~ bj*P2S8

P2S9 ~~ bk*P2S9

FU_P2S2 ~~ bd*FU_P2S2

FU_P2S3 ~~ be*FU_P2S3

FU_P2S4 ~~ bf*FU_P2S4

FU_P2S5 ~~ bg*FU_P2S5

FU_P2S6 ~~ bh*FU_P2S6

FU_P2S7 ~~ bi*FU_P2S7

FU_P2S8 ~~ bj*FU_P2S8

FU_P2S9 ~~ bk*FU_P2S9

FU_Susceptibility ~ B1*FU_Attitude + B2*FU_ExptInj + B3*FU_PBCavoid + CP*Intervention + Setting + Susceptibility + Attitude + ExptInj + PBCavoid + Gender + Age1 + Age2 + Ethnicity + SES1 + SES2

FU_Attitude ~ A1*Intervention + Setting + Susceptibility + Attitude + ExptInj + PBCavoid + Gender + Age1 + Age2 + Ethnicity + SES1 + SES2

FU_ExptInj ~ A2*Intervention + Setting + Susceptibility + Attitude + ExptInj + PBCavoid + Gender + Age1 + Age2 + Ethnicity + SES1 + SES2

FU_PBCavoid ~ A3*Intervention + Setting + Susceptibility + Attitude + ExptInj + PBCavoid + Gender + Age1 + Age2 + Ethnicity + SES1 + SES2

A1B1 := A1 * B1

A2B2 := A2 * B2

A3B3 := A3 * B3

AB := A1B1 + A2B2 + A3B3

C := CP + AB'

set.seed(1234)

fit2 <- sem(model = model2, data = data, std.lv = TRUE, ordered=c("FU_Susceptibility"), estimator="DWLS", se = "bootstrap", bootstrap = 10000, test = "scaled.shifted")

summary(fit2, fit.measures=TRUE, standardized=TRUE, rsquare=TRUE)

parameterEstimates(fit2, level=0.95, boot.ci.type = "bca.simple", standardized = TRUE)

standardizedSolution_boot_ci(fit2, level=0.95, type="std.all")

fitMeasures(fit2)

**Key**

| FU_Donation | Participants' responses to the experimental outcome (Donation to ASSIST/Dead Cool) at follow-up (outcome variable in model1). |
| --- | --- |
| FU_RiskSocial | Latent variable representing perceived social risks at follow-up (derived from individual items FU_RS1 to FU_RS3; mediator variable 1 in model1). |
| FU_RS1 to FU_RS3 | Participants' responses to the three items measuring perceived social risks at follow-up. |
| FU_AdvertMedia | Participants' responses to the exposure to advertising in the media outcome at follow-up (mediator variable 2 in model1). |
| Intervention | Dummy variable representing intervention group (0=Dead Cool, 1=ASSIST; predictor variable). |
| Donation | Participants' responses to the experimental outcome (Donation to ASSIST/Dead Cool) at baseline. |
| RiskSocial | Latent variable representing perceived social risks at baseline (derived from individual items RS1-RS3). |
| RS1 to RS3 | Participants' responses to the three items measuring perceived social risks at baseline. |
| AdvertMedia | Participants' responses to the exposure to advertising in the media outcome at baseline. |
| Setting | Dummy variable representing setting (0=Northern Ireland, 1=Bogotá). |
| Gender | Dummy variable representing participant gender (0=boy; 1=girl/prefer not to say). |
| Age1 | Dummy variable representing participant age (1=13 years; 0=otherwise). Base category=12 years or less. |
| Age2 | Dummy variable representing participant age (1=14 years or more; 0=otherwise). Base category=12 years or less. |
| Ethnicity | Dummy variable representing participant ethnicity (0=No ethnic minority; 1=ethnic minority). |
| SES1 | Dummy variable representing participant socio-economic status (1=Middle-Low/Middle [296.6<NIMDM2017≤593.2 in NI]; 0=otherwise). Base category=Informal settlement/Lowest/Low [NIMDM2017≤296.6 in NI]. |
| SES2 | Dummy variable representing participant socio-economic status (1=Middle-High/High [NIMDM2017>593.2 in NI]; 0=otherwise). Base category=Informal settlement/Lowest/Low [NIMDM2017≤296.6 in NI]. |
| FU_Susceptibility | Participants' responses to the smoking susceptibility outcome at follow-up (binary outcome variable in model2). |
| FU_Attitude | Latent variable representing attitudes towards smoking at follow-up (derived from individual items FU_AT1 to FU_AT12, excluding FU_AT2; mediator variable 1 in model2). |
| FU_AT1 to FU_AT12, excluding FU_AT2 | Participants' responses to the 11 items measuring attitudes towards smoking at follow-up. |
| FU_ExptInj | Latent variable representing experimentally measured injunctive smoking/vaping norms at follow-up (derived from individual items FU_P2S2 to FU_P2S9; mediator variable 2 in model2). |
| FU_P2S2 to FU_P2S9 | Participants' responses to the 8 items measuring experimentally measured injunctive smoking/vaping norms at follow-up. |
| FU_PBCavoid | Participants' responses to the perceived behavioral control (to avoid smoking) outcome at follow-up (mediator variable 3 in model2). |
| Susceptibility | Participants' responses to the smoking susceptibility outcome at baseline. |
| Attitude | Latent variable representing attitudes towards smoking at baseline (derived from individual items AT1 to AT12, excluding AT2). |
| AT1 to AT12, excluding AT2 | Participants' responses to the 11 items measuring attitudes towards smoking at baseline. |
| ExptInj | Latent variable representing experimentally measured injunctive smoking/vaping norms at baseline (derived from individual items P2S2 to P2S9). |
| P2S2 to P2S9 | Participants' responses to the 8 items measuring experimentally measured injunctive smoking/vaping norms at baseline. |
| PBCavoid | Participants' responses to the perceived behavioral control (to avoid smoking) outcome at baseline. |
| =~ | Latent variable operator, indicates “is measured by” (lavaan package) (24). |
| ~~ | Covariance operator, indicates “is correlated with” (lavaan package) (24). |
| ~1 | Intercept operator, defines the intercept (lavaan package) (24). |
| * | Used to define parameter labels and specify constraints. E.g., “a*RS1” and “a*FU_RS1” indicates “constrain the estimated factor loadings for RS1 and FU_RS1 to be equal”. Also used to indicate multiplication. |
| ~ | Regression operator, indicates “is regressed on” (lavaan package) (24). |
| := | Defined parameter operator (lavaan package) (24). |
| A1 | Coefficient representing the predictor variable (Intervention) effect on mediator variable 1 (FU_RiskSocial in model1, FU_Attitude in model2). |
| A2 | Coefficient representing the predictor variable (Intervention) effect on mediator variable 2 (FU_AdvertMedia in model1, FU_ExptInj in model2). |
| A3 | Coefficient representing the predictor variable (Intervention) effect on mediator variable 3 (FU_PBCavoid in model2). |
| B1 | Coefficient representing the association of mediator variable 1 (FU_RiskSocial in model1, FU_Attitude in model2) with the outcome variable (FU_Donation in model1, FU_Susceptibility in model2). |
| B2 | Coefficient representing the association of mediator variable 2 (FU_AdvertMedia in model1, FU_ExptInj in model2) with the outcome variable (FU_Donation in model1, FU_Susceptibility in model2). |
| B3 | Coefficient representing the association of mediator variable 3 (FU_PBCavoid in model2) with the outcome variable (FU_Susceptibility in model2). |
| A1B1 | Coefficient representing the indirect effect of the predictor variable on the outcome variable via mediator variable 1 (i.e., the product of the A1 and B1 coefficients). |
| A2B2 | Coefficient representing the indirect effect of the predictor variable on the outcome variable via mediator variable 2 (i.e., the product of the A2 and B2 coefficients). |
| A3B3 | Coefficient representing the indirect effect of the predictor variable on the outcome variable via mediator variable 3 (i.e., the product of the A3 and B3 coefficients). |
| AB | Coefficient representing the indirect effect of the predictor variable on the outcome variable via all mediators included in the model (i.e., the sum of the indirect effects via each individual mediator). |
| CP | Coefficient representing the direct effect (i.e., the effect of the predictor variable on the outcome variable adjusted for the mediators). |
| C | Coefficient representing the total effect (i.e., the effect of the predictor variable on the outcome variable unadjusted for the mediators). |
| sem | Function to fit a structural equation model (lavaan package) (24). |
| test = "yuan.bentler" | Requests the robust versions of the model fit statistics, similar to the robust maximum likelihood (MLR) estimator. |
| test = "scaled.shifted" | Requests the robust versions of the model fit statistics, similar to the weighted least square mean and variance adjusted (WLSMV) estimator. |
| std.lv | Unstandardized parameter estimates (standardizes all latent variables in the model only). |
| std.all | Standardized parameter estimates (standardizes all latent and observed variables in the model). |
| ML | Maximum-likelihood estimator. |
| DWLS | Diagonally weighted least squares estimator. |
| fiml | Full-information maximum likelihood. |
| ordered | Specifying “ordered = c("FU_Susceptibility")” in the “sem” call for “model2” indicates that “FU_Susceptibility” should be treated as a categorical outcome variable. |
| parameterEstimates | Function to report parameter estimates for a structural equation model (lavaan package) (24). In this example, bias-corrected bootstrap standard errors are requested (with 10,000 iterations). |
| standardizedSolution_boot_ci | Function to form bootstrap confidence intervals for the standardized solution (lavaan package) (24). |
| fitMeasures | Function to return the model fit statistics (lavaan package) (24). |

Note: The results of model1 and model2 are reported in Table 7 and Supplementary Table S23.

## Example syntax for multiple mediator models with predictor variables comparing ASSIST non-peer supporters and ASSIST peer supporters separately with Dead Cool, including data from MECHANISMS schools.

model <- 'Attitude =~ a*attitude1 + b*attitude3 + c*attitude4 + d*attitude5 + e*attitude6 + f*attitude7 + g*attitude8 + h*attitude9 + i*attitude10 + j*attitude11 + k*attitude12

FU_Attitude =~ a*fu_attitude1 + b*fu_attitude3 + c*fu_attitude4 + d*fu_attitude5 + e*fu_attitude6 + f*fu_attitude7 + g*fu_attitude8 + h*fu_attitude9 + i*fu_attitude10 + j*fu_attitude11 + k*fu_attitude12

attitude1 ~~ fu_attitude1

attitude3 ~~ fu_attitude3

attitude4 ~~ fu_attitude4

attitude5 ~~ fu_attitude5

attitude6 ~~ fu_attitude6

attitude7 ~~ fu_attitude7

attitude8 ~~ fu_attitude8

attitude9 ~~ fu_attitude9

attitude10 ~~ fu_attitude10

attitude11 ~~ fu_attitude11

attitude12 ~~ fu_attitude12

attitude4 ~~ l*attitude6

fu_attitude4 ~~ l*fu_attitude6

attitude7 ~~ m*attitude8

fu_attitude7 ~~ m*fu_attitude8

attitude11 ~~ n*attitude12

fu_attitude11 ~~ n*fu_attitude12

attitude1 + fu_attitude1 ~ o*1

attitude3 + fu_attitude3 ~ p*1

attitude4 + fu_attitude4 ~ q*1

attitude5 + fu_attitude5 ~ r*1

attitude6 + fu_attitude6 ~ s*1

attitude7 + fu_attitude7 ~ t*1

attitude8 + fu_attitude8 ~ u*1

attitude9 + fu_attitude9 ~ v*1

attitude10 + fu_attitude10 ~ w*1

attitude11 + fu_attitude11 ~ x*1

attitude12 + fu_attitude12 ~ y*1

Attitude ~~ 1*Attitude

FU_Attitude ~~ 1*FU_Attitude

attitude1 ~~ z*attitude1

attitude3 ~~ aa*attitude3

attitude4 ~~ ab*attitude4

attitude5 ~~ ac*attitude5

attitude6 ~~ ad*attitude6

attitude7 ~~ ae*attitude7

attitude8 ~~ af*attitude8

attitude9 ~~ ag*attitude9

attitude10 ~~ ah*attitude10

attitude11 ~~ ai*attitude11

attitude12 ~~ aj*attitude12

fu_attitude1 ~~ z*fu_attitude1

fu_attitude3 ~~ aa*fu_attitude3

fu_attitude4 ~~ ab*fu_attitude4

fu_attitude5 ~~ ac*fu_attitude5

fu_attitude6 ~~ ad*fu_attitude6

fu_attitude7 ~~ ae*fu_attitude7

fu_attitude8 ~~ af*fu_attitude8

fu_attitude9 ~~ ag*fu_attitude9

fu_attitude10 ~~ ah*fu_attitude10

fu_attitude11 ~~ ai*fu_attitude11

fu_attitude12 ~~ aj*fu_attitude12

SurveyDesc2 =~ ak*descriptivecrem2_1 + al*descriptivecrem2_2 + am*descriptivecrem2_3

FU_SurveyDesc2 =~ ak*fu_descriptivecrem2_1 + al*fu_descriptivecrem2_2 + am*fu_descriptivecrem2_3

descriptivecrem2_1 ~~ fu_descriptivecrem2_1

descriptivecrem2_2 ~~ fu_descriptivecrem2_2

descriptivecrem2_3 ~~ fu_descriptivecrem2_3

descriptivecrem2_1 + fu_descriptivecrem2_1 ~ an*1

descriptivecrem2_2 + fu_descriptivecrem2_2 ~ ao*1

descriptivecrem2_3 + fu_descriptivecrem2_3 ~ ap*1

SurveyDesc2 ~~ 1*SurveyDesc2

FU_SurveyDesc2 ~~ 1*FU_SurveyDesc2

descriptivecrem2_1 ~~ aq*descriptivecrem2_1

descriptivecrem2_2 ~~ ar*descriptivecrem2_2

descriptivecrem2_3 ~~ as*descriptivecrem2_3

fu_descriptivecrem2_1 ~~ aq*fu_descriptivecrem2_1

fu_descriptivecrem2_2 ~~ ar*fu_descriptivecrem2_2

fu_descriptivecrem2_3 ~~ as*fu_descriptivecrem2_3

RiskSocial =~ at*risksocial1 + au*risksocial2 + av*risksocial3

FU_RiskSocial =~ at*fu_risksocial1 + au*fu_risksocial2 + av*fu_risksocial3

risksocial1 ~~ fu_risksocial1

risksocial2 ~~ fu_risksocial2

risksocial3 ~~ fu_risksocial3

risksocial1 + fu_risksocial1 ~ aw*1

risksocial2 + fu_risksocial2 ~ 1

risksocial3 + fu_risksocial3 ~ ax*1

RiskSocial ~~ 1*RiskSocial

FU_RiskSocial ~~ 1*FU_RiskSocial

risksocial1 ~~ ay*risksocial1

risksocial2 ~~ az*risksocial2

risksocial3 ~~ ba*risksocial3

fu_risksocial1 ~~ ay*fu_risksocial1

fu_risksocial2 ~~ az*fu_risksocial2

fu_risksocial3 ~~ ba*fu_risksocial3

FU_Donation ~ B1*FU_Smoking + B2*FU_Attitude + B3*FU_SurveyDesc2 + B4*FU_RiskSocial + B5*FU_AdvertMedia + CP1*Intervention1 + CP2*Intervention2 + Setting + Donation + Smoking + Attitude + SurveyDesc2 + RiskSocial + AdvertMedia + Gender + Age1 + Age2 + Ethnicity + SES1 + SES2

FU_Smoking ~ A1*Intervention1 + A2*Intervention2 + Setting + Donation + Smoking + Attitude + SurveyDesc2 + RiskSocial + AdvertMedia + Gender + Age1 + Age2 + Ethnicity + SES1 + SES2

FU_Attitude ~ A3*Intervention1 + A4*Intervention2 + Setting + Donation + Smoking + Attitude + SurveyDesc2 + RiskSocial + AdvertMedia + Gender + Age1 + Age2 + Ethnicity + SES1 + SES2

FU_SurveyDesc2 ~ A5*Intervention1 + A6*Intervention2 + Setting + Donation + Smoking + Attitude + SurveyDesc2 + RiskSocial + AdvertMedia + Gender + Age1 + Age2 + Ethnicity + SES1 + SES2

FU_RiskSocial ~ A7*Intervention1 + A8*Intervention2 + Setting + Donation + Smoking + Attitude + SurveyDesc2 + RiskSocial + AdvertMedia + Gender + Age1 + Age2 + Ethnicity + SES1 + SES2

FU_AdvertMedia ~ A9*Intervention1 + A10*Intervention2 + Setting + Donation + Smoking + Attitude + SurveyDesc2 + RiskSocial + AdvertMedia + Gender + Age1 + Age2 + Ethnicity + SES1 + SES2

A1B1 := A1 * B1

A2B1 := A2 * B1

A3B2 := A3 * B2

A4B2 := A4 * B2

A5B3 := A5 * B3

A6B3 := A6 * B3

A7B4 := A7 * B4

A8B4 := A8 * B4

A9B5 := A9 * B5

A10B5 := A10 * B5

A1B := A1B1 + A3B2 + A5B3 + A7B4 + A9B5

A2B := A2B1 + A4B2 + A6B3 + A8B4 + A10B5

AB1 := A1B1 + A2B1

AB2 := A3B2 + A4B2

AB3 := A5B3 + A6B3

AB4 := A7B4 + A8B4

AB5 := A9B5 + A10B5

C1 := CP1 + A1B

C2 := CP2 + A2B

AB := A1B + A2B

CP := CP1 + CP2

C := C1 + C2'

set.seed(1234)

fit <- sem(model = model, data = data, std.lv = TRUE, estimator="ML", missing="fiml", se = "bootstrap", bootstrap = 10000, test = "yuan.bentler")

summary(fit, fit.measures=TRUE, standardized=TRUE, rsquare=TRUE)

parameterEstimates(fit, level=0.95, boot.ci.type = "bca.simple", standardized = TRUE)

standardizedSolution_boot_ci(fit, level=0.95, type="std.all")

fitMeasures(fit)

**Key**

| FU_Donation | Participants' responses to the experimental outcome (Donation to ASSIST/Dead Cool) at follow-up (outcome variable). |
| --- | --- |
| FU_Smoking | Participants' responses to the self-report smoking behavior outcome at follow-up (mediator variable 1). |
| FU_Attitude | Latent variable representing attitudes towards smoking at follow-up (derived from individual items FU_AT1 to FU_AT12, excluding FU_AT2; mediator variable 2). |
| FU_AT1 to FU_AT12, excluding FU_AT2 | Participants' responses to the 11 items measuring attitudes towards smoking at follow-up. |
| FU_SurveyDesc2 | Latent variable representing self-report descriptive smoking norms scale 2 at follow-up (derived from individual items FU_DN2.1 to FU_DN2.3; mediator variable 3). |
| FU_DN2.1 to FU_DN2.3 | Participants' responses to the 3 items measuring self-report descriptive smoking norms scale 2 at follow-up. |
| FU_RiskSocial | Latent variable representing perceived social risks at follow-up (derived from individual items FU_RS1 to FU_RS3; mediator variable 4). |
| FU_RS1 to FU_RS3 | Participants' responses to the three items measuring perceived social risks at follow-up. |
| FU_AdvertMedia | Participants' responses to the exposure to advertising in the media outcome at follow-up (mediator variable 5). |
| Intervention1 | Dummy variable representing intervention group (1=ASSIST non-peer supporters; 0=otherwise). Base category=Dead Cool (predictor variable 1). |
| Intervention2 | Dummy variable representing intervention group (1=ASSIST peer supporters; 0=otherwise). Base category=Dead Cool (predictor variable 2). |
| Donation | Participants' responses to the experimental outcome (Donation to ASSIST/Dead Cool) at baseline. |
| Smoking | Participants' responses to the self-report smoking behavior outcome at baseline. |
| Attitude | Latent variable representing attitudes towards smoking at baseline (derived from individual items AT1 to AT12, excluding AT2). |
| AT1 to AT12, excluding AT2 | Participants' responses to the 11 items measuring attitudes towards smoking at baseline. |
| SurveyDesc2 | Latent variable representing self-report descriptive smoking norms scale 2 at baseline (derived from individual items DN2.1 to DN2.3). |
| DN2.1 to DN2.3 | Participants' responses to the 3 items measuring self-report descriptive smoking norms scale 2 at baseline. |
| RiskSocial | Latent variable representing perceived social risks at baseline (derived from individual items RS1 to RS3). |
| RS1 to RS3 | Participants' responses to the three items measuring perceived social risks at baseline. |
| AdvertMedia | Participants' responses to the exposure to advertising in the media outcome at baseline. |
| Setting | Dummy variable representing setting (0=Northern Ireland, 1=Bogotá). |
| Gender | Dummy variable representing participant gender (0=boy; 1=girl/prefer not to say). |
| Age1 | Dummy variable representing participant age (1=13 years; 0=otherwise). Base category=12 years or less. |
| Age2 | Dummy variable representing participant age (1=14 years or more; 0=otherwise). Base category=12 years or less. |
| Ethnicity | Dummy variable representing participant ethnicity (0=No ethnic minority; 1=ethnic minority). |
| SES1 | Dummy variable representing participant socio-economic status (1=Middle-Low/Middle [296.6<NIMDM2017≤593.2 in NI]; 0=otherwise). Base category=Informal settlement/Lowest/Low [NIMDM2017≤296.6 in NI]. |
| SES2 | Dummy variable representing participant socio-economic status (1=Middle-High/High [NIMDM2017>593.2 in NI]; 0=otherwise). Base category=Informal settlement/Lowest/Low [NIMDM2017≤296.6 in NI]. |
| =~ | Latent variable operator, indicates “is measured by” (lavaan package) (24). |
| ~~ | Covariance operator, indicates “is correlated with” (lavaan package) (24). |
| ~1 | Intercept operator, defines the intercept (lavaan package) (24). |
| * | Used to define parameter labels and specify constraints. E.g., “a*AT1” and “a*FU_AT1” indicates “constrain the estimated factor loadings for AT1 and FU_AT1 to be equal”. Also used to indicate multiplication. |
| ~ | Regression operator, indicates “is regressed on” (lavaan package) (24). |
| := | Defined parameter operator (lavaan package) (24). |
| A1 | Coefficient representing the effect of ASSIST non-peer supporters compared to Dead Cool on mediator variable 1 (FU_Smoking). |
| A2 | Coefficient representing the effect of ASSIST peer supporters compared to Dead Cool on mediator variable 1 (FU_Smoking). |
| A3 | Coefficient representing the effect of ASSIST non-peer supporters compared to Dead Cool on mediator variable 2 (FU_Attitude). |
| A4 | Coefficient representing the effect of ASSIST peer supporters compared to Dead Cool on mediator variable 2 (FU_Attitude). |
| A5 | Coefficient representing the effect of ASSIST non-peer supporters compared to Dead Cool on mediator variable 3 (FU_SurveyDesc2). |
| A6 | Coefficient representing the effect of ASSIST peer supporters compared to Dead Cool on mediator variable 3 (FU_SurveyDesc2). |
| A7 | Coefficient representing the effect of ASSIST non-peer supporters compared to Dead Cool on mediator variable 4 (FU_RiskSocial). |
| A8 | Coefficient representing the effect of ASSIST peer supporters compared to Dead Cool on mediator variable 4 (FU_RiskSocial). |
| A9 | Coefficient representing the effect of ASSIST non-peer supporters compared to Dead Cool on mediator variable 5 (FU_AdvertMedia). |
| A10 | Coefficient representing the effect of ASSIST peer supporters compared to Dead Cool on mediator variable 5 (FU_AdvertMedia). |
| B1 | Coefficient representing the association of mediator variable 1 (FU_Smoking) with the outcome variable (FU_Donation). |
| B2 | Coefficient representing the association of mediator variable 2 (FU_Attitude) with the outcome variable (FU_Donation). |
| B3 | Coefficient representing the association of mediator variable 3 (FU_SurveyDesc2) with the outcome variable (FU_Donation). |
| B4 | Coefficient representing the association of mediator variable 4 (FU_RiskSocial) with the outcome variable (FU_Donation). |
| B5 | Coefficient representing the association of mediator variable 5 (FU_AdvertMedia) with the outcome variable (FU_Donation). |
| A1B1 | The indirect effect of ASSIST non-peer supporters compared to Dead Cool on the outcome variable via mediator 1 (i.e., the product of the A1 and B1 coefficients). |
| A2B1 | The indirect effect of ASSIST peer supporters compared to Dead Cool on the outcome variable via mediator 1 (i.e., the product of the A2 and B1 coefficients). |
| A3B2 | The indirect effect of ASSIST non-peer supporters compared to Dead Cool on the outcome variable via mediator 2 (i.e., the product of the A3 and B2 coefficients). |
| A4B2 | The indirect effect of ASSIST peer supporters compared to Dead Cool on the outcome variable via mediator 2 (i.e., the product of the A4 and B2 coefficients). |
| A5B3 | The indirect effect of ASSIST non-peer supporters compared to Dead Cool on the outcome variable via mediator 3 (i.e., the product of the A5 and B3 coefficients). |
| A6B3 | The indirect effect of ASSIST peer supporters compared to Dead Cool on the outcome variable via mediator 3 (i.e., the product of the A6 and B3 coefficients). |
| A7B4 | The indirect effect of ASSIST non-peer supporters compared to Dead Cool on the outcome variable via mediator 4 (i.e., the product of the A7 and B4 coefficients). |
| A8B4 | The indirect effect of ASSIST peer supporters compared to Dead Cool on the outcome variable via mediator 4 (i.e., the product of the A8 and B4 coefficients). |
| A9B5 | The indirect effect of ASSIST non-peer supporters compared to Dead Cool on the outcome variable via mediator 5 (i.e., the product of the A9 and B5 coefficients). |
| A10B5 | The indirect effect of ASSIST peer supporters compared to Dead Cool on the outcome variable via mediator 5 (i.e., the product of the A10 and B5 coefficients). |
| A1B | The indirect effect of ASSIST non-peer supporters compared to Dead Cool on the outcome variable via all mediators included in the model (i.e., the sum of A1B1, A3B2, A5B3, A7B4, and A9B5). |
| A2B | The indirect effect of ASSIST peer supporters compared to Dead Cool on the outcome variable via all mediators included in the model (i.e., the sum of A2B1, A4B2, A6B3, A8B4, and A10B5). |
| AB | The total indirect effect of ASSIST non-peer supporters and ASSIST peer supporters compared to Dead Cool on the outcome variable via all mediators included in the model (i.e., the sum of A1B and A2B). |
| CP1 | Coefficient representing the direct effect of ASSIST non-peer supporters compared to Dead Cool (i.e., the effect of predictor variable 1 on the outcome variable adjusted for the mediators). |
| CP2 | Coefficient representing the direct effect of ASSIST peer supporters compared to Dead Cool (i.e., the effect of the predictor variable 2 on the outcome variable adjusted for the mediators). |
| CP | The direct effect of ASSIST non-peer supporters and ASSIST peer supporters compared to Dead Cool on the outcome variable adjusted for the mediators (i.e., the sum of CP1 and CP2). |
| C1 | Coefficient representing the total effect of ASSIST non-peer supporters compared to Dead Cool (i.e., the effect of predictor variable 1 on the outcome variable unadjusted for the mediators). |
| C2 | Coefficient representing the total effect of ASSIST peer supporters compared to Dead Cool (i.e., the effect of the predictor variable 2 on the outcome variable unadjusted for the mediators). |
| C | The total effect of ASSIST non-peer supporters and ASSIST peer supporters compared to Dead Cool on the outcome variable unadjusted for the mediators (i.e., the sum of C1 and C2). |
| sem | Function to fit a structural equation model (lavaan package) (24). |
| test = "yuan.bentler" | Requests the robust versions of the model fit statistics, similar to the robust maximum likelihood (MLR) estimator. |
| std.lv | Unstandardized parameter estimates (standardizes all latent variables in the model only). |
| std.all | Standardized parameter estimates (standardizes all latent and observed variables in the model). |
| ML | Maximum-likelihood estimator. |
| fiml | Full-information maximum likelihood. |
| parameterEstimates | Function to report parameter estimates for a structural equation model (lavaan package) (24). In this example, bias-corrected bootstrap standard errors are requested (with 10,000 iterations). |
| standardizedSolution_boot_ci | Function to form bootstrap confidence intervals for the standardized solution (lavaan package) (24). |
| fitMeasures | Function to return the model fit statistics (lavaan package) (24). |

Note: The results of this model are reported in the first row of Table 8 and Supplementary Table S38.

## Example syntax for multiple mediator models including predictor variables comparing MECHANISMS setting/intervention groups with the Northern Ireland control group (sensitivity analyses).

model <- 'FU_Smoking ~ B1*FU_Intentions + B2*FU_AdvertShops + CP1*Group1 + CP2*Group2 + CP3*Group3 + CP4*Group4 + Smoking + Intentions + AdvertShops + Gender + Age1 + Age2 + Ethnicity + SES1 + SES2

FU_Intentions ~ A1*Group1 + A2*Group2 + A3*Group3 + A4*Group4 + Smoking + Intentions + AdvertShops + Gender + Age1 + Age2 + Ethnicity + SES1 + SES2

FU_AdvertShops ~ A5*Group1 + A6*Group2 + A7*Group3 + A8*Group4 + Smoking + Intentions + AdvertShops + Gender + Age1 + Age2 + Ethnicity + SES1 + SES2

A1B1 := A1 * B1

A2B1 := A2 * B1

A3B1 := A3 * B1

A4B1 := A4 * B1

A5B2 := A5 * B2

A6B2 := A6 * B2

A7B2 := A7 * B2

A8B2 := A8 * B2

C1 := CP1 + A1B1 + A5B2

C2 := CP2 + A2B1 + A6B2

C3 := CP3 + A3B1 + A7B2

C4 := CP4 + A4B1 + A8B2

AB1 := A1B1 + A2B1 + A3B1 + A4B1

AB2 := A5B2 + A6B2 + A7B2 + A8B2

AB := AB1 + AB2

CP := CP1 + CP2 + CP3 + CP4

C := C1 + C2 + C3 + C4'

set.seed(1234)

fit <- sem(model = model, data = data, std.lv = TRUE, estimator="ML", missing="fiml", se = "bootstrap", bootstrap = 10000, test = "yuan.bentler")

summary(fit, fit.measures=TRUE, standardized=TRUE, rsquare=TRUE)

parameterEstimates(fit, level=0.95, boot.ci.type = "bca.simple", standardized = TRUE)

standardizedSolution_boot_ci(fit, level=0.95, type="std.all")

fitMeasures(fit)

**Key**

| FU_Smoking | Participants' responses to the self-report smoking behavior outcome at follow-up (outcome variable). |
| --- | --- |
| FU_Intentions | Participants' responses to the self-report smoking intentions outcome at follow-up (mediator variable 1). |
| FU_AdvertShops | Participants' responses to the self-report exposure to advertising in shops outcome at follow-up (mediator variable 2). |
| Group1 | Dummy variable representing setting/intervention group (1=Northern Ireland Dead Cool; 0=otherwise). Base category=Northern Ireland Control group (predictor variable 1). |
| Group2 | Dummy variable representing setting/intervention group (1=Northern Ireland ASSIST; 0=otherwise). Base category=Northern Ireland Control group (predictor variable 2). |
| Group3 | Dummy variable representing setting/intervention group (1=Bogotá Dead Cool; 0=otherwise). Base category=Northern Ireland Control group (predictor variable 3). |
| Group4 | Dummy variable representing setting/intervention group (1=Bogotá ASSIST; 0=otherwise). Base category=Northern Ireland Control group (predictor variable 4). |
| Smoking | Participants' responses to the self-report smoking behavior outcome at baseline. |
| Intentions | Participants' responses to the self-report smoking intentions outcome at baseline. |
| AdvertShops | Participants' responses to the self-report exposure to advertising in shops outcome at baseline. |
| Gender | Dummy variable representing participant gender (0=boy; 1=girl/prefer not to say). |
| Age1 | Dummy variable representing participant age (1=13 years; 0=otherwise). Base category=12 years or less. |
| Age2 | Dummy variable representing participant age (1=14 years or more; 0=otherwise). Base category=12 years or less. |
| Ethnicity | Dummy variable representing participant ethnicity (0=No ethnic minority; 1=ethnic minority). |
| SES1 | Dummy variable representing participant socio-economic status (1=Middle-Low/Middle [296.6<NIMDM2017≤593.2 in NI]; 0=otherwise). Base category=Informal settlement/Lowest/Low [NIMDM2017≤296.6 in NI]. |
| SES2 | Dummy variable representing participant socio-economic status (1=Middle-High/High [NIMDM2017>593.2 in NI]; 0=otherwise). Base category=Informal settlement/Lowest/Low [NIMDM2017≤296.6 in NI]. |
| ~ | Regression operator, indicates “is regressed on” (lavaan package) (24). |
| := | Defined parameter operator (lavaan package) (24). |
| A1 | Coefficient representing the effect of Northern Ireland Dead Cool compared to the Northern Ireland Control group on mediator variable 1 (FU_Intentions). |
| A2 | Coefficient representing the effect of Northern Ireland ASSIST compared to the Northern Ireland Control group on mediator variable 1 (FU_Intentions). |
| A3 | Coefficient representing the effect of Bogotá Dead Cool compared to the Northern Ireland Control group on mediator variable 1 (FU_Intentions). |
| A4 | Coefficient representing the effect of Bogotá ASSIST compared to the Northern Ireland Control group on mediator variable 1 (FU_Intentions). |
| A5 | Coefficient representing the effect of Northern Ireland Dead Cool compared to the Northern Ireland Control group on mediator variable 2 (FU_AdvertShops). |
| A6 | Coefficient representing the effect of Northern Ireland ASSIST compared to the Northern Ireland Control group on mediator variable 2 (FU_AdvertShops). |
| A7 | Coefficient representing the effect of Bogotá Dead Cool compared to the Northern Ireland Control group on mediator variable 2 (FU_AdvertShops). |
| A8 | Coefficient representing the effect of Bogotá ASSIST compared to the Northern Ireland Control group on mediator variable 2 (FU_AdvertShops). |
| B1 | Coefficient representing the association of mediator variable 1 (FU_Intentions) with the outcome variable (FU_Smoking). |
| B2 | Coefficient representing the association of mediator variable 2 (FU_AdvertShops) with the outcome variable (FU_Smoking). |
| A1B1 | The indirect effect of Northern Ireland Dead Cool compared to the Northern Ireland Control group on the outcome variable via mediator variable 1 (i.e., the product of the A1 and B1 coefficients). |
| A2B1 | The indirect effect of Northern Ireland ASSIST compared to the Northern Ireland Control group on the outcome variable via mediator variable 1 (i.e., the product of the A2 and B1 coefficients). |
| A3B1 | The indirect effect of Bogotá Dead Cool compared to the Northern Ireland Control group on the outcome variable via mediator variable 1 (i.e., the product of the A3 and B1 coefficients). |
| A4B1 | The indirect effect of Bogotá ASSIST compared to the Northern Ireland Control group on the outcome variable via mediator variable 1 (i.e., the product of the A4 and B1 coefficients). |
| A5B2 | The indirect effect of Northern Ireland Dead Cool compared to the Northern Ireland Control group on the outcome variable via mediator variable 2 (i.e., the product of the A5 and B2 coefficients). |
| A6B2 | The indirect effect of Northern Ireland ASSIST compared to the Northern Ireland Control group on the outcome variable via mediator variable 2 (i.e., the product of the A6 and B2 coefficients). |
| A7B2 | The indirect effect of Bogotá Dead Cool compared to the Northern Ireland Control group on the outcome variable via mediator variable 2 (i.e., the product of the A7 and B2 coefficients). |
| A8B2 | The indirect effect of Bogotá ASSIST compared to the Northern Ireland Control group on the outcome variable via mediator variable 2 (i.e., the product of the A8 and B2 coefficients). |
| AB1 | The total indirect effect of Northern Ireland Dead Cool, Northern Ireland ASSIST, Bogotá Dead Cool, and Bogotá ASSIST compared to the Northern Ireland Control group on the outcome variable via mediator variable 1 (i.e., the sum of A1B1, A2B1, A3B1, and A4B1). |
| AB2 | The total indirect effect of Northern Ireland Dead Cool, Northern Ireland ASSIST, Bogotá Dead Cool, and Bogotá ASSIST compared to the Northern Ireland Control group on the outcome variable via mediator variable 2 (i.e., the sum of A5B2, A6B2, A7B2, and A8B2). |
| AB | The total indirect effect of Northern Ireland Dead Cool, Northern Ireland ASSIST, Bogotá Dead Cool, and Bogotá ASSIST compared to the Northern Ireland Control group on the outcome variable via mediator variables 1 and 2 (i.e., the sum of AB1 and AB2). |
| CP1 | Coefficient representing the direct effect of Northern Ireland Dead Cool compared to the Northern Ireland Control group (i.e., the effect of predictor variable 1 on the outcome variable adjusted for the mediators). |
| CP2 | Coefficient representing the direct effect of Northern Ireland ASSIST compared to the Northern Ireland Control group (i.e., the effect of predictor variable 2 on the outcome variable adjusted for the mediators). |
| CP3 | Coefficient representing the direct effect of Bogotá Dead Cool compared to the Northern Ireland Control group (i.e., the effect of predictor variable 3 on the outcome variable adjusted for the mediators). |
| CP4 | Coefficient representing the direct effect of Bogotá ASSIST compared to the Northern Ireland Control group (i.e., the effect of predictor variable 4 on the outcome variable adjusted for the mediators). |
| CP | The direct effect of Northern Ireland Dead Cool, Northern Ireland ASSIST, Bogotá Dead Cool, and Bogotá ASSIST compared to the Northern Ireland Control group on the outcome variable adjusted for the mediators (i.e., the sum of CP1, CP2, CP3, and CP4). |
| C1 | Coefficient representing the total effect of Northern Ireland Dead Cool compared to the Northern Ireland Control group (i.e., the effect of predictor variable 1 on the outcome variable unadjusted for the mediators). |
| C2 | Coefficient representing the total effect of Northern Ireland ASSIST compared to the Northern Ireland Control group (i.e., the effect of predictor variable 2 on the outcome variable unadjusted for the mediator). |
| C3 | Coefficient representing the total effect of Bogotá Dead Cool compared to the Northern Ireland Control group (i.e., the effect of predictor variable 3 on the outcome variable unadjusted for the mediators). |
| C4 | Coefficient representing the total effect of Bogotá ASSIST compared to the Northern Ireland Control group (i.e., the effect of predictor variable 4 on the outcome variable unadjusted for the mediators). |
| C | The total effect of Northern Ireland Dead Cool, Northern Ireland ASSIST, Bogotá Dead Cool, and Bogotá ASSIST compared to the Northern Ireland Control group on the outcome variable unadjusted for the mediators (i.e., the sum of C1, C2, C3, and C4). |
| sem | Function to fit a structural equation model (lavaan package) (24). |
| test = "yuan.bentler" | Requests the robust versions of the model fit statistics, similar to the robust maximum likelihood (MLR) estimator. |
| std.lv | Unstandardized parameter estimates (standardizes all latent variables in the model only). |
| std.all | Standardized parameter estimates (standardizes all latent and observed variables in the model). |
| ML | Maximum-likelihood estimator. |
| fiml | Full-information maximum likelihood. |
| parameterEstimates | Function to report parameter estimates for a structural equation model (lavaan package) (24). In this example, bias-corrected bootstrap standard errors are requested (with 10,000 iterations). |
| standardizedSolution_boot_ci | Function to form bootstrap confidence intervals for the standardized solution (lavaan package) (24). |
| fitMeasures | Function to return the model fit statistics (lavaan package) (24). |

Note: The results of this model are reported at the top of Supplementary Tables S70 and S71.

## Example syntax for multiple mediator models including predictor variables comparing MECHANISMS setting/intervention groups with the Northern Ireland control group, with ASSIST non-peer supporters and ASSIST peer supporters analyzed separately (sensitivity analyses).

model <- 'FU_Smoking ~ B1*FU_Intentions + B2*FU_AdvertShops + CP1*Group1 + CP2*Group2 + CP3*Group3 + CP4*Group4 + CP5*Group5 + CP6*Group6 + Smoking + Intentions + AdvertShops + Gender + Age1 + Age2 + Ethnicity + SES1 + SES2

FU_Intentions ~ A1*Group1 + A2*Group2 + A3*Group3 + A4*Group4 + A5*Group5 + A6*Group6 + Smoking + Intentions + AdvertShops + Gender + Age1 + Age2 + Ethnicity + SES1 + SES2

FU_AdvertShops ~ A7*Group1 + A8*Group2 + A9*Group3 + A10*Group4 + A11*Group5 + A12*Group6 + Smoking + Intentions + AdvertShops + Gender + Age1 + Age2 + Ethnicity + SES1 + SES2

A1B1 := A1 * B1

A2B1 := A2 * B1

A3B1 := A3 * B1

A4B1 := A4 * B1

A5B1 := A5 * B1

A6B1 := A6 * B1

A7B2 := A7 * B2

A8B2 := A8 * B2

A9B2 := A9 * B2

A10B2 := A10 * B2

A11B2 := A11 * B2

A12B2 := A12 * B2

C1 := CP1 + A1B1 + A7B2

C2 := CP2 + A2B1 + A8B2

C3 := CP3 + A3B1 + A9B2

C4 := CP4 + A4B1 + A10B2

C5 := CP5 + A5B1 + A11B2

C6 := CP6 + A6B1 + A12B2

AB1 := A1B1 + A2B1 + A3B1 + A4B1 + A5B1 + A6B1

AB2 := A7B2 + A8B2 + A9B2 + A10B2 + A11B2 + A12B2

AB := AB1 + AB2

CP := CP1 + CP2 + CP3 + CP4 + CP5 + CP6

C := C1 + C2 + C3 + C4 + C5 + C6'

set.seed(1234)

fit <- sem(model = model, data = data, std.lv = TRUE, estimator="ML", missing="fiml", se = "bootstrap", bootstrap = 10000, test = "yuan.bentler")

summary(fit, fit.measures=TRUE, standardized=TRUE, rsquare=TRUE)

parameterEstimates(fit, level=0.95, boot.ci.type = "bca.simple", standardized = TRUE)

standardizedSolution_boot_ci(fit, level=0.95, type="std.all")

fitMeasures(fit)

**Key**

| FU_Smoking | Participants' responses to the self-report smoking behavior outcome at follow-up (outcome variable). |
| --- | --- |
| FU_Intentions | Participants' responses to the self-report smoking intentions outcome at follow-up (mediator variable). |
| Group1 | Dummy variable representing setting/intervention group (1=Northern Ireland Dead Cool; 0=otherwise). Base category=Northern Ireland Control group (predictor variable 1). |
| Group2 | Dummy variable representing setting/intervention group (1=Northern Ireland ASSIST non-peer supporters; 0=otherwise). Base category=Northern Ireland Control group (predictor variable 2). |
| Group3 | Dummy variable representing setting/intervention group (1=Northern Ireland ASSIST peer supporters; 0=otherwise). Base category=Northern Ireland Control group (predictor variable 3). |
| Group4 | Dummy variable representing setting/intervention group (1=Bogotá Dead Cool; 0=otherwise). Base category=Northern Ireland Control group (predictor variable 4). |
| Group5 | Dummy variable representing setting/intervention group (1=Bogotá ASSIST non-peer supporters; 0=otherwise). Base category=Northern Ireland Control group (predictor variable 5). |
| Group6 | Dummy variable representing setting/intervention group (1=Bogotá ASSIST peer supporters; 0=otherwise). Base category=Northern Ireland Control group (predictor variable 6). |
| Smoking | Participants' responses to the self-report smoking behavior outcome at baseline. |
| Intentions | Participants' responses to the self-report smoking intentions outcome at baseline. |
| Gender | Dummy variable representing participant gender (0=boy; 1=girl/prefer not to say). |
| Age1 | Dummy variable representing participant age (1=13 years; 0=otherwise). Base category=12 years or less. |
| Age2 | Dummy variable representing participant age (1=14 years or more; 0=otherwise). Base category=12 years or less. |
| Ethnicity | Dummy variable representing participant ethnicity (0=No ethnic minority; 1=ethnic minority). |
| SES1 | Dummy variable representing participant socio-economic status (1=Middle-Low/Middle [296.6<NIMDM2017≤593.2 in NI]; 0=otherwise). Base category=Informal settlement/Lowest/Low [NIMDM2017≤296.6 in NI]. |
| SES2 | Dummy variable representing participant socio-economic status (1=Middle-High/High [NIMDM2017>593.2 in NI]; 0=otherwise). Base category=Informal settlement/Lowest/Low [NIMDM2017≤296.6 in NI]. |
| ~ | Regression operator, indicates “is regressed on” (lavaan package) (24). |
| := | Defined parameter operator (lavaan package) (24). |
| A1 | Coefficient representing the effect of Northern Ireland Dead Cool compared to the Northern Ireland Control group on mediator variable 1 (FU_Intentions). |
| A2 | Coefficient representing the effect of Northern Ireland ASSIST non-peer supporters compared to the Northern Ireland Control group on mediator variable 1 (FU_Intentions). |
| A3 | Coefficient representing the effect of Northern Ireland ASSIST peer supporters compared to the Northern Ireland Control group on mediator variable 1 (FU_Intentions). |
| A4 | Coefficient representing the effect of Bogotá Dead Cool compared to the Northern Ireland Control group on mediator variable 1 (FU_Intentions). |
| A5 | Coefficient representing the effect of Bogotá ASSIST non-peer supporters compared to the Northern Ireland Control group on mediator variable 1 (FU_Intentions). |
| A6 | Coefficient representing the effect of Bogotá ASSIST peer supporters compared to the Northern Ireland Control group on mediator variable 1 (FU_Intentions). |
| A7 | Coefficient representing the effect of Northern Ireland Dead Cool compared to the Northern Ireland Control group on mediator variable 2 (FU_AdvertShops). |
| A8 | Coefficient representing the effect of Northern Ireland ASSIST non-peer supporters compared to the Northern Ireland Control group on mediator variable 2 (FU_AdvertShops). |
| A9 | Coefficient representing the effect of Northern Ireland ASSIST peer supporters compared to the Northern Ireland Control group on mediator variable 2 (FU_AdvertShops). |
| A10 | Coefficient representing the effect of Bogotá Dead Cool compared to the Northern Ireland Control group on mediator variable 2 (FU_AdvertShops). |
| A11 | Coefficient representing the effect of Bogotá ASSIST non-peer supporters compared to the Northern Ireland Control group on mediator variable 2 (FU_AdvertShops). |
| A12 | Coefficient representing the effect of Bogotá ASSIST peer supporters compared to the Northern Ireland Control group on mediator variable 2 (FU_AdvertShops). |
| B1 | Coefficient representing the association of mediator variable 1 (FU_Intentions) with the outcome variable (FU_Smoking). |
| B2 | Coefficient representing the association of mediator variable 1 (FU_AdvertShops) with the outcome variable (FU_Smoking). |
| A1B1 | The indirect effect of Northern Ireland Dead Cool compared to the Northern Ireland Control group on the outcome variable via mediator variable 1 (i.e., the product of the A1 and B1 coefficients). |
| A2B1 | The indirect effect of Northern Ireland ASSIST non-peer supporters compared to the Northern Ireland Control group on the outcome variable via mediator variable 1 (i.e., the product of the A2 and B1 coefficients). |
| A3B1 | The indirect effect of Northern Ireland ASSIST peer supporters compared to the Northern Ireland Control group on the outcome variable via mediator variable 1 (i.e., the product of the A3 and B1 coefficients). |
| A4B1 | The indirect effect of Bogotá Dead Cool compared to the Northern Ireland Control group on the outcome variable via mediator variable 1 (i.e., the product of the A4 and B1 coefficients). |
| A5B1 | The indirect effect of Bogotá ASSIST non-peer supporters compared to the Northern Ireland Control group on the outcome variable via mediator variable 1 (i.e., the product of the A5 and B1 coefficients). |
| A6B1 | The indirect effect of Bogotá ASSIST peer supporters compared to the Northern Ireland Control group on the outcome variable via mediator variable 1 (i.e., the product of the A6 and B1 coefficients). |
| A7B2 | The indirect effect of Northern Ireland Dead Cool compared to the Northern Ireland Control group on the outcome variable via mediator variable 2 (i.e., the product of the A7 and B2 coefficients). |
| A8B2 | The indirect effect of Northern Ireland ASSIST non-peer supporters compared to the Northern Ireland Control group on the outcome variable via mediator variable 2 (i.e., the product of the A8 and B2 coefficients). |
| A9B2 | The indirect effect of Northern Ireland ASSIST peer supporters compared to the Northern Ireland Control group on the outcome variable via mediator variable 2 (i.e., the product of the A9 and B2 coefficients). |
| A10B2 | The indirect effect of Bogotá Dead Cool compared to the Northern Ireland Control group on the outcome variable via mediator variable 2 (i.e., the product of the A10 and B2 coefficients). |
| A11B2 | The indirect effect of Bogotá ASSIST non-peer supporters compared to the Northern Ireland Control group on the outcome variable via mediator variable 2 (i.e., the product of the A11 and B2 coefficients). |
| A12B2 | The indirect effect of Bogotá ASSIST peer supporters compared to the Northern Ireland Control group on the outcome variable via mediator variable 2 (i.e., the product of the A12 and B2 coefficients). |
| AB1 | The total indirect effect of Northern Ireland Dead Cool, Northern Ireland ASSIST non-peer supporters, Northern Ireland ASSIST peer supporters, Bogotá Dead Cool, Bogotá ASSIST non-peer supporters, and Bogotá ASSIST peer supporters compared to the Northern Ireland Control group on the outcome variable via mediator variable 1 (i.e., the sum of A1B1, A2B1, A3B1, A4B1, A5B1, and A6B1). |
| AB2 | The total indirect effect of Northern Ireland Dead Cool, Northern Ireland ASSIST non-peer supporters, Northern Ireland ASSIST peer supporters, Bogotá Dead Cool, Bogotá ASSIST non-peer supporters, and Bogotá ASSIST peer supporters compared to the Northern Ireland Control group on the outcome variable via mediator variable 2 (i.e., the sum of A7B2, A8B2, A9B2, A10B2, A11B2, and A12B2). |
| AB | The total indirect effect of Northern Ireland Dead Cool, Northern Ireland ASSIST non-peer supporters, Northern Ireland ASSIST peer supporters, Bogotá Dead Cool, Bogotá ASSIST non-peer supporters, and Bogotá ASSIST peer supporters compared to the Northern Ireland Control group on the outcome variable via mediator variables 1 and 2 (i.e., the sum of AB1 and AB2). |
| CP1 | Coefficient representing the direct effect of Northern Ireland Dead Cool compared to the Northern Ireland Control group (i.e., the effect of predictor variable 1 on the outcome variable adjusted for the mediators). |
| CP2 | Coefficient representing the direct effect of Northern Ireland ASSIST non-peer supporters compared to the Northern Ireland Control group (i.e., the effect of predictor variable 2 on the outcome variable adjusted for the mediators). |
| CP3 | Coefficient representing the direct effect of Northern Ireland ASSIST peer supporters compared to the Northern Ireland Control group (i.e., the effect of predictor variable 3 on the outcome variable adjusted for the mediators). |
| CP4 | Coefficient representing the direct effect of Bogotá Dead Cool compared to the Northern Ireland Control group (i.e., the effect of predictor variable 4 on the outcome variable adjusted for the mediators). |
| CP5 | Coefficient representing the direct effect of Bogotá ASSIST non-peer supporters compared to the Northern Ireland Control group (i.e., the effect of predictor variable 5 on the outcome variable adjusted for the mediators). |
| CP6 | Coefficient representing the direct effect of Bogotá ASSIST peer supporters compared to the Northern Ireland Control group (i.e., the effect of predictor variable 6 on the outcome variable adjusted for the mediators). |
| CP | The direct effect of Northern Ireland Dead Cool, Northern Ireland ASSIST non-peer supporters, Northern Ireland ASSIST peer supporters, Bogotá Dead Cool, Bogotá ASSIST non-peer supporters, and Bogotá ASSIST peer supporters compared to the Northern Ireland Control group on the outcome variable adjusted for the mediators (i.e., the sum of CP1, CP2, CP3, CP4, CP5, and CP6). |
| C1 | Coefficient representing the total effect of Northern Ireland Dead Cool compared to the Northern Ireland Control group (i.e., the effect of predictor variable 1 on the outcome variable unadjusted for the mediators). |
| C2 | Coefficient representing the total effect of Northern Ireland ASSIST non-peer supporters compared to the Northern Ireland Control group (i.e., the effect of predictor variable 2 on the outcome variable unadjusted for the mediators). |
| C3 | Coefficient representing the total effect of Northern Ireland ASSIST peer supporters compared to the Northern Ireland Control group (i.e., the effect of predictor variable 3 on the outcome variable unadjusted for the mediators). |
| C4 | Coefficient representing the total effect of Bogotá Dead Cool compared to the Northern Ireland Control group (i.e., the effect of predictor variable 4 on the outcome variable unadjusted for the mediators). |
| C5 | Coefficient representing the total effect of Bogotá ASSIST non-peer supporters compared to the Northern Ireland Control group (i.e., the effect of predictor variable 5 on the outcome variable unadjusted for the mediators). |
| C6 | Coefficient representing the total effect of Bogotá ASSIST peer supporters compared to the Northern Ireland Control group (i.e., the effect of predictor variable 6 on the outcome variable unadjusted for the mediators). |
| C | The total effect of Northern Ireland Dead Cool, Northern Ireland ASSIST non-peer supporters, Northern Ireland ASSIST peer supporters, Bogotá Dead Cool, Bogotá ASSIST non-peer supporters, and Bogotá ASSIST peer supporters compared to the Northern Ireland Control group on the outcome variable unadjusted for the mediators (i.e., the sum of C1, C2, C3, C4, C5, and C6). |
| sem | Function to fit a structural equation model (lavaan package) (24). |
| test = "yuan.bentler" | Requests the robust versions of the model fit statistics, similar to the robust maximum likelihood (MLR) estimator. |
| std.lv | Unstandardized parameter estimates (standardizes all latent variables in the model only). |
| std.all | Standardized parameter estimates (standardizes all latent and observed variables in the model). |
| ML | Maximum-likelihood estimator. |
| fiml | Full-information maximum likelihood. |
| parameterEstimates | Function to report parameter estimates for a structural equation model (lavaan package) (24). In this example, bias-corrected bootstrap standard errors are requested (with 10,000 iterations). |
| standardizedSolution_boot_ci | Function to form bootstrap confidence intervals for the standardized solution (lavaan package) (24). |
| fitMeasures | Function to return the model fit statistics (lavaan package) (24). |

Note: The results of this model are reported at the top of Supplementary Tables S74 and S75.

## Example syntax for Monte Carlo based statistical power analysis for mediation models.

model <- 'RiskSocial =~ start(20.4358243828813)*RS1 + start(21.7009294893858)*RS2 + start(18.1803296860002)*RS3

FU_RiskSocial =~ start(20.4358243828813)*FU_RS1 + start(21.7009294893858)*FU_RS2 + start(18.1803296860002)*FU_RS3

RS1 ~~ start(149.5690861447233)*FU_RS1

RS2 ~~ start(124.0865704892344)*FU_RS2

RS3 ~~ start(315.5666163578309)*FU_RS3

RS1 + FU_RS1 ~ start(75.2332813462424)*1

RS2 ~ start(70.2862703156870)*1

FU_RS2 ~ start(74.1927144309554)*1

RS3 + FU_RS3 ~ start(59.0478483658336)*1

RiskSocial ~~ start(1)*RiskSocial

FU_RiskSocial ~~ start(1)*FU_RiskSocial

RS1 ~~ start(557.9133369092744)*RS1

RS2 ~~ start(484.4368372560621)*RS2

RS3 ~~ start(863.3085034335868)*RS3

FU_RS1 ~~ start(557.9133369092744)*FU_RS1

FU_RS2 ~~ start(484.4368372560621)*FU_RS2

FU_RS3 ~~ start(863.3085034335868)*FU_RS3

FU_Donation ~ B1*FU_RiskSocial + start(0.2007033029261)*FU_RiskSocial + B2*FU_AdvertMedia + start(-0.0835793812653)*FU_AdvertMedia + CP*Intervention + start(0.2274177541938)*Intervention + start(0.6290349199657)*Setting + start(0.2655595401863)*Donation + start(-0.0652462047294)*RiskSocial + start(-0.0487314256073)*AdvertMedia + start(0.1998185886214)*Gender + start(-0.0189259660151)*Age1 + start(0.1075043342340)*Age2 + start(0.3488825742238)*Ethnicity + start(-0.2712155001097)*SES1 + start(-0.1178192407124)*SES2

FU_RiskSocial ~ A1*Intervention + start(-0.1462106277070)*Intervention + start(-0.2044426361243)*Setting + start(0.0240503496371)*Donation + start(0.4711407809713)*RiskSocial + start(0.0045190487662)*AdvertMedia + start(-0.0188777668414)*Gender + start(0.0794458715487)*Age1 + start(-0.0853987838363)*Age2 + start(0.0953591751715)*Ethnicity + start(0.0211749130986)*SES1 + start(0.0485357800165)*SES2

FU_AdvertMedia ~ A2*Intervention + start(-1.1858508826104)*Intervention + start(0.6347530515335)*Setting + start(-0.0289668047933)*Donation + start(0.1237084199874)*RiskSocial + start(0.4479553324737)*AdvertMedia + start(0.0480563809988)*Gender + start(0.1895049118868)*Age1 + start(0.4921336421205)*Age2 + start(-0.1378287449983)*Ethnicity + start(0.1830199059320)*SES1 + start(-0.0858872054165)*SES2'

indirect <- 'A1B1 := A1 * B1

A2B2 := A2 * B2

AB := A1B1 + A2B2

C := CP + AB'

set.seed(1234)

Power <- power.boot(model = model, indirect = indirect, nobs = nobs, nrep = 1000, nboot = 1000, alpha = 0.95, ovnames = names, se = "bootstrap", boot.type = "bca.simple", ci="BC", estimator = "ML", parallel = "parallel", ncore = 4)

summary(Power)

**Key**

| FU_Donation | Participants' responses to the experimental outcome (Donation to ASSIST/Dead Cool) at follow-up (outcome variable). |
| --- | --- |
| Donation | Participants' responses to the experimental outcome (Donation to ASSIST/Dead Cool) at baseline. |
| FU_RiskSocial | Latent variable representing perceived social risks of smoking at follow-up, derived from items FU_RS1 to FU_RS3 (mediator variable 1). |
| RiskSocial | Latent variable representing perceived social risks of smoking at baseline, derived from items RS1 to RS3. |
| FU_AdvertMedia | Participants' responses to exposure to advertising in the media at follow-up (mediator variable 2). |
| AdvertMedia | Participants' responses to exposure to advertising in the media at baseline. |
| RS1 | Participants' observed scores for item RS1 at baseline (similar definitions apply for items RS2 and RS3). |
| FU_RS1 | Participants' observed scores for item RS1 at follow-up (similar definitions apply for items FU_RS2 and FU_RS3). |
| Intervention | Dummy variable representing intervention group (0=Dead Cool, 1=ASSIST). |
| Setting | Dummy variable representing setting (0=Northern Ireland, 1=Bogotá). |
| Gender | Dummy variable representing participant gender (0=boy; 1=girl/prefer not to say). |
| Age1 | Dummy variable representing participant age (1=13 years; 0=otherwise). Base category=12 years or less. |
| Age2 | Dummy variable representing participant age (1=14 years or more; 0=otherwise). Base category=12 years or less. |
| Ethnicity | Dummy variable representing participant ethnicity (0=No ethnic minority; 1=ethnic minority). |
| SES1 | Dummy variable representing participant socio-economic status (1=Middle-Low/Middle [296.6<NIMDM2017≤593.2 in NI]; 0=otherwise). Base category=Informal settlement/Lowest/Low [NIMDM2017≤296.6 in NI]. |
| SES2 | Dummy variable representing participant socio-economic status (1=Middle-High/High [NIMDM2017>593.2 in NI]; 0=otherwise). Base category=Informal settlement/Lowest/Low [NIMDM2017≤296.6 in NI]. |
| =~ | Latent variable operator, indicates “is measured by” (lavaan package) (24). |
| ~~ | Covariance operator, indicates “is correlated with” (lavaan package) (24). |
| ~1 | Intercept operator, defines the intercept (lavaan package) (24). |
| * | Used to define parameter labels and specify starting values. |
| start() | Used to specify starting values for each parameter. The values in brackets are the parameter estimates extracted from the lavaan model output. |
| A1 | Coefficient representing the predictor variable (Intervention) effect on mediator variable 1 (FU_RiskSocial). |
| A2 | Coefficient representing the predictor variable (Intervention) effect on mediator variable 2 (FU_AdvertMedia). |
| B1 | Coefficient representing the association of mediator variable 1 (FU_RiskSocial) with the outcome variable (FU_Donation). |
| B2 | Coefficient representing the association of mediator variable 2 (FU_AdvertMedia) with the outcome variable (FU_Donation). |
| A1B1 | Coefficient representing the indirect effect of the predictor variable on the outcome variable via mediator variable 1 (i.e., the product of the A1 and B1 coefficients). |
| A2B2 | Coefficient representing the indirect effect of the predictor variable on the outcome variable via mediator variable 2 (i.e., the product of the A2 and B2 coefficients). |
| AB | Coefficient representing the indirect effect of the predictor variable on the outcome variable via all mediators included in the model (i.e., the sum of the indirect effects via each individual mediator). |
| CP | Coefficient representing the direct effect (i.e., the effect of the predictor variable on the outcome variable adjusted for the mediators). |
| C | Coefficient representing the total effect (i.e., the effect of the predictor variable on the outcome variable unadjusted for the mediators). |
| power.boot | Function to run Monte Carlo based statistical power analysis for mediation models ('bmem' package in R) (39,40). |
| model | Indicates the model specified with lavaan notation. |
| indirect | Indicates the indirect effect specified with lavaan notation. |
| nobs | Number of observations reported in the lavaan model output. |
| nrep | Number of replications for Monte Carlo simulation |
| nboot | Number of bootstrap draws. |
| alpha = 0.95 | The significance level used to compute confidence intervals for the model parameters. |
| ovnames = names | A vector containing the names of the observed variables. |
| se = "bootstrap", boot.type = "bca.simple", ci="BC" | Requests bias-corrected bootstrap standard errors and confidence intervals. |
| ML | Maximum-likelihood estimator. |
| parallel = "parallel", ncore = 4 | Requests parallel processing using 4 cores. |

Note: The results of this model pertains to the power calculation reported at the top of Table 7.

# Supplementary Figures

## Supplementary Figure S1. Study Flow diagram for MECHANISMS schools.

**CONSORT 2010 Flow Diagram**

Participated in follow-up (n=720)

(n=389 NI, n=331 Bogotá)

Received **Dead Cool** intervention (n=600)

(n=286 NI, n=314 Bogotá)

Received **ASSIST** intervention (n=745)

(n=405 NI, n=340 Bogotá)

Selected (N=12)

(N=6 NI, N=6 Bogotá)

**Follow-Up**

Entered the school (n=0)

Left the school (n=8)

Non-attendance to school (n=6)

Withdrawn (n=11)

**Excluded (n=51)**

♦  Declined to participate (n=43)

♦  Left the school (n=4)

♦  No response (n=4)

Assessed for eligibility

(n=648 children)

Assessed for eligibility

(n=796 children)

Allocated to **Dead Cool** intervention (N=6)

(N=3 NI, N=3 Bogotá)

Allocated to **ASSIST** intervention (N=6)

(N=3 NI, N=3 Bogotá)

**Allocation**

**Excluded (N=45)**

♦  Declined to participate (N=4)

♦  No answer (N=8)

♦  Unsuitable (N=28)

♦  Offered alternative (N=5)

Assessed for eligibility

(N=57 schools)

**Enrollment**

**Analysis**

**Excluded (n=48)**

♦  Declined to participate (n=45)

♦  Left the school (n=2)

♦  No response (n=1)

Left the school (n=7)

Non-attendance to school (n=8)

Withdrawn (n=2)

Entered the school (n=2)

Participated in follow-up (n=585)

(n=281 NI, n=304 Bogotá)

**Analysed (n=704)**

♦  Not comparable T1 vs T2 (n=16)

♦  Missing data (n=0)

**Analysed (n=579)**

♦  Not comparable T1 vs T2 (n=6)

♦  Missing data (n=0)

## Supplementary Figure S2. Proposed logic model for Dead Cool intervention from the published MECHANISMS study protocol*.

-Self-efficacy to quit tobacco/remain tobacco-free

-Intentions toward tobacco-related behaviours

-Knowledge about tobacco and tobacco-related behaviours

-Attitudes toward tobacco-related behaviours

-Perceived behavioural control

-Perceived risks of tobacco use

-Perceived benefits of tobacco use

-Perceived prevalence of peer smoking (descriptive norm)

-Perceived parental/family approval (injunctive norm)

-Perceived peer approval (injunctive norm)

-Exposure to advertising (media and shops).

^1^BCTs are coded based on Michie and colleagues' 93-item taxonomy:

Michie S, Richardson M, Johnston M, et al. The behavior change technique taxonomy (v1) of 93 hierarchically clustered techniques: building an international consensus for the reporting of behavior change interventions. Ann Behav Med. 2013;46(1):81-95.

-Increased knowledge about tobacco.

-Increased knowledge about long-term health consequences.

-Fear arousal and anticipated regret.

-Increased awareness of factors influencing adolescent tobacco –related behaviour (e.g. influence of friends, parents/carers/family, media).

-Increased awareness of availability of support and how to seek support from family/friends.

-Reduce smoking intention (increase intentions to quit for adolescents who are already engaging in tobacco-related behaviours).

-Alignment of perceived (descriptive) social norms with actual social norms.

**Context**

-Appropriate school rules.

-Family and peer availability and exposure.

-School policy and enforcement.

-School exposure or accessibility.

Theory of Planned Behaviour

**Reduced morbidity and mortality.**

**Improved health and mental wellbeing.**

**Planned behaviour**

-Aversion to tobacco use.

-Delayed average age at first use.

-Reduced initiation of tobacco-related behaviours.

-Longer-term reduction in intention to engage in tobacco-related behaviours

**Possible Mediators**

Five-week intervention (DVD and four lessons) with information about consequences, social support, and problem solving.

The following BCTs are included: Information about health consequences; Information about social and environmental consequences; Information about emotional consequences; Salience of consequences; Anticipated regret; Information about antecedents; Pros and cons; Demonstration of the behaviour; Problem solving; Behavioural practice/rehearsal; Social comparison; Information about others' approval; Identification of self as role model; Social support; Credible source.

**Short term outcomes**

**Long term outcomes**

-Increased family and peer support.

**Medium term outcomes**

-Increase in planned behaviour strategies (e.g. how to refuse cigarettes).

-Increased self-efficacy to quit tobacco/remain tobacco-free.

-Knowledge and attitude change towards tobacco-related behaviours (e.g. negative attitude towards smoking).

-Perceived social norms and informed decision making geared toward NOT engaging in tobacco-related behaviours.

**Intervention^1^**

-Intention to NOT engage in tobacco-related behaviours.

-Increased well-being and reduced deviant behaviour.

**Underpinning Theoretical Framework**

*Note: This figure is reproduced under a Creative Commons license (CC BY 2020) from Hunter RF, Montes F, Murray JM, et al. MECHANISMS Study: using Game Theory to assess the effects of social norms and social networks on adolescent smoking in schools—study protocol. Front Public Heal 2020; 8: 377. doi:10.3389/fpubh.2020.00377 (16). BCT = Behavior change technique.

## The Dead Cool program.

Supplementary Figure S2 shows the proposed logic model depicting assumed pathways of change for participants in the Dead Cool intervention. The intervention lasts for eight weeks and consists of a DVD and eight lessons delivered by teachers in participating schools (15,41). It is based on the theory of planned behavior (14,42) and includes a range of behavior change techniques (BCTs) including provision of information about consequences, information about behavioral antecedents (e.g., influence from friends, family and the media), problem solving, and social support. In the short-term it is hypothesized that the intervention's information provision components should lead directly to increased knowledge about tobacco and the long-term health consequences, with emphasis on the salience of outcomes leading to fear arousal and anticipated regret (43,44). Provision of normative information regarding the prevalence of smoking and tobacco related behaviors for the participants' age group should reduce 'misperceptions' and act to align perceived social (descriptive) norms with actual norms (45–47). The intervention should lead to increased awareness of the sources of support available, and of how to seek support from family and friends. Self-efficacy to quit tobacco, or remain tobacco-free, should be increased by increases in social support (i.e., vicarious experience) (48). In the medium term, increases in perceived social support from family and peers, changing attitudes towards tobacco-related behaviors (e.g., negative attitude towards smoking) and changes in perceived social norms should lead participants to form intentions not to smoke or engage in tobacco-related behaviors (14,42). In the longer-term, the intervention is expected to lead to reduced rates of initiation of tobacco-related behaviors, delayed average age at first tobacco-use, reduced morbidity and mortality, and improved health and mental wellbeing (49–52).

Hypothesized mediators include:

- Self-efficacy to quit tobacco/remain tobacco-free;
- Perceived behavioral control;
- Perceived risks of tobacco use;
- Perceived benefits of tobacco use;
- Perceived prevalence of peer smoking (descriptive norm);
- Perceived parental/family approval (injunctive norm);
- Perceived peer approval (injunctive norm);
- Exposure to advertising (media);
- Exposure to advertising (shops).

Hypothesized outcomes include:

- Engagement in tobacco-related behaviors;
- Intentions toward tobacco-related behaviors;
- Knowledge about tobacco and tobacco-related behaviors;
- Attitudes toward tobacco-related behaviors.

##

## Supplementary Figure S3. Proposed logic model for ASSIST intervention from the published MECHANISMS study protocol*.

-Self-efficacy to quit tobacco/remain tobacco-free

-Knowledge about tobacco and tobacco-related behaviours

-Perceived risks of tobacco use

-Perceived benefits of tobacco use

-Perceived behavioural control

-Attitudes toward tobacco-related behaviours

-Intentions toward tobacco-related behaviours

-Perceived prevalence of peer smoking (descriptive norm)

-Perceived peer approval (injunctive norm).

Ten-week intervention (two-day training of peer supporters and four follow-up sessions) with a focus on building skills necessary to be an effective peer supporter, and information provision on tobacco.

**Being an effective peer supporter**

The following BCTs are included: Identification of self as role model; Valued self-identity; Verbal persuasion about capability; Focus on past success; Demonstration of the behaviour; Goal setting (behaviour); Action planning; Prompts/cues; Behavioural practice/rehearsal; Self-monitoring of behaviour; Feedback on behaviour; Monitoring of emotional consequences; Problem solving; Social support (unspecified); Social support (practical); Social comparison; Social reward; Non-specific incentive; Non-specific reward; Rewarding completion.

**Smoking prevention**

The following BCTs are included: Information about health consequences; Information about social and environmental consequences; Information about emotional consequences; Information about antecedents; Pros and cons; Identification of self as role model; Social comparison; Adding objects to the environment.

^1^BCTs are coded based on Michie and colleagues' 93-item taxonomy:

Michie S, Richardson M, Johnston M, et al. The behavior change technique taxonomy (v1) of 93 hierarchically clustered techniques: building an international consensus for the reporting of behavior change interventions. Ann Behav Med. 2013;46(1):81-95.

**Context**

-Social system interconnectedness.

-Appropriate school rules.

-School policy and enforcement.

-School exposure or accessibility.

-Relative advantage, compatibility, complexity, trialability, observability.

**Confirmation**

-**Early adopters and early majority** gain support (on-going from peer supporters) for decision to implement ***the innovation***.

-Remaining individuals (i.e. **late majority and laggards**) encouraged to undergo same process (**Knowledge-Confirmation**) via increased *peer support*, changed *social norms* and role modelling over ***time***.

Communication channels

**Reduced morbidity and mortality.**

**Improved health and mental wellbeing.**

-Aversion to tobacco use.

-Delayed average age at first use.

-Reduced initiation of tobacco-related behaviours.

-Longer-term reduction in intention to engage in tobacco-related behaviours.

**Implementation**

-**Early adopters and early majority** have reduced smoking *intention* (increase intentions to quit for those who are already engaging in tobacco-related behaviours) and implement ***the innovation***.

Diffusion of Innovations Theory

**Persuasion**

-*Knowledge and attitude* change towards tobacco-related behaviours (e.g. negative attitude towards smoking) for approached individuals (i.e. **early adopters, early majority**).

-Increased perception of *peer support* for **early adopters and early majority**.

**Knowledge**

-Peer supporters have increased knowledge about tobacco and the long-term health consequences which require prevention of tobacco use (i.e. ***the innovation***).

-Reduced smoking *intention* for peer supporters (increase intentions to quit for those who are already engaging in tobacco-related behaviours).

-Peer supporters approach members of the social network (i.e. friendship group) to convey accurate information about the risks and benefits of tobacco (i.e. ***communication channels, social system***).

**Possible Mediators**

**Short term outcomes**

**Medium term outcomes**

**Long term outcomes**

**Intervention^1^**

**Decision**

-**Early adopters and early majority** have increased *self-efficacy* to quit tobacco/remain tobacco-free.

-Individuals weigh up *pros and cons* of adopting ***the innovation***.

-Increased *knowledge, attitude change and perceptions of peer support* influence individuals towards adopting ***the innovation***.

**Underpinning Theoretical Framework**

*Note: This figure is reproduced under a Creative Commons license (CC BY 2020) from Hunter RF, Montes F, Murray JM, et al. MECHANISMS Study: using Game Theory to assess the effects of social norms and social networks on adolescent smoking in schools—study protocol. Front Public Heal 2020; 8: 377. doi:10.3389/fpubh.2020.00377 (16). BCT = Behavior change technique.

## The ASSIST program.

Supplementary Figure S3 shows the proposed logic model depicting assumed pathways of change for participants receiving the ASSIST program. The intervention lasts for ten weeks and consists of a two-day training course for peer supporters (18% of pupils identified as being influential within the school year group) with four follow-up sessions. It is based on the diffusion of innovations theory (13), and is intended to provide peer supporters with the knowledge and skills necessary to spread information about the harms of tobacco-use amongst the remaining members of the school year group (i.e., peer education and diffusion). According to Rogers, diffusion is “the process in which an innovation is communicated through certain channels over time among the members of a social system” (13). Therefore, the four key components in the diffusion of an innovation is the innovation (e.g., smoking prevention message), communication channels (e.g., one-to-one conversations by peer supporters in their friendship groups), time (e.g., the ten-week period during which the message is being spread), and the social system (e.g., the school year group) (13,53).

The five stages of the innovation-decision process, leading to adoption (i.e., “full use of an innovation as the best course of action available”(13,53)) or rejection (i.e., a decision “not to adopt an innovation”(13,53)) of the smoking prevention message for each individual in the school year group are: (1) Knowledge; (2) Persuasion; (3) Decision; (4) Implementation; and (5) Confirmation. Uncertainty in adoption is reduced when individuals are well informed about the pros and cons of adopting the message (13,53). Therefore, the communication channels (i.e., one-to-one conversations between peer supporters and their friends) are vital to progress the innovation-decision process from “Knowledge” to “Confirmation” for all members of the school year group. In the short term, it is hypothesized that the intervention will directly lead to increased knowledge regarding tobacco and its long-term health consequences for peer supporters. Therefore, peer supporters will have reduced intentions to engage in tobacco-related behaviors. During the “Knowledge” stage, peer supporters approach members of their social network (i.e., friendship groups) to convey accurate information about the risks and benefits (i.e., pros and cons) of tobacco use. During the “Persuasion” stage, early adopters and early majority (i.e., well-connected individuals who are first to receive the message), undergo a knowledge and attitude change towards tobacco-related behaviors (e.g., negative attitude towards smoking) and have increased perceptions of peer support. In the medium term, self-efficacy to quit tobacco, or remain tobacco-free, should be increased by increases in social support (i.e., vicarious experience) (48). During the “Decision” stage, early adopters and early majority weigh up the pros and cons of adopting the message (54). Increased knowledge, attitude changes and perceptions of peer support influence the early adopters and early majority towards implementing the innovation and reducing their smoking intentions during the “Implementation” stage (14,42). During the “Confirmation” stage, they gain support (on-going from peer supporters) for the decision to implement the innovation. Furthermore, in the medium term, the remaining individuals in the social system (i.e., late majority and laggards) who are less well-connected than the early adopters and early majority are encouraged to undergo the same process (i.e., “Knowledge” to “Confirmation”) due to increased perceptions of peer support, changed social norms and role modelling (48,55). In the longer-term, the intervention is expected to lead to reduced rates of initiation of tobacco-related behaviors, delayed average age at first tobacco-use, reduced morbidity and mortality, and improved health and mental wellbeing (49–52).

According to the diffusion of innovations theory, contextual factors influencing the rate of adoption include: relative advantage (i.e., the degree to which an innovation is perceived as being better than the preceding idea); compatibility (i.e., the degree to which an innovation is perceived as being consistent with existing values, experiences and needs of adopters); complexity (i.e., the degree to which the innovation is perceived as being difficult to understand and use); trialability (i.e., the degree to which an innovation may be experimented with on a limited basis); and observability (i.e., the degree to which the results of the innovation are observable to others via role modelling for example) (13,53).

Hypothesized mediators include:

- Self-efficacy to quit tobacco/remain tobacco-free;
- Perceived risks of tobacco use;
- Perceived benefits of tobacco use;
- Perceived behavioral control;
- Perceived prevalence of peer smoking (descriptive norm);
- Perceived peer approval (injunctive norm).

Hypothesized outcomes include:

- Engagement in tobacco-related behaviors;
- Intentions toward tobacco-related behaviors;
- Knowledge about tobacco and tobacco-related behaviors;
- Attitudes toward tobacco-related behaviors.

## Supplementary Figure S4. Histograms for experimentally measured injunctive smoking/vaping norms showing: (a) baseline distribution for all MECHANISMS schools; (b) follow-up distribution for all MECHANISMS schools; (c) distribution of changes between baseline and follow-up by intervention group; (d) distribution of changes between baseline and follow-up by setting.

|  |  |
| --- | --- |
| 1. Baseline distribution for all MECHANISMS schools. | 1. Follow-up distribution for all MECHANISMS schools. |
|  |  |
| (c) Distribution of changes between baseline and follow-up by intervention group. | (d) Distribution of changes between baseline and follow-up by setting. |

## Supplementary Figure S5. Histograms for experimentally measured descriptive smoking/vaping norms showing: (a) baseline distribution for all MECHANISMS schools; (b) follow-up distribution for all MECHANISMS schools; (c) distribution of changes between baseline and follow-up by intervention group; (d) distribution of changes between baseline and follow-up by setting.

|  |  |
| --- | --- |
| 1. Baseline distribution for all MECHANISMS schools. | 1. Follow-up distribution for all MECHANISMS schools. |
|  |  |
| (c) Distribution of changes between baseline and follow-up by intervention group. | (d) Distribution of changes between baseline and follow-up by setting. |

## Supplementary Figure S6. Histograms for experimental donations to ASSIST/Dead Cool (willingness to pay to support anti-smoking norms) showing: (a) baseline distribution for all MECHANISMS schools; (b) follow-up distribution for all MECHANISMS schools; (c) distribution of changes between baseline and follow-up by intervention group; (d) distribution of changes between baseline and follow-up by setting.

|  |  |
| --- | --- |
| 1. Baseline distribution for all MECHANISMS schools. | 1. Follow-up distribution for all MECHANISMS schools. |
|  |  |
| (c) Distribution of changes between baseline and follow-up by intervention group. | (d) Distribution of changes between baseline and follow-up by setting. |

## Supplementary Figure S7. Histograms for self-report injunctive smoking norms: (a) baseline distribution for all MECHANISMS schools; (b) follow-up distribution for all MECHANISMS schools; (c) distribution of changes between baseline and follow-up by intervention group; (d) distribution of changes between baseline and follow-up by setting.

|  |  |
| --- | --- |
| 1. Baseline distribution for all MECHANISMS schools. | 1. Follow-up distribution for all MECHANISMS schools. |
|  |  |
| (c) Distribution of changes between baseline and follow-up by intervention group. | (d) Distribution of changes between baseline and follow-up by setting. |

## Supplementary Figure S8. Histograms for self-report descriptive smoking norms scale 1: (a) baseline distribution for all MECHANISMS schools; (b) follow-up distribution for all MECHANISMS schools; (c) distribution of changes between baseline and follow-up by intervention group; (d) distribution of changes between baseline and follow-up by setting.

|  |  |
| --- | --- |
| 1. Baseline distribution for all MECHANISMS schools. | 1. Follow-up distribution for all MECHANISMS schools. |
|  |  |
| (c) Distribution of changes between baseline and follow-up by intervention group. | (d) Distribution of changes between baseline and follow-up by setting. |

## Supplementary Figure S9. Histograms for self-report descriptive smoking norms scale 2: (a) baseline distribution for all MECHANISMS schools; (b) follow-up distribution for all MECHANISMS schools; (c) distribution of changes between baseline and follow-up by intervention group; (d) distribution of changes between baseline and follow-up by setting.

|  |  |
| --- | --- |
| 1. Baseline distribution for all MECHANISMS schools. | 1. Follow-up distribution for all MECHANISMS schools. |
|  |  |
| (c) Distribution of changes between baseline and follow-up by intervention group. | (d) Distribution of changes between baseline and follow-up by setting. |

## Supplementary Figure S10. Histograms for self-report smoking behavior: (a) baseline distribution for all MECHANISMS schools, and NI control schools; (b) follow-up distribution for all MECHANISMS schools, and NI control schools; (c) distribution of changes between baseline and follow-up by intervention group; (d) distribution of changes between baseline and follow-up by setting.

|  |  |
| --- | --- |
| 1. Baseline distribution for all MECHANISMS schools, and NI control schools. | 1. Follow-up distribution for all MECHANISMS schools, and NI control schools. |
|  |  |
| (c) Distribution of changes between baseline and follow-up by intervention group. | (d) Distribution of changes between baseline and follow-up by setting. |

## Supplementary Figure S11. Histograms for self-report smoking intentions: (a) baseline distribution for all MECHANISMS schools, and NI control schools; (b) follow-up distribution for all MECHANISMS schools, and NI control schools; (c) distribution of changes between baseline and follow-up by intervention group; (d) distribution of changes between baseline and follow-up by setting.

|  |  |
| --- | --- |
| 1. Baseline distribution for all MECHANISMS schools, and NI control schools. | 1. Follow-up distribution for all MECHANISMS schools, and NI control schools. |
|  |  |
| (c) Distribution of changes between baseline and follow-up by intervention group. | (d) Distribution of changes between baseline and follow-up by setting. |

## Supplementary Figure S12. Histograms for smoking susceptibility: (a) baseline distribution for all MECHANISMS schools, and NI control schools; (b) follow-up distribution for all MECHANISMS schools, and NI control schools; (c) distribution of changes between baseline and follow-up by intervention group; (d) distribution of changes between baseline and follow-up by setting.

|  |  |
| --- | --- |
| 1. Baseline distribution for all MECHANISMS schools, and NI control schools. | 1. Follow-up distribution for all MECHANISMS schools, and NI control schools. |
|  |  |
| (c) Distribution of changes between baseline and follow-up by intervention group. | (d) Distribution of changes between baseline and follow-up by setting. |

## Supplementary Figure S13. Histograms for knowledge of smoking: (a) baseline distribution for all MECHANISMS schools; (b) follow-up distribution for all MECHANISMS schools; (c) distribution of changes between baseline and follow-up by intervention group; (d) distribution of changes between baseline and follow-up by setting.

|  |  |
| --- | --- |
| 1. Baseline distribution for all MECHANISMS schools. | 1. Follow-up distribution for all MECHANISMS schools. |
|  |  |
| (c) Distribution of changes between baseline and follow-up by intervention group. | (d) Distribution of changes between baseline and follow-up by setting. |

## Supplementary Figure S14. Histograms for attitudes towards smoking: (a) baseline distribution for all MECHANISMS schools; (b) follow-up distribution for all MECHANISMS schools; (c) distribution of changes between baseline and follow-up by intervention group; (d) distribution of changes between baseline and follow-up by setting.

|  |  |
| --- | --- |
| 1. Baseline distribution for all MECHANISMS schools. | 1. Follow-up distribution for all MECHANISMS schools. |
|  |  |
| (c) Distribution of changes between baseline and follow-up by intervention group. | (d) Distribution of changes between baseline and follow-up by setting. |

## Supplementary Figure S15. Histograms for self-efficacy (emotional subscale): (a) baseline distribution for all MECHANISMS schools; (b) follow-up distribution for all MECHANISMS schools; (c) distribution of changes between baseline and follow-up by intervention group; (d) distribution of changes between baseline and follow-up by setting.

|  |  |
| --- | --- |
| 1. Baseline distribution for all MECHANISMS schools. | 1. Follow-up distribution for all MECHANISMS schools. |
|  |  |
| (c) Distribution of changes between baseline and follow-up by intervention group. | (d) Distribution of changes between baseline and follow-up by setting. |

## Supplementary Figure S16. Histograms for self-efficacy (friends subscale): (a) baseline distribution for all MECHANISMS schools; (b) follow-up distribution for all MECHANISMS schools; (c) distribution of changes between baseline and follow-up by intervention group; (d) distribution of changes between baseline and follow-up by setting.

|  |  |
| --- | --- |
| 1. Baseline distribution for all MECHANISMS schools. | 1. Follow-up distribution for all MECHANISMS schools. |
|  |  |
| (c) Distribution of changes between baseline and follow-up by intervention group. | (d) Distribution of changes between baseline and follow-up by setting. |

## Supplementary Figure S17. Histograms for self-efficacy (opportunity subscale): (a) baseline distribution for all MECHANISMS schools; (b) follow-up distribution for all MECHANISMS schools; (c) distribution of changes between baseline and follow-up by intervention group; (d) distribution of changes between baseline and follow-up by setting.

|  |  |
| --- | --- |
| 1. Baseline distribution for all MECHANISMS schools. | 1. Follow-up distribution for all MECHANISMS schools. |
|  |  |
| (c) Distribution of changes between baseline and follow-up by intervention group. | (d) Distribution of changes between baseline and follow-up by setting. |

## Supplementary Figure S18. Histograms for perceived physical risks: (a) baseline distribution for all MECHANISMS schools; (b) follow-up distribution for all MECHANISMS schools; (c) distribution of changes between baseline and follow-up by intervention group; (d) distribution of changes between baseline and follow-up by setting.

|  |  |
| --- | --- |
| 1. Baseline distribution for all MECHANISMS schools. | 1. Follow-up distribution for all MECHANISMS schools. |
|  |  |
| (c) Distribution of changes between baseline and follow-up by intervention group. | (d) Distribution of changes between baseline and follow-up by setting. |

## Supplementary Figure S19. Histograms for perceived social risks: (a) baseline distribution for all MECHANISMS schools; (b) follow-up distribution for all MECHANISMS schools; (c) distribution of changes between baseline and follow-up by intervention group; (d) distribution of changes between baseline and follow-up by setting.

|  |  |
| --- | --- |
| 1. Baseline distribution for all MECHANISMS schools. | 1. Follow-up distribution for all MECHANISMS schools. |
|  |  |
| (c) Distribution of changes between baseline and follow-up by intervention group. | (d) Distribution of changes between baseline and follow-up by setting. |

## Supplementary Figure S20. Histograms for perceived addiction risks: (a) baseline distribution for all MECHANISMS schools; (b) follow-up distribution for all MECHANISMS schools; (c) distribution of changes between baseline and follow-up by intervention group; (d) distribution of changes between baseline and follow-up by setting.

|  |  |
| --- | --- |
| 1. Baseline distribution for all MECHANISMS schools. | 1. Follow-up distribution for all MECHANISMS schools. |
|  |  |
| (c) Distribution of changes between baseline and follow-up by intervention group. | (d) Distribution of changes between baseline and follow-up by setting. |

## Supplementary Figure S21. Histograms for perceived benefits: (a) baseline distribution for all MECHANISMS schools; (b) follow-up distribution for all MECHANISMS schools; (c) distribution of changes between baseline and follow-up by intervention group; (d) distribution of changes between baseline and follow-up by setting.

|  |  |
| --- | --- |
| 1. Baseline distribution for all MECHANISMS schools. | 1. Follow-up distribution for all MECHANISMS schools. |
|  |  |
| (c) Distribution of changes between baseline and follow-up by intervention group. | (d) Distribution of changes between baseline and follow-up by setting. |

## Supplementary Figure S22. Histograms for perceived behavioral control (easy to quit smoking): (a) baseline distribution for all MECHANISMS schools; (b) follow-up distribution for all MECHANISMS schools; (c) distribution of changes between baseline and follow-up by intervention group; (d) distribution of changes between baseline and follow-up by setting.

|  |  |
| --- | --- |
| 1. Baseline distribution for all MECHANISMS schools. | 1. Follow-up distribution for all MECHANISMS schools. |
|  |  |
| (c) Distribution of changes between baseline and follow-up by intervention group. | (d) Distribution of changes between baseline and follow-up by setting. |

## Supplementary Figure S23. Histograms for perceived behavioral control (to avoid smoking): (a) baseline distribution for all MECHANISMS schools; (b) follow-up distribution for all MECHANISMS schools; (c) distribution of changes between baseline and follow-up by intervention group; (d) distribution of changes between baseline and follow-up by setting.

|  |  |
| --- | --- |
| 1. Baseline distribution for all MECHANISMS schools. | 1. Follow-up distribution for all MECHANISMS schools. |
|  |  |
| (c) Distribution of changes between baseline and follow-up by intervention group. | (d) Distribution of changes between baseline and follow-up by setting. |

## Supplementary Figure S24. Histograms for exposure to advertising in the media: (a) baseline distribution for all MECHANISMS schools; (b) follow-up distribution for all MECHANISMS schools; (c) distribution of changes between baseline and follow-up by intervention group; (d) distribution of changes between baseline and follow-up by setting.

|  |  |
| --- | --- |
| 1. Baseline distribution for all MECHANISMS schools. | 1. Follow-up distribution for all MECHANISMS schools. |
|  |  |
| (c) Distribution of changes between baseline and follow-up by intervention group. | (d) Distribution of changes between baseline and follow-up by setting. |

## Supplementary Figure S25. Histograms for exposure to advertising in shops: (a) baseline distribution for all MECHANISMS schools, and NI control schools; (b) follow-up distribution for all MECHANISMS schools, and NI control schools; (c) distribution of changes between baseline and follow-up by intervention group; (d) distribution of changes between baseline and follow-up by setting.

|  |  |
| --- | --- |
| 1. Baseline distribution for all MECHANISMS schools, and NI control schools. | 1. Follow-up distribution for all MECHANISMS schools, and NI control schools. |
|  |  |
| (c) Distribution of changes between baseline and follow-up by intervention group. | (d) Distribution of changes between baseline and follow-up by setting. |

## Supplementary Figure S26. Histograms for objectively measured smoking behavior: (a) baseline distribution for all MECHANISMS schools, and NI control schools; (b) follow-up distribution for all MECHANISMS schools, and NI control schools; (c) distribution of changes between baseline and follow-up by intervention group; (d) distribution of changes between baseline and follow-up by setting.

|  |  |
| --- | --- |
| 1. Baseline distribution for all MECHANISMS schools, and NI control schools. | 1. Follow-up distribution for all MECHANISMS schools, and NI control schools. |
|  |  |
| (c) Distribution of changes between baseline and follow-up by intervention group. | (d) Distribution of changes between baseline and follow-up by setting. |

#

# Supplementary Tables

## Supplementary Table S1. Baseline pupil characteristics. Mean (SD) unless otherwise stated.

|  | **ASSIST schools (N=6)** | **Dead Cool schools (N=6)** | **Northern Ireland MECHANISMS schools (N=6)^a^** | **Bogotá MECHANISMS schools (N=6)^a^** | **Northern Ireland Control schools (N=10)^a^** | **Test for baseline differences (Intervention)** | | **Test for baseline differences (Setting)** | |
| --- | --- | --- | --- | --- | --- | --- | --- | --- | --- |
|  |  |  |  |  |  | **Three-group**  **(0=NI control; 1=ASSIST; 2=Dead Cool)^a,b^** | **Pairwise^a,c^** | **Three-group**  **(0=NI control; 1=NI; 2=****Bogotá)^a,b^** | **Pairwise^a,c^** |
| Setting, N | | | | | | | | | |
| *Northern Irish schools* | 3 | 3 | 3 | 3 | 10 | N/A. | N/A. | N/A. | N/A. |
| *Bogotá schools* | 3 | 3 | 3 | 3 | N/A. |  |  |  |  |
| No. of classes, N | 29 | 26 | 31 | 24 | 10 |  |  |  |  |
| *Northern Irish schools* | 18 | 13 | 31 | 0 | 10 |  |  |  |  |
| *Bogotá schools* | 11 | 13 | 0 | 24 | N/A. |  |  |  |  |
| No. of pupils, n | 796 | 648 | 718 | 726 | 235 |  |  |  |  |
| *Northern Irish schools* | 423 | 295 | 718 | 0 | 235 |  |  |  |  |
| *Bogotá schools* | 373 | 353 | 0 | 726 | N/A. |  |  |  |  |
| Participation, n (%) | 745 (93.6%) | 599 (92.4%) | 691 (96.2%) | 653 (89.9%) | 235 (100.0%) |  |  |  |  |
| *Northern Irish schools* | 405 (95.7%) | 286 (96.9%) | 691 (96.2%) | 0 (0.0%) | 235 (100.0%) |  |  |  |  |
| *Bogotá schools* | 340 (91.2%) | 313 (88.7%) | 0 (0.0%) | 653 (89.9%) |  |  |  |  |  |
| School NIMDM (1-890)^d^ | 401.3 (254.9) | 261.3 (166.0) | 343.8 (233.0) | N/A. | N/A. |  |  |  |  |
| School INSE (1-4)^e^ | 2.4 (0.5) | 2.7 (0.5) | N/A. | 2.5 (0.5) | N/A. |  |  |  |  |
| Individual NIMDM (1-890)^d^ | 400.2 (262.4) | 295.7 (193.4) | 356.7 (241.5) | N/A. | 367.5 (269.6) |  |  |  |  |
| Individual DANE SES (0-6)^f^ | 2.3 (0.8) | 2.5 (0.9) | N/A. | 2.4 (0.9) | N/A. |  |  |  |  |
| Individual socio-economic status, n (%)^g^ | | | | | | | | | |
| *1 (Low)* | 332 (44.6%) | 270 (45.1%) | 259 (37.5%) | 343 (52.5%) | 83 (35.3%) | **χ^2^=14.34, df=2, p=0.0008^j^** | NIC vs. A: p=0.10  **NIC vs. DC: p=0.0002^j^**  **A vs. DC: p=0.01** | **χ^2^=45.49, df=2, p=0.0001^j^** | NIC vs. NI: p=0.87  **NIC vs. Bog: p<0.0001^j^**  **NI vs. Bog: p<0.0001^j^** |
| *2 (Middle)* | 210 (28.2%) | 252 (42.1%) | 170 (24.6%) | 292 (44.7%) | 73 (31.1%) |  |  |  |  |
| *3 (High)* | 120 (16.1%) | 15 (2.5%) | 133 (19.2%) | 2 (0.3%) | 39 (16.6%) |  |  |  |  |
| Gender, n(%) | | | | | | | | | |
| *Boys* | 366 (49.1%) | 259 (43.2%) | 298 (43.1%) | 327 (50.1%) | 111 (47.2%) | **χ^2^=6.19, df=2, p=0.05** | NIC vs. A: p=0.19  NIC vs. DC: p=0.61  **A vs. DC: p=0.01** | χ^2^=1.80, df=2, p=0.41 | Not conducted. |
| *Girls* | 329 (44.2%) | 304 (50.8%) | 321 (46.5%) | 312 (47.8%) | 124 (52.8%) |  |  |  |  |
| *Prefer not to say* | 7 (0.9%) | 9 (1.5%) | 11 (1.6%) | 5 (0.8%) | N/A. |  |  |  |  |
| Age, n (%) | | | | | | | | | |
| *11 years old* | 3 (0.4%) | 4 (0.7%) | 1 (0.1%) | 6 (0.9%) | 0 (0.0%) | **χ^2^=29.73, df=2, p=0.0001^j^** | **NIC vs. A: p<0.0001^j^**  **NIC vs. DC: p<0.0001^j^**  A vs. DC: p=0.28 | **χ^2^=75.02, df=2, p=0.0001^j^** | **NIC vs. NI: p=0.004**  **NIC vs. Bog: p<0.0001^j^**  **NI vs. Bog: p<0.0001^j^** |
| *12 years old* | 259 (34.8%) | 201 (33.6%) | 243 (35.2%) | 217 (33.2%) | 116 (49.4%) |  |  |  |  |
| *13 years old* | 358 (48.1%) | 279 (46.6%) | 380 (55.0%) | 257 (39.4%) | 118 (50.2%) |  |  |  |  |
| *14 years old* | 66 (8.9%) | 48 (8.0%) | 6 (0.9%) | 108 (16.5%) | 1 (0.4%) |  |  |  |  |
| *15 or more years old* | 23 (3.1%) | 43 (7.2%) | 0 (0.0%) | 66 (10.1%) | 0 (0.0%) |  |  |  |  |
| Ethnicity, n (%)^h^ | | | | | | | | | |
| *White British* | 103 (13.8%) | 62 (10.4%) | 165 (23.9%) | N/A. | 53 (22.6%) | χ^2^=2.33, df=2, p=0.31 | Not conducted. | **χ^2^=14.39, df=2, p=0.0008^j^** | NIC vs. NI: p=0.98  **NIC vs. Bog: p=0.02**  **NI vs. Bog: p=0.0006^j^** |
| *White Irish* | 224 (30.1%) | 190 (31.7%) | 414 (59.9%) | N/A. | 161 (68.5%) |  |  |  |  |
| *Colombian: No ethnic minority* | 294 (39.5%) | 261 (43.6%) | N/A. | 555 (85.0%) | N/A. |  |  |  |  |
| *Ethnic minority* | 79 (10.6%) | 59 (9.8%) | 49 (7.1%) | 89 (13.6%) | 18 (7.7%) |  |  |  |  |
| Family | | | | | | | | | |
| *Lives with mother, n (%)* | 641 (86.0%) | 515 (86.0%) | 608 (88.0%) | 548 (83.9%) | 212 (90.2%) | **χ^2^=12.52, df=2, p=0.002^j^** | **NIC vs. A: p=0.002^j^**  **NIC vs. DC: p=0.0004^j^**  A vs. DC: p=0.34 | **χ^2^=70.00, df=2, p=0.0001^j^** | NIC vs. NI: p=0.51  **NIC vs. Bog: p<0.0001^j^**  **NI vs. Bog: p<0.0001^j^** |
| *Lives with father, n (%)* | 425 (57.0%) | 368 (61.4%) | 477 (69.0%) | 316 (48.4) | 164 (69.8%) | **χ^2^=15.92, df=2, p=0.0003^j^** | **NIC vs. A: p=0.0001^j^**  **NIC vs. DC: p=0.003**  A vs. DC: p=0.19 | **χ^2^=114.16, df=2, p=0.0001^j^** | NIC vs. NI: p=0.91  **NIC vs. Bog: p<0.0001^j^**  **NI vs. Bog: p<0.0001^j^** |
| *Number of family members in household^i^* | 2.5 (0.8) | 2.6 (0.8) | 2.6 (0.7) | 2.5 (0.9) | 2.5 (0.7) | *F*=2.24, df=2, p=0.11 | Not conducted. | ***F*=4.23, df=2, p=0.01** | NIC vs. NI: p=0.07  NIC vs. Bog: p=0.71  **NI vs. Bog: p=0.005** |

^a^N/A = Not applicable.

^b^Tests for baseline differences on pupil characteristics between three groups (Intervention [0=NI control; 1=Dead Cool; 2=ASSIST], or Setting [0=NI control; 1=NI; 2= Bogotá]) were conducted using Kruskal-Wallis tests with adjustment for ties for ordinal variables, and analyses of variance (ANOVAs) for continuous variables. Bold and underlined text indicates tests which were significant at the p<0.05 level. NIC = NI control; A = ASSIST; DC = Dead Cool; NI = Northern Ireland; Bog = Bogotá; χ^2^ = chi-square statistic for Kruskal-Wallis tests; df = degrees of freedom; *F* = F-statistic for ANOVAs.

^c^For pupil characteristics with significant differences between three groups, pairwise comparison tests were conducted. Pairwise comparison tests were conducted using Wilcoxon rank-sum (Mann-Whitney) tests with adjustment for ties for ordinal variables, and independent samples t-tests (two-sided) for continuous variables. Bold and underlined text indicates tests which were significant at the p<0.05 level. NIC = NI control; A = ASSIST; DC = Dead Cool; NI = Northern Ireland; Bog = Bogotá.

^d^Northern Ireland Multiple Deprivation Measure (NI only; 1=most deprived to 890=least deprived). Calculated based on ranking of NI postcodes for seven domains of deprivation including income; employment; health and disability; education, skills and training; access to services; living environment; crime and disorder. Provided by Northern Ireland Statistics and Research Agency (NISRA) (8).

^e^Socio-economic level index (Bogotá only; 1=Lower; 2=Middle-low; 3=Middle-high; 4=Higher). Calculated each year using a sample from each school, based on the characteristics of the home and its infrastructure, some household assets, the relationship of the children with their parents, among other characteristics. Schools are then classified into four levels according to the average of the responses of the pupils enrolled in them. Provided by the Instituto Colombiano para el Fomento de la Educación Superior (ICFES; “Colombian Institute for the Promotion of Higher Education”) (56).

^f^Socioeconomic level index of individual pupils (Bogotá only; 0=Informal settlement; 1=Lowest; 2=Low; 3=Middle-Low; 4=Middle; 5=Middle-High; 6=High), according to the Departamento Administrativo Nacional de Estadística (DANE; "National Administrative Department of Statistics") (57).

^g^Categorical outcome indicating individual pupils' socio-economic status (NI: 1=NIMDM2017≤296.6; 2=296.6<NIMDM2017≤593.2; 3=NIMDM2017>593.2; Bogotá: 1=Informal settlement/Lowest/Low; 2=Middle-Low/Middle; 3=Middle-High/High; see Supplementary Table S3 for details of variables).

^h^White Irish (NI only), White British (NI only); Colombian: No ethnic minority (Bogotá only). In Northern Ireland, the ethnic minority groups are "African", "Asian", "Chinese", "Any other ethnic group". The non-ethnic minority groups are "White British", "White Irish". In Bogotá, the ethnic minority groups are "Indigenous", "Gypsy/Roma", "Archipelago Raizal", "Palenquero of San Basilio", "Black/Mulatto/Afro-Colombian/Afro-descendant". The non-ethnic minority groups are "None of the above" (i.e., Colombian no ethnic minority). Tests for baseline differences are conducted with the binary variable (0="no ethnic minority"; 1="ethnic minority").

^i^Continuous outcome variable. Tests for baseline differences between three groups were conducted using ANOVAs. Pairwise comparisons were conducted using independent samples t-tests (two-sided). All other participant characteristics were treated as ordinal variables. See footnotes b-c.

^j^Retained statistical significance at the 5% level after using the Holm-Bonferroni procedure to correct the p-values for multiple testing (p≤0.05; based on all tests for baseline differences reported in Supplementary Table S1, i.e., 47 tests).

## Supplementary Table S2. Behavior change techniques included in the ASSIST and Dead Cool interventions and targeted mediators.

| **Target behavior** | **Behavior Change Technique** | **Example** | **Intervention activity** | **Targeted mediating constructs** |
| --- | --- | --- | --- | --- |
| **Dead Cool (Behavior change techniques included in the original and updated Dead Cool programs).^a^** | | | | |
| Smoking prevention. | Information about health consequences. | At the start of each lesson, pupils complete the “Quick Quiz Check” testing the knowledge they have picked up from the video. Lesson 2 asks “Which substance is nicotine more addictive than? Answer: Heroin” and “What effect does smoking have on your skin? Answer: Ages the skin and causes wrinkles”. | Lesson 2, “Activity 1 – Quick Quiz Check”. | Smoking intentions. Knowledge of smoking. Attitudes towards smoking. Perceived risks of tobacco use. |
|  | Information about social and environmental consequences. | “For children who live with parents or siblings who smoke, statistics reveal that they are up to three times more likely to become smokers themselves than children of non-smoking households” (Teachers Guide, page 21). During lesson 4, pupils learn about the dangers of second-hand smoke, and decide how they might negotiate approaches they might take to minimize their exposure to second-hand smoke at home. | Lesson 4, “Dead Easy: Parents, Family, Relationships”. | Smoking intentions. Attitudes towards smoking. Perceived risks of tobacco use, Perceived benefits of tobacco use. Perceived peer approval (injunctive norm). Perceived parental/family approval (injunctive norm). |
|  | Information about emotional consequences. | During lesson 1, pupils learn about the physical and emotional consequences of smoking. During activity 2, they are asked to respond (Agree/Disagree/Don’t know) to a series of statements about smoking, e.g., “I might regret starting to smoke because I might become addicted and spend a lot of money on cigarettes”. | Lesson 1, “Activity 2 – Statements About Smoking”. | Smoking intentions. Knowledge of smoking. Attitudes towards smoking. Perceived risks of tobacco use, Perceived benefits of tobacco use. Self-efficacy to quit tobacco/remain tobacco-free. Perceived behavioral control. |
|  | Salience of consequences. | Pupils watch DVD clips at the start of each lesson during which they see children their age talking about smoking. The negative health consequences are often emphasized. “I don’t want black lungs” (Lesson 2), “…seeing the advertisements about it and how harmful it is to yourself and how harmful it can be in the long run” (Lesson 4), “Every year 1000 families in Northern Ireland lose a loved one due to lung cancer. 85% caused by smoking” (Lesson 4). The “Teachers’ Fact Files” resources includes images of cigarette packets showing graphic images of the health consequences. | Lesson 2 and Lesson 4, DVD clips. Teachers’ Fact Files. | Smoking intentions. Knowledge of smoking. Attitudes towards smoking. Perceived risks of tobacco use. Self-efficacy to quit tobacco/remain tobacco-free. Perceived behavioral control. Exposure to advertising in the media. Exposure to advertising in shops. |
|  | Anticipated regret. | During the “Statements About Smoking” activity, pupils are prompted to think about the reasons why someone might regret starting to smoke (e.g., friends not wanting to be around them, odour, poor health, waste of money, causing damage to others via second-hand smoke). | Lesson 1, “Activity 2 – Statements About Smoking”. | Smoking intentions. Knowledge of smoking. Attitudes towards smoking. Perceived risks of tobacco use, Perceived benefits of tobacco use. Self-efficacy to quit tobacco/remain tobacco-free. Perceived behavioral control. |
|  | Information about antecedents. | Throughout the Dead Cool program, there is ongoing focus on the influences on smoking that adolescents experience from friends, family, and in the media/social media. E.g., In lesson 1, pupils practice using persuasive language to debunk some myths they might hear about smoking from their friends. In lesson 4, they practice negotiating with adult smokers in their household to enable them to avoid second-hand smoke. During lesson 5, they view images featuring celebrities smoking, and discuss how tobacco companies have used product placements (e.g., in feature films with famous celebrities) to attempt to manipulate people into buying their products. | Lesson 1, “Activity 4 – Persuasive Argument”. Lesson 4, “Activity 3 – Negotiating and Compromising”. Lesson 5, “Activity 3 – “Spot the Product” and “Activity 4 – Caught Out”. | Smoking intentions. Attitudes towards smoking. Perceived risks of tobacco use, Perceived benefits of tobacco use. Perceived peer approval (injunctive norm). Perceived parental/family approval (injunctive norm). Perceived prevalence of peer smoking (descriptive norm). Self-efficacy to quit tobacco/remain tobacco-free. Perceived behavioral control. Exposure to advertising in the media. Exposure to advertising in shops. |
|  | Pros and cons. | In lesson 1, pupils think about whether they agree with some potential outcomes of smoking (e.g., getting cancer, making parents angry or whether parents who are smokers themselves wouldn’t mind, fitting in socially because “most people do it”) during the “Statements About Smoking” activity. Pupils also practice using persuasive language to debunk some myths they might hear about smoking from their friends. E.g., “If you don’t do it, you will be left behind”. | Lesson 1, “Activity 2 – Statements About Smoking”, “Activity 3 – Myths about Smoking”, and “Activity 4 – Persuasive Argument”. | Smoking intentions. Knowledge of smoking. Attitudes towards smoking. Perceived risks of tobacco use, Perceived benefits of tobacco use. Perceived peer approval (injunctive norm). Perceived parental/family approval (injunctive norm). Perceived prevalence of peer smoking (descriptive norm). |
|  | Demonstration of the behavior. | At the start of lesson 2, pupils watch a DVD clip of a role-play scene during which they see a teenage boy resisting peer pressure from his friends to smoke. | Lesson 2, DVD clip. | Smoking intentions. Attitudes towards smoking. Self-efficacy to quit tobacco/remain tobacco-free. Perceived behavioral control. |
|  | Problem solving. | During lesson 2, pupils discuss some refusal tactics they might use if a friend was pressuring them to smoke (e.g., “Just say no”). They are asked to consider how they might use it, what are the pros and cons of using the tactic, and why the tactic might work for some pupils but not others. At the end of the activity each pupil is asked to decide which tactic would work best for them personally. | Lesson 2, “Activity 2 – Refusal Tactics”. | Smoking intentions. Attitudes towards smoking. Self-efficacy to quit tobacco/remain tobacco-free. Perceived behavioral control. |
|  | Behavioral practice/rehearsal. | During activity 3 of lesson 2, pupils are divided into groups of three and asked to role-play a situation in which two of them are attempting to bully the third into smoking a cigarette. Pupil three is asked to practice a range of refusal tactics (learned during the previous activity in the lesson) to decide which of them they are most comfortable using if resisting pressure to smoke cigarettes. | Lesson 2, “Activity 3 – Role-Play”. | Smoking intentions. Attitudes towards smoking. Self-efficacy to quit tobacco/remain tobacco-free. Perceived behavioral control. |
|  | Social comparison. | Pupils are provided with accurate information on the percentage of adults and teenagers who smoke. E.g., After discussing the statement “Most adults and most teenagers in Northern Ireland smoke”, Northern Irish pupils were told that only 20% of adults aged 16 and over, and only 4% of young people (11-16 years) are smokers in Northern Ireland. | Lesson 1, “Activity 2 – Statements About Smoking”. | Smoking intentions. Perceived prevalence of peer smoking (descriptive norm). Self-efficacy to quit tobacco/remain tobacco-free. Perceived behavioral control. |
|  | Information about others' approval. | During lesson 1, pupils discuss responses, using accurate smoking prevalence statistics, to debunk some “myths” about smoking. These myths mostly revolve around the idea that most other teenagers are smoking or will “judge you” for not smoking. | Lesson 1, “Activity 3 – Myths about Smoking” and “Activity 4 – Persuasive Argument”. | Smoking intentions. Attitudes towards smoking. Perceived peer approval (injunctive norm). Perceived parental/family approval (injunctive norm). |
|  | Identification of self as role model. | Pupils are asked to identify themselves as a “No Smoking Hero”, and to encourage others to do the same. During lesson 5, they sign a “Self-Promise Contract” to remain smoke-free, agreeing that they will encourage others to do the same. | Lesson 1, “Homework”. Lesson 3, “Activity 5 – Self-Promise Contract”. | Smoking intentions. Self-efficacy to quit tobacco/remain tobacco-free. Perceived behavioral control. Perceived peer approval (injunctive norm). |
|  | Social support. | When signing the “Self-Promise Contract” during lesson 5, pupils nominate “Support Friends” to help them to achieve their non-smoking goals. | Lesson 3, “Activity 5 – Self-Promise Contract”. | Smoking intentions. Self-efficacy to quit tobacco/remain tobacco-free. Perceived behavioral control. Perceived peer approval (injunctive norm). |
|  | Credible source. | Throughout the Teachers’ Fact Files resource, information on the health consequences and prevalence of smoking is provided from individuals and organizations who would be considered experts in the field. E.g., The number of cases of lung cancer reported by the Northern Ireland Cancer Registry, the prevalence of smoking during pregnancy in Northern Ireland as reported by the Public Health Agency NI, and the consequences of passive smoking for children as reported by the Royal College of Physicians. | Teachers’ Fact Files. | Smoking intentions. Knowledge of smoking. Attitudes towards smoking. Perceived risks of tobacco use. Self-efficacy to quit tobacco/remain tobacco-free. Perceived behavioral control. |
| **Dead Cool (Behavior change techniques included in the updated Dead Cool program only).^a^** | | | | |
| Smoking prevention. | Goal setting (behavior). | During lesson 3, pupils sign a “Self-Promise Contract” agreeing to remain smoke-free and to encourage others to do the same, nominate support friends to sign the contract with them, and make plans to reward themselves for achieving their goals after one, two, three, and twenty years. | Lesson 3, “Activity 5 – Self-Promise Contract”. | Smoking intentions. Self-efficacy to quit tobacco/remain tobacco-free. Perceived behavioral control. Perceived peer approval (injunctive norm). |
|  | Behavioral contract. | During lesson 3, pupils sign a “Self-Promise Contract” agreeing to remain smoke-free. | Lesson 3, “Activity 5 – Self-Promise Contract”. | Smoking intentions. Self-efficacy to quit tobacco/remain tobacco-free. Perceived behavioral control. |
|  | Self-incentive. | During activity 5 of lesson 3, pupils make plans to reward themselves for achieving their goals to remain smoke-free after one, two, three, and twenty years. | Lesson 3, “Activity 5 – Self-Promise Contract”. | Smoking intentions. Self-efficacy to quit tobacco/remain tobacco-free. Perceived behavioral control. |
|  | Feedback on behavior. | During activity 5 of lesson 3, pupils identify friends/family members to support them in achieving their non-smoking goals, and to let them know if they think they are at risk of becoming a smoker. | Lesson 3, “Activity 5 – Self-Promise Contract”. | Smoking intentions. Self-efficacy to quit tobacco/remain tobacco-free. Perceived peer approval (injunctive norm). |
|  | Verbal persuasion about capability. | During activity 4 of lesson 1, pupils practice using persuasive arguments in pairs to persuade their partners that the myths they have heard about smoking are not true. | Lesson 1, “Activity 4 – Persuasive Argument”. | Smoking intentions. Attitudes towards smoking. Self-efficacy to quit tobacco/remain tobacco-free. Perceived behavioral control. Perceived peer approval (injunctive norm). |
|  | Instruction on how to perform the behavior. | During activity 2 of lesson 2, pupils are presented with various ways of refusing cigarettes, discuss the pros and cons of each approach, and decide which approach would work best for them. | Lesson 2, “Activity 2 – Refusal Tactics”. | Smoking intentions. Self-efficacy to quit tobacco/remain tobacco-free. Perceived behavioral control. |
|  | Self-talk. | During activity 3 of lesson 3, pupils are prompted to use self-talk to overcome negative inner thoughts about smoking into positive self-talk to help them resist the temptation to smoke. | Lesson 3, “Activity 3 – Self-Talk”. | Smoking intentions. Attitudes towards smoking. Self-efficacy to quit tobacco/remain tobacco-free. Perceived behavioral control. |
|  | Comparative imagining of future outcomes. | During activity 4 of lesson 3, pupils are asked to visualize themselves achieving their non-smoking goals in the future. | Lesson 3, “Activity 4 – Visualization”. | Smoking intentions. Attitudes towards smoking. Self-efficacy to quit tobacco/remain tobacco-free. Perceived behavioral control. Perceived peer approval (injunctive norm). Perceived parental/family approval (injunctive norm). |
|  | Mental rehearsal of successful performance. | During activity 4 of lesson 3, pupils are asked to visualize themselves achieving their non-smoking goals in the future. | Lesson 3, “Activity 4 – Visualization”. | Smoking intentions. Attitudes towards smoking. Self-efficacy to quit tobacco/remain tobacco-free. Perceived behavioral control. |
|  | Imaginary reward. | During activity 4 of lesson 3, pupils are asked to visualize positive outcomes from achieving their non-smoking goals in future (e.g., feeling happy, making your friends/family/teachers proud). | Lesson 3, “Activity 3 – Self-Talk” and “Activity 4 – Visualization”. | Smoking intentions. Attitudes towards smoking. Self-efficacy to quit tobacco/remain tobacco-free. Perceived behavioral control. Perceived peer approval (injunctive norm). Perceived parental/family approval (injunctive norm). |
|  | Reduce negative emotions/increase positive emotions. | During activity 3 of lesson 3, pupils are prompted to use self-talk to overcome negative inner thoughts about smoking into positive self-talk to help them resist the temptation to smoke. During activity 4 of lesson 3, pupils are asked to visualize positive outcomes from achieving their non-smoking goals in future (e.g., feeling happy, making your friends/family/teachers proud). | Lesson 3, “Activity 3 – Self-Talk” and “Activity 4 – Visualization”. | Smoking intentions. Attitudes towards smoking. Self-efficacy to quit tobacco/remain tobacco-free. Perceived behavioral control. Perceived peer approval (injunctive norm). Perceived parental/family approval (injunctive norm). |
|  | Restructuring the social environment. | During activity 4 of lesson 2, pupils discuss ways they can remain friends with smokers whilst staying smoke-free (e.g., avoiding social situations which are likely to lead to smoking). | Lesson 2, “Activity 4 – Choosing and Keeping Friends”. | Smoking intentions. Attitudes towards smoking. Self-efficacy to quit tobacco/remain tobacco-free. Perceived behavioral control. |
|  | Avoidance/reducing exposure to cues for the behavior. | During activity 4 of lesson 2, pupils discuss ways they can remain friends with smokers whilst staying smoke-free (e.g., avoiding social situations which are likely to lead to smoking). | Lesson 2, “Activity 4 – Choosing and Keeping Friends”. | Smoking intentions. Attitudes towards smoking. Self-efficacy to quit tobacco/remain tobacco-free. Perceived behavioral control. |
|  | Social reward. | During lesson 8, pupils are encouraged to congratulate their classmates for being a non-smoker. | Lesson 8, “Activity 2a – Group Affirmation”. | Smoking intentions. Attitudes towards smoking. Self-efficacy to quit tobacco/remain tobacco-free. Perceived peer approval (injunctive norm). |
|  | Non-specific reward. | Pupils are presented with certificates for completing the Dead Cool program. | Lesson 8, “Activity 2b – Certificates”. | Smoking intentions. Self-efficacy to quit tobacco/remain tobacco-free. |
|  | Rewarding completion. | Pupils are presented with certificates for completing the Dead Cool program. | Lesson 8, “Activity 2b – Certificates”. | Smoking intentions. Self-efficacy to quit tobacco/remain tobacco-free. |
| **ASSIST** | | | | |
| Smoking prevention. | Information about health consequences. | Peer supporters learn about the ingredients of a cigarette and discuss their poisonous nature. | Training day 1, “Ready Steady Cook”. | Smoking intentions. Knowledge of smoking. Attitudes towards smoking. Perceived risks of tobacco use. |
|  | Information about social and environmental consequences. | Peer supporters take part in a group activity in which they are asked to discuss and list the social and environmental consequences of smoking from prior knowledge. | Training day 1, “What Do We Know About Smoking?” | Smoking intentions. Attitudes towards smoking. Perceived risks of tobacco use, Perceived benefits of tobacco use. Perceived peer approval (injunctive norm). |
|  | Information about emotional consequences. | Peer supporters are asked to consider whether it is true that being smoke-free helps to relieve anxiety, stress, and depression. | Training day 1, “True/False Statements”. | Smoking intentions. Knowledge of smoking. Attitudes towards smoking. Perceived risks of tobacco use, Perceived benefits of tobacco use. Self-efficacy to quit tobacco/remain tobacco-free. Perceived behavioral control. |
|  | Information about antecedents. | Peer supporters are asked to consider some of the reasons why people choose to smoke. | Training day 1, “Why Do People Smoke?” | Smoking intentions. Knowledge of smoking. Attitudes towards smoking. Perceived risks of tobacco use, Perceived benefits of tobacco use. Perceived prevalence of peer smoking (descriptive norm). Perceived peer approval (injunctive norm). |
|  | Pros and cons. | During a group activity, Peer supporters are asked to list the positives and negatives of: (1) smoking; (2) being smoke-free. | Training day 1, “What Do We Know About Smoking?” | Smoking intentions. Knowledge of smoking. Attitudes towards smoking. Perceived risks of tobacco use, Perceived benefits of tobacco use. Perceived prevalence of peer smoking (descriptive norm). Perceived peer approval (injunctive norm). Self-efficacy to quit tobacco/remain tobacco-free. Perceived behavioral control. |
|  | Identification of self as role model. | Peer supporters are reminded that they have been nominated by others in their year group because they are trusted and respected. Therefore, it is important for them to pass on accurate facts. | Training day 1, “True/False Statements”. | Knowledge of smoking. Perceived peer approval (injunctive norm). Self-efficacy to quit tobacco/remain tobacco-free. |
|  | Social comparison. | Peer supporters are provided with accurate statistics about the proportion of people who smoke, and who start smoking before the age of 18. | Training day 1, “True/False Statements”. | Smoking intentions. Perceived prevalence of peer smoking (descriptive norm). Self-efficacy to quit tobacco/remain tobacco-free. Perceived behavioral control. |
|  | Adding objects to the environment. | Peer supporters are invited to select anti-smoking posters to display in their school. | Training day 1, “Jigsaws”. | Smoking intentions. Knowledge of smoking. Attitudes towards smoking. Perceived risks of tobacco use. |
| Being an effective peer supporter. | Identification of self as role model. | Peer supporters are asked to think about the ways in which they can influence their peers to make healthy informed choices. | Training day 2, “Influences on Attitudes and Values”. | Skills-building (to increase peer supporters’ confidence and enable them to have effective conversations about smoking with peers in their school year group). |
|  | Valued self-identity. | Peer supporters are told that they will take part in an activity in which they will explore “the things you yourself are good at, interested in, and proud of”. | Training day 1, “Personal Shield”. | Skills-building. |
|  | Verbal persuasion about capability. | Peer supporters take part in an activity in which they review the strengths and characteristics of a peer supporter. The trainers highlight that they have already seen lots of evidence that the peer supporters display these strengths and characteristics and give some examples. They also highlight that these strengths will help them deal with any tricky situations that may arise. | Follow-up 2, “Strengths and Characteristics of a Peer Supporter”. | Skills-building. |
|  | Focus on past success. | Peer supporters are asked to recap on issues that arose during practice conversations, and how they overcame challenging responses or situations. | Training day 2, “Problem Solving”. | Skills-building. |
|  | Demonstration of the behavior. | Peer supporters watch trainers demonstrate an example conversation. | Training day 2, “Practice Conversations”. | Skills-building. |
|  | Goal setting (behavior). | At the end of the training course, peer supporters are asked to have informal conversations with their peers about smoking using the facts that they have learned, to record these conversations in a diary, and to attend four follow-up sessions. | Training day 2, “What Happens Next?” | Skills-building. |
|  | Action planning. | Peer supporters are asked to plan how, when, and where they might use the fact about smoking they have learned in a conversation. | Training day 2, “How, When and Where?” | Skills-building. |
|  | Prompts/cues. | Peer supporters are asked to think about how they could use health promotion resources, like posters, as prompts for conversations. They are invited to select anti-smoking posters to display in their school. | Training day 1, “Jigsaws”. | Skills-building. |
|  | Behavioral practice/rehearsal. | Peer supporters are asked to script a conversation in small groups, and to act it out. | Training day 2, “Practice Conversations”. | Skills-building. |
|  | Feedback on behavior. | Trainers explain that the purpose of the diaries is so that they can see how the Peer supporters’ conversations are going, and offer feedback, help, and support throughout the follow-up sessions. | Training day 2, “Diaries”. Feedback is provided ongoing throughout the four follow-up sessions. | Skills-building. |
|  | Monitoring of emotional consequences. | Peer supporters are asked to review how they feel about the role, looking at what they have written in their diaries, and identifying any challenges that have arisen. | Follow-up 1, “How Do You Feel?” | Skills-building. |
|  | Problem solving. | In groups, peer supporters are asked to think of a concern they have about the role, to talk it through, and see if they can come up with a solution. | Training day 2, “Problem Solving”. | Skills-building. |
|  | Social support (unspecified). | Throughout the follow-up sessions, peer supporters are reminded that they can talk to the trainers in confidence about the role, or speak to the contact teacher at their school. At each session they are invited to use sticky notes to write individual concerns. | Ongoing throughout the follow-up sessions. | Skills-building. |
|  | Social support (practical). | Peer supporters are asked to remember one fact about smoking to use in conversations. They are encouraged to view the other peer supporters as a resource and learning community to support each other. If they forget their fact, they are encouraged to ask the other peer supporters for help. | Training day 1, “Information Islands”. | Skills-building. |
|  | Social comparison. | Peer supporters are asked to script practice conversations in small groups and to act them out. The rest of the group is invited to give feedback. | Training day 2, “Practice Conversations”. | Skills-building. |
|  | Social reward. | At the end of the last follow-up session, peer supporters are allowed to choose a favorite game from the program as a reward for their hard work. | Follow-up 4, “Thank you, Presentation, and Close”. | Skills-building. |
|  | Non-specific incentive. | Peer supporters are told that they will receive a certificate in recognition of their hard work at the end of the program if they take on the role of peer supporter, and attend the training course and four follow-up sessions. | Peer supporter Recruitment Meeting, “What Will the Trainers Do?” | Skills-building. |
|  | Self-monitoring of behavior. | Peer supporters are asked how they feel about the role, reflecting on their experiences of conversations so far, and referring to their diaries. | Follow-up 1, “First Experience Reflection Activity”. | Skills-building. |
|  | Non-specific reward. | Peer supporters are thanked for their participation, and presented with certificates. | Follow-up 4, “Thank you, Presentation, and Close”. | Skills-building. |
|  | Rewarding completion. | Peer supporters are thanked for their participation, and presented with certificates. | Follow-up 4, “Thank you, Presentation, and Close”. | Skills-building. |

^a^Examples are provided from the updated Dead Cool program.

## Supplementary Table S3. Measurement instruments and coding of outcome variables.

| **Variable name** | **Scenario/Question** | **Responses/Coding** |
| --- | --- | --- |
| **Experiment Part 2: Injunctive smoking/vaping norms (α=0.78).^a^** | | |
| Part 2 Situation 2 (P2S2) | Parent smoking in their own home in front of children under age of 5. | -1=Extremely socially inappropriate; -0.6=Very socially inappropriate; -0.2=Somewhat socially inappropriate; +0.2=Somewhat socially appropriate; +0.6=Very socially appropriate; +1=Extremely socially appropriate. |
| Part 2 Situation 3 (P2S3) | An adult smoking in a car with children under the age of 16 in the car. | *As per P2S2.* |
| Part 2 Situation 4 (P2S4) | Someone selling cigarettes to a teenager who looks younger than 16 without requesting proof of age. | *As per P2S2.* |
| Part 2 Situation 5 (P2S5) | In a recent superhero movie the lead actor is seen smoking in the opening scene. | *As per P2S2.* |
| Part 2 Situation 6 (P2S6) | An older student from school is smoking outside school, for example, at a bus stop. | *As per P2S2.* |
| Part 2 Situation 7 (P2S7) | A pupil from school is using an e-cigarette while walking to school. | *As per P2S2.* |
| Part 2 Situation 8 (P2S8) | A pupil from school shares a photograph of him/herself using an e-cigarette on social media. | *As per P2S2.* |
| Part 2 Situation 9 (P2S9) | A pupil from school is chewing tobacco. | *As per P2S2.* |
| Experiment Part 2 | Experimentally measured injunctive smoking/vaping norms. | Average of items P2S2 to P2S9. |
| Experiment Part 2 non-vaping items | Experimentally measured injunctive smoking norms. | Average of items P2S2 to P2S6 and P2S9. |
| Experiment Part 2 vaping items | Experimentally measured injunctive vaping norms. | Average of items P2S7 to P2S8. |
| **Experiment Part 3: Descriptive smoking/vaping norms (α=0.85).^a^** | | |
| Part 3 Question 1 (P3Q1) | The proportion of my peers who would be accepting of a close friend smoking. | -1=None of my peers; -0.6=Only a few of my peers; -0.2=Some of my peers; +0.2=A lot of my peers; +0.6=Most of my peers; +1=All of my peers. |
| Part 3 Question 2 (P3Q2) | The proportion of my peers who would be accepting of a close friend vaping. | *As per P3Q1.* |
| Experiment Part 3 | Experimentally measured descriptive smoking/vaping norms. | Average of items P3Q1 to P3Q2. |
| **Experiment Part 4: Willingness to pay to support anti-smoking norms.** | | |
| Number of tokens donated to ASSIST/Dead Cool (Donation to ASSIST/Dead Cool) | You will be given 10 virtual tokens. Each token is worth 50 pence. That means you will receive tokens worth £5. You will then have the opportunity to give any amount of your £5 to the ASSIST/Dead Cool Program… The value of any tokens you do not give to ASSIST/Dead Cool will be your earnings for this Part… How many tokens do you want to give to ASSIST/Dead Cool? | 0 (0 tokens/£0.00 donated to ASSIST/Dead Cool) to 10 (10 tokens/£5.00 donated to ASSIST/Dead Cool). |
| **Survey: Self-report injunctive smoking norms (α=0.75).^b,c^** | | |
| Injunctive Norms 1 (IN1) | Most of the people who are important to me think that I… | -2=Definitely should smoke; -1=Maybe should smoke; 0=Don't know/neutral; +1=Maybe should not smoke; +2=Definitely should not smoke. |
| Injunctive Norms 2 (IN2) | My mother thinks that I… | *As per IN1. Responses of “I don’t have a mother” were also set to 0.* |
| Injunctive Norms 3 (IN3) | My father thinks that I… | *As per IN1. Responses of “I don’t have a father” were also set to 0.* |
| Injunctive Norms 4 (IN4) | My brother(s) think(s) that I… | *As per IN1. Responses of “I don’t have a brother” were also set to 0.* |
| Injunctive Norms 5 (IN5) | My sister(s) think(s) that I… | *As per IN1. Responses of “I don’t have a sister” were also set to 0.* |
| Injunctive Norms 6 (IN6) | My friends think that I… | *As per IN1. Responses of “I don’t have a friend” were also set to 0.* |
| Injunctive Norms 7 (IN7) | My best friend thinks that I… | *As per IN1. Responses of “I don’t have a best friend” were also set to 0.* |
| Injunctive Norms | Self-report injunctive smoking norms. | Average of items IN1 to IN7. |
| **Survey: Self-report descriptive smoking norms 1 (α=0.54).^b,d^** | | |
| Descriptive Norms 1.1 (DN1.1) | Does your best friend smoke? | 1=Very often; 2=Often; 3=Occasionally; 4=Rarely; 5=Never/Don't know. *Responses of “I don’t have a best friend” were also set to 5.* |
| Descriptive Norms 1.2 (DN1.2) | Does your mother smoke? | *As per DN1.1. Responses of “I don’t have a mother” were also set to 5.* |
| Descriptive Norms 1.3 (DN1.3) | Does your father smoke? | *As per DN1.1. Responses of “I don’t have a father” were also set to 5.* |
| Descriptive Norms 1.4 (DN1.4) | Do any of your brothers smoke? | *As per DN1.1. Responses of “I don’t have a brother” were also set to 5.* |
| Descriptive Norms 1.5 (DN1.5) | Do any of your sisters smoke? | *As per DN1.1. Responses of “I don’t have a sister” were also set to 5.* |
| Descriptive Norms 1 | Self-report descriptive smoking norms scale 1. | Average of items DN1.1 to DN1.5. |
| **Survey: Self-report descriptive smoking norms 2 (α=0.53).^b,d^** | | |
| Descriptive Norms 2.1 (DN2.1) | How many of your friends smoke? | 1=Almost all of them; 2=Many of them; 3=Half of them; 4=A few of them; 5=Almost none of them/Don't know. *Responses of “I don’t have a best friend” were also set to 5.* |
| Descriptive Norms 2.2 (DN2.2) | How many of your other family members smoke? | *As per DN2.1. Responses of “I don’t have other family members” were also set to 5.* |
| Descriptive Norms 2.3 (DN2.3) | How many of your classmates smoke? | *As per DN2.1. Responses of “I don’t have classmates” were also set to 5.* |
| Descriptive Norms 2 | Self-report descriptive smoking norms scale 2. | Average of items DN2.1 to DN2.3. |
| **Survey: Self-report smoking behavior (α=0.83).^b^** | | |
| Current smoking behavior | Do you smoke cigarettes at all nowadays? | 1=Does currently smoke; 2=Does not currently smoke. |
| Smoking behavior | Now read the following statements carefully and tick the box next to the one which best describes you. (I have never smoked; I have only ever tried smoking once; I used to smoke sometimes but I never smoke a cigarette now; I sometimes smoke cigarettes now but I don’t smoke as many as one a week). | 1=Sometimes smoke; 2=Previous smoker; 3=Smoked once; 4=Never smoked. |
| Past/current smoking behavior | Just to check, read the statements below carefully and tick the box next to the one which best describes you. (I have never tried smoking a cigarette, not even a puff or two; I did once have a puff or two of a cigarette, but I never smoke now; I do sometimes smoke cigarettes). | 1=Sometimes smoke; 2=Tried smoking; 3=Never smoked. |
| **Survey: Self-report smoking intentions (α=0.77).^b^** | | |
| Intentions (to quit smoking) | If you DO currently smoke, do you intend to quit smoking in the next six months? | 1=Definitely remain a smoker; 2=Probably remain; 3=Don't know; 4=Probably quit; 5=Definitely quit; 6=I don't smoke. |
| Intentions (to try smoking) | Do you think you will try a cigarette soon? | 1=Yes; 2=Don't know; 3=No. |
| Intentions (friends) | If one of your best friends were to offer you a cigarette, would you smoke it? | 1=Definitely yes; 2=Probably yes; 3=Not sure; 4=Probably not; 5=Definitely not. |
| Intentions | If you DON’T currently smoke, do you intend to take up smoking in the next 6 months?  Northern Ireland control group: Do you think you will smoke a cigarette at any time in the next year? | 1=I am a smoker; 2=Definitely start smoking; 3=Probably start smoking; 4=Don't know; 5=Probably remain; 6=Definitely remain a non-smoker.  Northern Ireland control group: 2=Definitely yes; 3=Probably yes; 4=Don't know; 5=Probably not; 6=Definitely not. |
| **Survey: Self-report susceptibility towards smoking.^b^** | | |
| Smoking susceptibility | Susceptibility to commencing smoking: Calculated from 3 items: 1. Intentions (to try smoking); 2. Intentions (friends); 3. Intentions. | 0=Not susceptible; 1=Susceptible. Coded 0 if participant responses to the 3 items are: 1. No; 2. Definitely not; 3. Definitely remain a non-smoker/Definitely not. Coded 1 otherwise. |
| **Survey: Self-report smoking knowledge and attitudes.^b^** | | |
| Knowledge | Knowledge of smoking. | Count of number of correct answers to 6 questions assessing knowledge of the effects of smoking: 0 (0 correct) to 6 (6 correct). |
| Attitudes | Attitudes towards smoking. | Average of 12 items (AT1-AT12), each coded 1 to 5: 1 (least anti-smoking attitudes) to 5 (greatest anti-smoking attitudes), α=0.81. |
| **Survey: Psychosocial constructs and mediators.^b^** | | |
| Self-efficacy: Emotional | Self-efficacy (Emotional subscale). | Average of 9 items (SEE1-SEE9), each coded 1 to 6: 1 (lowest self-efficacy to resist smoking) to 6 (greatest self-efficacy to resist smoking), α=0.97. |
| Self-efficacy: Friends | Self-efficacy (Friends subscale). | Average of 9 items (SEF1-SEF9), each coded 1 to 6: 1 (lowest self-efficacy to resist smoking) to 6 (greatest self-efficacy to resist smoking), α=0.96. |
| Self-efficacy: Opportunity | Self-efficacy (Opportunity subscale). | Average of 11 items (SEO1-SEO11), each coded 1 to 6: 1 (lowest self-efficacy to resist smoking) to 6 (greatest self-efficacy to resist smoking), α=0.98. |
| Perceived physical risks | Perceived risks of tobacco-use (Physical subscale). | Average of 7 items (RP1-RP7), each coded 0% to 100%: 0% (lowest perceived risk) to 100% (highest perceived risk), α=0.87. |
| Perceived social risks | Perceived risks of tobacco-use (Social subscale). | Average of 3 items (RS1-RS3), each coded 0% to 100%: 0% (lowest perceived risk) to 100% (highest perceived risk), α=0.71. |
| Perceived addiction risks | Perceived risks of tobacco-use (Addiction subscale). | Average of 3 items (RA1-RA3), each coded 0% to 100%: 0% (lowest perceived risk) to 100% (highest perceived risk), α=0.49. |
| Perceived benefits | Perceived benefits of tobacco-use. | Average of 5 items (BE1-BE5), each coded 0% to 100%: 0% (lowest perceived benefit) to 100% (highest perceived benefit), α=0.79. |
| Perceived behavioral control: easy to quit smoking | If I smoked regularly, I'm sure that it would be easy for me to quit. | 1=Strongly disagree; 2=Disagree; 3=Unsure; 4=Agree; 5=Strongly agree. |
| Perceived behavioral control: to avoid smoking | If I decided not to smoke, I am sure I could avoid smoking. | 1=Strongly disagree; 2=Disagree; 3=Unsure; 4=Agree; 5=Strongly agree. |
| Exposure to advertising in the media | Have you seen any advertisements for tobacco… (1) On television?; (2) In movies?; (3) In cinema halls?; (4) In newspapers, magazines or other print media?; (5) On hoardings, posters or walls?; (6) At sports events?; (7) At cultural events?; (8) In computer games? | Count number of media locations where tobacco advertisements have been viewed: 0 (0 of the possible locations) to 8 (8 of the possible locations). |
| Exposure to advertising in shops | In the past year, have you seen cigarette packets on display in any of the shops listed below?... (1) A supermarket?; (2) A newsagent, tobacconist or a sweet shop?; (3) A petrol station or garage shop?; (4) Some other type of shop? | Count number of different types of shop where tobacco advertisements have been viewed: 0 (0 of the possible shop types) to 4 (4 of the possible shop types). |
| **Smokerlyzer readings: Objectively measured smoking behavior.** | | |
| Carbon monoxide reading | Objectively measured smoking behavior over the past 24 hours captured using hand-held carbon monoxide monitors (PICOAdvantage Smokerlyzer, Bedfont) to measure expelled air carbon monoxide in parts per million (ppm) in a range of 0–150 ppm with an accuracy of 2ppm/5% (whichever is greater). | Continuous variable (ppm; 0 to 30). |
| **Survey: Self-report socio-demographic variables.** | | |
| Intervention | Intervention program delivered in participant’s school. | 0=Dead Cool; 1=ASSIST. |
| Intervention (ASSIST non-peer supporters and peer supporters analyzed separately). | Intervention program delivered in participant’s school. | 0=Dead Cool; 1=ASSIST non-peer supporters; 2=ASSIST peer supporters.  Entered as predictors in structural equation models with two dummy variables. |
| Setting | Participant setting. | 0=Northern Ireland; 1=Bogotá. |
| Setting/Intervention | Variable jointly indicating the intervention program delivered in participant's school, and participant's setting. Used for models including Northern Ireland control group data from the Dead Cool study. | 0=Northern Ireland control; 1=Northern Ireland Dead Cool; 2=Northern Ireland ASSIST; 3=Bogotá Dead Cool; 4=Bogotá ASSIST.  Entered as predictors in structural equation models with four dummy variables. |
| Setting/Intervention (ASSIST non-peer supporters and peer supporters analyzed separately). | Variable jointly indicating the intervention program delivered in participant's school, and participant's setting. Used for models including Northern Ireland control group data from the Dead Cool study. | 0=Northern Ireland control; 1=Northern Ireland Dead Cool; 2=Northern Ireland ASSIST non-peer supporters; 3=Northern Ireland ASSIST peer supporters; 4=Bogotá Dead Cool; 5=Bogotá ASSIST non-peer supporters; 6=Bogotá ASSIST peer supporters.  Entered as predictors in structural equation models with six dummy variables. |
| Gender | Participant gender. | 0=Boy; 1=Girl/Prefer not to say. |
| Age | Participant age. | 1=12 years or less; 2=13 years; 3=14 years or more.  Entered as covariates in structural equation models with two dummy variables. |
| Ethnicity | Participant ethnicity. | 0=No ethnic minority; 1=ethnic minority.  NI: The ethnic minority groups are "African", "Asian", "Chinese", "Any other ethnic group". The non-ethnic minority groups are "White British", "White Irish".  Bogotá: The ethnic minority groups are "Indigenous", "Gypsy/Roma", "Archipelago Raizal", "Palenquero of San Basilio", "Black/Mulatto/Afro-Colombian/Afro-descendant". The non-ethnic minority groups are "None of the above". |
| Socio-economic status^e^ | Participant socio-economic status. | NI: 1=NIMDM2017≤296.6; 2=296.6<NIMDM2017≤593.2; 3=NIMDM2017>593.2.  Bogotá: 1=Informal settlement/Lowest/Low; 2=Middle-Low/Middle; 3=Middle-High/High.  Entered as covariates in structural equation models with two dummy variables. |
| **Latent variables.** | | |
| Experiment injunctive smoking/vaping norms | Experimentally measured injunctive smoking/vaping norms. | Latent variable derived from items P2S2-P2S9. |
| Experiment injunctive smoking norms (non-vaping) | Experimentally measured injunctive smoking norms (non-vaping). | Latent variable derived from items P2S2-P2S6 and P2S9. |
| Experiment injunctive vaping norms (vaping only)^f^ | Experimentally measured injunctive vaping norms. | Latent variable derived from items P2S7-P2S8. |
| Experiment descriptive smoking/vaping norms^f^ | Experimentally measured descriptive smoking/vaping norms. | Latent variable derived from items P3Q1-P3Q2 (factor loadings constrained equal). |
| Self-report injunctive smoking norms | Self-report injunctive smoking norms from the survey. | Latent variable derived from items IN1-IN7. |
| Self-report descriptive smoking norms 1 | Self-report descriptive smoking norms scale 1 from the survey. | Latent variable derived from items DN1.1-DN1.5. |
| Self-report descriptive smoking norms 2 | Self-report descriptive smoking norms scale 2 from the survey. | Latent variable derived from items DN2.1-DN2.3. |
| Attitudes | Attitudes towards smoking. | Latent variable derived from items AT1-AT12 (item AT2 excluded due to poor factor loading). |
| Self-efficacy: Emotional | Self-efficacy (Emotional subscale). | Latent variable derived from items SEE1-SEE9. |
| Self-efficacy: Friends | Self-efficacy (Friends subscale). | Latent variable derived from items SEF1-SEF9. |
| Self-efficacy: Opportunity | Self-efficacy (Opportunity subscale). | Latent variable derived from items SEO1-SEO11. |
| Perceived physical risks | Perceived risks of tobacco-use (Physical subscale). | Latent variable derived from items RP1-RP7. |
| Perceived social risks | Perceived risks of tobacco-use (Social subscale). | Latent variable derived from items RS1-RS3. |
| Perceived addiction risks | Perceived risks of tobacco-use (Addiction subscale). | Latent variable derived from items RA1-RA3. |
| Perceived benefits | Perceived benefits of tobacco-use. | Latent variable derived from items BE1-BE5. |

^a^Responses to items from Parts 2 and 3 of the experiment were numerically coded to run between -1 and +1 following procedures adopted in Krupka and Weber (2013) (21).

^b^All items on the survey were coded such that higher numerical values represented greater anti-smoking norms, greater anti-smoking behavior or intentions, greater knowledge of smoking, greater anti-smoking attitudes, or higher values of the psychosocial constructs.

^c^Responses to self-report injunctive norms items were numerically coded to run between -2 and +2 following Cremers et al., (2014) (58).

^d^Responses to self-report descriptive norms items were numerically coded to run between +1 and +5 following Cremers et al., (2014) (58).

^e^In Northern Ireland, socio-economic status was based on the Northern Ireland Multiple Deprivation Measure (NIMDM2017), which ranks postcodes based on seven domains of deprivation (1=most deprived to 890=least deprived) (8). In Bogotá, socio-economic status was determined as the socio-economic level index provided by the Colombian National Administrative Department of Statistics (59).

^f^Factor loadings for two-item factors were constrained to be equal to achieve convergence, following the recommendations of Kenny et al., (1998) (60).

## Supplementary Table S4. Baseline and follow-up summary statistics.

|  | **ASSIST schools (N=6)** | | **Dead Cool schools (N=6)** | | **Northern Ireland MECHANISMS schools (N=6)** | | **Bogotá MECHANISMS schools (N=6)** | | | **Northern Ireland Control schools (N=10)^a^** | |
| --- | --- | --- | --- | --- | --- | --- | --- | --- | --- | --- | --- |
|  | **Baseline** | **Follow-up** | **Baseline** | **Follow-up** | **Baseline** | **Follow-up** | **Baseline** | | **Follow-up** | **Baseline** | **Follow-up** |
| Experiment, n | 692 | 691 | 579 | 560 | 625 | 620 | 646 | | 631 | Not conducted. | |
| Survey, n | 702 | 658 | 572 | 551 | 630 | 590 | 644 | | 619 | 220 | 207 |
| Carbon monoxide readings, n | 668 | 667 | 571 | 544 | 591 | 591 | 648 | | 620 | 200 | 172 |
| **Experiment Part 2: Injunctive smoking/vaping social norms (-1 to +1)** | | | | | | | | | | | |
| P2S2: Situation 2 (Parent smoking in front of young children) | | | | | | | | | | Not measured. | |
| *Mean (SD)* | -0.9 (0.3) | -0.8 (0.3) | -0.9 (0.2) | -0.9 (0.3) | -0.8 (0.3) | -0.8 (0.4) | -0.9 (0.2) | | -0.9 (0.2) |  |  |
| *Median (IQR)* | -1 (-1 to -1) | -1 (-1 to -0.6) | -1 (-1 to -1) | -1 (-1 to -1) | -1 (-1 to -0.6) | -1 (-1 to -0.6) | -1 (-1 to -1) | | -1 (-1 to -1) |  |  |
| *Modal response, n (%)* | 577 (83.4%) | 505 (73.1%) | 458 (79.1%) | 422 (75.4%) | 450 (72.0%) | 406 (65.5%) | 585 (90.6%) | | 521 (82.6%) |  |  |
| P2S3: Situation 3 (Adult smoking in car with under 16's onboard) | | | | | | | | | |  |  |
| *Mean (SD)* | -0.7 (0.4) | -0.7 (0.4) | -0.7 (0.3) | -0.7 (0.3) | -0.7 (0.4) | -0.7 (0.4) | -0.7 (0.3) | | -0.7 (0.3) |  |  |
| *Median (IQR)* | -0.6 (-1 to -0.6) | -0.6 (-1 to -0.6) | -0.6 (-1 to -0.6) | -0.6 (-1 to -0.6) | -0.6 (-1 to -0.6) | -0.6 (-1 to -0.6) | -0.6 (-1 to -0.6 | | -0.6 (-1 to -0.6) |  |  |
| *Modal response, n (%)* | 311 (44.9%) | 319 (46.2%) | 276 (47.7%) | 292 (52.1%) | 287 (45.9%) | 285 (46.0%) | 296 (45.8%) | | 284 (45.0%) |  |  |
| P2S4: Situation 4 (Selling cigarettes to someone who looks younger than 16 without asking for proof of age) | | | | | | | | | |  |  |
| *Mean (SD)* | -0.8 (0.4) | -0.8 (0.4) | -0.9 (0.3) | -0.8 (0.3) | -0.9 (0.3) | -0.8 (0.3) | -0.9 (0.3) | | -0.8 (0.3) |  |  |
| *Median (IQR)* | -1 (-1 to -0.6) | -1 (-1 to -0.6) | -1 (-1 to -1) | -1 (-1 to -0.6) | -1 (-1 to -1) | -1 (-1 to -0.6) | -1 (-1 to -1) | | -1 (-1 to -0.6) |  |  |
| *Modal response, n (%)* | 512 (74.0%) | 431 (62.4%) | 459 (79.3%) | 372 (66.4%) | 483 (77.3%) | 430 (69.4%) | 488 (75.5%) | | 373 (59.1%) |  |  |
| P2S5: Situation 5 (Lead actor smoking in opening scene of a recent superhero movie) | | | | | | | | | |  |  |
| *Mean (SD)* | -0.4 (0.5) | -0.3 (0.4) | -0.4 (0.4) | -0.3 (0.4) | -0.3 (0.4) | -0.3 (0.4) | -0.4 (0.4) | | -0.4 (0.4) |  |  |
| *Median (IQR)* | -0.2 (-0.6 to -0.2) | -0.2 (-0.6 to -0.2) | -0.2 (-0.6 to -0.2) | -0.2 (-0.6 to -0.2) | -0.2 (-0.6 to -0.2) | -0.2 (-0.6 to -0.2) | -0.2 (-0.6 to -0.2) | | -0.2 (-0.6 to -0.2) |  |  |
| *Modal response, n (%)* | 270 (39.0%) | 313 (45.3%) | 260 (44.9%) | 271 (48.4%) | 267 (42.7%) | 303 (48.9%) | 263 (40.7%) | | 281 (44.5%) |  |  |
| P2S6: Situation 6 (Older student smoking outside school) | | | | | | | | | |  |  |
| *Mean (SD)* | -0.5 (0.4) | -0.4 (0.4) | -0.6 (0.4) | -0.5 (0.4) | -0.5 (0.4) | -0.5 (0.4) | -0.5 (0.4) | | -0.5 (0.4) |  |  |
| *Median (IQR)* | -0.6 (-1 to -0.2) | -0.6 (-0.6 to -0.2) | -0.6 (-1 to -0.2) | -0.6 (-1 to -0.2) | -0.6 (-1 to -0.2) | -0.6 (-0.6 to -0.2) | -0.6 (-1 to -0.2) | | -0.6 (-1 to -0.2) |  |  |
| *Modal response, n (%)* | 253 (36.6%) | 310 (44.9%) | 228 (39.4%) | 249 (44.5%) | 222 (35.5%) | 202 (32.6%) | 206 (31.9%) | | 205 (32.5%) |  |  |
| P2S7: Situation 7 (School pupil using an e-cigarette whilst walking to school) | | | | | | | | | |  |  |
| *Mean (SD)* | -0.5 (0.4) | -0.4 (0.4) | -0.6 (0.4) | -0.5 (0.4) | -0.5 (0.4) | -0.5 (0.4) | -0.5 (0.4) | | -0.5 (0.4) |  |  |
| *Median (IQR)* | -0.6 (-1 to -0.2) | -0.6 (-0.6 to -0.2) | -0.6 (-1 to -0.2) | -0.6 (-1 to -0.2) | -0.6 (-1 to -0.2) | -0.6 (-0.6 to -0.2) | -0.6 (-1 to -0.2) | | -0.6 (-1 to -0.2) |  |  |
| *Modal response, n (%)* | 250 (36.1%) | 261 (37.8%) | 229 (39.6%) | 216 (38.6%) | 235 (37.6%) | 210 (33.9%) | 208 (32.2%) | | 213 (33.8%) |  |  |
| P2S8: Situation 8 (School pupil sharing a photograph of his/her e-cigarette use on social media) | | | | | | | | | |  |  |
| *Mean (SD)* | -0.5 (0.4) | -0.4 (0.4) | -0.5 (0.4) | -0.5 (0.4) | -0.5 (0.4) | -0.5 (0.4) | -0.4 (0.4) | | -0.4 (0.4) |  |  |
| *Median (IQR)* | -0.6 (-1 to -0.2) | -0.6 (-0.6 to -0.2) | -0.6 (-1 to -0.2) | -0.6 (-0.6 to -0.2) | -0.6 (-1 to -0.2) | -0.6 (-0.6 to -0.2) | -0.6 (-0.6 to -0.2) | | -0.6 (-0.6 to -0.2) |  |  |
| *Modal response, n (%)* | 237 (34.2%) | 262 (37.9%) | 244 (42.1%) | 232 (41.4%) | 201 (32.2%) | 207 (33.4%) | 245 (37.9%) | | 243 (38.5%) |  |  |
| P2S9: Situation 9 (School pupil chewing tobacco) | | | | | | | | | |  |  |
| *Mean (SD)* | -0.8 (0.3) | -0.7 (0.4) | -0.8 (0.3) | -0.7 (0.3) | -0.8 (0.4) | -0.7 (0.4) | -0.8 (0.3) | | -0.7 (0.3) |  |  |
| *Median (IQR)* | -1 (-1 to -0.6) | -1 (-1 to -0.6) | -1 (-1 to -0.6) | -1 (-1 to -0.6) | -1 (-1 to -0.6) | -1 (-1 to -0.6) | -1 (-1 to -0.6) | | -1 (-1 to -0.6) |  |  |
| *Modal response, n (%)* | 432 (62.4%) | 354 (51.2%) | 378 (65.3%) | 308 (55.0%) | 382 (61.1%) | 313 (50.5%) | 428 (66.3%) | | 349 (55.3%) |  |  |
| Experimental injunctive smoking/vaping norms scale (average P2S2 to P2S9) | | | | | | | | | |  |  |
| *Mean (SD)* | -0.6 (0.3) | -0.6 (0.3) | -0.7 (0.2) | -0.6 (0.2) | -0.6 (0.3) | -0.6 (0.3) | -0.7 (0.2) | | -0.6 (0.2) |  |  |
| *Median (IQR)* | -0.7 (-1 to 0.2) | -0.6 (-1 to 0.2) | -0.7 (-1 to -0.05) | -0.7 (-1 to 0.1) | -0.7 (-0.8 to -0.5) | -0.6 (-0.8 to -0.5) | -0.7 (-0.8 to -0.6) | | -0.7 (-0.8 to -0.5) |  |  |
| Experimental injunctive smoking norms scale (average P2S2 to P2S6 and P2S9) | | | | | | | | | |  |  |
| *Mean (SD)* | -0.7 (0.2) | -0.6 (0.2) | -0.7 (0.2) | -0.7 (0.2) | -0.7 (0.2) | -0.6 (0.2) | -0.7 (0.2) | | -0.7 (0.2) |  |  |
| *Median (IQR)* | -0.7 (-0.9 to -0.5) | -0.7 (-0.8 to -0.5) | -0.7 (-0.9 to -0.6) | -0.7 (-0.8 to -0.5) | -0.7 (-0.8 to -0.5) | -0.7 (-0.8 to -0.5) | -0.7 (-0.9 to -0.6) | | -0.7 (-0.8 to -0.5) |  |  |
| Experimental injunctive vaping norms scale (average P2S7 to P2S8) | | | | | | | | | |  |  |
| *Mean (SD)* | -0.5 (0.4) | -0.4 (0.4) | -0.5 (0.4) | -0.5 (0.3) | -0.5 (0.4) | -0.5 (0.4) | -0.5 (0.4) | | -0.5 (0.4) |  |  |
| *Median (IQR)* | -0.6 (-0.8 to -0.2) | -0.4 (-0.6 to -0.2) | -0.6 (-0.8 to -0.2) | -0.6 (-0.8 to -0.2) | -0.6 (-0.8 to -0.2) | -0.4 (-0.8 to -0.2) | -0.6 (-0.8 to -0.2) | | -0.4 (-0.8 to -0.2) |  |  |
| **Experiment Part 3: Descriptive smoking/vaping social norms (-1 to +1)** | | | | | | | | | | | |
| P3Q1: Question 1 (Proportion of peers who would be accepting of a close friend smoking) | | | | | | | | | | Not measured. | |
| *Mean (SD)* | -0.4 (0.5) | -0.3 (0.5) | -0.5 (0.4) | -0.4 (0.5) | -0.5 (0.5) | -0.3 (0.5) | | -0.5 (0.5) | -0.3 (0.5) |  |  |
| *Median (IQR)* | -0.6 (-1 to -0.2) | -0.6 (-0.6 to -0.2) | -0.6 (-1 to -0.2) | -0.6 (-0.6 to -0.2) | -0.6 (-1 to -0.2) | -0.6 (-0.6 to -0.2) | | -0.6 (-1 to -0.2) | -0.6 (-0.6 to -0.2) |  |  |
| *Modal response, n (%)* | 265 (38.3%) | 251 (36.3%) | 237 (40.9%) | 213 (38.0%) | 230 (36.8%) | 221 (35.6%) | | 239 (37.0%) | 228 (36.1%) |  |  |
| P3Q2: Question 2 (Proportion of peers who would be accepting of a close friend vaping) | | | | | | | | | |  |  |
| *Mean (SD)* | -0.3 (0.6) | -0.2 (0.6) | -0.4 (0.5) | -0.2 (0.5) | -0.3 (0.6) | -0.2 (0.6) | -0.4 (0.5) | | -0.3 (0.6) |  |  |
| *Median (IQR)* | -0.6 (-0.6 to 0.2) | -0.2 (-0.6 to 0.2) | -0.6 (-1 to -0.2) | -0.2 (-0.6 to 0.2) | -0.6 (-0.6 to 0.2) | -0.2 (-0.6 to 0.2) | -0.6 (-1 to -0.2) | | -0.2 (-0.6 to 0.2) |  |  |
| *Modal response, n (%)* | 226 (32.7%) | 206 (29.8%) | 205 (35.4%) | 190 (33.9%) | 173 (27.7%) | 174 (28.1%) | 221 (34.2%) | | 196 (31.1%) |  |  |
| Experimental descriptive smoking/vaping norms scale (average P3Q1 to P3Q2) | | | | | | | | | |  |  |
| *Mean (SD)* | -0.4 (0.5) | -0.3 (0.5) | -0.5 (0.4) | -0.3 (0.5) | -0.4 (0.5) | -0.3 (0.5) | -0.5 (0.5) | | -0.3 (0.5) |  |  |
| *Median (IQR)* | -0.4 (-1 to 1) | -0.4 (-1 to 1) | -0.6 (-1 to 0.8) | -0.4 (-1 to 0.8) | -0.5 (-0.8 to 0) | -0.4 (-0.6 to 0) | -0.6 (-0.8 to -0.2) | | -0.4 (-0.6 to 0) |  |  |
| **Experiment Part 4: Willingness to pay to support anti-smoking norms (0 to 10)** | | | | | | | | | | | |
| Donation: number of tokens donated to ASSIST/Dead Cool; 0-10 | | | | | | | | | | Not measured. | |
| *Mean (SD)* | 3.6 (2.8) | 3.4 (2.6) | 3.9 (2.9) | 3.2 (2.7) | 3.5 (3.1) | 3.0 (2.8) | 3.9 (2.6) | | 3.6 (2.4) |  |  |
| *Median (IQR)* | 4 (1 to 5) | 4 (1 to 5) | 4 (2 to 5) | 3 (0 to 5) | 3 (1 to 5) | 2 (0 to 5) | 5 (2 to 5) | | 4 (1 to 5) |  |  |
| **Survey: Self-report injunctive smoking social norms (-2 to +2)** | | | | | | | | | | | |
| IN1: Most of the people who are important to me think that I… | | | | | | | | | | Not measured. | |
| *Mean (SD)* | 1.8 (0.7) | 1.7 (0.7) | 1.7 (0.7) | 1.7 (0.7) | 1.7 (0.7) | 1.7 (0.7) | 1.8 (0.7) | | 1.7 (0.8) |  |  |
| *Definitely should smoke-Maybe should not smoke, n (%)* | 106 (15.01%) | 121 (18.4%) | 88 (15.4%) | 83 (15.1%) | 94 (14.9%) | 97 (16.4%) | 97 (15.1%) | | 107 (17.3%) |  |  |
| *Definitely should not smoke, n (%)* | 595 (84.8%) | 529 (80.4%) | 481 (84.1%) | 467 (84.8%) | 529 (84.0%) | 484 (82.0%) | 547 (84.9%) | | 512 (82.7%) |  |  |
| IN2: My mother thinks that I… | | | | | | | | | |  |  |
| *Mean (SD)* | 1.9 (0.4) | 1.9 (0.5) | 1.9 (0.3) | 1.9 (0.4) | 1.9 (0.3) | 1.9 (0.4) | 1.9 (0.4) | | 1.9 (0.5) |  |  |
| *Definitely should smoke-Maybe should not smoke, n (%)* | 40 (5.7%) | 42 (6.4%) | 22 (3.8%) | 27 (4.9%) | 24 (3.8%) | 24 (4.1%) | 38 (5.9%) | | 45 (7.3%) |  |  |
| *Definitely should not smoke, n (%)* | 655 (93.3%) | 606 (92.1%) | 546 (95.5%) | 520 (94.4%) | 598 (94.9%) | 556 (94.2%) | 603 (93.6%) | | 570 (92.1%) |  |  |
| *I don't have a mother, n (%)* | 4 (0.6%) | 3 (0.5%) | 1 (0.2%) | 3 (0.5%) | 2 (0.3%) | 2 (0.3%) | 3 (0.5%) | | 4 (0.6%) |  |  |
| IN3: My father thinks that I… | | | | | | | | | |  |  |
| *Mean (SD)* | 1.7 (0.7) | 1.7 (0.7) | 1.8 (0.6) | 1.8 (0.6) | 1.8 (0.6) | 1.8 (0.6) | 1.7 (0.7) | | 1.7 (0.7) |  |  |
| *Definitely should smoke-Maybe should not smoke, n (%)* | 59 (8.4%) | 74 (11.2%) | 38 (6.6%) | 42 (7.6%) | 30 (4.8%) | 38 (6.4%) | 67 (10.4%) | | 78 (12.6%) |  |  |
| *Definitely should not smoke, n (%)* | 579 (82.5%) | 538 (81.8% | 504 (88.1%) | 480 (87.1%) | 566 (89.8%) | 520 (88.1%) | 517 (80.3%) | | 498 (80.5%) |  |  |
| *I don't have a father, n (%)* | 60 (8.5%) | 38 (5.8%) | 25 (4.4%) | 28 (5.1%) | 25 (4.0%) | 23 (3.9%) | 60 (9.3%) | | 43 (6.9%) |  |  |
| IN4: My brother(s) think(s) that I… | | | | | | | | | |  |  |
| *Mean (SD)* | 1.3 (0.9) | 1.4 (0.9) | 1.4 (0.9) | 1.4 (0.9) | 1.4 (0.9) | 1.4 (0.9) | 1.4 (0.9) | | 1.5 (0.8) |  |  |
| *Definitely should smoke-Maybe should not smoke, n (%)* | 104 (14.8%) | 100 (15.2%) | 87 (15.2%) | 72 (13.1%) | 70 (11.1%) | 75 (12.7%) | 121 (18.8%) | | 97 (15.7%) |  |  |
| *Definitely should not smoke, n (%)* | 447 (63.7%) | 444 (67.5%) | 391 (68.4%) | 384 (69.7%) | 415 (65.9%) | 389 (65.9%) | 423 (65.7%) | | 439 (70.9%) |  |  |
| *I don't have a brother, n (%)* | 145 (20.7%) | 106 (16.1%) | 90 (15.7%) | 94 (17.1%) | 135 (21.4%) | 117 (19.8%) | 100 (15.5%) | | 83 (13.4%) |  |  |
| IN5: My sister(s) think(s) that I… | | | | | | | | | |  |  |
| *Mean (SD)* | 1.3 (1.0) | 1.4 (0.9) | 1.4 (0.9) | 1.4 (0.9) | 1.4 (0.9) | 1.4 (0.9) | 1.3 (0.9) | | 1.4 (0.8) |  |  |
| *Definitely should smoke-Maybe should not smoke, n (%)* | 84 (12.0%) | 78 (11.9%) | 54 (9.4%) | 54 (9.8%) | 43 (6.8%) | 56 (9.5%) | 95 (14.8%) | | 76 (12.3%) |  |  |
| *Definitely should not smoke, n (%)* | 447 (63.7%) | 439 (66.7%) | 395 (69.1%) | 382 (69.3%) | 436 (69.2%) | 396 (67.1%) | 406 (63.0%) | | 425 (68.7%) |  |  |
| *I don't have a sister, n (%)* | 167 (23.8%) | 133 (20.2%) | 119 (20.8%) | 114 (20.7%) | 143 (22.7%) | 129 (21.9%) | 143 (22.2%) | | 118 (19.1%) |  |  |
| IN6: My friends think that I… | | | | | | | | | |  |  |
| *Mean (SD)* | 1.4 (0.9) | 1.4 (1.0) | 1.4 (0.9) | 1.5 (0.9) | 1.5 (0.9) | 1.5 (0.9) | 1.3 (0.9) | | 1.3 (0.9) |  |  |
| *Definitely should smoke-Maybe should not smoke, n (%)* | 261 (37.2%) | 237 (36.0%) | 193 (33.7%) | 171 (31.0%) | 193 (30.6%) | 149 (25.3%) | 261 (40.5%) | | 259 (41.8%) |  |  |
| *Definitely should not smoke, n (%)* | 434 (61.8%) | 409 (62.2%) | 372 (65.0%) | 374 (67.9%) | 425 (67.5%) | 425 (72.0%) | 381 (59.2%) | | 358 (57.8%) |  |  |
| *I don't have a friend, n (%)* | 4 (0.6%) | 4 (0.6%) | 3 (0.5%) | 5 (0.9%) | 5 (0.8%) | 7 (1.2%) | 2 (0.3%) | | 2 (0.3%) |  |  |
| IN7: My best friend thinks that I… | | | | | | | | | |  |  |
| *Mean (SD)* | 1.5 (0.8) | 1.5 (0.9) | 1.6 (0.8) | 1.6 (0.7) | 1.7 (0.7) | 1.7 (0.8) | 1.5 (0.9) | | 1.5 (0.9) |  |  |
| *Definitely should smoke-Maybe should not smoke, n (%)* | 157 (22.4%) | 151 (22.9%) | 105 (18.4%) | 104 (18.9%) | 106 (16.8%) | 101 (17.1%) | 156 (24.2%) | | 154 (24.9%) |  |  |
| *Definitely should not smoke, n (%)* | 517 (73.6%) | 472 (71.7%) | 443 (77.4%) | 427 (77.5%) | 508 (80.6%) | 472 (80.0%) | 452 (70.2%) | | 427 (69.0%) |  |  |
| *I don't have a best friend, n (%)* | 25 (3.6%) | 28 (4.3%) | 20 (3.5%) | 19 (3.4%) | 9 (1.4%) | 9 (1.5%) | 36 (5.6%) | | 38 (6.1%) |  |  |
| Self-report injunctive smoking norms scale (average IN1 to IN7) | | | | | | | | | |  |  |
| *Mean (SD)* | 1.6 (0.5) | 1.6 (0.5) | 1.6 (0.5) | 1.6 (0.5) | 1.6 (0.5) | 1.6 (0.5) | 1.5 (0.5) | | 1.6 (0.5) |  |  |
| *Median (IQR)* | 1.7 (0 to 2) | 1.7 (-0.4 to 2) | 1.7 (0 to 2) | 1.7 (0 to 2) | 1.7 (1.4 to 2) | 1.7 (1.4 to 2) | 1.7 (1.3 to 2) | | 1.7 (1.3 to 2) |  |  |
| **Survey: Self-report descriptive smoking social norms 1 (1 to 5)** | | | | | | | | | | | |
| DN1.1: Does your best friend smoke? | | | | | | | | | | Not measured. | |
| *Mean (SD)* | 4.7 (0.8) | 4.7 (0.8) | 4.9 (0.5) | 4.9 (0.5) | 4.8 (0.8) | 4.7 (0.8) | 4.9 (0.6) | | 4.8 (0.6) |  |  |
| *Very often-Rarely, n (%)* | 81 (11.5%) | 88 (13.4%) | 36 (6.3%) | 48 (8.7%) | 71 (11.3%) | 80 (13.6%) | 46 (7.1%) | | 56 (9.0%) |  |  |
| *Never/Don't know, n (%)* | 563 (80.2%) | 505 (76.7%) | 501 (87.6%) | 464 (84.2%) | 538 (85.4%) | 474 (80.3%) | 526 (81.7%) | | 495 (80.0%) |  |  |
| *I don't have a best friend, n (%)* | 54 (7.7%) | 59 (9.0%) | 33 (5.8%) | 38 (6.9%) | 15 (2.4%) | 29 (4.9%) | 72 (11.2%) | | 68 (11.0%) |  |  |
| DN1.2: Does your mother smoke? | | | | | | | | | |  |  |
| *Mean (SD)* | 4.3 (1.3) | 4.3 (1.2) | 4.6 (1.0) | 4.6 (1.0) | 4.2 (1.4) | 4.3 (1.3) | 4.6 (0.9) | | 4.6 (0.9) |  |  |
| *Very often-Rarely, n (%)* | 195 (27.8%) | 172 (26.1%) | 114 (19.9%) | 100 (18.1%) | 193 (30.6%) | 170 (28.8%) | 116 (18.0%) | | 102 (16.5%) |  |  |
| *Never/Don't know, n (%)* | 500 (71.2%) | 475 (72.2%) | 454 (79.4%) | 446 (80.9%) | 430 (68.3%) | 408 (69.2%) | 524 (81.4%) | | 513 (82.9%) |  |  |
| *I don't have a mother, n (%)* | 4 (0.6%) | 5 (0.8%) | 2 (0.3%) | 4 (0.7%) | 2 (0.3%) | 5 (0.8%) | 4 (0.6%) | | 4 (0.6%) |  |  |
| DN1.3: Does your father smoke? | | | | | | | | | |  |  |
| *Mean (SD)* | 4.2 (1.4) | 4.3 (1.3) | 4.4 (1.2) | 4.4 (1.2) | 4.1 (1.4) | 4.2 (1.4) | 4.4 (1.1) | | 4.5 (1.1) |  |  |
| *Very often-Rarely, n (%)* | 212 (30.2%) | 189 (28.7%) | 139 (24.3%) | 135 (24.5%) | 191 (30.3%) | 175 (29.7%) | 97 (15.1%) | | 149 (24.1%) |  |  |
| *Never/Don't know, n (%)* | 425 (60.5%) | 414 (62.9%) | 400 (69.9%) | 382 (69.3%) | 402 (63.8%) | 375 (63.6%) | 125 (19.4%) | | 421 (68.0%) |  |  |
| *I don't have a father, n (%)* | 62 (8.8%) | 49 (7.4%) | 30 (5.2%) | 33 (6.0%) | 31 (4.9%) | 33 (5.6%) | 361 (56.1%) | | 49 (7.9%) |  |  |
| DN1.4: Do any of your brothers smoke? | | | | | | | | | |  |  |
| *Mean (SD)* | 4.7 (0.9) | 4.6 (1.0) | 4.8 (0.8) | 4.7 (0.8) | 4.7 (0.9) | 4.7 (0.9) | 4.7 (0.8) | | 4.7 (0.9) |  |  |
| *Very often-Rarely, n (%)* | 87 (12.4%) | 95 (14.4%) | 51 (8.9%) | 61 (11.1%) | 64 (10.2%) | 75 (12.7%) | 74 (11.5%) | | 81 (13.1%) |  |  |
| *Never/Don't know, n (%)* | 435 (62.0%) | 425 (64.6%) | 403 (70.5%) | 369 (67.0%) | 397 (63.0%) | 364 (61.7%) | 441 (68.5%) | | 428 (69.1%) |  |  |
| *I don't have a brother, n (%)* | 176 (25.1%) | 132 (20.1%) | 116 (20.3%) | 120 (21.8%) | 163 (25.9%) | 142 (24.1%) | 129 (20.0%) | | 110 (17.8%) |  |  |
| DN1.5: Do any of your sisters smoke? | | | | | | | | | |  |  |
| *Mean (SD)* | 4.8 (0.7) | 4.8 (0.8) | 4.8 (0.7) | 4.8 (0.7) | 4.8 (0.7) | 4.8 (0.8) | 4.8 (0.7) | | 4.8 (0.7) |  |  |
| *Very often-Rarely, n (%)* | 58 (8.3%) | 65 (9.9%) | 36 (6.3%) | 34 (6.2%) | 45 (7.1%) | 48 (8.1%) | 49 (7.6%) | | 51 (8.2%) |  |  |
| *Never/Don't know, n (%)* | 433 (61.7%) | 396 (60.2%) | 392 (68.5%) | 374 (67.9%) | 413 (65.6%) | 376 (63.7%) | 412 (64.0%) | | 394 (63.7%) |  |  |
| *I don't have a sister, n (%)* | 207 (29.5%) | 191 (29.0%) | 142 (24.8%) | 142 (25.8%) | 166 (26.3%) | 159 (26.9%) | 183 (28.4%) | | 174 (28.1%) |  |  |
| Self-report descriptive smoking norms scale 1 (average DN1.1 to DN1.5) | | | | | | | | | |  |  |
| *Mean (SD)* | 4.5 (0.7) | 4.5 (0.7) | 4.7 (0.5) | 4.7 (0.5) | 4.5 (0.7) | 4.5 (0.7) | 4.7 (0.5) | | 4.7 (0.5) |  |  |
| *Median (IQR)* | 4.8 (2.2 to 5) | 4.8 (2.2 to 5) | 5 (3 to 5) | 5 (2.8 to 5) | 4.8 (4.2 to 5) | 4.8 (4.2 to 5) | 5 (4.4 to 5) | | 5 (4.6 to 5) |  |  |
| **Survey: Self-report descriptive smoking social norms 2 (1 to 5)** | | | | | | | | | | | |
| DN2.1: How many of your friends smoke? | | | | | | | | | | Not measured. | |
| *Mean (SD)* | 4.7 (0.8) | 4.6 (0.9) | 4.8 (0.6) | 4.8 (0.6) | 4.7 (0.7) | 4.6 (0.8) | 4.7 (0.6) | | 4.7 (0.7) |  |  |
| *Almost all-A few of them, n (%)* | 162 (23.1%) | 172 (26.1%) | 92 (16.1%) | 101 (18.3%) | 139 (22.1%) | 146 (24.7%) | 115 (17.9%) | | 127 (20.5%) |  |  |
| *Almost none/Don’t know, n (%)* | 523 (74.5%) | 468 (71.1%) | 467 (81.6%) | 436 (79.1%) | 475 (75.4%) | 424 (71.9%) | 515 (80.0%) | | 480 (77.5%) |  |  |
| *I don’t have friends, n (%)* | 14 (2.0%) | 12 (1.8%) | 11 (1.9%) | 13 (2.4%) | 11 (1.7%) | 13 (2.2%) | 14 (2.2%) | | 12 (1.9%) |  |  |
| DN2.2: How many of your other family members smoke? | | | | | | | | | |  |  |
| *Mean (SD)* | 4.2 (1.0) | 4.2 (1.0) | 4.4 (0.9) | 4.4 (0.9) | 4.1 (1.0) | 4.1 (1.1) | 4.4 (0.9) | | 4.5 (0.9) |  |  |
| *Almost all-A few of them, n (%)* | 345 (49.1%) | 306 (46.5%) | 239 (41.8%) | 227 (41.2%) | 350 (55.6%) | 310 (52.5%) | 234 (36.3%) | | 223 (36.0%) |  |  |
| *Almost none/Don’t know, n (%)* | 350 (49.9%) | 342 (52.0%) | 323 (56.5%) | 315 (57.2%) | 273 (43.3%) | 264 (44.7%) | 400 (62.1%) | | 393 (63.5%) |  |  |
| *I don’t have other family members, n (%)* | 4 (0.6%) | 4 (0.6%) | 8 (1.4%) | 7 (1.3%) | 2 (0.3%) | 8 (1.4%) | 10 (1.6%) | | 3 (0.5%) |  |  |
| DN2.3: How many of your classmates smoke? | | | | | | | | | |  |  |
| *Mean (SD)* | 4.7 (0.6) | 4.6 (0.7) | 4.8 (0.5) | 4.8 (0.6) | 4.7 (0.7) | 4.6 (0.7) | 4.8 (0.5) | | 4.8 (0.6) |  |  |
| *Almost all-A few of them, n (%)* | 158 (22.5%) | 167 (25.4%) | 74 (12.9%) | 81 (14.7%) | 149 (23.7%) | 158 (26.8%) | 83 (12.9%) | | 90 (14.5%) |  |  |
| *Almost none/Don’t know, n (%)* | 538 (76.6%) | 480 (72.9%) | 491 (85.8%) | 461 (82.8%) | 474 (75.2%) | 420 (71.2%) | 555 (86.2%) | | 521 (84.2%) |  |  |
| *I don’t have classmates, n (%)* | 3 (0.4%) | 5 (0.8%) | 5 (0.9%) | 8 (1.5%) | 2 (0.3%) | 5 (0.8%) | 6 (0.9%) | | 8 (1.3%) |  |  |
| Self-report descriptive smoking norms scale 2 (average DN2.1 to DN2.3) | | | | | | | | | |  |  |
| *Mean (SD)* | 4.5 (0.6) | 4.5 (0.7) | 4.7 (0.5) | 4.6 (0.5) | 4.5 (0.6) | 4.5 (0.7) | 4.7 (0.5) | | 4.6 (0.5) |  |  |
| *Median (IQR)* | 4.7 (2.3 to 5) | 4.7 (2 to 5) | 4.7 (3 to 5) | 4.7 (2.7 to 5) | 4.7 (4.3 to 5) | 4.7 (4.3 to 5) | 5 (4.7 to 5) | | 5 (4.7 to 5) |  |  |
| **Survey: Self-report smoking behavior** | | | | | | | | | | | |
| Self-report smoking behavior (1 to 4) | | | | | | | | | | | |
| *Mean (SD)* | 3.7 (0.7) | 3.7 (0.7) | 3.8 (0.5) | 3.7 (0.6) | 3.8 (0.6) | 3.8 (0.7) | 3.7 (0.7) | | 3.6 (0.7) | 3.8 (0.5) | 3.8 (0.6) |
| *Sometimes smoke, n (%)* | 16 (2.3%) | 19 (2.9%) | 4 (0.7%) | 8 (1.5%) | 13 (2.1%) | 18 (3.1%) | 7 (1.1%) | | 9 (1.5%) | 4 (1.8%) | 3 (1.4%) |
| *Previous smoker, n (%)* | 46 (6.6%) | 45 (6.8%) | 26 (4.5%) | 39 (7.1%) | 19 (3.0%) | 24 (4.1%) | 53 (8.2%) | | 60 (9.7%) | 3 (1.4%) | 5 (2.4%) |
| *Smoked once, n (%)* | 59 (8.4%) | 73 (11.1%) | 40 (7.0%) | 42 (7.6%) | 43 (6.8%) | 43 (7.3%) | 56 (8.7%) | | 72 (11.6%) | 18 (8.2%) | 24 (1.2%) |
| *Never smoked, n (%)* | 581 (82.8%) | 521 (79.2%) | 502 (87.8%) | 462 (83.8%) | 555 (88.1%) | 505 (85.6%) | 528 (82.0%) | | 478 (77.2%) | 195 (88.6%) | 172 (83.1%) |
| **Survey: Smoking intentions and susceptibility** | | | | | | | | | | | |
| Intentions to take up smoking in the next 6 months (1 to 6) | | | | | | | | | | | |
| *Mean (SD)* | 5.6 (1.0) | 5.4 (1.3) | 5.7 (0.9) | 5.6 (1.0) | 5.7 (0.8) | 5.7 (0.9) | 5.5 (1.1) | | 5.3 (1.3) | 5.6 (0.8) | 5.6 (0.8) |
| *I am a smoker, n (%)* | 20 (2.8%) | 34 (5.2%) | 13 (2.3%) | 11 (2.0%) | 10 (1.6%) | 12 (2.0%) | 23 (3.6%) | | 33 (5.3%) | N/A. | N/A. |
| *Definitely start smoking, n (%)* | 2 (0.3%) | 0 (0.0%) | 0 (0.0%) | 1 (0.2%) | 1 (0.2%) | 1 (0.2%) | 1 (0.2%) | | 0 (0.0%) | 2 (0.9%) | 4 (1.9%) |
| *Probably start smoking, n (%)* | 3 (4.3%) | 8 (1.2%) | 2 (0.3%) | 6 (1.1%) | 0 (0.0%) | 2 (0.3%) | 5 (0.8%) | | 12 (1.9%) | 4 (1.8%) | 0 (0.0%) |
| *Don’t know, n (%)* | 67 (9.5%) | 71 (10.8%) | 43 (7.5%) | 50 (9.1%) | 46 (7.3%) | 47 (0.8%) | 64 (9.9%) | | 74 (12.0%) | 18 (8.2%) | 18 (8.7%) |
| *Probably remain a non-smoker, n (%)* | 47 (6.7%) | 53 (8.1%) | 36 (6.3%) | 45 (8.2%) | 41 (6.5%) | 40 (6.8%) | 42 (6.5%) | | 58 (9.4%) | 27 (12.3%) | 31 (15.0%) |
| *Definitely remain a non-smoker, n (%)* | 561 (79.9%) | 490 (74.5%) | 476 (83.2%) | 437 (79.3%) | 528 (83.8%) | 485 (82.2%) | 509 (79.0%) | | 442 (71.4%) | 167 (75.9%) | 153 (73.9%) |
| Susceptibility to commencing smoking (0=not susceptible, 1=susceptible), n(%) | | | | | | | | | | | |
| *Susceptible* | 261 (37.2%) | 297 (45.1%) | 197 (34.4%) | 217 (39.4%) | 199 (31.6%) | 199 (33.7%) | 259 (40.2%) | | 315 (50.9%) | 67 (30.5%) | 69 (33.3%) |
| *Not susceptible* | 439 (62.5%) | 359 (54.6%) | 373 (65.2%) | 333 (60.4%) | 427 (67.8%) | 388 (65.8%) | 385 (59.8%) | | 304 (49.1%) | 146 (66.4%) | 134 (64.7%) |
| **Survey: Smoking knowledge, and attitudes** | | | | | | | | | | | |
| Knowledge: Knowledge of smoking (0 to 6) | | | | | | | | | | Not measured. | |
| *Mean (SD)* | 2.5 (1.5) | 2.7 (1.6) | 2.7 (1.5) | 3.1 (1.4) | 3.0 (1.5) | 3.3 (1.5) | 2.2 (1.4) | | 2.5 (1.5) |  |  |
| *Median (IQR)* | 3 (2 to 3) | 3 (2 to 4) | 3 (2 to 4) | 3 (2 to 4) | 3 (2 to 4) | 3 (2 to 4) | 2 (1 to 3) | | 3 (2 to 3) |  |  |
| Attitudes: Attitudes towards smoking (1 to 5) | | | | | | | | | |  |  |
| *Mean (SD)* | 3.9 (0.6) | 3.9 (0.7) | 4.0 (0.6) | 4.0 (0.6) | 4.0 (0.6) | 4.0 (0.6) | 3.9 (0.7) | | 3.9 (0.7) |  |  |
| *Median (IQR)* | 4 (3.5 to 4.3) | 4 (3.4 to 4.4) | 4.1 (3.7 to 4.5) | 4.1 (3.7 to 4.5) | 4 (3.6 to 4.4) | 4.1 (3.6 to 4.5) | 4 (3.5 to 4.4) | | 4 (3.5 to 4.4) |  |  |
| **Survey: Psychosocial variables and mediators** | | | | | | | | | | | |
| Self-efficacy (Emotional; 1 to 6) | | | | | | | | | | Not measured. | |
| *Mean (SD)* | 5.6 (0.9) | 5.5 (1.0) | 5.7 (0.7) | 5.6 (0.8) | 5.7 (0.8) | 5.7 (0.9) | 5.6 (0.8) | | 5.4 (0.9) |  |  |
| *Median (IQR)* | 6 (5.6 to 6) | 6 (5.1 to 6) | 6 (5.8 to 6) | 6 (5.4 to 6) | 6 (6 to 6) | 6 (6 to 6) | 6 (5.3 to 6) | | 6 (5 to 6) |  |  |
| Self-efficacy (Friends; 1 to 6) | | | | | | | | | |  |  |
| *Mean (SD)* | 5.6 (0.9) | 5.5 (0.9) | 5.7 (0.6) | 5.6 (0.8) | 5.7 (0.8) | 5.7 (0.8) | 5.6 (0.7) | | 5.5 (0.9) |  |  |
| *Median (IQR)* | 6 (5.6 to 6) | 6 (5.3 to 6) | 6 (5.8 to 6) | 6 (5.6 to 6) | 6 (5.8 to 6) | 6 (5.7 to 6) | 6 (5.6 to 6) | | 5.9 (5.1 to 6) |  |  |
| Self-efficacy (Opportunity; 1 to 6) | | | | | | | | | |  |  |
| *Mean (SD)* | 5.7 (0.7) | 5.7 (0.8) | 5.9 (0.4) | 5.8 (0.6) | 5.8 (0.6) | 5.8 (0.6) | 5.7 (0.6) | | 5.6 (0.8) |  |  |
| *Median (IQR)* | 6 (6 to 6) | 6 (5.9 to 6) | 6 (6 to 6) | 6 (6 to 6) | 6 (6 to 6) | 6 (6 to 6) | 6 (5.9 to 6) | | 6 (5.6 to 6) |  |  |
| Perceived physical risks (0% to 100%) | | | | | | | | | |  |  |
| *Mean (SD)* | 60.1 (24.1) | 62.1 (23.4) | 61.9 (24.4) | 67.1 (22.4) | 62.5 (21.6) | 66.0 (20.4) | 59.4 (26.5) | | 62.9 (25.3) |  |  |
| *Median (IQR)* | 62 (45.2 to 77.9) | 64.3 (46.7 to 78.7) | 63.8 (46.1 to 79.3) | 70 (54.3 to 84.1) | 63.8 (49.1 to 77.8) | 67.7 (53.9 to 80) | 61.4 (41.1 to 79.6) | | 67.4 (45.9 to 82.1) |  |  |
| Perceived social risks (0% to 100%) | | | | | | | | | |  |  |
| *Mean (SD)* | 67.5 (26.6) | 67.4 (25.8) | 68.9 (26.9) | 72.5 (24.6) | 75.1 (22.0) | 75.9 (22.2) | 61.5 (29.1) | | 63.8 (26.8) |  |  |
| *Median (IQR)* | 70 (50 to 88.3) | 70 (52.3 to 87.7) | 73.7 (51.3 to 91.7) | 76.7 (57 to 94.3) | 80 (63.3 to 92.7) | 80 (64.3 to 95) | 66.5 (41 to 84.3) | | 67 (47.3 to 84) |  |  |
| Perceived addiction risks (0% to 100%) | | | | | | | | | |  |  |
| *Mean (SD)* | 35.3 (25.2) | 38.1 (25.6) | 35.1 (27.1) | 39.7 (26.6) | 43.4 (24.9) | 47.5 (24.0) | 27.7 (24.9) | | 30.2 (25.1) |  |  |
| *Median (IQR)* | 33.3 (13.3 to 54.7) | 36.7 (16.7 to 57.3) | 33.5 (6.7 to 56) | 38.3 (17.7 to 60.3) | 43.2 (28.3 to 63.3) | 50 (33.3 to 66) | 27 (2.7 to 44.8) | | 31 (4 to 49.7) |  |  |
| Perceived benefits (0% to 100%) | | | | | | | | | |  |  |
| *Mean (SD)* | 24.4 (22.1) | 23.6 (21.5) | 22.7 (20.9) | 24.1 (21.5) | 23.4 (22.1) | 24.0 (20.9) | 23.8 (21.1) | | 23.7 (22.0) |  |  |
| *Median (IQR)* | 20 (5.8 to 36.8) | 20 (4.4 to 35.9) | 18.9 (4.4 to 34.6) | 20 (6.2 to 36.2) | 19 (4 to 36.2) | 20 (5 to 38) | 20 (6.1 to 34.9) | | 20 (5.2 to 35.2) |  |  |
| Perceived behavioral control (easy to quit; 1 to 5) | | | | | | | | | |  |  |
| *Mean (SD)* | 3.0 (1.4) | 3.0 (1.5) | 3.0 (1.5) | 2.9 (1.4) | 2.5 (1.4) | 2.4 (1.4) | 3.5 (1.3) | | 3.5 (1.3) |  |  |
| *Strongly disagree/Disagree* | 261 (37.2%) | 246 (37.4%) | 209 (36.5%) | 215 (39.0%) | 339 (53.8%) | 328 (55.6%) | 131 (20.3%) | | 133 (21.5%) |  |  |
| *Unsure* | 187 (26.6%) | 178 (27.1%) | 146 (25.5%) | 143 (26.0%) | 140 (22.2%) | 138 (23.4%) | 193 (30.0%) | | 183 (29.6%) |  |  |
| *Strongly agree/Agree* | 247 (35.2%) | 231 (35.1%) | 212 (37.1%) | 190 (34.5%) | 139 (22.1%) | 118 (20.0%) | 320 (49.7%) | | 303 (48.9%) |  |  |
| Perceived behavioral control (to avoid smoking; 1 to 5) | | | | | | | | | |  |  |
| *Mean (SD)* | 4.1 (1.2) | 4.1 (1.2) | 4.2 (1.2) | 4.2 (1.1) | 4.3 (1.1) | 4.3 (1.0) | 4.0 (1.3) | | 4.0 (1.3) |  |  |
| *Strongly disagree/Disagree* | 74 (10.5%) | 68 (10.3%) | 52 (9.1%) | 47 (8.5%) | 49 (7.8%) | 37 (6.3%) | 77 (12.0%) | | 78 (12.6%) |  |  |
| *Unsure* | 103 (14.7%) | 105 (16.0%) | 69 (12.1%) | 74 (13.4%) | 67 (10.6%) | 70 (11.9%) | 105 (16.3%) | | 109 (17.6%) |  |  |
| *Strongly agree/Agree* | 522 (74.4%) | 482 (73.3%) | 448 (78.3%) | 426 (77.3%) | 508 (80.6%) | 476 (80.7%) | 462 (71.7%) | | 432 (69.8%) |  |  |
| Exposure to advertising in the media (0 to 8) | | | | | | | | | |  |  |
| *Mean (SD)* | 2.7 (2.1) | 2.8 (2.3) | 2.4 (2.0) | 3.9 (2.2) | 2.4 (2.2) | 2.8 (2.5) | 2.8 (1.9) | | 3.7 (2.0) |  |  |
| *Median (IQR)* | 3 (1 to 4) | 3 (1 to 4) | 2 (1 to 4) | 4 (2 to 5) | 2 (0 to 4) | 3 (0 to 4) | 3 (1 to 4) | | 4 (2 to 5) |  |  |
| Exposure to advertising in shops (0 to 4) | | | | | | | | | | | |
| *Mean (SD)* | 2.2 (1.2) | 2.2 (1.3) | 2.2 (1.2) | 2.4 (1.2) | 2.4 (1.3) | 2.5 (1.3) | 2.1 (1.1) | | 2.1 (1.2) | 2.5 (1.1) | 2.6 (1.2) |
| *Median (IQR)* | 2 (1 to 3) | 2 (1 to 3) | 2 (1 to 3) | 2 (2 to 3) | 3 (1 to 3) | 3 (1 to 4) | 2 (1 to 3) | | 2 (1 to 3) | 3 (2 to 3) | 3 (2 to 4) |
| **Objectively measured smoking behavior** | | | | | | | | | | | |
| Carbon monoxide reading (expelled air; 0 to 30 parts per million) | | | | | | | | | | | |
| *Mean (SD)* | 2.7 (1.8) | 2.7 (1.6) | 2.3 (1.6) | 2.8 (1.5) | 1.5 (1.4) | 2.0 (1.0) | 3.4 (1.5) | | 3.5 (1.7) | 2.3 (1.9) | 1.9 (1.1) |
| *Non-smoker (≤9 ppm), n (%)* | 664 (99.4%) | 663 (99.4%) | 569 (99.6%) | 542 (99.6%) | 590 (99.8%) | 591 (100.0%) | 643 (99.2%) | | 614 (99.0%) | 197 (98.5%) | 172 (100.0%) |
| *Smoker (>9 ppm), n (%)* | 4 (0.6%) | 4 (0.6%) | 2 (0.3%) | 2 (0.4%) | 1 (0.2%) | 0 (0.0%) | 5 (0.8%) | | 6 (1.0%) | 3 (1.5%) | 0 (0.0%) |

^a^N/A: Not applicable.

##

## Supplementary Table S5. Spearman's rank-order correlations between baseline variables, including data from MECHANISMS schools.

|  | **(1)** | **(2)** | **(3)** | **(4)** | **(5)** | **(6)** | **(7)** | **(8)** | **(9)** | **(10)** | **(11)** |
| --- | --- | --- | --- | --- | --- | --- | --- | --- | --- | --- | --- |
|  | **Expt. injunctive norms** | **Expt. descriptive norms** | **Self-report injunctive norms** | **Self-report descriptive norms 1** | **Self-report descriptive norms 2** | **Donation** | **Self-report smoking behavior** | **Intentions** | **Knowledge** | **Attitudes** | **Objectively measured smoking behavior** |
| **(1)** | 1.00 |  |  |  |  |  |  |  |  |  |  |
| **(2)** | 0.27*** | 1.00 |  |  |  |  |  |  |  |  |  |
| **(3)** | -0.16*** | -0.13*** | 1.00 |  |  |  |  |  |  |  |  |
| **(4)** | -0.11*** | -0.15*** | 0.13*** | 1.00 |  |  |  |  |  |  |  |
| **(5)** | -0.11*** | -0.26*** | 0.18*** | 0.40*** | 1.00 |  |  |  |  |  |  |
| **(6)** | -0.08*** | -0.05* | 0.004 | -0.003 | 0.03 | 1.00 |  |  |  |  |  |
| **(7)** | -0.11*** | -0.17*** | 0.17*** | 0.29*** | 0.25*** | 0.03 | 1.00 |  |  |  |  |
| **(8)** | -0.13*** | -0.15*** | 0.21*** | 0.22*** | 0.22*** | 0.02 | 0.40*** | 1.00 |  |  |  |
| **(9)** | -0.05* | 0.08** | 0.10*** | -0.003 | -0.11*** | -0.02 | 0.08*** | 0.13*** | 1.00 |  |  |
| **(10)** | -0.29*** | -0.21*** | 0.28*** | 0.17*** | 0.23*** | 0.08*** | 0.24*** | 0.31*** | 0.18*** | 1.00 |  |
| **(11)** | 0.04 | 0.03 | -0.08** | 0.06** | 0.08*** | 0.03 | -0.11*** | -0.08*** | -0.15*** | -0.07** | 1.00 |

Expt. = Experiment; ***p<0.01; **p<0.05; *p<0.10.

##

## Supplementary Table S6. Spearman's rank-order correlations between baseline variables, including data from MECHANISMS schools.

|  | **(1)** | **(2)** | **(3)** | **(4)** | **(5)** | **(6)** | **(7)** | **(8)** | **(9)** | **(10)** | **(11)** | **(12)** | **(13)** | **(14)** | **(15)** | **(16)** | **(17)** |
| --- | --- | --- | --- | --- | --- | --- | --- | --- | --- | --- | --- | --- | --- | --- | --- | --- | --- |
|  | **Self-efficacy emotional** | **Self-efficacy friends** | **Self-efficacy opp.** | **Perceived physical risks** | **Perceived social risks** | **Perceived addiction risks** | **Perceived benefits** | **PBC (easy to quit)** | **PBC (to avoid smoking)** | **Exposure to adverts in media** | **Exposure to adverts in shops** | **Donation** | **Self-report smoking behavior** | **Intentions** | **Knowledge** | **Attitudes** | **Objectively measured smoking behavior** |
| **(1)** | 1.00 |  |  |  |  |  |  |  |  |  |  |  |  |  |  |  |  |
| **(2)** | 0.76*** | 1.00 |  |  |  |  |  |  |  |  |  |  |  |  |  |  |  |
| **(3)** | 0.75*** | 0.73*** | 1.00 |  |  |  |  |  |  |  |  |  |  |  |  |  |  |
| **(4)** | 0.25*** | 0.28*** | 0.24*** | 1.00 |  |  |  |  |  |  |  |  |  |  |  |  |  |
| **(5)** | 0.24*** | 0.23*** | 0.24*** | 0.61*** | 1.00 |  |  |  |  |  |  |  |  |  |  |  |  |
| **(6)** | -0.007 | -0.04 | 0.01 | 0.06** | 0.10*** | 1.00 |  |  |  |  |  |  |  |  |  |  |  |
| **(7)** | -0.16*** | -0.18*** | -0.11*** | 0.14*** | 0.11*** | 0.28*** | 1.00 |  |  |  |  |  |  |  |  |  |  |
| **(8)** | -0.04 | -0.06* | -0.09*** | -0.02 | -0.06** | -0.37*** | -0.08** | 1.00 |  |  |  |  |  |  |  |  |  |
| **(9)** | 0.22*** | 0.21*** | 0.21*** | 0.08** | 0.16*** | -0.14*** | -0.18*** | 0.29*** | 1.00 |  |  |  |  |  |  |  |  |
| **(10)** | -0.20*** | -0.19*** | -0.19*** | -0.05* | -0.05* | 0.01 | 0.14*** | 0.03 | -0.08*** | 1.00 |  |  |  |  |  |  |  |
| **(11)** | -0.04 | -0.07** | -0.02 | 0.002 | 0.04 | 0.10** | 0.10*** | -0.09*** | -0.02 | 0.22*** | 1.00 |  |  |  |  |  |  |
| **(12)** | 0.02 | 0.05 | 0.05* | 0.03 | 0.03 | -0.04 | -0.01 | -0.02 | 0.02 | -0.04 | -0.02 | 1.00 |  |  |  |  |  |
| **(13)** | 0.37*** | 0.39*** | 0.40*** | 0.17*** | 0.16*** | 0.07** | -0.04 | -0.07** | 0.11*** | -0.21*** | -0.07** | 0.04 | 1.00 |  |  |  |  |
| **(14)** | 0.44*** | 0.44*** | 0.42*** | 0.20*** | 0.21*** | 0.03 | -0.07** | -0.008 | 0.20*** | -0.16*** | -0.02 | 0.04 | 0.39*** | 1.00 |  |  |  |
| **(15)** | 0.09*** | 0.12*** | 0.12*** | 0.22*** | 0.25*** | 0.21*** | 0.08*** | -0.18*** | 0.08*** | -0.02 | 0.10*** | -0.04 | 0.09*** | 0.14*** | 1.00 |  |  |
| **(16)** | 0.38*** | 0.42*** | 0.35*** | 0.25*** | 0.26*** | -0.04 | -0.20*** | -0.001 | 0.18*** | -0.15*** | -0.06** | 0.08*** | 0.24*** | 0.31*** | 0.19*** | 1.00 |  |
| **(17)** | -0.14*** | -0.10*** | -0.14*** | -0.008 | -0.13*** | -0.15*** | 0.03 | 0.20*** | -0.10*** | 0.11*** | -0.10*** | 0.009 | -0.11*** | -0.08*** | -0.18*** | -0.08** | 1.00 |

Opp. = opportunity; PBC = perceived behavioral control; ***p<0.01; **p<0.05; *p<0.10.

## Supplementary Table S7. Model fit statistics for longitudinal measurement invariance confirmatory factor analysis models, including data from MECHANISMS schools.

| **Outcome^a^** | **Model fit statistic^b^** | **Robust/ standard^c^** | **Configural^d^** | **Weak/Metric** | **Strong/Scalar** | **Strict/Residual** | **Measurement invariance present?** | **Decision^d^** |
| --- | --- | --- | --- | --- | --- | --- | --- | --- |
| Attitudes (AT1 to AT12, excluding AT2)^e^. | Observations | S/R | 1318 | 1318 | 1318 | 1318 | Strict/Residual. | Run subsequent SEMs with strict/ residual MI constraints included. |
|  | No. of estimated parameters | S/R | 81 | 70 | 59 | 48 |  |  |
|  | χ^2^, df, p-value | S | 764.26, df=194, p<0.0001 | 776.18, df=205, p<0.0001 | 830.49, df=216, p<0.0001 | 884.31, df=227, p<0.0001 |  |  |
|  |  | R | 626.85, df=194, p<0.0001 | 639.19, df=205, p<0.0001 | 690.51, df=216, p<0.0001 | 718.03, df=227, p<0.0001 |  |  |
|  | Δ χ^2^, df, p-value^g^ | R | N/A. | **10.56, df=11, p=0.48** | 55.06, df=11, p<0.0001 | 29.93, df=11, p=0.002 |  |  |
|  | CFI | S | 0.933 | 0.933 | 0.928 | 0.923 |  |  |
|  |  | R | 0.937 | 0.937 | 0.932 | 0.928 |  |  |
|  | Δ CFI^h^ | R | N/A. | 0.000029 | -0.005154 | **-0.003867** |  |  |
|  | TLI | S | 0.921 | 0.925 | 0.923 | 0.922 |  |  |
|  |  | R | 0.926 | 0.930 | 0.928 | 0.927 |  |  |
|  | Δ TLI^h^ | R | N/A. | 0.004027 | -0.001926 | **-0.000429** |  |  |
|  | RMSEA (90% CI) | S | 0.047 (0.044, 0.051) | 0.046 (0.043, 0.049) | 0.046 (0.043, 0.050) | 0.047 (0.044, 0.050) |  |  |
|  |  | R | 0.047 (0.043, 0.051) | 0.046 (0.042, 0.050) | 0.047 (0.043, 0.051) | 0.047 (0.043, 0.051) |  |  |
|  | Δ RMSEA^h^ | R | N/A. | -0.001295 | 0.000624 | **0.000138** |  |  |
|  | SRMR | S/R | 0.041 | 0.045 | 0.046 | 0.047 |  |  |
|  | Δ SRMR^h^ | S/R | N/A. | 0.003641 | 0.001073 | **0.001096** |  |  |
|  | AIC | S/R | 75732.040 | 75721.956 | 75754.270 | 75786.087 |  |  |
|  | Δ AIC^h^ | S/R | N/A. | **-10.084** | 32.313 | 31.818 |  |  |
|  | BIC | S/R | 76151.933 | 76084.827 | 76060.118 | 76034.913 |  |  |
|  | Δ BIC^h^ | S/R | N/A. | -67.106 | -24.709 | **-25.205** |  |  |
|  | Adjusted BIC | S/R | 75894.634 | 75862.470 | 75872.702 | 75882.439 |  |  |
|  | Δ Adjusted BIC^h^ | S/R | N/A. | **-32.164** | 10.233 | 9.737 |  |  |
|  | Baseline factor loadings (unstandardized)^i^ | S/R | 0.500 to 0.731 | 0.535 to 0.739 | 0.534 to 0.737 | 0.534 to 0.738 |  |  |
|  | FU factor loadings (unstandardized)^i^ | S/R | 0.536 to 0.749 | 0.535 to 0.739 | 0.534 to 0.737 | 0.534 to 0.738 |  |  |
|  | Baseline factor loadings (standardized)^j^ | S/R | 0.360 to 0.685 | 0.395 to 0.707 | 0.396 to 0.706 | 0.404 to 0.697 |  |  |
|  | FU Factor loadings (standardized)^j^ | S/R | 0.454 to 0.716 | 0.423 to 0.701 | 0.423 to 0.701 | 0.404 to 0.697 |  |  |
|  | Additional correlation paths (baseline and FU)^k^ | S/R | AT4~~AT6, AT7~~AT8, AT11~~AT12 | | | |  |  |
| Experiment injunctive smoking/ vaping norms (P2S2 to P2S9). | Observations | S/R | 1329 | 1329 | 1329 | 1329 | Strict/Residual. | Run subsequent SEMs with strict/ residual MI constraints included. |
|  | No. of estimated parameters | S/R | 60 | 52 | 44 | 36 |  |  |
|  | χ^2^, df, p-value | S | 378.18, df=92, p<0.0001 | 397.07, df=100, p<0.0001 | 459.07, df=108, p<0.0001 | 489.32, df=116, p<0.0001 |  |  |
|  |  | R | 267.59, df=92, p<0.0001 | 276.97, df=100, p<0.0001 | 326.58, df=108, p<0.0001 | 328.26, df=116, p<0.0001 |  |  |
|  | Δ χ^2^, df, p-value^g^ | R | N/A. | **11.33, df=8, p=0.18** | 58.65, df=8, p<0.0001 | 11.47, df=8, p=0.18 |  |  |
|  | CFI | S | 0.952 | 0.950 | 0.941 | 0.938 |  |  |
|  |  | R | 0.959 | 0.957 | 0.948 | 0.946 |  |  |
|  | Δ CFI^h^ | R | N/A. | -0.001129 | -0.009317 | **-0.001642** |  |  |
|  | TLI | S | 0.938 | 0.940 | 0.935 | 0.936 |  |  |
|  |  | R | 0.946 | 0.949 | 0.942 | 0.945 |  |  |
|  | Δ TLI^h^ | R | N/A. | 0.002975 | -0.006564 | **0.002281** |  |  |
|  | RMSEA (90% CI) | S | 0.048 (0.043, 0.053) | 0.047 (0.042, 0.052) | 0.049 (0.045, 0.054) | 0.049 (0.045, 0.054) |  |  |
|  |  | R | 0.047 (0.040, 0.053) | 0.045 (0.039, 0.052) | 0.048 (0.042, 0.054) | 0.047 (0.041, 0.053) |  |  |
|  | Δ RMSEA^h^ | R | N/A. | -0.001298 | 0.002818 | **-0.000960** |  |  |
|  | SRMR | S/R | 0.038 | 0.044 | 0.048 | 0.049 |  |  |
|  | Δ SRMR^h^ | S/R | N/A. | 0.006247 | 0.004369 | **0.000555** |  |  |
|  | AIC | S/R | 11684.259 | 11687.151 | 11733.157 | 11747.402 |  |  |
|  | Δ AIC^h^ | S/R | N/A. | **2.891** | 46.007 | 14.245 |  |  |
|  | BIC | S/R | 11995.790 | 11957.144 | 11961.613 | 11934.321 |  |  |
|  | Δ BIC^h^ | S/R | N/A. | -38.646 | 4.469 | **-27.293** |  |  |
|  | Adjusted BIC | S/R | 11805.197 | 11791.963 | 11821.845 | 11819.965 |  |  |
|  | Δ Adjusted BIC^h^ | S/R | N/A. | **-13.234** | 29.882 | -1.880 |  |  |
|  | Baseline factor loadings (unstandardized)^i^ | S/R | 0.086 to 0.319 | 0.105 to 0.320 | 0.107 to 0.321 | 0.108 to 0.320 |  |  |
|  | FU factor loadings (unstandardized)^i^ | S/R | 0.126 to 0.323 | 0.105 to 0.320 | 0.107 to 0.321 | 0.108 to 0.320 |  |  |
|  | Baseline factor loadings (standardized)^j^ | S/R | 0.309 to 0.740 | 0.370 to 0.740 | 0.374 to 0.740 | 0.369 to 0.750 |  |  |
|  | FU Factor loadings (standardized)^j^ | S/R | 0.415 to 0.767 | 0.355 to 0.765 | 0.359 to 0.766 | 0.369 to 0.750 |  |  |
|  | Additional correlation paths (baseline and FU)^k^ | S/R | P2S2~~P2S3, P2S2~~P2S4, P2S7~~P2S8 | | | |  |  |
| Experiment injunctive smoking norms (P2S2 to P2S6 and P2S9). | Observations | S/R | 1329 | 1329 | 1329 | 1329 | Weak/Metric. | Strong/scalar MI not supported for CFI and TLI. Examine partial strong/scalar MI. |
|  | No. of estimated parameters | S/R | 45 | 39 | 33 | 27 |  |  |
|  | χ^2^, df, p-value | S | 134.77, df=45, p<0.0001 | 153.23, df=51, p<0.0001 | 216.19, df=57, p<0.0001 | 233.58, df=63, p<0.0001 |  |  |
|  |  | R | 93.91, df=45, p<0.0001 | 101.12, df=51, p<0.0001 | 147.08, df=57, p<0.0001 | 145.07, df=63, p<0.0001 |  |  |
|  | Δ χ^2^, df, p-value^g^ | R | N/A. | **8.72, df=6, p=0.19** | 58.14, df=6, p<0.0001 | 5.91, df=6, p=0.43 |  |  |
|  | CFI | S | 0.971 | 0.967 | 0.948 | 0.944 |  |  |
|  |  | R | 0.977 | 0.975 | 0.956 | 0.956 |  |  |
|  | Δ CFI^h^ | R | N/A. | **-0.002096** | -0.019050 | 0.000063 |  |  |
|  | TLI | S | 0.957 | 0.957 | 0.940 | 0.941 |  |  |
|  |  | R | 0.967 | 0.968 | 0.949 | 0.954 |  |  |
|  | Δ TLI^h^ | R | N/A. | **0.001191** | -0.018690 | 0.004893 |  |  |
|  | RMSEA (90% CI) | S | 0.039 (0.031, 0.046) | 0.039 (0.032, 0.046) | 0.046 (0.039, 0.052) | 0.045 (0.039, 0.051) |  |  |
|  |  | R | 0.035 (0.025, 0.046) | 0.034 (0.024, 0.045) | 0.043 (0.035, 0.052) | 0.041 (0.032, 0.050) |  |  |
|  | Δ RMSEA^h^ | R | N/A. | -0.000636 | 0.008923 | **-0.002149** |  |  |
|  | SRMR | S/R | 0.029 | 0.039 | 0.047 | 0.048 |  |  |
|  | Δ SRMR^h^ | S/R | N/A. | 0.010453 | 0.007321 | **0.001074** |  |  |
|  | AIC | S/R | 8886.924 | 8893.388 | 8944.345 | 8949.734 |  |  |
|  | Δ AIC^h^ | S/R | **N/A.** | 6.464 | 50.957 | 5.389 |  |  |
|  | BIC | S/R | 9120.572 | 9095.883 | 9115.687 | 9089.923 |  |  |
|  | Δ BIC^h^ | S/R | N/A. | -24.689 | 19.804 | **-25.764** |  |  |
|  | Adjusted BIC | S/R | 8977.627 | 8971.997 | 9010.861 | 9004.156 |  |  |
|  | Δ Adjusted BIC^h^ | S/R | N/A. | **-5.630** | 38.863 | -6.705 |  |  |
|  | Baseline factor loadings (unstandardized)^i^ | S/R | 0.114 to 0.241 | 0.137 to 0.248 | 0.138 to 0.250 | 0.139 to 0.251 |  |  |
|  | FU factor loadings (unstandardized)^i^ | S/R | 0.162 to 0.255 | 0.137 to 0.248 | 0.138 to 0.250 | 0.139 to 0.251 |  |  |
|  | Baseline factor loadings (standardized)^j^ | S/R | 0.410 to 0.581 | 0.475 to 0.594 | 0.478 to 0.597 | 0.471 to 0.602 |  |  |
|  | FU Factor loadings (standardized)^j^ | S/R | 0.516 to 0.610 | 0.458 to 0.601 | 0.461 to 0.604 | 0.471 to 0.602 |  |  |
|  | Additional correlation paths (baseline and FU)^k^ | S/R | P2S2~~P2S3, P2S2~~P2S4 | | | |  |  |
| Experiment injunctive vaping norms (P2S7 to P2S8)^f^. | Observations | S/R | 1329 | 1329 | 1329 | 1329 | Strict/Residual. | Run subsequent SEMs with strict/ residual MI constraints included. Strong/scalar MI not supported for RMSEA (no improvement when freeing either of the intercepts). Accept strict/residual model because the values of CFI, TLI, RMSEA, and SRMR are all acceptable. Strict/ residual MI is indicated for most of the other indices. The RMSEA also decreases between the strong/scalar and strict/residual models. |
|  | No. of estimated parameters | S/R | 13 | 12 | 10 | 8 |  |  |
|  | χ^2^, df, p-value | S | 1.51, df=1, p=0.22 | 2.39, df=2, p=0.30 | 12.73, df=4, p=0.01 | 14.47, df=6, p=0.03 |  |  |
|  |  | R | 1.36, df=1, p=0.24 | 2.04, df=2, p=0.36 | 11.60, df=4, p=0.02 | 11.60, df=6, p=0.07 |  |  |
|  | Δ χ^2^, df, p-value^g^ | R | N/A. | **0.71, df=1, p=0.40** | 10.13, df=2, p=0.006 | 1.12, df=2, p=0.57 |  |  |
|  | CFI | S | 1.000 | 1.000 | 0.994 | 0.994 |  |  |
|  |  | R | 1.000 | 1.000 | 0.994 | 0.995 |  |  |
|  | Δ CFI^h^ | R | N/A. | 0.000275 | -0.006001 | **0.000960** |  |  |
|  | TLI | S | 0.998 | 0.999 | 0.991 | 0.994 |  |  |
|  |  | R | 0.998 | 1.000 | 0.991 | 0.995 |  |  |
|  | Δ TLI^h^ | R | N/A. | 0.001659 | -0.009011 | **0.003961** |  |  |
|  | RMSEA (90% CI) | S | 0.020 (0.000, 0.079) | 0.012 (0.000, 0.057) | 0.041 (0.014, 0.070) | 0.033 (0.011, 0.054) |  |  |
|  |  | R | 0.018 (0.000, 0.087) | 0.000 (0.000, 0.062) | 0.041 (0.017, 0.067) | 0.031 (0.000, 0.057) |  |  |
|  | Δ RMSEA^h^ | R | N/A. | **-0.017660** | 0.041255 | -0.010383 |  |  |
|  | SRMR | S/R | 0.008 | 0.014 | 0.027 | 0.029 |  |  |
|  | Δ SRMR^h^ | S/R | N/A. | 0.005881 | 0.012370 | **0.002715** |  |  |
|  | AIC | S/R | 4146.218 | 4145.099 | 4151.441 | 4149.175 |  |  |
|  | Δ AIC^h^ | S/R | N/A. | **-1.119** | 6.342 | -2.266 |  |  |
|  | BIC | S/R | 4213.717 | 4207.405 | 4203.363 | 4190.713 |  |  |
|  | Δ BIC^h^ | S/R | N/A. | -6.311 | -4.042 | **-12.651** |  |  |
|  | Adjusted BIC | S/R | 4172.421 | 4169.287 | 4171.598 | 4165.300 |  |  |
|  | Δ Adjusted BIC^h^ | S/R | N/A. | -3.135 | 2.311 | **-6.298** |  |  |
|  | Baseline factor loadings (unstandardized)^i^ | S/R | 0.328 to 0.328 | 0.323 to 0.323 | 0.323 to 0.323 | 0.323 to 0.323 |  |  |
|  | FU factor loadings (unstandardized)^i^ | S/R | 0.317 to 0.317 | 0.323 to 0.323 | 0.323 to 0.323 | 0.323 to 0.323 |  |  |
|  | Baseline factor loadings (standardized)^j^ | S/R | 0.762 to 0.781 | 0.755 to 0.774 | 0.756 to 0.775 | 0.757 to 0.783 |  |  |
|  | FU Factor loadings (standardized)^j^ | S/R | 0.751 to 0.785 | 0.757 to 0.792 | 0.758 to 0.792 | 0.757 to 0.783 |  |  |
|  | Additional correlation paths (baseline and FU)^k^ | S/R | N/A. | | | |  |  |
| Experiment descriptive smoking/ vaping norms (P3Q1 to P3Q2)^f^. | Observations | S/R | 1328 | 1328 | 1328 | 1328 | Weak/Metric. | Run subsequent SEMs with weak/metric MI constraints included. Strong/scalar MI not supported based on all indices (no improvement when freeing either of the intercepts). Weak/metric MI not supported for RMSEA (no improvement when freeing one of the four factor loadings). Accept weak/metric model because the values of CFI, TLI, RMSEA, and SRMR are all acceptable. Weak/metric MI is indicated for most of the other indices. |
|  | No. of estimated parameters | S/R | 13 | 12 | 10 | 8 |  |  |
|  | χ^2^, df, p-value | S | 0.01, df=1, p=0.91 | 4.49, df=2, p=0.11 | 99.72, df=4, p<0.0001 | 111.93, df=6, p<0.0001 |  |  |
|  |  | R | 0.01, df=1, p=0.92 | 4.02, df=2, p=0.13 | 90.77, df=4, p<0.0001 | 85.04, df=6, p<0.0001 |  |  |
|  | Δ χ^2^, df, p-value^g^ | R | **N/A.** | 4.41, df=1, p=0.04 | 88.32, df=2, p<0.0001 | 6.97, df=2, p=0.03 |  |  |
|  | CFI | S | 1.000 | 0.999 | 0.959 | 0.955 |  |  |
|  |  | R | 1.000 | 0.999 | 0.959 | 0.955 |  |  |
|  | Δ CFI^h^ | R | N/A. | **-0.000971** | -0.040408 | -0.003619 |  |  |
|  | TLI | S | 1.003 | 0.997 | 0.939 | 0.955 |  |  |
|  |  | R | 1.003 | 0.997 | 0.938 | 0.955 |  |  |
|  | Δ TLI^h^ | R | N/A. | **-0.006106** | -0.059155 | 0.017070 |  |  |
|  | RMSEA (90% CI) | S | 0.000 (0.000, 0.029) | 0.031 (0.000, 0.069) | 0.134 (0.112, 0.158) | 0.115 (0.097, 0.134) |  |  |
|  |  | R | 0.000 (0.000, 0.031) | 0.030 (0.000, 0.073) | 0.140 (0.116, 0.164) | 0.119 (0.098, 0.141) |  |  |
|  | Δ RMSEA^h^ | R | **N/A.** | 0.030223 | 0.109298 | -0.020726 |  |  |
|  | SRMR | S/R | 0.001 | 0.030 | 0.081 | 0.087 |  |  |
|  | Δ SRMR^h^ | S/R | N/A. | **0.029049** | 0.051445 | 0.005444 |  |  |
|  | AIC | S/R | 5650.580 | 5653.062 | 5744.284 | 5752.501 |  |  |
|  | Δ AIC^h^ | S/R | N/A. | **2.482** | 91.222 | 8.218 |  |  |
|  | BIC | S/R | 5718.068 | 5715.359 | 5796.198 | 5794.033 |  |  |
|  | Δ BIC^h^ | S/R | N/A. | **-2.709** | 80.839 | -2.165 |  |  |
|  | Adjusted BIC | S/R | 5676.773 | 5677.240 | 5764.432 | 5768.620 |  |  |
|  | Δ Adjusted BIC^h^ | S/R | N/A. | **0.467** | 87.192 | 4.188 |  |  |
|  | Baseline factor loadings (unstandardized)^i^ | S/R | 0.446 to 0.446 | 0.460 to 0.460 | 0.466 to 0.466 | 0.466 to 0.466 |  |  |
|  | FU factor loadings (unstandardized)^i^ | S/R | 0.475 to 0.475 | 0.460 to 0.460 | 0.466 to 0.466 | 0.466 to 0.466 |  |  |
|  | Baseline factor loadings (standardized)^j^ | S/R | 0.811 to 0.916 | 0.818 to 0.925 | 0.821 to 0.928 | 0.825 to 0.905 |  |  |
|  | FU Factor loadings (standardized)^j^ | S/R | 0.832 to 0.891 | 0.823 to 0.883 | 0.827 to 0.885 | 0.825 to 0.905 |  |  |
|  | Additional correlation paths (baseline and FU)^k^ | S/R | N/A. | | | |  |  |
| Self-report injunctive smoking norms (IN1 to IN7). | Observations | S/R | 1318 | 1318 | 1318 | 1318 | Strict/Residual. | Run subsequent SEMs with strict/ residual MI constraints included. |
|  | No. of estimated parameters | S/R | 53 | 46 | 39 | 32 |  |  |
|  | χ^2^, df, p-value | S | 333.39, df=66, p<0.0001 | 362.40, df=73, p<0.0001 | 376.76, df=80, p<0.0001 | 403.39, df=87, p<0.0001 |  |  |
|  |  | R | 249.26, df=66, p<0.0001 | 250.26, df=73, p<0.0001 | 267.90, df=80, p<0.0001 | 261.16, df=87, p<0.0001 |  |  |
|  | Δ χ^2^, df, p-value^g^ | R | N/A. | **11.65, df=7, p=0.11** | 14.79, df=7, p=0.04 | 8.52, df=7, p=0.29 |  |  |
|  | CFI | S | 0.954 | 0.950 | 0.949 | 0.945 |  |  |
|  |  | R | 0.958 | 0.956 | 0.954 | 0.953 |  |  |
|  | Δ CFI^h^ | R | N/A. | -0.002269 | -0.001267 | **-0.000844** |  |  |
|  | TLI | S | 0.936 | 0.938 | 0.942 | 0.943 |  |  |
|  |  | R | 0.942 | 0.945 | 0.948 | 0.951 |  |  |
|  | Δ TLI^h^ | R | N/A. | 0.002746 | 0.003406 | **0.003300** |  |  |
|  | RMSEA (90% CI) | S | 0.055 (0.050, 0.061) | 0.055 (0.049, 0.061) | 0.053 (0.048, 0.059) | 0.053 (0.047, 0.058) |  |  |
|  |  | R | 0.055 (0.048, 0.063) | 0.054 (0.046, 0.061) | 0.052 (0.045, 0.059) | 0.050 (0.043, 0.058) |  |  |
|  | Δ RMSEA^h^ | R | N/A. | -0.001317 | -0.001680 | **-0.001681** |  |  |
|  | SRMR | S/R | 0.043 | 0.053 | 0.054 | 0.055 |  |  |
|  | Δ SRMR^h^ | S/R | N/A. | 0.010198 | 0.000411 | **0.001629** |  |  |
|  | AIC | S/R | 33222.114 | 33237.125 | 33237.480 | 33250.116 |  |  |
|  | Δ AIC^h^ | S/R | **N/A.** | 15.011 | 0.356 | 12.636 |  |  |
|  | BIC | S/R | 33496.859 | 33475.583 | 33439.651 | 33416.000 |  |  |
|  | Δ BIC^h^ | S/R | N/A. | -21.276 | -35.932 | **-23.651** |  |  |
|  | Adjusted BIC | S/R | 33328.502 | 33329.462 | 33315.766 | 33314.351 |  |  |
|  | Δ Adjusted BIC^h^ | S/R | N/A. | 0.960 | -13.696 | **-1.415** |  |  |
|  | Baseline factor loadings (unstandardized)^i^ | S/R | 0.203 to 0.566 | 0.231 to 0.576 | 0.231 to 0.576 | 0.232 to 0.577 |  |  |
|  | FU factor loadings (unstandardized)^i^ | S/R | 0.266 to 0.579 | 0.231 to 0.576 | 0.231 to 0.576 | 0.232 to 0.577 |  |  |
|  | Baseline factor loadings (standardized)^j^ | S/R | 0.441 to 0.619 | 0.443 to 0.633 | 0.442 to 0.633 | 0.447 to 0.640 |  |  |
|  | FU Factor loadings (standardized)^j^ | S/R | 0.459 to 0.653 | 0.454 to 0.645 | 0.453 to 0.646 | 0.447 to 0.640 |  |  |
|  | Additional correlation paths (baseline and FU)^k^ | S/R | IN2 ~~ IN3, IN4 ~~ IN5, IN6 ~~ IN7 | | | |  |  |
| Self-report descriptive smoking norms scale 1 (DN1.1 to DN1.5). | Observations | S/R | 1318 | 1318 | 1318 | 1318 | Strict/Residual. | Run subsequent SEMs with strict/ residual MI constraints included. |
|  | No. of estimated parameters | S/R | 36 | 31 | 26 | 21 |  |  |
|  | χ^2^, df, p-value | S | 143.16, df=29, p<0.0001 | 166.15, df=34, p<0.0001 | 185.83, df=39, p<0.0001 | 216.30, df=44, p<0.0001 |  |  |
|  |  | R | 68.73, df=29, p<0.0001 | 75.17, df=34, p=0.0001 | 90.81, df=39, p<0.0001 | 96.38, df=44, p<0.0001 |  |  |
|  | Δ χ^2^, df, p-value^g^ | R | N/A. | **7.79, df=5, p=0.17** | 21.16, df=5, p=0.001 | 8.05, df=5, p=0.15 |  |  |
|  | CFI | S | 0.978 | 0.975 | 0.972 | 0.967 |  |  |
|  |  | R | 0.986 | 0.985 | 0.982 | 0.980 |  |  |
|  | Δ CFI^h^ | R | N/A. | -0.001592 | -0.002830 | **-0.002229** |  |  |
|  | TLI | S | 0.966 | 0.967 | 0.968 | 0.966 |  |  |
|  |  | R | 0.979 | 0.980 | 0.979 | 0.979 |  |  |
|  | Δ TLI^h^ | R | N/A. | 0.000984 | -0.000696 | **0.000076** |  |  |
|  | RMSEA (90% CI) | S | 0.055 (0.046, 0.064) | 0.054 (0.046, 0.063) | 0.053 (0.046, 0.061) | 0.055 (0.047, 0.062) |  |  |
|  |  | R | 0.046 (0.029, 0.062) | 0.045 (0.029, 0.061) | 0.046 (0.032, 0.060) | 0.046 (0.032, 0.059) |  |  |
|  | Δ RMSEA^h^ | R | N/A. | -0.001089 | 0.000772 | **-0.000084** |  |  |
|  | SRMR | S/R | 0.042 | 0.045 | 0.046 | 0.049 |  |  |
|  | Δ SRMR^h^ | S/R | N/A. | 0.003430 | 0.000722 | **0.003333** |  |  |
|  | AIC | S/R | 27952.286 | 27965.270 | 27974.953 | 27995.425 |  |  |
|  | Δ AIC^h^ | S/R | **N/A.** | 12.985 | 9.683 | 20.472 |  |  |
|  | BIC | S/R | 28138.905 | 28125.970 | 28109.734 | 28104.286 |  |  |
|  | Δ BIC^h^ | S/R | N/A. | -12.935 | -16.237 | **-5.447** |  |  |
|  | Adjusted BIC | S/R | 28024.550 | 28027.498 | 28027.144 | 28037.579 |  |  |
|  | Δ Adjusted BIC^h^ | S/R | N/A. | 2.948 | **-0.354** | 10.435 |  |  |
|  | Baseline factor loadings (unstandardized)^i^ | S/R | 0.259 to 0.704 | 0.289 to 0.692 | 0.288 to 0.692 | 0.292 to 0.695 |  |  |
|  | FU factor loadings (unstandardized)^i^ | S/R | 0.323 to 0.686 | 0.289 to 0.692 | 0.288 to 0.692 | 0.292 to 0.695 |  |  |
|  | Baseline factor loadings (standardized)^j^ | S/R | 0.352 to 0.588 | 0.387 to 0.577 | 0.386 to 0.578 | 0.380 to 0.590 |  |  |
|  | FU Factor loadings (standardized)^j^ | S/R | 0.405 to 0.591 | 0.359 to 0.601 | 0.357 to 0.601 | 0.380 to 0.590 |  |  |
|  | Additional correlation paths (baseline and FU)^k^ | S/R | N/A. | | | |  |  |
| Self-report descriptive smoking norms scale 2 (DN2.1 to DN2.3). | Observations | S/R | 1318 | 1318 | 1318 | 1318 | Strict/Residual. | Run subsequent SEMs with strict/ residual MI constraints included. |
|  | No. of estimated parameters | S/R | 22 | 19 | 16 | 13 |  |  |
|  | χ^2^, df, p-value | S | 15.84, df=5, p=0.007 | 35.20, df=8, p<0.0001 | 47.67, df=11, p<0.0001 | 67.93, df=14, p<0.0001 |  |  |
|  |  | R | 9.64, df=5, p=0.09 | 17.59, df=8, p=0.02 | 27.65, df=11, p=0.004 | 30.83, df=14, p=0.006 |  |  |
|  | Δ χ^2^, df, p-value^g^ | R | N/A. | **7.46, df=3, p=0.06** | 12.65, df=3, p=0.005 | 5.12, df=3, p=0.16 |  |  |
|  | CFI | S | 0.994 | 0.984 | 0.979 | 0.969 |  |  |
|  |  | R | 0.996 | 0.989 | 0.984 | 0.979 |  |  |
|  | Δ CFI^h^ | R | N/A. | -0.006566 | -0.005594 | **-0.004742** |  |  |
|  | TLI | S | 0.981 | 0.971 | 0.971 | 0.967 |  |  |
|  |  | R | 0.987 | 0.980 | 0.978 | 0.977 |  |  |
|  | Δ TLI^h^ | R | N/A. | -0.007467 | -0.002069 | **-0.000269** |  |  |
|  | RMSEA (90% CI) | S | 0.041 (0.019, 0.064) | 0.051 (0.034, 0.069) | 0.050 (0.036, 0.065) | 0.054 (0.042, 0.067) |  |  |
|  |  | R | 0.036 (0.000, 0.071) | 0.045 (0.014, 0.074) | 0.047 (0.025, 0.070) | 0.048 (0.025, 0.070) |  |  |
|  | Δ RMSEA^h^ | R | N/A. | 0.009198 | 0.002233 | **0.000283** |  |  |
|  | SRMR | S/R | 0.019 | 0.041 | 0.044 | 0.059 |  |  |
|  | Δ SRMR^h^ | S/R | N/A. | 0.022192 | 0.003198 | **0.014776** |  |  |
|  | AIC | S/R | 15312.791 | 15326.153 | 15332.622 | 15346.879 |  |  |
|  | Δ AIC^h^ | S/R | **N/A.** | 13.362 | 6.469 | 14.256 |  |  |
|  | BIC | S/R | 15426.837 | 15424.647 | 15415.564 | 15414.269 |  |  |
|  | Δ BIC^h^ | S/R | N/A. | -2.190 | -9.083 | **-1.295** |  |  |
|  | Adjusted BIC | S/R | 15356.953 | 15364.293 | 15364.740 | 15372.974 |  |  |
|  | Δ Adjusted BIC^h^ | S/R | **N/A.** | 7.340 | 0.447 | 8.234 |  |  |
|  | Baseline factor loadings (unstandardized)^i^ | S/R | 0.327 to 0.507 | 0.357 to 0.543 | 0.355 to 0.543 | 0.354 to 0.548 |  |  |
|  | FU factor loadings (unstandardized)^i^ | S/R | 0.391 to 0.576 | 0.357 to 0.543 | 0.355 to 0.543 | 0.354 to 0.548 |  |  |
|  | Baseline factor loadings (standardized)^j^ | S/R | 0.328 to 0.745 | 0.354 to 0.777 | 0.352 to 0.777 | 0.356 to 0.758 |  |  |
|  | FU Factor loadings (standardized)^j^ | S/R | 0.395 to 0.754 | 0.364 to 0.730 | 0.362 to 0.730 | 0.356 to 0.758 |  |  |
|  | Additional correlation paths (baseline and FU)^k^ | S/R | N/A. | | | |  |  |
| Self-efficacy (emotional; SEE1 to SEE9). | Observations | S/R | 1319 | 1319 | 1319 | 1319 | Configural. | Weak/metric MI not supported for SRMR. Examine partial weak/metric MI. |
|  | No. of estimated parameters | S/R | 66 | 57 | 48 | 39 |  |  |
|  | χ^2^, df, p-value | S | 813.43, df=123, p<0.0001 | 841.73, df=132, p<0.0001 | 868.02, df=141, p<0.0001 | 904.96, df=150, p<0.0001 |  |  |
|  |  | R | 216.41, df=123, p<0.0001 | 227.59, df=132, p<0.0001 | 246.21, df=141, p<0.0001 | 247.88, df=150, p<0.0001 |  |  |
|  | Δ χ^2^, df, p-value^g^ | R | N/A. | **9.85, df=9, p=0.36** | 26.52, df=9, p=0.002 | 6.58, df=9, p=0.68 |  |  |
|  | CFI | S | 0.974 | 0.974 | 0.973 | 0.972 |  |  |
|  |  | R | 0.987 | 0.987 | 0.986 | 0.987 |  |  |
|  | Δ CFI^h^ | R | N/A. | -0.000131 | -0.000661 | **0.000448** |  |  |
|  | TLI | S | 0.968 | 0.969 | 0.971 | 0.971 |  |  |
|  |  | R | 0.984 | 0.985 | 0.985 | 0.986 |  |  |
|  | Δ TLI^h^ | R | N/A. | 0.000955 | 0.000258 | **0.001358** |  |  |
|  | RMSEA (90% CI) | S | 0.065 (0.061, 0.070) | 0.064 (0.060, 0.068) | 0.063 (0.059, 0.067) | 0.062 (0.058, 0.066) |  |  |
|  |  | R | 0.048 (0.037, 0.059) | 0.047 (0.036, 0.057) | 0.046 (0.036, 0.056) | 0.044 (0.034, 0.054) |  |  |
|  | Δ RMSEA^h^ | R | N/A. | -0.001437 | -0.000396 | **-0.002144** |  |  |
|  | SRMR | S/R | 0.021 | 0.062 | 0.065 | 0.066 |  |  |
|  | Δ SRMR^h^ | S/R | **N/A.** | 0.041224 | 0.003375 | 0.000926 |  |  |
|  | AIC | S/R | 35389.835 | 35400.133 | 35408.422 | 35427.361 |  |  |
|  | Δ AIC^h^ | S/R | **N/A.** | 10.298 | 8.289 | 18.939 |  |  |
|  | BIC | S/R | 35732.020 | 35695.657 | 35657.284 | 35629.561 |  |  |
|  | Δ BIC^h^ | S/R | N/A. | -36.364 | -38.373 | **-27.723** |  |  |
|  | Adjusted BIC | S/R | 35522.369 | 35514.594 | 35504.810 | 35505.676 |  |  |
|  | Δ Adjusted BIC^h^ | S/R | N/A. | -7.775 | -9.784 | **0.866** |  |  |
|  | Baseline factor loadings (unstandardized)^i^ | S/R | 0.776 to 0.846 | 0.807 to 0.898 | 0.808 to 0.899 | 0.807 to 0.898 |  |  |
|  | FU factor loadings (unstandardized)^i^ | S/R | 0.840 to 0.951 | 0.807 to 0.898 | 0.808 to 0.899 | 0.807 to 0.898 |  |  |
|  | Baseline factor loadings (standardized)^j^ | S/R | 0.828 to 0.907 | 0.842 to 0.914 | 0.842 to 0.914 | 0.843 to 0.904 |  |  |
|  | FU Factor loadings (standardized)^j^ | S/R | 0.856 to 0.917 | 0.843 to 0.907 | 0.843 to 0.908 | 0.843 to 0.904 |  |  |
|  | Additional correlation paths (baseline and FU)^k^ | S/R | SEE1~~SEE2, SEE7~~SEE9 | | | |  |  |
| Self-efficacy (friends; SEF1 to SEF9). | Observations | S/R | 1319 | 1319 | 1319 | 1319 | Strict/Residual. | Run subsequent SEMs with strict/ residual MI constraints included. |
|  | No. of estimated parameters | S/R | 68 | 59 | 50 | 41 |  |  |
|  | χ^2^, df, p-value | S | 838.31, df=121, p<0.0001 | 879.84, df=130, p<0.0001 | 905.47, df=139, p<0.0001 | 967.62, df=148, p<0.0001 |  |  |
|  |  | R | 278.22, df=121, p<0.0001 | 290.65, df=130, p<0.0001 | 312.87, df=139, p<0.0001 | 308.67, df=148, p<0.0001 |  |  |
|  | Δ χ^2^, df, p-value^g^ | R | N/A. | **12.92, df=9, p=0.17** | 26.36, df=9, p=0.002 | 9.07, df=9, p=0.43 |  |  |
|  | CFI | S | 0.971 | 0.970 | 0.969 | 0.967 |  |  |
|  |  | R | 0.980 | 0.980 | 0.979 | 0.979 |  |  |
|  | Δ CFI^h^ | R | N/A. | -0.000504 | -0.000758 | **-0.000087** |  |  |
|  | TLI | S | 0.964 | 0.965 | 0.966 | 0.966 |  |  |
|  |  | R | 0.975 | 0.976 | 0.977 | 0.978 |  |  |
|  | Δ TLI^h^ | R | N/A. | 0.001143 | 0.000716 | **0.001322** |  |  |
|  | RMSEA (90% CI) | S | 0.067 (0.063, 0.071) | 0.066 (0.062, 0.070) | 0.065 (0.061, 0.069) | 0.065 (0.061, 0.069) |  |  |
|  |  | R | 0.057 (0.048, 0.067) | 0.056 (0.047, 0.065) | 0.055 (0.047, 0.064) | 0.054 (0.045, 0.062) |  |  |
|  | Δ RMSEA^h^ | R | N/A. | -0.001325 | -0.000846 | **-0.001597** |  |  |
|  | SRMR | S/R | 0.039 | 0.059 | 0.061 | 0.063 |  |  |
|  | Δ SRMR^h^ | S/R | N/A. | 0.019752 | 0.002300 | **0.001735** |  |  |
|  | AIC | S/R | 35070.114 | 35093.646 | 35101.275 | 35145.424 |  |  |
|  | Δ AIC^h^ | S/R | **N/A.** | 23.532 | 7.629 | 44.148 |  |  |
|  | BIC | S/R | 35422.669 | 35399.539 | 35360.507 | 35357.993 |  |  |
|  | Δ BIC^h^ | S/R | N/A. | -23.129 | -39.033 | **-2.513** |  |  |
|  | Adjusted BIC | S/R | 35206.664 | 35212.123 | 35201.680 | 35227.755 |  |  |
|  | Δ Adjusted BIC^h^ | S/R | N/A. | 5.460 | **-10.444** | 26.076 |  |  |
|  | Baseline factor loadings (unstandardized)^i^ | S/R | 0.628 to 0.910 | 0.631 to 0.926 | 0.632 to 0.928 | 0.631 to 0.927 |  |  |
|  | FU factor loadings (unstandardized)^i^ | S/R | 0.633 to 0.944 | 0.631 to 0.926 | 0.632 to 0.928 | 0.631 to 0.927 |  |  |
|  | Baseline factor loadings (standardized)^j^ | S/R | 0.776 to 0.904 | 0.775 to 0.913 | 0.775 to 0.913 | 0.775 to 0.901 |  |  |
|  | FU Factor loadings (standardized)^j^ | S/R | 0.777 to 0.899 | 0.776 to 0.889 | 0.777 to 0.889 | 0.775 to 0.901 |  |  |
|  | Additional correlation paths (baseline and FU)^k^ | S/R | SEF5~~SEF6, SEF5~~SEF7, SEF6~~SEF7, SEF8~~SEF9 | | | |  |  |
| Self-efficacy (opportunity; SEO1 to SEO11). | Observations | S/R | 1319 | 1319 | 1319 | 1319 | Configural. | Weak/metric MI not supported for SRMR. Examine partial weak/metric MI. |
|  | No. of estimated parameters | S/R | 83 | 72 | 61 | 50 |  |  |
|  | χ^2^, df, p-value | S | 1964.63, df=192, p<0.0001 | 2029.63, df=203, p<0.0001 | 2048.42, df=214, p<0.0001 | 2328.72, df=225, p<0.0001 |  |  |
|  |  | R | 338.53, df=192, p<0.0001 | 351.93, df=203, p<0.0001 | 370.98, df=214, p<0.0001 | 394.64, df=225, p<0.0001 |  |  |
|  | Δ χ^2^, df, p-value^g^ | R | N/A. | 12.66, df=11, p=0.32 | **18.98, df=11, p=0.06** | 21.11, df=11, p=0.03 |  |  |
|  | CFI | S | 0.951 | 0.950 | 0.950 | 0.942 |  |  |
|  |  | R | 0.977 | 0.976 | 0.976 | 0.972 |  |  |
|  | Δ CFI^h^ | R | N/A. | -0.000508 | -0.000224 | **-0.003829** |  |  |
|  | TLI | S | 0.942 | 0.943 | 0.946 | 0.941 |  |  |
|  |  | R | 0.972 | 0.973 | 0.974 | 0.971 |  |  |
|  | Δ TLI^h^ | R | N/A. | 0.000941 | 0.001151 | **-0.002663** |  |  |
|  | RMSEA (90% CI) | S | 0.084 (0.080, 0.087) | 0.083 (0.079, 0.086) | 0.081 (0.077, 0.084) | 0.084 (0.081, 0.087) |  |  |
|  |  | R | 0.060 (0.048, 0.071) | 0.059 (0.047, 0.069) | 0.057 (0.047, 0.068) | 0.060 (0.050, 0.070) |  |  |
|  | Δ RMSEA^h^ | R | N/A. | -0.001008 | -0.001257 | **0.002870** |  |  |
|  | SRMR | S/R | 0.042 | 0.100 | 0.102 | 0.104 |  |  |
|  | Δ SRMR^h^ | S/R | **N/A.** | 0.057603 | 0.001922 | 0.002446 |  |  |
|  | AIC | S/R | 24664.894 | 24707.892 | 24704.684 | 24962.989 |  |  |
|  | Δ AIC^h^ | S/R | **N/A.** | 42.998 | -3.208 | 258.304 |  |  |
|  | BIC | S/R | 25095.218 | 25081.186 | 25020.947 | 25222.220 |  |  |
|  | Δ BIC^h^ | S/R | N/A. | -14.033 | **-60.239** | 201.273 |  |  |
|  | Adjusted BIC | S/R | 24831.566 | 24852.475 | 24827.178 | 25063.393 |  |  |
|  | Δ Adjusted BIC^h^ | S/R | N/A. | 20.909 | **-25.297** | 236.215 |  |  |
|  | Baseline factor loadings (unstandardized)^i^ | S/R | 0.536 to 0.647 | 0.599 to 0.698 | 0.600 to 0.699 | 0.603 to 0.699 |  |  |
|  | FU factor loadings (unstandardized)^i^ | S/R | 0.643 to 0.749 | 0.599 to 0.698 | 0.600 to 0.699 | 0.603 to 0.699 |  |  |
|  | Baseline factor loadings (standardized)^j^ | S/R | 0.854 to 0.932 | 0.869 to 0.939 | 0.870 to 0.940 | 0.854 to 0.928 |  |  |
|  | FU Factor loadings (standardized)^j^ | S/R | 0.844 to 0.930 | 0.820 to 0.923 | 0.820 to 0.923 | 0.854 to 0.928 |  |  |
|  | Additional correlation paths (baseline and FU)^k^ | S/R | SEO1~~SEO3, SEO3~~SEO4, SEO4~~SEO11, SEO8~~SEO9, SEO8~~SEO10 | | | |  |  |
| Perceived physical risks (RP1 to RP7). | Observations | S/R | 1319 | 1319 | 1319 | 1319 | Weak/Metric. | Strong/scalar MI not supported for CFI and TLI. Examine partial strong/scalar MI. |
|  | No. of estimated parameters | S/R | 53 | 46 | 39 | 32 |  |  |
|  | χ^2^, df, p-value | S | 500.97, df=66, p<0.0001 | 509.67, df=73, p<0.0001 | 658.53, df=80, p<0.0001 | 692.56, df=87, p<0.0001 |  |  |
|  |  | R | 400.90, df=66, p<0.0001 | 421.53, df=73, p<0.0001 | 554.83, df=80, p<0.0001 | 585.25, df=87, p<0.0001 |  |  |
|  | Δ χ^2^, df, p-value^g^ | R | N/A. | **10.52, df=7, p=0.16** | 155.81, df=7, p<0.0001 | 29.78, df=7, p=0.0001 |  |  |
|  | CFI | S | 0.950 | 0.950 | 0.934 | 0.930 |  |  |
|  |  | R | 0.951 | 0.951 | 0.934 | 0.931 |  |  |
|  | Δ CFI^h^ | R | N/A. | **-0.000320** | -0.016362 | -0.003022 |  |  |
|  | TLI | S | 0.931 | 0.938 | 0.924 | 0.927 |  |  |
|  |  | R | 0.932 | 0.938 | 0.925 | 0.928 |  |  |
|  | Δ TLI^h^ | R | N/A. | **0.006090** | -0.013223 | 0.002858 |  |  |
|  | RMSEA (90% CI) | S | 0.071 (0.065, 0.077) | 0.067 (0.062, 0.073) | 0.074 (0.069, 0.079) | 0.073 (0.068, 0.078) |  |  |
|  |  | R | 0.073 (0.066, 0.080) | 0.069 (0.063, 0.076) | 0.076 (0.070, 0.083) | 0.075 (0.069, 0.081) |  |  |
|  | Δ RMSEA^h^ | R | N/A. | -0.003349 | 0.007086 | **-0.001475** |  |  |
|  | SRMR | S/R | 0.046 | 0.050 | 0.058 | 0.060 |  |  |
|  | Δ SRMR^h^ | S/R | N/A. | 0.003811 | 0.007855 | **0.001890** |  |  |
|  | AIC | S/R | 160957.061 | 160951.757 | 161086.615 | 161106.654 |  |  |
|  | Δ AIC^h^ | S/R | N/A. | **-5.304** | 134.857 | 20.039 |  |  |
|  | BIC | S/R | 161231.847 | 161190.250 | 161288.815 | 161272.562 |  |  |
|  | Δ BIC^h^ | S/R | N/A. | **-41.596** | 98.565 | -16.253 |  |  |
|  | Adjusted BIC | S/R | 161063.490 | 161044.129 | 161164.930 | 161170.913 |  |  |
|  | Δ Adjusted BIC^h^ | S/R | N/A. | **-19.361** | 120.801 | 5.983 |  |  |
|  | Baseline factor loadings (unstandardized)^i^ | S/R | 18.850 to 25.077 | 17.630 to 24.764 | 17.461 to 24.853 | 17.477 to 24.865 |  |  |
|  | FU factor loadings (unstandardized)^i^ | S/R | 16.370 to 24.413 | 17.630 to 24.764 | 17.461 to 24.853 | 17.477 to 24.865 |  |  |
|  | Baseline factor loadings (standardized)^j^ | S/R | 0.614 to 0.771 | 0.588 to 0.767 | 0.582 to 0.770 | 0.588 to 0.777 |  |  |
|  | FU Factor loadings (standardized)^j^ | S/R | 0.571 to 0.778 | 0.599 to 0.782 | 0.594 to 0.784 | 0.588 to 0.777 |  |  |
|  | Additional correlation paths (baseline and FU)^k^ | S/R | RP1~~RP2, RP5~~RP6, RP6~~RP7 | | | |  |  |
| Perceived social risks (RS1 to RS3). | Observations | S/R | 1318 | 1318 | 1318 | 1318 | Weak/Metric. | Strong/scalar MI not supported for CFI, TLI, and RMSEA. Examine partial strong/scalar MI. |
|  | No. of estimated parameters | S/R | 22 | 19 | 16 | 13 |  |  |
|  | χ^2^, df, p-value | S | 8.90, df=5, p=0.11 | 16.83, df=8, p=0.03 | 38.24, df=11, p=0.0001 | 39.92, df=14, p=0.0003 |  |  |
|  |  | R | 7.95, df=5, p=0.16 | 15.51, df=8, p=0.05 | 36.10, df=11, p=0.0002 | 38.48, df=14, p=0.0004 |  |  |
|  | Δ χ^2^, df, p-value^g^ | R | N/A. | **7.71, df=3, p=0.05** | 21.62, df=3, p=0.0001 | 1.76, df=3, p=0.62 |  |  |
|  | CFI | S | 0.998 | 0.995 | 0.985 | 0.985 |  |  |
|  |  | R | 0.998 | 0.995 | 0.985 | 0.986 |  |  |
|  | Δ CFI^h^ | R | N/A. | **-0.002690** | -0.010375 | 0.000628 |  |  |
|  | TLI | S | 0.993 | 0.991 | 0.979 | 0.984 |  |  |
|  |  | R | 0.994 | 0.991 | 0.980 | 0.985 |  |  |
|  | Δ TLI^h^ | R | N/A. | **-0.002835** | -0.011769 | 0.005064 |  |  |
|  | RMSEA (90% CI) | S | 0.024 (0.000, 0.050) | 0.029 (0.008, 0.048) | 0.043 (0.029, 0.059) | 0.037 (0.024, 0.051) |  |  |
|  |  | R | 0.024 (0.000, 0.053) | 0.029 (0.000, 0.051) | 0.045 (0.029, 0.061) | 0.039 (0.024, 0.054) |  |  |
|  | Δ RMSEA^h^ | R | N/A. | **0.005194** | 0.015504 | -0.005903 |  |  |
|  | SRMR | S/R | 0.014 | 0.027 | 0.033 | 0.036 |  |  |
|  | Δ SRMR^h^ | S/R | N/A. | 0.013220 | 0.005358 | **0.003739** |  |  |
|  | AIC | S/R | 71272.422 | 71274.354 | 71289.760 | 71285.443 |  |  |
|  | Δ AIC^h^ | S/R | N/A. | **1.932** | 15.406 | -4.317 |  |  |
|  | BIC | S/R | 71386.467 | 71372.847 | 71372.702 | 71352.834 |  |  |
|  | Δ BIC^h^ | S/R | N/A. | -13.620 | -0.145 | **-19.868** |  |  |
|  | Adjusted BIC | S/R | 71316.583 | 71312.493 | 71321.877 | 71311.539 |  |  |
|  | Δ Adjusted BIC^h^ | S/R | N/A. | -4.090 | 9.384 | **-10.339** |  |  |
|  | Baseline factor loadings (unstandardized)^i^ | S/R | 20.203 to 24.846 | 19.745 to 23.053 | 19.819 to 23.101 | 19.823 to 23.182 |  |  |
|  | FU factor loadings (unstandardized)^i^ | S/R | 19.250 to 21.925 | 19.745 to 23.053 | 19.819 to 23.101 | 19.823 to 23.182 |  |  |
|  | Baseline factor loadings (standardized)^j^ | S/R | 0.568 to 0.754 | 0.560 to 0.716 | 0.561 to 0.716 | 0.562 to 0.727 |  |  |
|  | FU Factor loadings (standardized)^j^ | S/R | 0.552 to 0.694 | 0.561 to 0.734 | 0.562 to 0.734 | 0.562 to 0.727 |  |  |
|  | Additional correlation paths (baseline and FU)^k^ | S/R | N/A. | | | |  |  |
| Perceived addiction risks (RA1 to RA3). | Observations | S/R | 1317 | 1317 | 1317 | 1317 | Weak/Metric. | Strong/scalar MI not supported for CFI. Examine partial strong/scalar MI. |
|  | No. of estimated parameters | S/R | 22 | 19 | 16 | 13 |  |  |
|  | χ^2^, df, p-value | S | 18.87, df=5, p=0.002 | 24.65, df=8, p=0.002 | 42.71, df=11, p<0.0001 | 46.29, df=14, p<0.0001 |  |  |
|  |  | R | 18.36, df=5, p=0.003 | 25.40, df=8, p=0.001 | 43.61, df=11, p<0.0001 | 52.21, df=14, p<0.0001 |  |  |
|  | Δ χ^2^, df, p-value^g^ | R | N/A. | **6.61, df=3, p=0.09** | 18.00, df=3, p=0.0004 | 6.55, df=3, p=0.09 |  |  |
|  | CFI | S | 0.988 | 0.986 | 0.973 | 0.973 |  |  |
|  |  | R | 0.988 | 0.985 | 0.973 | 0.971 |  |  |
|  | Δ CFI^h^ | R | N/A. | **-0.002505** | -0.012618 | -0.001559 |  |  |
|  | TLI | S | 0.965 | 0.974 | 0.963 | 0.971 |  |  |
|  |  | R | 0.964 | 0.973 | 0.963 | 0.969 |  |  |
|  | Δ TLI^h^ | R | N/A. | 0.008960 | -0.009719 | **0.006296** |  |  |
|  | RMSEA (90% CI) | S | 0.046 (0.025, 0.069) | 0.040 (0.023, 0.058) | 0.047 (0.032, 0.062) | 0.042 (0.029, 0.056) |  |  |
|  |  | R | 0.049 (0.027, 0.075) | 0.043 (0.025, 0.062) | 0.050 (0.035, 0.066) | 0.046 (0.033, 0.059) |  |  |
|  | Δ RMSEA^h^ | R | N/A. | -0.006512 | 0.007024 | **-0.004427** |  |  |
|  | SRMR | S/R | 0.025 | 0.029 | 0.036 | 0.038 |  |  |
|  | Δ SRMR^h^ | S/R | N/A. | 0.004057 | 0.006039 | **0.002069** |  |  |
|  | AIC | S/R | 72249.206 | 72248.990 | 72261.045 | 72258.625 |  |  |
|  | Δ AIC^h^ | S/R | N/A. | **-0.216** | 12.056 | -2.421 |  |  |
|  | BIC | S/R | 72363.235 | 72347.469 | 72343.975 | 72326.005 |  |  |
|  | Δ BIC^h^ | S/R | N/A. | -15.766 | -3.494 | **-17.970** |  |  |
|  | Adjusted BIC | S/R | 72293.351 | 72287.115 | 72293.151 | 72284.710 |  |  |
|  | Δ Adjusted BIC^h^ | S/R | N/A. | -6.236 | 6.036 | **-8.440** |  |  |
|  | Baseline factor loadings (unstandardized)^i^ | S/R | 4.806 to 36.128 | 6.264 to 33.331 | 6.384 to 33.423 | 6.294 to 33.820 |  |  |
|  | FU factor loadings (unstandardized)^i^ | S/R | 7.702 to 32.475 | 6.264 to 33.331 | 6.384 to 33.423 | 6.294 to 33.820 |  |  |
|  | Baseline factor loadings (standardized)^j^ | S/R | 0.132 to 0.893 | 0.171 to 0.830 | 0.174 to 0.830 | 0.175 to 0.848 |  |  |
|  | FU Factor loadings (standardized)^j^ | S/R | 0.218 to 0.831 | 0.178 to 0.846 | 0.181 to 0.846 | 0.175 to 0.848 |  |  |
|  | Additional correlation paths (baseline and FU)^k^ | S/R | N/A. | | | |  |  |
| Perceived benefits (BE1 to BE5). | Observations | S/R | 1315 | 1315 | 1315 | 1315 | Strict/Residual. | Run subsequent SEMs with strict/ residual MI constraints included. |
|  | No. of estimated parameters | S/R | 37 | 32 | 27 | 22 |  |  |
|  | χ^2^, df, p-value | S | 78.41, df=28, p<0.0001 | 110.82, df=33, p<0.0001 | 125.81, df=38, p<0.0001 | 132.52, df=43, p<0.0001 |  |  |
|  |  | R | 59.92, df=28, p=0.0004 | 83.62, df=33, p<0.0001 | 98.19, df=38, p<0.0001 | 92.38, df=43, p<0.0001 |  |  |
|  | Δ χ^2^, df, p-value^g^ | R | **N/A.** | 22.83, df=5, p=0.0004 | 15.14, df=5, p=0.01 | 2.58, df=5, p=0.76 |  |  |
|  | CFI | S | 0.991 | 0.986 | 0.984 | 0.983 |  |  |
|  |  | R | 0.992 | 0.988 | 0.986 | 0.987 |  |  |
|  | Δ CFI^h^ | R | N/A. | -0.004647 | -0.001810 | **0.001190** |  |  |
|  | TLI | S | 0.985 | 0.980 | 0.981 | 0.983 |  |  |
|  |  | R | 0.987 | 0.983 | 0.983 | 0.986 |  |  |
|  | Δ TLI^h^ | R | N/A. | -0.004442 | 0.000086 | **0.003206** |  |  |
|  | RMSEA (90% CI) | S | 0.037 (0.027, 0.047) | 0.042 (0.034, 0.051) | 0.042 (0.034, 0.050) | 0.040 (0.032, 0.048) |  |  |
|  |  | R | 0.035 (0.022, 0.048) | 0.041 (0.030, 0.053) | 0.041 (0.031, 0.052) | 0.037 (0.026, 0.048) |  |  |
|  | Δ RMSEA^h^ | R | N/A. | 0.005811 | -0.000104 | **-0.004114** |  |  |
|  | SRMR | S/R | 0.029 | 0.036 | 0.037 | 0.036 |  |  |
|  | Δ SRMR^h^ | S/R | N/A. | 0.007379 | 0.000875 | **-0.000938** |  |  |
|  | AIC | S/R | 110602.186 | 110624.593 | 110629.586 | 110626.297 |  |  |
|  | Δ AIC^h^ | S/R | **N/A.** | 22.406 | 4.993 | -3.289 |  |  |
|  | BIC | S/R | 110793.905 | 110790.404 | 110769.489 | 110740.292 |  |  |
|  | Δ BIC^h^ | S/R | N/A. | -3.502 | -20.915 | **-29.197** |  |  |
|  | Adjusted BIC | S/R | 110676.373 | 110688.754 | 110683.722 | 110670.408 |  |  |
|  | Δ Adjusted BIC^h^ | S/R | N/A. | 12.381 | -5.032 | **-13.314** |  |  |
|  | Baseline factor loadings (unstandardized)^i^ | S/R | 9.541 to 24.596 | 10.738 to 23.262 | 10.733 to 23.271 | 10.707 to 23.223 |  |  |
|  | FU factor loadings (unstandardized)^i^ | S/R | 11.930 to 22.247 | 10.738 to 23.262 | 10.733 to 23.271 | 10.707 to 23.223 |  |  |
|  | Baseline factor loadings (standardized)^j^ | S/R | 0.280 to 0.905 | 0.312 to 0.883 | 0.312 to 0.884 | 0.312 to 0.876 |  |  |
|  | FU Factor loadings (standardized)^j^ | S/R | 0.345 to 0.879 | 0.314 to 0.868 | 0.314 to 0.868 | 0.312 to 0.876 |  |  |
|  | Additional correlation paths (baseline and FU)^k^ | S/R | BE1~~BE2 | | | |  |  |

^a^Structural equation models were specified using maximum likelihood estimation with robust (Huber-White) standard errors (MLR estimator) and imputation of missing data by full information maximum likelihood (FIML). Latent variables were measured from individual items on the experiment and survey as described in Supplementary Table S3.

^b^ꭓ^2^ = Chi-square Goodness of Fit statistic; df = degrees of freedom (ꭓ^2^ test); Δ = change in model fit index (between configural and weak/metric, between weak/metric and strong/scalar, or between strong/scalar and strict/residual); CFI = Comparative Fit Index; TLI = Tucker Lewis Index; RMSEA = Root Mean Square Error of Approximation; CI = confidence interval; SRMR = Standardized Root Mean Square Residual; AIC = Akaike Information Criterion; BIC = Bayesian Information Criterion; FU = follow-up.

^c^S = standard; R = robust. The "robust" version of the chi-square test produces a scaled test statistic that is asymptotically equal to the Yuan-Bentler test statistic (61).

^d^N/A = not applicable; SEM = structural equation model; MI = measurement invariance.

^e^Item AT2 removed due to poor factor loading.

^f^Factor loadings for two-item factors were constrained to be equal to achieve convergence, following the recommendations of Kenny et al., (1998) (60).

^g^Scaled chi-square difference tests (i.e., log-likelihood ratio tests) were calculated using the Satorra and Bentler (2001) method between the current column model and the preceding column model (i.e., between configural and weak/metric, between weak/metric and strong/scalar, or between strong/scalar and strict/residual) (31). Bold and underlined text indicates the preferred model according to the chi-square difference tests.

^h^Δ CFI, Δ TLI, Δ RMSEA, Δ SRMR, Δ AIC, Δ BIC, and Δ adjusted BIC were calculated as the change in the model fit index between the current column model and the preceding column model (i.e., between configural and weak/metric, between weak/metric and strong/scalar, or between strong/scalar and strict/residual). Where applicable, changes in model fit indices were calculated based on the robust version of the model fit index. Bold and underlined text indicates the preferred model according to the index.

^i^Unstandardized parameter values (std.lv, which standardizes all latent variables in the model only).

^j^Standardized parameter values (std.all, which standardizes all latent and observed variables in the model).

^k^Models which did not originally meet the cut-off criteria for all the model fit indices, were respecified including correlation paths between indicators on the same latent variable (which we would theoretically expect to be correlated). The same correlation paths were included for baseline and follow-up, and were fixed to be equal between baseline and follow-up. N/A = not applicable.

## Supplementary Table S8. Model fit statistics for partial longitudinal measurement invariance confirmatory factor analysis models, including data from MECHANISMS schools.

| **Outcome^a^** | **Model fit statistic^b^** | **Robust/ standard^c^** | **Configural^d^** | **Weak/Metric** | **Strong/Scalar** | **Strict/Residual** | **Measurement invariance present?^d^** | **Decision^d^** |
| --- | --- | --- | --- | --- | --- | --- | --- | --- |
| Experiment injunctive smoking norms (P2S2 to P2S6 and P2S9). | Observations | S/R | 1329 | 1329 | 1329 | 1329 | Partial Strict/Residual. | Run subsequent SEMs with partial strict/residual MI constraints included (free intercepts for P2S2, P2S4, P2S9). |
| No. of estimated parameters | S/R | 45 | 39 | 36 | 30 |
| χ^2^, df, p-value | S | 134.77, df=45, p<0.0001 | 153.23, df=51, p<0.0001 | 174.65, df=54, p<0.0001 | 192.21, df=60, p<0.0001 |
| R | 93.91, df=45, p<0.0001 | 101.12, df=51, p<0.0001 | 117.41, df=54, p<0.0001 | 117.64, df=60, p<0.0001 |
| Δ χ^2^, df, p-value^e^ | R | N/A. | **8.72, df=6, p=0.19** | 21.15, df=3, p<0.0001 | 5.95, df=6, p=0.43 |
| CFI | S | 0.971 | 0.967 | 0.960 | 0.957 |
| R | 0.977 | 0.975 | 0.969 | 0.969 |
| Δ CFI^f^ | R | N/A. | -0.002096 | -0.006145 | **0.000018** |
| TLI | S | 0.957 | 0.957 | 0.952 | 0.952 |
| R | 0.967 | 0.968 | 0.962 | 0.966 |
| Δ TLI^f^ | R | N/A. | 0.001191 | -0.005733 | **0.003792** |
| RMSEA (90% CI) | S | 0.039 (0.031, 0.046) | 0.039 (0.032, 0.046) | 0.041 (0.034, 0.048) | 0.041 (0.034, 0.047) |
| R | 0.035 (0.025, 0.046) | 0.034 (0.024, 0.045) | 0.037 (0.028, 0.047) | 0.036 (0.026, 0.045) |
| Δ RMSEA | R | N/A. | -0.000636 | 0.002964 | **-0.001933** |
| SRMR | S/R | 0.029 | 0.039 | 0.041 | 0.042 |
| Δ SRMR^f^ | S/R | N/A. | 0.010453 | 0.002041 | **0.001170** |
| AIC | S/R | 8886.924 | 8893.388 | 8908.807 | 8914.362 |
| Δ AIC^f^ | S/R | **N/A.** | 6.464 | 15.419 | 5.555 |
| BIC | S/R | 9120.572 | 9095.883 | 9095.726 | 9070.127 |
| Δ BIC^f^ | S/R | N/A. | -24.689 | -0.157 | **-25.599** |
| Adjusted BIC | S/R | 8977.627 | 8971.997 | 8981.370 | 8974.831 |
| Δ Adjusted BIC^f^ | S/R | N/A. | -5.630 | **9.373** | -6.539 |
| Baseline factor loadings (unstandardized)^g^ | S/R | 0.114 to 0.241 | 0.137 to 0.248 | 0.137 to 0.249 | 0.137 to 0.250 |
| FU factor loadings (unstandardized)^g^ | S/R | 0.162 to 0.255 | 0.137 to 0.248 | 0.137 to 0.249 | 0.137 to 0.250 |
| Baseline factor loadings (standardized)^h^ | S/R | 0.410 to 0.581 | 0.475 to 0.594 | 0.475 to 0.595 | 0.468 to 0.599 |
| FU Factor loadings (standardized)^h^ | S/R | 0.516 to 0.610 | 0.458 to 0.601 | 0.458 to 0.602 | 0.468 to 0.599 |
| Free factor loadings^i^ | S/R | All (P2S2 to P2S6, P2S9) | None (P2S2 to P2S6, P2S9 fixed) | None (P2S2 to P2S6, P2S9 fixed) | None (P2S2 to P2S6, P2S9 fixed) |
| Free intercepts^j^ | S/R | All (P2S2 to P2S6, P2S9) | All (P2S2 to P2S6, P2S9) | P2S2, P2S4, P2S9 | P2S2, P2S4, P2S9 |
| Free residual variances^k^ | S/R | All (P2S2 to P2S6, P2S9) | All (P2S2 to P2S6, P2S9) | All (P2S2 to P2S6, P2S9) | None (P2S2 to P2S6, P2S9 fixed) |
| Correlation paths^l^ | S/R | P2S2~~P2S3, P2S2~~P2S4 | | | |
| Self-efficacy (emotional; SEE1 to SEE9) | Observations | S/R | 1319 | 1319 | 1319 | 1319 | Partial Strict/Residual. | Run subsequent SEMs with partial strict/residual MI constraints included (free factor loadings for SEE1, SEE2, SEE6, SEE7). |
| No. of estimated parameters | S/R | 66 | 61 | 52 | 43 |
| χ^2^, df, p-value | S | 813.43, df=123, p<0.0001 | 825.42, df=128, p<0.0001 | 851.86, df=137, p<0.0001 | 888.12, df=146, p<0.0001 |
| R | 216.41, df=123, p<0.0001 | 221.04, df=128, p<0.0001 | 239.65, df=137, p<0.0001 | 241.15, df=146, p<0.0001 |
| Δ χ^2^, df, p-value^e^ | R | N/A. | **3.83, df=5, p=0.57** | 26.43, df=9, p=0.002 | 6.43, df=9, p=0.70 |
| CFI | S | 0.974 | 0.974 | 0.973 | 0.972 |
| R | 0.987 | 0.987 | 0.986 | 0.987 |
| Δ CFI^f^ | R | N/A. | 0.000119 | -0.000664 | **0.000469** |
| TLI | S | 0.968 | 0.969 | 0.970 | 0.971 |
| R | 0.984 | 0.985 | 0.985 | 0.986 |
| Δ TLI^f^ | R | N/A. | 0.000777 | 0.000274 | **0.001427** |
| RMSEA (90% CI) | S | 0.065 (0.061, 0.070) | 0.064 (0.060, 0.068) | 0.063 (0.059, 0.067) | 0.062 (0.058, 0.066) |
| R | 0.048 (0.037, 0.059) | 0.047 (0.036, 0.058) | 0.047 (0.036, 0.056) | 0.044 (0.034, 0.054) |
| Δ RMSEA | R | N/A. | -0.001166 | -0.000419 | **-0.002243** |
| SRMR | S/R | 0.021 | 0.049 | 0.053 | 0.054 |
| Δ SRMR^f^ | S/R | N/A. | 0.028501 | 0.004040 | **0.000953** |
| AIC | S/R | 35389.835 | 35391.818 | 35400.259 | 35418.522 |
| Δ AIC^f^ | S/R | N/A. | **1.983** | 8.441 | 18.263 |
| BIC | S/R | 35732.020 | 35708.080 | 35669.859 | 35641.461 |
| Δ BIC^f^ | S/R | N/A. | -23.940 | -38.221 | **-28.399** |
| Adjusted BIC | S/R | 35522.369 | 35514.312 | 35504.679 | 35504.870 |
| Δ Adjusted BIC^f^ | S/R | N/A. | -8.057 | -9.632 | **0.190** |
| Baseline factor loadings (unstandardized)^g^ | S/R | 0.776 to 0.846 | 0.807 to 0.895 | 0.808 to 0.897 | 0.808 to 0.897 |
| FU factor loadings (unstandardized)^g^ | S/R | 0.840 to 0.951 | 0.807 to 0.917 | 0.808 to 0.918 | 0.808 to 0.916 |
| Baseline factor loadings (standardized)^h^ | S/R | 0.828 to 0.907 | 0.842 to 0.914 | 0.843 to 0.914 | 0.843 to 0.903 |
| FU Factor loadings (standardized)^h^ | S/R | 0.856 to 0.917 | 0.842 to 0.911 | 0.843 to 0.911 | 0.843 to 0.907 |
| Free factor loadings^i^ | S/R | All (SEE1 to SEE9) | SEE1, SEE2, SEE6, SEE7 | SEE1, SEE2, SEE6, SEE7 | SEE1, SEE2, SEE6, SEE7 |
| Free intercepts^j^ | S/R | All (SEE1 to SEE9) | All (SEE1 to SEE9) | None (SEE1 to SEE9 fixed) | None (SEE1 to SEE9 fixed) |
| Free residual variances^k^ | S/R | All (SEE1 to SEE9) | All (SEE1 to SEE9) | All (SEE1 to SEE9) | None (SEE1 to SEE9 fixed) |
| Correlation paths^l^ | S/R | SEE1~~SEE2, SEE7~~SEE9 | | | |
| Self-efficacy (opportunity; SEO1 to SEO11) | Observations | S/R | 1319 | 1319 | 1319 | 1319 | Partial Strict/Residual.  Partial Strict/Residual MI is achieved based on the changes in the CFI, TLI, RMSEA, and SRMR. However, there is a large increase in the BIC between the strong/scalar and strict/residual models. | Run subsequent SEMs with partial strong/scalar MI constraints included (free factor loadings SEO1, SEO2, SEO3, SEO5, SEO6, SEO8, SEO9, SEO10, SEO11). |
| No. of estimated parameters | S/R | 83 | 81 | 70 | 59 |
| χ^2^, df, p-value | S | 1964.63, df=192, p<0.0001 | 1980.86, df=194, p<0.0001 | 1999.65, df=205, p<0.0001 | 2283.48, df=216, p<0.0001 |
| R | 338.53, df=192, p<0.0001 | 340.76, df=194, p<0.0001 | 360.02, df=205, p<0.0001 | 383.77, df=216, p<0.0001 |
| Δ χ^2^, df, p-value^e^ | R | N/A. | 2.41, df=2, p=0.30 | **18.97, df=11, p=0.06** | 21.30, df=11, p=0.03 |
| CFI | S | 0.951 | 0.951 | 0.951 | 0.943 |
| R | 0.977 | 0.977 | 0.976 | 0.972 |
| Δ CFI^f^ | R | N/A. | -0.000094 | -0.000220 | **-0.003970** |
| TLI | S | 0.942 | 0.942 | 0.945 | 0.939 |
| R | 0.972 | 0.972 | 0.973 | 0.970 |
| Δ TLI^f^ | R | N/A. | 0.000177 | 0.001247 | **-0.002891** |
| RMSEA (90% CI) | S | 0.084 (0.080, 0.087) | 0.084 (0.080, 0.087) | 0.081 (0.078, 0.085) | 0.085 (0.082, 0.088) |
| R | 0.060 (0.048, 0.071) | 0.059 (0.048, 0.070) | 0.058 (0.047, 0.069) | 0.061 (0.050, 0.072) |
| Δ RMSEA | R | N/A. | -0.000189 | -0.001344 | **0.003072** |
| SRMR | S/R | 0.042 | 0.071 | 0.073 | 0.075 |
| Δ SRMR^f^ | S/R | N/A. | 0.028505 | 0.002188 | **0.002092** |
| AIC | S/R | 24664.894 | 24677.128 | 24673.912 | 24935.745 |
| Δ AIC^f^ | S/R | **N/A.** | 12.234 | -3.216 | 261.832 |
| BIC | S/R | 25095.218 | 25097.083 | 25036.836 | 25241.638 |
| Δ BIC^f^ | S/R | N/A. | 1.864 | **-60.247** | 204.801 |
| Adjusted BIC | S/R | 24831.566 | 24839.783 | 24814.479 | 25054.222 |
| Δ Adjusted BIC^f^ | S/R | N/A. | 8.218 | **-25.305** | 239.743 |
| Baseline factor loadings (unstandardized)^g^ | S/R | 0.536 to 0.647 | 0.564 to 0.689 | 0.566 to 0.689 | 0.567 to 0.691 |
| FU factor loadings (unstandardized)^g^ | S/R | 0.643 to 0.749 | 0.607 to 0.716 | 0.608 to 0.717 | 0.607 to 0.721 |
| Baseline factor loadings (standardized)^h^ | S/R | 0.854 to 0.932 | 0.865 to 0.938 | 0.865 to 0.938 | 0.841 to 0.929 |
| FU Factor loadings (standardized)^h^ | S/R | 0.844 to 0.930 | 0.832 to 0.922 | 0.833 to 0.923 | 0.864 to 0.929 |
| Free factor loadings^i^ | S/R | All (SEO1 to SEO11) | SEO1, SEO2, SEO3, SEO5, SEO6, SEO8, SEO9, SEO10, SEO11 | SEO1, SEO2, SEO3, SEO5, SEO6, SEO8, SEO9, SEO10, SEO11 | SEO1, SEO2, SEO3, SEO5, SEO6, SEO8, SEO9, SEO10, SEO11 |
| Free intercepts^j^ | S/R | All (SEO1 to SEO11) | All (SEO1 to SEO11) | None (SEO1 to SEO11 fixed) | None (SEO1 to SEO11 fixed) |
| Free residual variances^k^ | S/R | All (SEO1 to SEO11) | All (SEO1 to SEO11) | All (SEO1 to SEO11) | None (SEO1 to SEO11 fixed) |
| Correlation paths^l^ | S/R | SEO1~~SEO3, SEO3~~SEO4, SEO4~~SEO11, SEO8~~SEO9, SEO8~~SEO10 | | | |
| Perceived physical risks (RP1 to RP7) | Observations | S/R | 1319 | 1319 | 1319 | 1319 | Partial Strict/Residual. | Run subsequent SEMs with partial strict/residual MI constraints included (free intercept for RP7). |
| No. of estimated parameters | S/R | 53 | 46 | 40 | 33 |
| χ^2^, df, p-value | S | 500.97, df=66, p<0.0001 | 509.67, df=73, p<0.0001 | 532.11, df=79, p<0.0001 | 566.20, df=86, p<0.0001 |
| R | 400.90, df=66, p<0.0001 | 421.53, df=73, p<0.0001 | 447.03, df=79, p<0.0001 | 477.14, df=86, p<0.0001 |
| Δ χ^2^, df, p-value^e^ | R | N/A. | **10.52, df=7, p=0.16** | 23.33, df=6, p=0.001 | 29.76, df=7, p=0.0001 |
| CFI | S | 0.950 | 0.950 | 0.948 | 0.945 |
| R | 0.951 | 0.951 | 0.949 | 0.946 |
| Δ CFI^f^ | R | N/A. | -0.000320 | -0.002019 | **-0.002989** |
| TLI | S | 0.931 | 0.938 | 0.940 | 0.942 |
| R | 0.932 | 0.938 | 0.941 | 0.942 |
| Δ TLI^f^ | R | N/A. | 0.006090 | 0.002351 | **0.001659** |
| RMSEA (90% CI) | S | 0.071 (0.065, 0.077) | 0.067 (0.062, 0.073) | 0.066 (0.061, 0.071) | 0.065 (0.060, 0.070) |
| R | 0.073 (0.066, 0.080) | 0.069 (0.063, 0.076) | 0.068 (0.062, 0.074) | 0.067 (0.061, 0.073) |
| Δ RMSEA | R | N/A. | -0.003349 | -0.001337 | **-0.000960** |
| SRMR | S/R | 0.046 | 0.050 | 0.052 | 0.054 |
| Δ SRMR^f^ | S/R | N/A. | 0.003811 | 0.001754 | **0.002115** |
| AIC | S/R | 160957.061 | 160951.757 | 160962.201 | 160982.293 |
| Δ AIC^f^ | S/R | N/A. | **-5.304** | 10.444 | 20.092 |
| BIC | S/R | 161231.847 | 161190.250 | 161169.586 | 161153.385 |
| Δ BIC^f^ | S/R | N/A. | -41.596 | -20.664 | **-16.201** |
| Adjusted BIC | S/R | 161063.490 | 161044.129 | 161042.525 | 161048.560 |
| Δ Adjusted BIC^f^ | S/R | N/A. | -19.361 | **-1.605** | 6.035 |
| Baseline factor loadings (unstandardized)^g^ | S/R | 18.850 to 25.077 | 17.630 to 24.764 | 17.561 to 24.812 | 17.577 to 24.822 |
| FU factor loadings (unstandardized)^g^ | S/R | 16.370 to 24.413 | 17.630 to 24.764 | 17.561 to 24.812 | 17.577 to 24.822 |
| Baseline factor loadings (standardized)^h^ | S/R | 0.614 to 0.771 | 0.588 to 0.767 | 0.585 to 0.768 | 0.591 to 0.776 |
| FU Factor loadings (standardized)^h^ | S/R | 0.571 to 0.778 | 0.599 to 0.782 | 0.597 to 0.783 | 0.591 to 0.776 |
| Free factor loadings^i^ | S/R | All (RP1 to RP7) | None (RP1 to RP7 fixed) | None (RP1 to RP7 fixed) | None (RP1 to RP7 fixed) |
| Free intercepts^j^ | S/R | All (RP1 to RP7) | All (RP1 to RP7) | RP7 | RP7 |
| Free residual variances^k^ | S/R | All (RP1 to RP7) | All (RP1 to RP7) | All (RP1 to RP7) | None (RP1 to RP7 fixed) |
| Correlation paths^l^ | S/R | RP1~~RP2, RP5~~RP6, RP6~~RP7 | | | |
| Perceived social risks (RS1 to RS3) | Observations | S/R | 1318 | 1318 | 1318 | 1318 | Partial Strict/Residual. | Run subsequent SEMs with partial strict/residual MI constraints included (free intercept for RS2). |
| No. of estimated parameters | S/R | 22 | 19 | 17 | 14 |
| χ^2^, df, p-value | S | 8.90, df=5, p=0.11 | 16.83, df=8, p=0.03 | 25.50, df=10, p=0.004 | 27.20, df=13, p=0.01 |
| R | 7.95, df=5, p=0.16 | 15.51, df=8, p=0.05 | 23.89, df=10, p=0.008 | 26.08, df=13, p=0.02 |
| Δ χ^2^, df, p-value^e^ | R | N/A. | **7.71, df=3, p=0.05** | 8.71, df=2, p=0.01 | 1.77, df=3, p=0.62 |
| CFI | S | 0.998 | 0.995 | 0.991 | 0.992 |
| R | 0.998 | 0.995 | 0.992 | 0.992 |
| Δ CFI^f^ | R | N/A. | -0.002690 | -0.003791 | **0.000626** |
| TLI | S | 0.993 | 0.991 | 0.987 | 0.991 |
| R | 0.994 | 0.991 | 0.987 | 0.991 |
| Δ TLI^f^ | R | N/A. | -0.002835 | -0.003942 | **0.003645** |
| RMSEA (90% CI) | S | 0.024 (0.000, 0.050) | 0.029 (0.008, 0.048) | 0.034 (0.018, 0.051) | 0.029 (0.013, 0.044) |
| R | 0.024 (0.000, 0.053) | 0.029 (0.000, 0.051) | 0.035 (0.017, 0.054) | 0.030 (0.012, 0.046) |
| Δ RMSEA | R | N/A. | 0.005194 | 0.005966 | **-0.005474** |
| SRMR | S/R | 0.014 | 0.027 | 0.029 | 0.033 |
| Δ SRMR^f^ | S/R | N/A. | 0.013220 | 0.002073 | **0.004029** |
| AIC | S/R | 71272.422 | 71274.354 | 71279.017 | 71274.721 |
| Δ AIC^f^ | S/R | N/A. | 1.932 | 4.663 | **-4.295** |
| BIC | S/R | 71386.467 | 71372.847 | 71367.142 | 71347.296 |
| Δ BIC^f^ | S/R | N/A. | -13.620 | -5.705 | **-19.847** |
| Adjusted BIC | S/R | 71316.583 | 71312.493 | 71313.141 | 71302.824 |
| Δ Adjusted BIC^f^ | S/R | N/A. | -4.090 | 0.648 | **-10.317** |
| Baseline factor loadings (unstandardized)^g^ | S/R | 20.203 to 24.846 | 19.745 to 23.053 | 19.733 to 23.074 | 19.738 to 23.154 |
| FU factor loadings (unstandardized)^g^ | S/R | 19.250 to 21.925 | 19.745 to 23.053 | 19.733 to 23.074 | 19.738 to 23.154 |
| Baseline factor loadings (standardized)^h^ | S/R | 0.568 to 0.754 | 0.560 to 0.716 | 0.559 to 0.717 | 0.560 to 0.727 |
| FU Factor loadings (standardized)^h^ | S/R | 0.552 to 0.694 | 0.561 to 0.734 | 0.560 to 0.735 | 0.560 to 0.727 |
| Free factor loadings^i^ | S/R | All (RS1 to RS3) | None (RS1 to RS3 fixed) | None (RS1 to RS3 fixed) | None (RS1 to RS3 fixed) |
| Free intercepts^j^ | S/R | All (RS1 to RS3) | All (RS1 to RS3) | RS2 | RS2 |
| Free residual variances^k^ | S/R | All (RS1 to RS3) | All (RS1 to RS3) | All (RS1 to RS3) | None (RS1 to RS3 fixed) |
| Correlation paths^l^ | S/R | N/A. | | | |
| Perceived addiction risks (RA1 to RA3) | Observations | S/R | 1317 | 1317 | 1317 | 1317 | Partial Strict/Residual. | Run subsequent SEMs with partial strict/residual MI constraints included (free intercept for RA2). |
| No. of estimated parameters | S/R | 22 | 19 | 17 | 14 |
| χ^2^, df, p-value | S | 18.87, df=5, p=0.002 | 24.65, df=8, p=0.002 | 30.54, df=10, p=0.001 | 34.15, df=13, p=0.001 |
| R | 18.36, df=5, p=0.003 | 25.40, df=8, p=0.001 | 31.22, df=10, p=0.001 | 38.92, df=13, p=0.0002 |
| Δ χ^2^, df, p-value^e^ | R | N/A. | 6.61, df=3, p=0.09 | 5.84, df=2, p=0.05 | **6.67, df=3, p=0.08** |
| CFI | S | 0.988 | 0.986 | 0.983 | 0.982 |
| R | 0.988 | 0.985 | 0.982 | 0.980 |
| Δ CFI^f^ | R | N/A. | -0.002505 | -0.003287 | **-0.001575** |
| TLI | S | 0.965 | 0.974 | 0.974 | 0.979 |
| R | 0.964 | 0.973 | 0.973 | 0.977 |
| Δ TLI^f^ | R | N/A. | 0.008960 | 0.000561 | **0.004389** |
| RMSEA (90% CI) | S | 0.046 (0.025, 0.069) | 0.040 (0.023, 0.058) | 0.039 (0.024, 0.056) | 0.035 (0.021, 0.050) |
| R | 0.049 (0.027, 0.075) | 0.043 (0.025, 0.062) | 0.042 (0.026, 0.060) | 0.039 (0.025, 0.053) |
| Δ RMSEA | R | N/A. | -0.006512 | -0.000441 | **-0.003622** |
| SRMR | S/R | 0.025 | 0.029 | 0.032 | 0.034 |
| Δ SRMR^f^ | S/R | N/A. | 0.004057 | 0.002184 | **0.002300** |
| AIC | S/R | 72249.206 | 72248.990 | 72250.872 | 72248.485 |
| Δ AIC^f^ | S/R | N/A. | -0.216 | 1.882 | **-2.386** |
| BIC | S/R | 72363.235 | 72347.469 | 72338.984 | 72321.049 |
| Δ BIC^f^ | S/R | N/A. | -15.766 | -8.484 | **-17.936** |
| Adjusted BIC | S/R | 72293.351 | 72287.115 | 72284.983 | 72276.577 |
| Δ Adjusted BIC^f^ | S/R | N/A. | -6.236 | -2.131 | **-8.406** |
| Baseline factor loadings (unstandardized)^g^ | S/R | 4.806 to 36.128 | 6.264 to 33.331 | 6.303 to 33.327 | 6.204 to 33.812 |
| FU factor loadings (unstandardized)^g^ | S/R | 7.702 to 32.475 | 6.264 to 33.331 | 6.303 to 33.327 | 6.204 to 33.812 |
| Baseline factor loadings (standardized)^h^ | S/R | 0.132 to 0.893 | 0.171 to 0.830 | 0.172 to 0.830 | 0.173 to 0.849 |
[truncated: 871,252 more chars]
